# Epiplasts: Membrane Skeletons and Epiplastin Proteins in Euglenids, Glaucophytes, Cryptophytes, Ciliates, Dinoflagellates, and Apicomplexans

**DOI:** 10.1128/mBio.02020-18

**Published:** 2018-10-30

**Authors:** Ursula Goodenough, Robyn Roth, Thamali Kariyawasam, Amelia He, Jae-Hyeok Lee

**Affiliations:** a Department of Biology, Washington University, St. Louis, Missouri, USA; b Center for Cellular Imaging, Washington University School of Medicine, St. Louis, Missouri, USA; c Department of Botany, University of British Columbia, Vancouver, British Columbia, Canada; Stanford University; Duke University; American Museum of Natural History; University of Cologne; University of Cologne; London School of Hygiene and Tropical Medicine

**Keywords:** electron microscopy, eukaryotic microalgae, evolution, membrane skeleton, protists

## Abstract

Animals and amoebae assemble actin/spectrin-based plasma membrane skeletons, forming what is often called the cell cortex, whereas euglenids and alveolates (ciliates, dinoflagellates, and apicomplexans) have been shown to assemble a thin, viscoelastic, actin/spectrin-free membrane skeleton, here called the epiplast. Epiplasts include a class of proteins, here called the epiplastins, with a head/medial/tail domain organization, whose medial domains have been characterized in previous studies by their low-complexity amino acid composition. We have identified two additional features of the medial domains: a strong enrichment of acid/base amino acid dyads and a predicted β-strand/random coil secondary structure. These features have served to identify members in two additional unicellular eukaryotic radiations—the glaucophytes and cryptophytes—as well as additional members in the alveolates and euglenids. We have analyzed the amino acid composition and domain structure of 219 epiplastin sequences and have used quick-freeze deep-etch electron microscopy to visualize the epiplasts of glaucophytes and cryptophytes. We define epiplastins as proteins encoded in organisms that assemble epiplasts, but epiplastin-like proteins, of unknown function, are also encoded in Insecta, Basidiomycetes, and *Caulobacter* genomes. We discuss the diverse cellular traits that are supported by epiplasts and propose evolutionary scenarios that are consonant with their distribution in extant eukaryotes.

## Abstract (precis)

Membrane skeletons associate with the inner surface of the plasma membrane to provide support for the fragile lipid bilayer and an elastic framework for the cell itself. Several radiations, including animals, organize such skeletons using actin/spectrin proteins, but four major radiations of eukaryotic unicellular organisms, including disease-causing parasites such as *Plasmodium*, have been known to construct an alternative and essential skeleton (the epiplast) using a class of proteins that we term epiplastins. We have identified epiplastins in two additional radiations and present images of their epiplasts using electron microscopy. We analyze the sequences and secondary structure of 219 epiplastins and present an in-depth overview and analysis of their known and posited roles in cellular organization and parasite infection. An understanding of epiplast assembly may suggest therapeutic approaches to combat infectious agents such as *Plasmodium* as well as approaches to the engineering of useful viscoelastic biofilms.

## INTRODUCTION

Two general strategies have evolved for stabilizing the surfaces of cells. The most widespread is the secretion and self-assembly of a cell wall exterior to the plasma membrane. The second, and our focus here, is the assembly of a membrane skeleton beneath the plasma membrane, giving the plasma membrane direct access to the external environment via intrinsic and extrinsic glycoproteins (often designated the “glycocalyx”).

In animals and Amoebozoa, the membrane skeleton, commonly called the cell cortex, consists of actin filaments that associate with the membrane and one another via actin-binding proteins (1), often accompanied by a spectrin network (2, 3), an association that is malleable during endocytosis/exocytosis and amoeboid movement.

**FIG 1:**
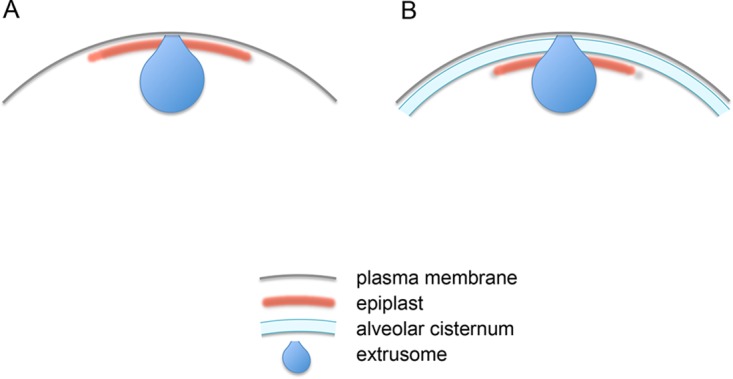
Epiplast configurations. (A) Direct association with plasma membrane, found in euglenids and cryptophytes. (B) Direct association with alveolar membranes, which in turn directly associate with the plasma membrane, found in alveolates and glaucophytes.

Several lineages of eukaryotic microbes, however, assemble a thin membrane skeleton that lacks an actin/spectrin component and maintains its integrity when cells are treated with nonionic detergent (4–9). In euglenids and cryptophytes, the skeleton makes direct contact with the plasma membrane (Fig. 1A), while in members of the Alveolata superphylum (ciliates, dinoflagellates, and apicomplexans) and in glaucophytes, a layer of membranous cisternae, called alveoli, lies beneath the plasma membrane, and the membrane skeleton then makes direct contact with the inner alveolar membrane (Fig. 1B).

In euglenids, this skeleton has been called the dense fibrillary layer (10), the submembranous layer (11), or the membrane (cyto)skeleton (12, 13), and identified protein components are called articulins (14). The layer is called the inner periplast component in cryptophytes (15), and candidate protein components are reported in this study. In ciliates, the skeleton is called the epiplasm or epiplasmic layer (16–19), and associated proteins include articulins (20, 21), alveolins (22–24), and epiplasmins (25–29). It is called the pellicular layer in dinoflagellates (30), and alveolin-class components have been identified (22). In apicomplexans, it is called the subpellicular network (8, 31), and components include alveolins/IMC proteins (22, 23, 32–36). Here we report that such a membrane skeleton also localizes beneath the alveoli of *Cyanophora*, a glaucophyte alga, and we identify its candidate protein components.

We present data indicating that these non-actin-based membrane skeletons are structurally related to one another and that the proteins listed above are members of a single class. This leads us to propose a single name for the membrane skeleton and a single name for the protein class. We designate the submembranous domains “epiplasts” (Gr. plastós: formed, molded), honoring the epiplasm terminology of protist pioneer E. Fauré-Fremiet (16, 37), and designate the protein class the “epiplastins,” where, as detailed in this report, epiplastins exhibit three features: a distinctive low-complexity medial domain (center of the polypeptide sequence); a predicted predominance of β-strand secondary structure; and inclusion in the genomes of organisms that construct epiplasts. We do not expect these terms to replace the current lineage-specific names, as those are well embedded in the literature; rather, the collective terms are intended to facilitate their general consideration.

As detailed in Results, epiplasts adopt two configurations; they usually form filamentous meshworks, but in some lineages the proteins pack so tightly together that they form homogeneous plates, commonly linked to one another like armor, with filamentous edges. The filaments often make patterned contacts with their overlying membranes, in some cases participating in the generation of sculpted surface features, and zones of membrane contact are often marked by patterned arrays of intramembranous particles (IMPs) or by zones devoid of IMPs. We review published ultrastructural features of epiplasts and present new observations utilizing quick-freeze deep-etch electron microscopy (QFDEEM).

While epiplastins may not be the sole components of a given epiplast (38–41), many have been localized to epiplasts using antibodies or fluorescent tags in euglenids (42), in ciliates (24, 25, 28, 43–46), and in apicomplexans (8, 32, 33, 36, 47–51) (see reference 52 for a review of apicomplexan studies). In several of those reports, the proteins were localized to particular cellular regions and/or shown to be expressed in particular life cycle stages. Moreover, mutation of individual epiplastin genes results in often-severe morphological defects and inviability in ciliates (24, 29, 53) and in apicomplexans (47, 50, 51, 54–56). Since each genome encodes multiple epiplastins, the fact that defects in a single gene can have strong morphogenetic consequences indicates that the proteins are not, in general, functionally redundant.

Previous studies, detailed in Results, have established that each epiplastin carries a medial domain, of varying length, that is enriched in a small subset of amino acids (the most frequently noted being V, E, I, K, Q, and P) and flanked by N-terminal (N-term) (head) and C-terminal (C-term) (tail) domains with full amino acid representation. The medial low-complexity domain in the widespread articulin subclass is enriched in the motif VPV, but in general, the sequences do not display constrained “repeat motifs” in the fashion of the GPX units of collagens (57) or the SPPPP units of plant cell wall proteins (58).

We did, however, notice a pattern. The medial domains of the epiplastins include numerous acid-base dyads (ABDs) (e.g., EK, KE, and DR) separated by intervals (“strings”) of other amino acids in the low-complexity subset (e.g., …EKVVIDRIPVIPQVREPK…), leading us to call them ABD domains. The ABD hallmark has facilitated searches for epiplastins in genomic/transcriptomic databases. It has also guided the delineation of the boundaries and organization of the medial domains more consistently than is possible using general descriptions of amino acid composition such as “charged repeat motifs” (23).

We also analyzed the predicted secondary structure of epiplastins and found that while the head and tail domains include α-helices and occasionally coiled-coils, the ABD domains are, with a few exceptions found largely in the cryptophytes, scored as adopting β-strand and/or random coil conformations and devoid of α-helices. Hence, epiplastins do not share structural homology with intermediate-filament proteins, whose hallmark is a medial α-helical coiled-coil. Moreover, the only intermediate-filament proteins reported to form a membrane skeleton are the nuclear lamins (59). Therefore, referring to epiplastins as intermediate filaments or intermediate filament-like (24, 27, 32, 36, 51, 60–62) can be misleading. Rather, the epiplastins represent a novel protein class that proves to be restricted, with a few interesting exceptions, to lineages that assemble epiplasts, a restriction that may be informative in assigning evolutionary relationships between various taxa.

## RESULTS

### Epiplastins: database searches, amino acid organization, and predicted secondary structure. (i) Database searches.

This project initiated with a genomic/ultrastructural study of Cyanophora paradoxa, a unicellular alga in the glaucophyte lineage that is commonly posited to have branched early in the radiation leading to red and green algae and land plants. Glaucophytes had previously been shown to assemble a system of submembranous cisternae that resemble the alveoli of alveolates, an unexpected trait given that glaucophytes and alveolates are distantly separated in phylogenomic trees (63, 64) and a finding that led Cavalier-Smith (65, 66) to propose a protoalveolate as the host for the primary endosymbiotic event that led to the cyanobacterium → chloroplast transition.

We proceeded to identify, in newly available glaucophyte genomes and transcriptomes (D. C. Price, U. W. Goodenough, R. Roth, J.-H. Lee, T. Kariyawasam, M. Mutwil, C. Ferrari, F. Facchinell, S. G. Ball, U. Cenci, C. X. Chan, H. S. Yoon, A. P. M. Weber, and D. Bhattacharya, submitted for publication), several proteins with epiplastin hallmarks, and this led to three rounds of genomic/transcriptomic database searches for proteins with similar characteristics in other radiations. Text S1 to S7 in the supplemental material display the 219 epiplastins identified in this study, categorized by lineage, showing their ABD domain organization and examples of secondary structure predictions. Table S1 in the supplemental material lists the genome resources queried and summarizes the bioinformatics data according to species; Table S2 provides profiles of the ABD amino acid compositions and any conserved motifs for each protein analyzed. Text S8 lists proteins, identified in other studies as (putatively) localized to cell surface domains, that do not meet the criteria for epiplastin designation described below.

10.1128/mBio.02020-18.1 TEXT S1 Apicomplexans, parasite epiplastin sequences. Download Text S1, PDF file, 1.6 MB. Copyright © 2018 Goodenough et al. 2018 Goodenough et al. This content is distributed under the terms of the Creative Commons Attribution 4.0 International license.

10.1128/mBio.02020-18.2 TEXT S2 Apicomplexans, photosynthetic epiplastin sequences. Download Text S2, PDF file, 0.8 MB. Copyright © 2018 Goodenough et al. 2018 Goodenough et al. This content is distributed under the terms of the Creative Commons Attribution 4.0 International license.

10.1128/mBio.02020-18.3 TEXT S3 Ciliate epiplastin sequences. Download Text S3, PDF file, 1.3 MB. Copyright © 2018 Goodenough et al. 2018 Goodenough et al. This content is distributed under the terms of the Creative Commons Attribution 4.0 International license.

10.1128/mBio.02020-18.4 TEXT S4 Cryptophyte epiplastin sequences. Download Text S4, PDF file, 4.0 MB. Copyright © 2018 Goodenough et al. 2018 Goodenough et al. This content is distributed under the terms of the Creative Commons Attribution 4.0 International license.

10.1128/mBio.02020-18.5 TEXT S5 Dinoflagellate epiplastin sequences. Download Text S5, PDF file, 1.0 MB. Copyright © 2018 Goodenough et al. 2018 Goodenough et al. This content is distributed under the terms of the Creative Commons Attribution 4.0 International license.

10.1128/mBio.02020-18.6 TEXT S6 Euglenid epiplastin sequences. Download Text S6, PDF file, 1.0 MB. Copyright © 2018 Goodenough et al. 2018 Goodenough et al. This content is distributed under the terms of the Creative Commons Attribution 4.0 International license.

10.1128/mBio.02020-18.7 TEXT S7 Glaucophyte epiplastin sequences. Download Text S7, PDF file, 0.5 MB. Copyright © 2018 Goodenough et al. 2018 Goodenough et al. This content is distributed under the terms of the Creative Commons Attribution 4.0 International license.

10.1128/mBio.02020-18.8 TEXT S8 Nonepiplastin sequences. Download Text S8, PDF file, 1.7 MB. Copyright © 2018 Goodenough et al. 2018 Goodenough et al. This content is distributed under the terms of the Creative Commons Attribution 4.0 International license.

10.1128/mBio.02020-18.13 TABLE S1 Genome resources and epiplastin summary. Download Table S1, DOCX file, 0.1 MB. Copyright © 2018 Goodenough et al. 2018 Goodenough et al. This content is distributed under the terms of the Creative Commons Attribution 4.0 International license.

10.1128/mBio.02020-18.14 TABLE S2 ABD domain sequence characteristics. Download Table S2, XLSX file, 0.1 MB. Copyright © 2018 Goodenough et al. 2018 Goodenough et al. This content is distributed under the terms of the Creative Commons Attribution 4.0 International license.

We first conducted a Pfam search of numerous eukaryotic genomes and transcriptomes using IMCp (see Materials and Methods) (Table S1) and recovered strong candidates from glaucophytes, euglenids, cryptophytes, and alveolates, whereas none were recovered from the other eukaryotic groups queried, including red and green algae, animals and their sister lineages, fungi, Amoebozoa, Rhizaria, stramenopiles, haptophytes, and noneuglenid Excavates. We also included for consideration candidate epiplastins found in the bacterium *Caulobacter* (36).

Given that the Pfam domain is based on apicomplexan sequences, we next conducted searches for low-complexity regions (LCRs) (see Materials and Methods) with epiplastin characteristics (described below) that yielded additional epiplastin candidates in other radiations. The following results give a sense of the scope of the two searches: the Pfam search yielded 20 *Eutreptiella* (euglenid) epiplastins, while the LCR-based search recovered 18/20 of these plus an additional 24 proteins, for a total of 42 proteins.

Finally, we conducted a BLAST search (NCBI) using the ABD domain sequence of the cryptophyte *Chroomonas* MMETSP0047_c25199_g1_i1_g48336 protein (see Fig. 8 in Text S4 [cited as Text S4.8; i.e., Text S#.Fig. #]) and recovered two additional groups of epiplastin-like proteins, one restricted to Basidiomycetes and the other to Insecta (Text S10.16 to S10.26).

#### (ii) Identifying ABD domains by amino acid sequence.

**FIG 2:**
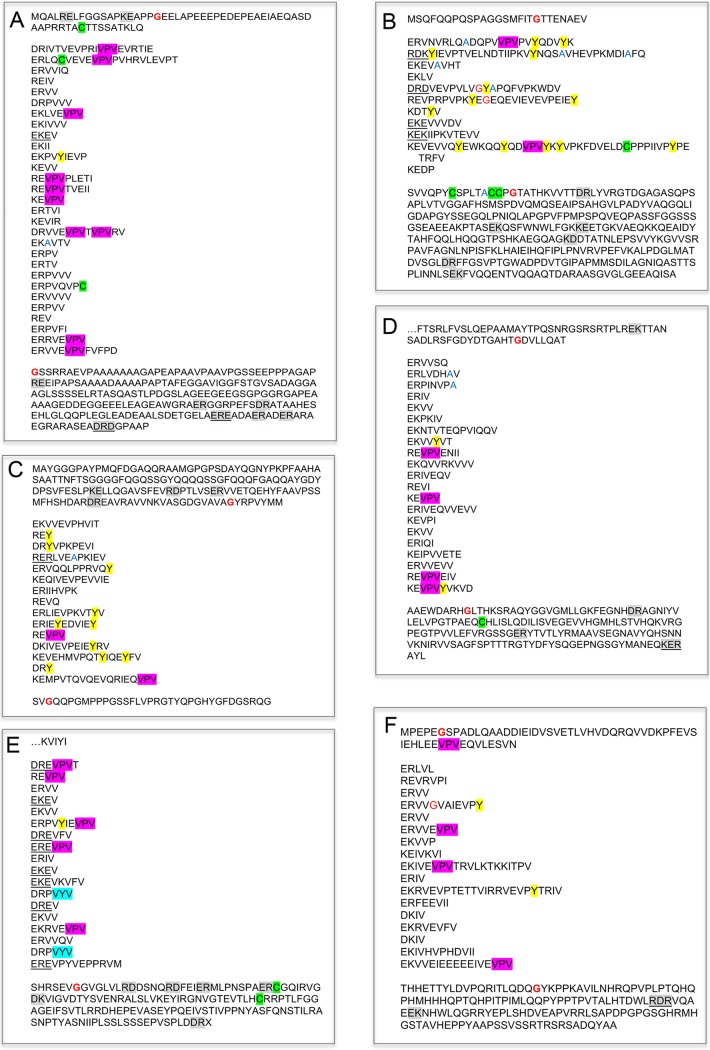
Representative epiplastins with medial ABD domains “parsed” into “strings.” (A) *Cyanophora* (glaucophyte) (Text S7.6). (B) *Toxoplasma* IMC10 (apicomplexan) (Text S1.41). (C) *Kryptoperidinium* (dinoflagellate) (Text S5.15). (D) *Chroomonas* (cryptophyte) (Text S4.12). (E) *Goniomonas* (cryptophyte) with VYV modules (Text S4.20). (F) *Eutreptiella* (euglenid) (Text S6.33).

Fig. 2 displays examples of epiplastin amino acid sequences from several lineages, “parsed” to display their ABD domains and the amino acid strings that separate each dyad. A full set of parsed epiplastin sequences is found in Text S1 to S7.

ABD domains were characterized using the following criteria:

- Acid-base dyads. Dyads utilize E>D and K>R and more commonly initiate with an acid than with a base. One also encounters triads (e.g., EKE) and, infrequently, tetrads (e.g., EKER). Acid-base dyads are occasionally found in the head (N-terminal) and tail (C-terminal) domains of the epiplastins as well (shaded gray in Fig. 2 and Text S1 to S7) but with greatly reduced frequency compared with the ABD domains and in a very different amino acid context.
- Short intervals (strings) separating the dyads. Dyads are almost always separated by at least two amino acids and are usually flanked by V or I residues.
- Quantitation of the amino acid compositions of each ABD domain is provided in Table S2. Rounded amino acid percentiles are given below, with means ± standard deviations (SD) and medians given in parentheses; the close mean/median correspondences and small SDs point to the uniformity of this profile. For comparison, published amino acid percentiles for vertebrate proteins (http://www.tiem.utk.edu/~gross/bioed/webmodules/aminoacid.htm) are given in brackets.
  - A strong enrichment for hydrophobic amino acids V (25% ± 7%; 25%) [6.8%] and I (9% ± 4%; 8%) [3.8%] but a much weaker enrichment for L (2% ± 2%; 2%) [7.6%], often occurring in clusters (VV, VVI). The mean level of V+I content is 33% ± 7% (35%) [18.2%].
  - An enrichment for charged amino acids (35% ± 6%; 36%) [23.1%], with a preference for E (16% ± 5%; 16%) and K (9% ± 4%; 8%) over D (3% ± 2%; 3%) and R (7% ± 3%; 7%). The mean charged content is 35% ± 6%; 36% [23.1%], and the net charge (Table S2, column E) is almost always either nearly neutral or negative (−9 ± 13; −7).
  - A variable endowment of Y (shaded yellow in Fig. 2 and Text S1 to S7; Table S2, column AB), C (shaded green in Fig. 2 and Text S1 to S7; Table S2, column J), Q (Table S2, column V), and P (Table S2, column U), ranging from absent to prominent. Species-specific patterns are noted in subsequent sections.
  - A near-exclusion of G residues (0.4% ± 0.7%; 000%) [7.4%] (red font in Fig. 2 and Text S1 to S7; Table S2, column N) and A residues (1% ± 1%; 2%) [7.4%] (blue font in Fig. 2 and Text S1 to S7; Table S2, column I), and a low density of S (1% ± 2%; 1%) [8.1%] and T (3% ± 2%; 3%) [6.2%] residues (Table S2, columns X and Y). By contrast, the head and tail domains flanking the ABD domains have abundant G, A, S, and T endowments (Fig. 2 and Text S1 to S7), a criterion used to mark ABD domain boundaries. While these boundaries were determined by subjective evaluation and therefore cannot be considered precise, the boundaries in our *Toxoplasma* ABD domain set match closely the boundaries determined for *Toxoplasma* low-complexity regions based on independent criteria (32).
- Predicted coiled-coil (C-C) domains are present within two ABD domains: one in *Chromera* (Table S2, row 17) and one in *Goniomonas* (row 99). In 14 cases, and notably in *Goniomonas*, a coiled-coil domain predicted for the head (nt) or tail (ct) extends into the ABD domain (Table S2, rows 88, 90, 92, 93, 96, 97, 104, 150, 151, 168, 183, 205, 208, and 213).
- No general sequence preferences are evident except the VPV enrichment in articulins (see below). As expected for any low-complexity protein domain, repeated combinations are encountered, and in some instances (noted in Text S1 to S7) they appear to represent iterations derived from endoreduplication; such repeats are specifically noted in *Plasmodium* (36). That said, what seems to be important is the restricted amino acid composition itself and not repeats of particular motifs.

#### (iii) Identifying ABD domains by secondary structure.

**FIG 3:**
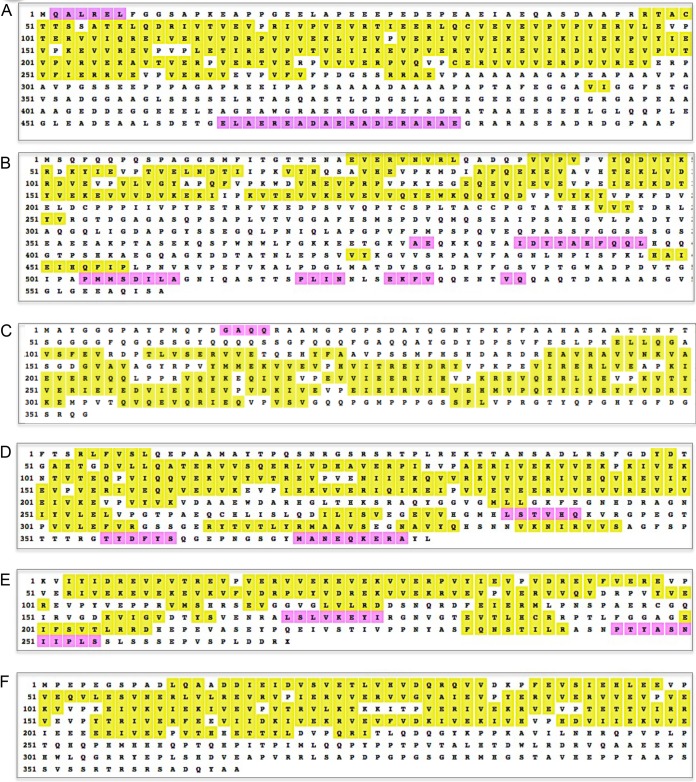
Secondary structure predictions for epiplastins shown in Fig. 2. Yellow highlight, β-strands; magenta highlight, α-helices.

Secondary structure algorithms (see Materials and Methods) predict that ABD domains are generally (i) dominated by long β-strands, presumably interacting to form β-sheets, interspersed with regions of random coil, and (ii) devoid of α-helices. Figure 3 displays examples; many others are found in Text S1 to S7.

While these predictions await biophysical confirmation (e.g., nuclear magnetic resonance [NMR] and circular dichroism [CD] spectroscopy), they have served as an important second criterion for epiplastin identification, particularly in ambiguous cases. As an example, four proteins in cytoskeletal preparations of the parabasalid Trichomonas vaginalis and a family of pox-virus A-like proteins were scored as “charged low complexity” and proposed as members of the family that includes alveolins (23, 67). The proteins, while rich in charged amino acids and hence amenable to being parsed into ABD strings, lack the amino acid profile of epiplastins, and, when queried, all were predicted to be fully α-helical (Text S8.4 to S8.10). Interestingly, a fifth protein in the *Trichomonas* collection is predicted to be fully β-stranded (Text S8.23), although its amino acid profile is totally different from the epiplastin profile.

#### (iv) Articulins.

Marrs and Bouck (14) first noticed an abundance of VPV motifs in epiplast-derived proteins from *Euglena* that they named articulins, and Huttenlauch et al. (20) found similar proteins in a ciliate and proposed that the VPV motif is unique to articulins (21). While it might be expected that ABD domains enriched in V and P would include VPV modules on a regular basis, in fact, the triad is infrequently encountered (in *Toxoplasma* and *Plasmodium*, the median is one per ABD domain), except in articulins, where multiple VPV iterations are found (highlighted in pink in Fig. 2 and Text S1 to S7). Occasional VPVPV units are found but never VPVVPV. We arbitrarily elected to score proteins with >3 VPV modules in their ABD domains as indicating membership in the articulin subclass, and articulins were identified in all the taxa sampled in our study except *Tetrahymena*.

#### (v) Head and tail domains.

Most ABD domains are flanked by head (N-term) and tail (C-term) domains. In a few cases, noted in Text S1 to S7, their sequences are orthologous to other epiplastin proteins in a given genome, presumably as a consequence of endoreduplication, but in general they are very different from one another, although they are sometimes enriched in lineage-specific amino acids (e.g., N-rich in *Plasmodium*, Q-rich in *Paramecium*, and G-rich in euglenids). Columns F and H in Table S2 list predicted protein motifs and coiled-coil domains in head and tail domains.

#### Presentation of results.

Below, we first consider the epiplast structure and epiplastin endowments of *Cyanophora* and members of the Alveolata, where the epiplast lies beneath alveolar cisternae (Fig. 1B). We then consider euglenids and cryptophytes, where the epiplast is directly contiguous to the plasma membrane (Fig. 1A). Finally, we consider five cases in which epiplastin-like proteins have been identified in organisms not known to construct an epiplast. Features specific to each lineage are presented in Results; more general patterns are considered in Discussion.

### The alveoli and epiplast of *Cyanophora*.

**FIG 4:**
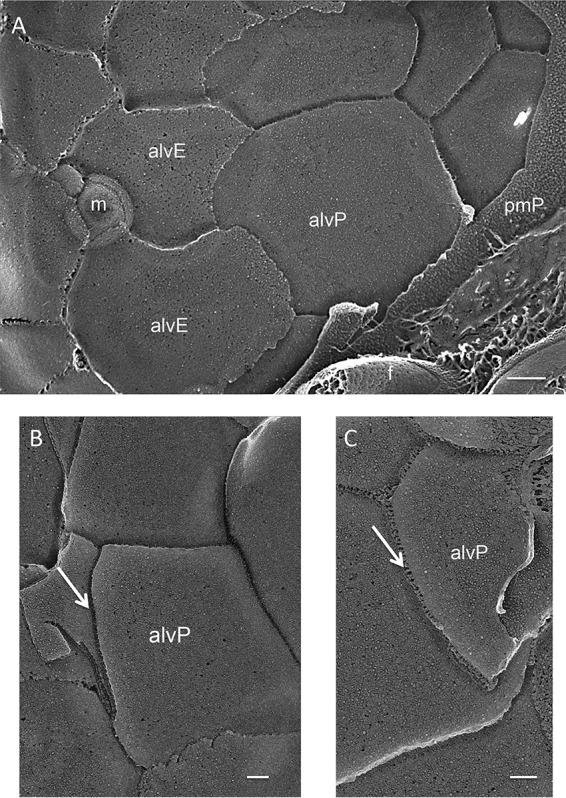
Alveolar cisternae of glaucophyte *Cyanophora paradoxa*. (A) Overview. alvP, P fracture face of alvelolar membrane; alvE, E fracture face of alveolar membrane; pmP, P fracture face of plasma membrane; m, mucocyst. (B and C) Delicate and wispy (B) or more robust (C) sutures interconnecting the alveoli (arrows). Scale bars: A, 500 nm; B and C, 100 nm.

Several investigators have described the overlapping alveolar cisternae in the glaucophyte algae (68–74) and have noted their similarity to the alveoli found in alveolates (65, 71, 74, 75). Figure 4 shows the *Cyanophora* system using QFDEEM. Figure 4A displays the two fracture faces of the cisternal membranes—alvP (plasma membrane- or cytoplasm contiguous) and alvE (cisternal lumen contiguous)—with a mucocyst (m) at the cisternal boundaries. The alveolar sutures that interconnect the cisternal membranes can appear wispy (Fig. 4A and B) or more robust (Fig. 4C).

**FIG 5:**
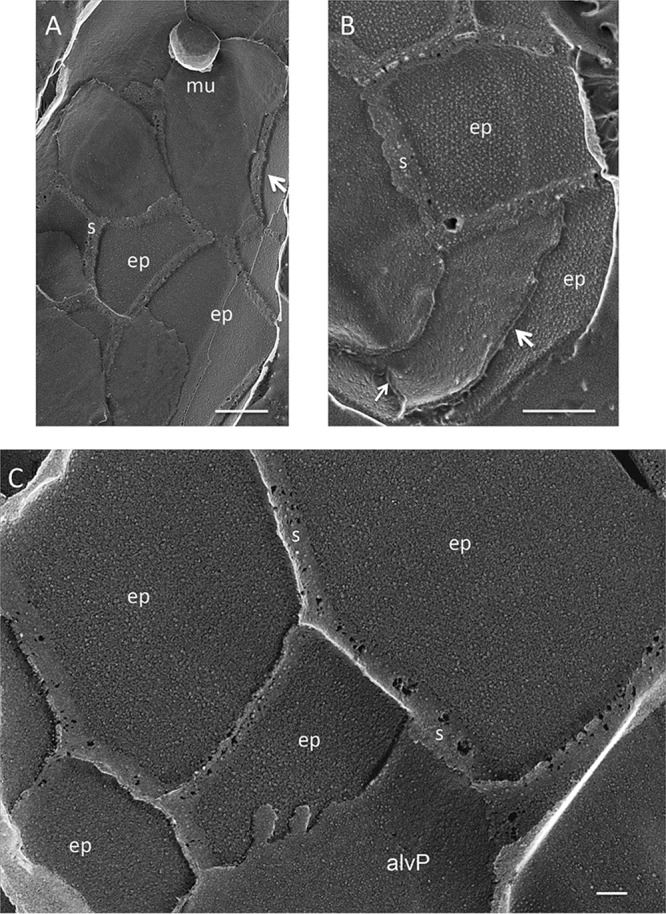
Epiplast of glaucophyte *Cyanophora paradoxa*. ep, epiplast plates connected by thick sutures (s). Arrows, views of the cisternal lumens. mu, mucoplast in suture domain. Scales bars: A and B, 500 nm; C, 100 nm.

Figure 5 and Text S9.2 to S9.4 document that this alveolar system is supported by a system of interconnected epiplastic plates (ep), The plates have the same dimensions as the cisternae and also often carry mucocysts at their boundaries (Text S9.2 and S9.3). They are interconnected by sutures that underlie the alveolar sutures but are broad and gummy-looking: these presumably correspond to the polygonal surface ridges seen with scanning electron microscopy (SEM) (72, 73). Striations and aligned small particles endow the plates with a semicrystalline appearance. The cisternae are subtended by a system of microtubules (69).

10.1128/mBio.02020-18.9 TEXT S9 Figures. Download Text S9, PDF file, 7.5 MB. Copyright © 2018 Goodenough et al. 2018 Goodenough et al. This content is distributed under the terms of the Creative Commons Attribution 4.0 International license.

10.1128/mBio.02020-18.10 TEXT S10 Epiplastin-like protein sequences. Download Text S10, PDF file, 2.0 MB. Copyright © 2018 Goodenough et al. 2018 Goodenough et al. This content is distributed under the terms of the Creative Commons Attribution 4.0 International license.

The location of the epiplast plates is as yet unresolved. Kugrens et al. (71) noted a thin layer of material residing within the cisternae in fixed thin-sectioned images and suggested that it corresponds to the plates seen with freeze fracture, an interpretation also offered by Heimann et al. (68), whereas we saw no material within the lumen (Fig. 5A and B, arrows) and therefore interpret the epiplast system to lie beneath the alveoli.

Inspection of stationary-phase cultures of *Cyanophora* by phase microscopy revealed the presence of cell-sized refractile profiles, many aggregated in the medium (Text S9.6). *Cyanophora* lacks a cell wall; hence, these “shells” represented candidate discarded epiplasts, much like those visualized when *Cyanophora* is subjected to osmotic shock (68) (see Fig. 6 in ref. 68). Since epiplasts have been shown to be stable in the presence of nonionic detergent exposure in several other lineages (see below), generating cell “ghosts,” we asked whether this is also the case for *Cyanophora* epiplasts. As shown in Text S9.7 and S9.8, cells exposed to even high (5%) concentrations of NP-40 detergent retain phase-refractile boundary material. The chloroplasts, encased in a layer of peptidoglycan, are themselves impermeable with respect to detergent and retain their pigments, but other cellular contents are solubilized (Text S9.9).

### The epiplastins of glaucophytes.

We identified five epiplastins in the Cyanophora paradoxa genome and two each in transcriptomes of glaucophytes Cyanoptyche gloeocystis and Gloeochaete witrockiana (Text S7). Their ABD domains all carry VPV motifs, and five carry >3 VPVs and are therefore classified as articulins. All are poor in or devoid of Y compared with most other lineages. One head domain is predicted to adopt a coiled-coil configuration (Table S2, row 51).

### The alveoli and epiplast of alveolates: parasitic apicomplexans.

The alveolar cisternae of the parasitic apicomplexans, called the inner membrane complex (IMC), are separated from the plasma membrane by proteins that function in actin/myosin-based gliding motility (76) (considered more fully in Discussion). The cisternae are interconnected by sutures that resemble those in *Cyanophora*, where a single suture bridges two large cisternae in *Plasmodium* sporozoites (77–79) and multiple sutures bridge smaller cisternae in *Plasmodium* gametocytes and in *Toxoplasma* (41, 80–82) and *Eimeria* (83). Several alveolar membrane proteins have been identified in *Toxoplasma* (39–41, 84).

The P-fracture faces of the inner (cytoplasm-facing) alveolar membranes display two sets of aligned intramembranous particles (IMPs) (76, 77, 81–83, 85, 86). The rows containing paired IMPs lie above the underlying microtubules, while the more numerous single-IMP rows are ∼30 nm apart (81) (Text S9.10 and S9.11). Morrissette and Sibley (87) hypothesize that the single-IMP rows are entrained by the underlying epiplast, a hypothesis supported by our studies (see below).

**FIG 6:**
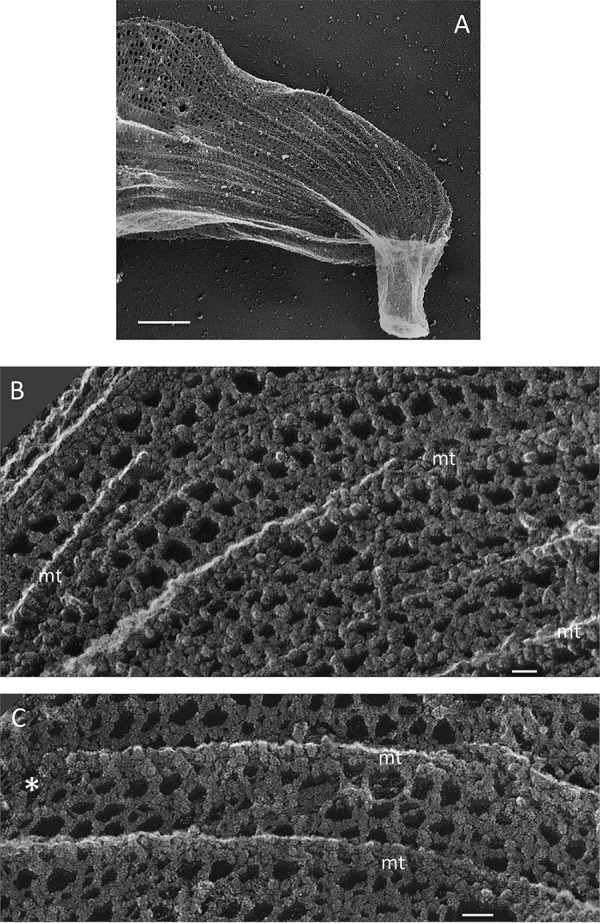
Ghosts of *Toxoplasma gondi* apicomplexans. (A) Overview. (B and C) Epiplast lattice between microtubules (mt). Asterisk, region of angled cross-bridges. (Stereo images are provided in Text S9.12 and 9.13.) Scale bars: A, 1 μm; B and C, 25 nm.

In thin-section transmission electron microscopy (TEM), the native apicomplexan epiplast appears as a fluffy density beneath the inner alveolar membrane (see Fig. 5 in reference 32, Fig. 21 in reference 82, Fig. 1 in reference 83, Fig. 1 in reference 88, and Fig. 6 in reference 89).

D’Haese et al. (6) first showed that after several parasitic apicomplexans were subjected to nonionic detergent extraction, the resultant cell ghosts retained their full-length cellular shape even though in some cases the cortical microtubules extended for only half the length of the cells, suggesting the presence of an additional skeletal component (see also references 34 and 90). Mann and Beckers (8) made similar ghost preparations using *Toxoplasma* and detected a filamentous meshwork between the microtubules that they termed the subpellicular network and that we designate here the epiplast.

Figure 6A shows a freeze-dried Toxoplasma gondii ghost, prepared as described by Heuser and Kirschner (91); Fig. 6B and C show details of its filamentous meshwork (stereo images in Text S9.13 and S9.14; see also reference 87). Longitudinal microtubules (mt) are intercalated with longitudinal filaments, ∼5 nm in diameter and spaced ∼30 nm apart; these are cross-bridged at right angles by 5-nm filaments, also spaced at ∼30 nm, to form square-shaped units. In some fields (Fig. 6C, arrows), the cross-bridges are instead angled 30° from the longitudinal, a shift, perhaps generated during specimen preparation, which indicates a flexibility that may contribute to the elasticity of the apicomplexan cell surface (81) (see Discussion). The longitudinal filaments are likely to be components of the “tracks” that undergird actin/myosin-based gliding motility of the apicomplexans (see Discussion).

### The epiplastins of parasitic apicomplexans.

The genome of Toxoplasma gondii encodes 14 epiplastins, denoted IMCs, all previously identified by others (Text S1.31 to S1.45). Five proteins annotated as IMCs do not display the amino acid profile of epiplastins (Text S8.2 and S8.15 to S8.17). The 16 epiplastins from *Plasmodium falciparum* were compiled by researchers in the Dessens laboratory (50) (Text S1.14 to S1.30), and a sampling of 12 sequences from other apicomplexan parasites (e.g., *Cryptosporidium* and *Cyclospora*) was collected from the NCBI database (Text S1.2 to S1.13). As noted in the introduction, many of the epiplastins of the apicomplexans have been localized to the cell periphery; gene disruption generates aberrant cell shape and organization; and some have been localized to particular cell surface domains and/or have been found to be expressed in certain life cycle stages.

The ABD domains are, in general, quite similar (Table S2), consonant with their inclusion in the original alveolin/IMC subclass (22). Most have significant (4% to 6%) Y content and a high level of P endowment (9% to 13%). At least one VPV module is present in one or more of the three domains (head, ABD, and tail) in 77% of the sequences, but only 4 of the 42 proteins meet the articulin threshold of having >3 VPV modules per ABD domain (*Babesia* [Text S1.3]; *Cryptosporidium* [Text S1.7]; *Plasmodium* [Text S1.20]; *Toxoplasma* [Text S1.38]).

Some of the tail domains in *Plasmodium* epiplastins are N-rich, with runs of up to 12 contiguous Ns (Text S1.20), reminiscent of the Q-rich tails in *Paramecium* and the G-rich tails in euglenids (see below). A coiled-coil configuration is predicted within one *Cyclospora* head and two *Toxoplasma* tails (Table S2).

### The alveoli and epiplasts of alveolates: photosynthetic apicomplexans.

The free-living chromerids (92, 93), including *Chromera velia* and *Vitrella brassicaformis*, form a sister group to the parasitic apicomplexans, a key distinction being that they retain a photosynthetic chloroplast which is reduced to a nonphotosynthetic organelle, the apicoplast, in the parasites. Since the relationships between these organisms are still being evaluated, we refer to them here as photosynthetic apicomplexans.

**FIG 7:**
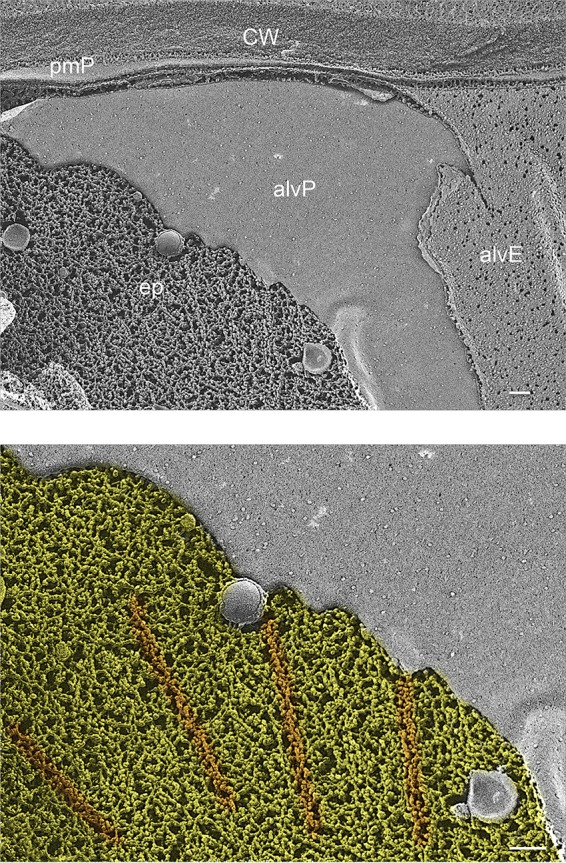
Epiplast of chromerid *Vitrella brassicaformis*. ep, epiplast; alvP, P fracture face of alveolar membrane; alvE, E fracture face of alveolar membrane; pmP, P fracture face of plasma membrane; CW, cell wall. Colored image: yellow, epiplast; orange, microtubules. Scale bars: 100 nm.

Thin-section TEM shows a vaguely filamentous epiplast layer between the alveolar undersurface and the subtending microtubules in *Chromera* (see Fig. 24 and 26 in reference 94). An *en face* QFDEEM image of the epiplast in *Vitrella* (Fig. 7) shows a meshwork of fine filaments interspersed with microtubules. Chromerid cells are encased in robust walls (94, 95) (Fig. 7), suggesting that their epiplasts may not be as important for structural support as in the naked parasites and pointing to epiplastic participation in other facets of cell organization (see Discussion).

### The epiplastins of photosynthetic apicomplexans.

We recovered 13 epiplastin sequences in *Chromera* and 11 in *Vitrella* (Text S2), comparable to the 14 identified in *Toxoplasma* and 16 in *Plasmodium*. Of these, there are four articulins in *Chromera* and seven in *Vitrella* (four orthologous) compared with approximately one articulin per genome in the parasitic apicomplexans. The dinoflagellates, representing a sister lineage of the apicomplexans, have a chromerid-level representation of articulins (see below). One *Chromera* protein is predicted to form a coiled-coil in its ABD domain (Text S2.4; see also Table S2, row 17).

Except for the disparity in the levels of articulin representation, the ABD domains in the photosynthetic and parasitic apicomplexans have similar amino acid profiles (Table S1), as expected given their common phylogeny.

### The alveoli and epiplasts of alveolates: dinoflagellates.

Dinoflagellates are classified as thecate (cellulosic walls present) or athecate (absent), where the thecate group (∼50% of known species) is monophyletic (96), and thecal size and organization serve as important taxonomic markers. Several studies (97–99) have documented the presence of narrow “thecal plates” in thin-sectioned alveolar cisternae of several dinoflagellates, whereas such entities are not evident in freeze fracture replicas (reference 99; see also Text S9.16). During the complex cell division process called ecdysis, the cell produces an external bilayered “pellicle” (99, 100); its outer PI layer, posited to contain sporopolllenin (101), resembles the algaenan/sporopollenin layer of *Nannochloropsis* (102), and its inner PII layer consists of cellulose microfibrils (Text S9.17). The relationship among the thecal plates, the pellicle, and the armored morphology of thecate dinoflagellates is complex (103) and merits further investigation, as does the ultrastructural basis for the prominent ridges seen in SEM images of athecate dinoflagellates (104, 105).

**FIG 8:**
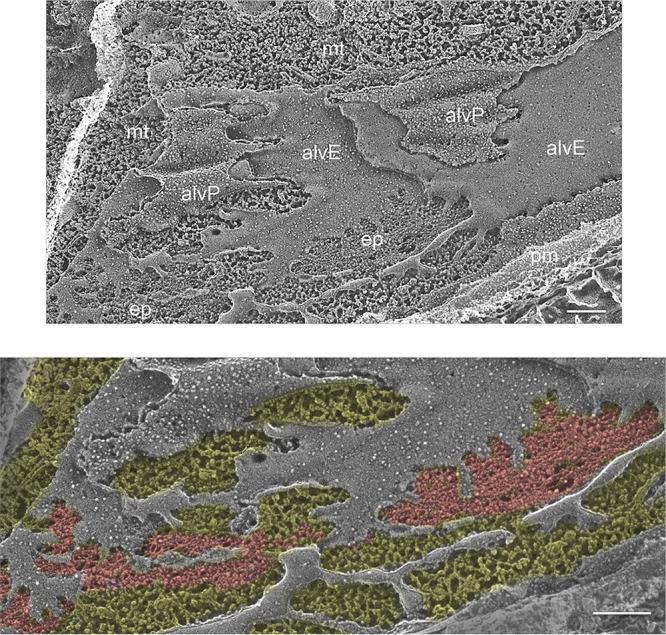
Epiplast of dinoflagellate *Glenodinium foliaceum*. ep, epiplast; alvP, P fracture face of alveolar membrane; alvE, E fracture face of alveolar membrane; pm, plasma membrane; mt, microtubules. Colored image: red, epiplast; yellow, cytoplasm. Scale bars: 200 nm.

A thin epiplast between the alveolar cisternae and the subtending microtubules has been visualized in thin sections (see Fig. 21 in reference 104) and in cross-fracture (Text S9.16); it is considerably thicker in the enormous *Noctiluca* species (30). An *en face* QFDEEM view of the epiplast in Glenodinium foliaceum (Fig. 8) displays enmeshed filaments, similarly to those visualized for the chromerid *Vitrella* (Fig. 7), lying slightly above the microtubules.

### The epiplastins of dinoflagellates.

The transcriptome of Kryptoperidinium foliaceum includes 17 epiplastins, two of which are articulins; that of *Symbiodinium* sp. contains 21 epiplastins, four of which are articulins (Text S5). We also analyzed three articulin orthologues from Karlodinium veneficum and two nonarticulin sequences from the athecate Oxyrrhis marina (22) (Text S5). Two 130 K bands from an Amphidinium carterae lysate are recognized in Western blots by antisera raised against ciliate epiplastins (21). The Y content of their ABD domains is low.

### The alveoli and epiplasts of alveolates: ciliates.

The ciliates have specialized in elaborating a highly complex cortex, with numerous basal bodies/cilia and interconnecting fibers (17, 106), but all retain an epiplast layer, which is continuous in Pseudomicrothorax dubius and Tetrahymena thermophila and subtends individual alveoli in Paramecium aurelia (9, 46, 107, 108). The alveoli are connected by sutures in the fashion of the glaucophytes and apicomplexans (see Fig. 7 in reference 19 and Fig. 5 in reference 109), and the epiplast itself is fibrillary (5, 18, 19, 106, 107, 110, 111), as in apicomplexans and dinoflagellates. It resists detergent or glycerol extraction, forming the outer matrix of cell ghosts in *Tetrahymena* (4, 5, 43, 44) and *Pseudomicrothorax* (18, 107, 112). It also appears to participate in sculpting the shape of the oral apparatus in *Tetrahymena* (113). Text S9.19 shows thin-section images of the epiplast in *Tetrahymena* ghosts.

### The epiplastins of ciliates.

The four ciliates evaluated were found to carry distinctive epiplastins and are considered separately below.

### (i) Pseudomicrothorax dubius.

The three epiplastins characterized, first identified by Huttenlauch et al. (21, 114), are classic articulins, with multiple VPV units present in the ABD domain and extending into the tail (Text S3.24 to 3.26).

### (ii) Pseudocohnilembus persalinus.

A parasite of marine fish (115), P. persalinus contains three classic articulins (Text S3.18 to 3.23), two of which also have numerous VYP modules found in cryptophytes and *Caulobacter* as well (see below).

### (iii) Paramecium tetraurelia.

A group of small epiplastins comprise the epiplasmin subclass (29), designated EPI, described as unique to *Paramecium* (46) (but see below). Extracts enriched in dissociated epiplasmins form abundant 5-nm-diameter filaments upon dialysis (26) (see Discussion). The 51 annotated epiplasmin genes fall into five subclasses, each with many variants. We analyzed a subset of those that have been localized to the epiplast (see Table 1 of reference 46) (Text S3.6 to 3.17). Their short ABD domains have more Y (mean of 14%) than any other epiplastin, and they are low in P residues (Table S2); all but one carry a single VPV unit. A distinguishing feature is a strong endowment of Q in both the head and tail, often repeating in blocks of three or more residues (see, e.g., Text S3.14). All the epiplasmin ABD domains yield a positive score for the DUF2816 motif (where “DUF” represents “domain of unknown function”; http://pfam.xfam.org/family/PF10992) (Table S2), likely because of their high Y content.

The *Paramecium* genome also encodes three additional epiplastins, all articulins (Text S3.2 to 3.5). Two orthologues (Text S3.2 to 3.4) share two distinctive features: (i) P residues, whose presence precludes the adoption of coiled-coils, are absent from the heads and the first halves of the ABD domains, permitting the adoption of several predicted coiled-coils (Table S2); (ii) the second halves of the ABD domains contain multiple VPV modules that characterize the articulins. Coiled-coils are also predicted in their tail domains and in the head of the third protein (Table S2, rows 150 to 152).

### Tetrahymena thermophila.

Two epiplastins (Text S3.35 and 3.36) appear to be in the same subclass as the *Paramecium* epiplasmins, including showing a positive score for the DUF2816 motif (Table S2). It is not known whether the group’s representation has been reduced or whether more such genes will be identified.

*Tetrahymena* epiplastins ALV1 and ALV2 (Text S3.27 to 3.30) have been previously identified (24), and two other sequences resulting from our search, representing either the same genes or orthologues, are included for reference (Text S3.31 to 3.34). Reminiscent of the two proteins in *Paramecium*, coiled-coil domains are predicted in P-absent regions of their heads and the first part of their ABD domains, but the rest of their long ABD domains is canonical. None of the *Tetrahymena* epiplastins is an articulin, representing the one organism in our study where such a protein has not yet been identified.

Twelve additional proteins (the 12 are listed in Text S8.2 and a subset parsed in Text S8.10 to 8.13) have been identified in cortical preparations of *Tetrahymena* (24); some have been further localized to the cell surface via tagged constructs, and some display versions of ABD domains. However, none has the amino acid profile of an epiplastin; all are predicted to be fully α-helical; and several have since been annotated in the NCBI database as associated with kinetodesmal fibers.

### The epiplasts of cryptophytes.

Cryptophytes, which do not have alveoli (Fig. 1A), either are colorless (116, 117) or possess a plastid acquired via the secondary endosymbiosis of a red alga (15). Numerous publications (7, 118–127; for reviews, see references 15 and 128) have documented that the cryptophytes assemble a striking variety of epiplasts, called inner periplast components, beneath their plasma membranes.

Some Crytophyte epiplasts take the form of a single sheet, but most are organized as plates, with ejectosomes (secretory organelles) localized to the plate boundaries. The plates adopt various sizes and shapes (rectangular, hexagonal, or polygonal; summarized in Table 1 of reference 15) and are interconnected by sutures. In *Cryptomonas* and Proteomonas sulcata, the epiplast can transition from plate to sheet (7, 125). The plates can display fine striations (119) (Text S9.29) that are reminiscent of the plates in *Cyanophora* (Fig. 5) or can appear homogenous or crystalline (118, 123) or fibrillary (117). In SEM images, *Goniomonas*, an early branching colorless cryptophyte, displays ridges extending anterior to posterior to delineate 7 plates, with ejectosomes located along the boundaries (117), and TEM thin sections of an unidentified colorless cryptophyte show a fuzzy epiplast layer (116).

*Cryptomonas* epiplasts have been shown to be stable in the presence of nonionic detergent exposure, generating cell ghosts (7) (Text S9.30). Since the cryptophyte epiplast is not subtended by a microtubule cytoskeleton (124), the ghosts are flattened on themselves rather than three-dimensional like the ghosts of alveolates (K. Hoef-Emden, personal communication).

**FIG 9:**
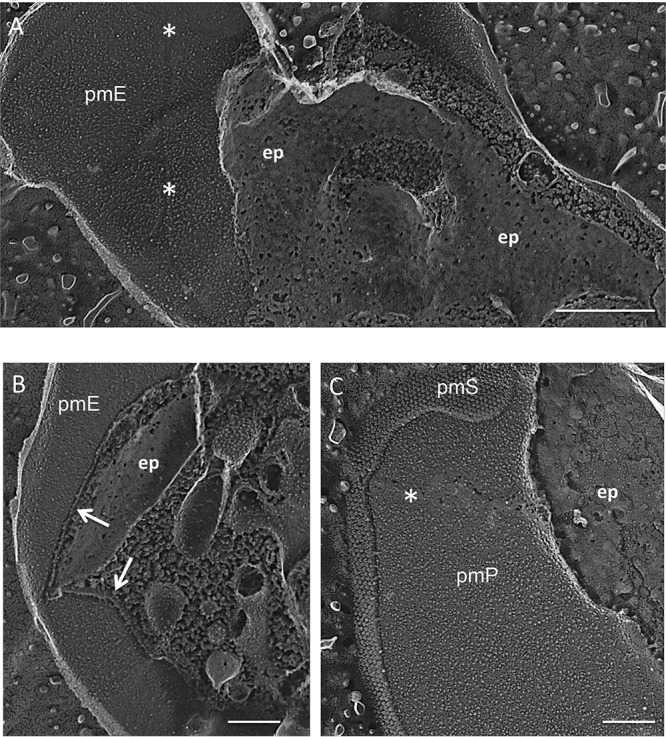
Epiplast of cryptophyte *Guillardia theta*. ep, epiplast *en face*; arrow, epiplast cross-fracture; pmP, P fracture face of plasma membrane; pmE, E fracture face of plasma membrane; pmS, etched surface of plasma membrane; asterisks, IMP-free bands in plasma membrane fracture faces. Scale bars: A, 500 nm; B and C, 200 nm.

We used QFDEEM to analyze two cryptophytes in well-separated lineages (15). The first, Guillardia theta, was previously reported to produce an inner periplast component consisting of a single thin sheet (180), as does Cryptomonas cryophila (now Geminigera cryophila) (121). We confirmed this observation; the *Guillardia* plasma membrane, which displays a knobbly surface (Fig. 9C; see also Text S9.21), is underlain by an apparently continuous layer, very thin in cross-fracture (Fig. 9B, arrows), with a gummy *en face* texture studded with small perforations (Fig. 9) that were possibly generated during deep etching. The P-fracture face of the plasma membrane displays occasional IMP-free bands (Fig. 9A and C, asterisks) that may represent sites of epiplast association (see below).

**FIG 10:**
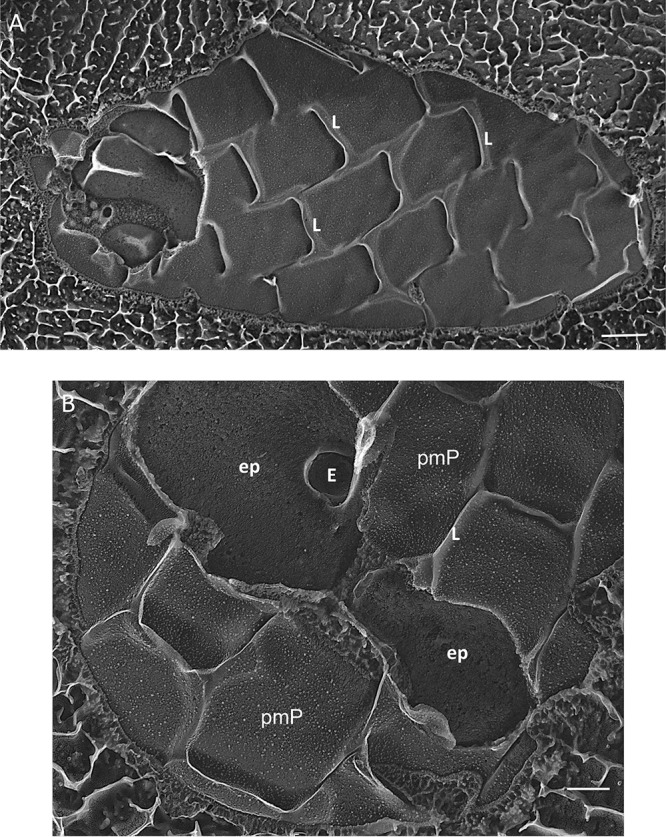
Survey of cryptophyte *Chroomonas mesostigmatica*. (A) Overview of cell with plasma membrane lips (L). (B) ep, epiplast; pmP, P fracture face of plasma membrane; E, ejectosome. Scale bars: A, 500 nm; B, 200 nm.

The second cryptophyte, Chroomonas mesostigmatica (Text S9.23), displays a striking plasma membrane topology noted in previous publications (118, 120, 122, 123): curved protuberances, which we call lips, align in rows along the anterior-posterior axis (Fig. 10A). The lips are reminiscent of euglenid pellicle projections (see below) but are more widely spaced along the anterior-posterior axis and are staggered rather than aligned along the left-right axis. The spacing distance decreases in the gullet region (Text S9.27), indicating organism-wide control over this surface patterning. Ejectosomes localize to the lip boundaries (Fig. 10B; see also Text S9.24 and Fig. 8 in reference 120).

**FIG 11:**
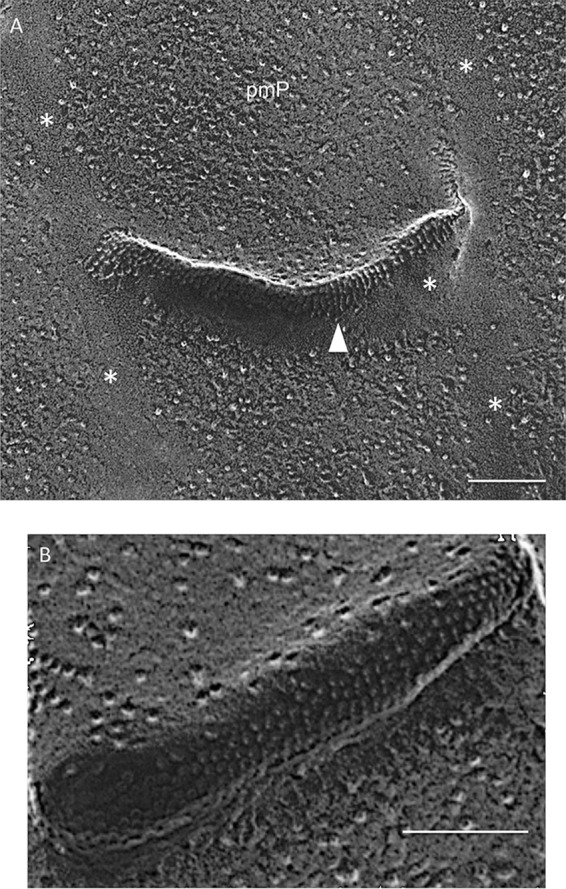
Lips of cryptophyte *Chroomonas mesostigmatica*. Arrowhead, ordered IMPs in lip P fracture faces; asterisks, IMP-free bands in plasma membrane fracture face. Scale bars, 100 nm.

P-faces of the plasma membrane display IMP-free bands that define the longitudinal and left-right borders of the rows (Fig. 11A and Text S9.25 and 9.26, asterisks); these are to be contrasted with the infrequent and more randomly placed IMP-free bands in the plasma membrane of *Guillardia* (Fig. 9A and C, asterisks), whose epiplast lacks a plate organization and apparently makes less-frequent membrane contact. The P-face of the concave portion of each lip carries aligned IMPs (Fig. 11), spaced 10 nm apart, that often appear as striations due to platinum confluence.

**FIG 12:**
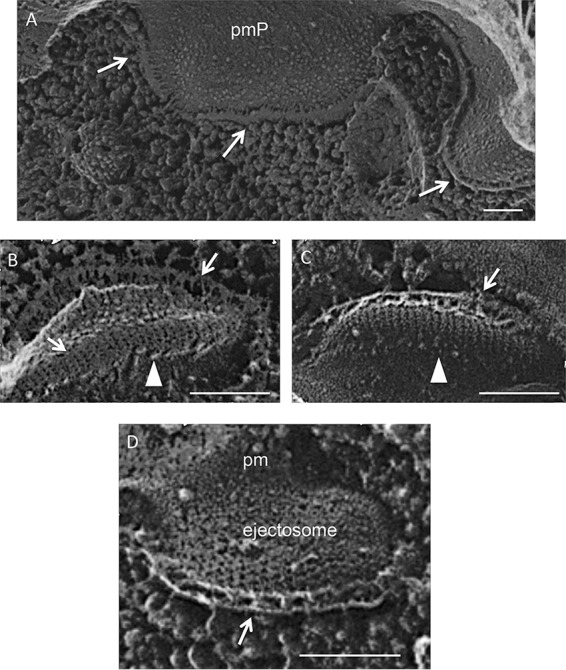
Epiplast cross-fractures (arrows) of cryptophyte *Chroomonas mesostigmatica.* Arrowheads, ordered IMPs in lip P fracture faces. Scale bars, 100 nm.

In TEM thin sections, the *Chroomonas* epiplast appears as a thin (25-nm) layer that is removed with trypsin digestion (118). In QFDEEM cross-fracture, the epiplast makes direct contact with the plasma membrane (Fig. 12, arrows), where contact is mediated by thin filaments (Fig. 12B–D). Where it extends underneath the lip modules (Fig. 12B and C), filaments appear to define the placement of the aligned IMPs (arrowheads). Panels B and C in Fig. 12 document its ladder-like topology; the ladder in Fig. 12D, which surrounds an ejectosome, is ∼15 nm wide and the rungs are spaced ∼25 nm apart.

Published images of various *Chroomonas* species show homogeneous, striated, and crystalline plates (118, 123), consonant with the variability of this trait. By QFDEEM, *en face* views of the plates of Chroomonas mesostigmatica (Fig. 10B) display a gummy substructure reminiscent of the *Guillardia* epiplast (Fig. 9) but lacking perforations.

Previous studies (118, 122, 123) showed that the plates are conjoined by anterior-posterior and left-right sutures that are coincident with the IMP-free domains of the cell membrane marked with asterisks in Fig. 11A and Text S9.24 and 9.25. In a striking negative-stained image of a sonicated preparation of *Chroomonas* sp. (see Fig. 6 in reference 118; image reproduced as Text S9.31), the plates are seen to taper and converge toward a “central element” at the basal end of the cell (see also Fig. 13 of reference 123) and to separate along both the longitudinal and left-right suture interfaces during specimen preparation.

### The epiplastins of cryptophytes.

Articulins are the dominant epiplastin subclass in the cryptophytes: 15/16 in Chroomonas mesostigmatica; 11/18 in Goniomonas pacifica; 4/9 in Guillardia theta; and 8/10 in Rhodomonas salina (Text S4). Two *Guillardia* articulins (Text S4.43 and S4.44) carry predicted transmembrane domains in their tail sequences, the sole examples of such motifs in our survey. VYV modules are found in the ABD domains of an articulin (Text S4.44) and a nonarticulin (Text S4.51) in *Guillardia*, in an articulin (Text S4.9) in *Chroomonas*, and in eight *Goniomonas* epiplastins (all but one articulins) (Text S4.20 to S4.30); they are also present in two articulins from the marine ciliate Pseudocohnilembus persalinus (Text S3.20 to S3.23) and in the epiplastin-related proteins in *Caulobacter* (see below). Many of the cryptophyte ABD domains contain numerous short strings, similarly to the euglenids (see below).

A prominent feature of cryptophyte epiplastins is the inclusion of a PDZ motif that is present in most of the tails in all lineages and also in most of the heads of *Goniomonas* proteins but absent from all other epiplastins in our survey (Table S2). PDZ domains mediate interactions between membrane proteins and cytoskeletal elements and fold into globular domains with internal β-sheets (129). They have been identified in bacteria, plants, and opisthokonts http://www.ebi.ac.uk/interpro/entry/IPR001478?q=PDZ%20domain but not, to our knowledge, in unicellular microbes other than yeasts. Several of the heads and tails of the cryptophyte proteins carry predicted coiled-coil domains (Table S2), and one *Goniomonas* protein (Table S2, row 99) has a predicted stand-alone coiled-coil in its ABD domain; in several other *Goniomonas* and one *Rhodomonas* protein, a predicted coiled-coil in a head or tail domain extends into the ABD domain (Table S2).

### The epiplasts of euglenids.

The euglenids represent a large radiation of both colorless bacteriovores and osmotrophs and lineages carrying a green chloroplast acquired by secondary endosymbiosis (130–132). The plasma membrane is organized in ridge/groove units, reminiscent of the lips of Chroomonas mesostigmatica, but the units are closely spaced and are aligned in anterior-posterior rows called strips. Each strip is subtended by a patterned microtubular cytoskeleton (12, 133–137), and in most species the strips adopt a helical twist over the length of the organism (11). The ridge/groove units are also aligned along the left-right axis, with the ridge of one unit facing the groove of the unit on the adjacent strip. Mucocysts/muciferous bodies dock and secrete their contents in association with the groove regions (134, 135).

In Euglena gracilis, the epiplast lies directly beneath the plasma membrane (Fig. 1A), following the contours of the ridges and grooves in the same fashion that the *Chroomonas* epiplast follows the contours of the lips. It varies considerably in thickness (18 nm in E. gracilis and 175 nm in E. ehrenbergii [138]). It appears finely filamentous *in situ* (10) and in extracted preparations (13, 138) and differentiates into several striated subdomains in the groove regions (136). It retains its membrane association after exposure to chaotropic agents (138), and the ridge/groove topology is unperturbed by nonionic detergent exposure that removes the plasma membrane (13, 139) and by sonication that removes the microtubules (11). When the epiplast is extracted with NaOH, the overlying membrane vesiculates (13, 140). Hence, the epiplast is directly and perhaps solely involved in sculpting the intricate surface topology of the euglenids.

### The epiplastins of euglenids.

Bouck and collaborators (13, 14) identified and characterized two articulins—called 80K and 86K (Text S6.2 to S6.5)—that constitute 60% of the isolated *Euglena* epiplast and noted their abundant VPV endowment. Using the Pfam search, we found five additional articulins in Euglena gracilis, while 44 epiplastins were found in Eutreptiella gymnastica (141) using the LCR search; the latter can be aligned as two related but highly divergent groups (Text S6.42 to S6.44). All but two (Text S6.25 and S6.40) of the members of the *Eutreptiella* protein subset that we analyzed are scored as articulins. VPV units are also found in many of the head domains in both species; they are also found in three *Euglena* tail domains but in none of the *Eutreptiella* tail domains.

Several of the articulin ABD domains in both species are remarkable for their number of short strings: one *Euglena* protein (Text S6.6) has 144 short strings bearing 61 VPV modules, while a *Eutreptiella* protein (Text S6.28) has 112 strings and 34 VPV modules. A similar ABD architecture is also found the cryptophytes: a *Rhodomonas* protein (Text S4.62), for example, has 77 short strings and 29 VPV modules. Most of the euglenid domains have very low levels of Y, whereas the cryptophytes have average levels (Table S2, column AB).

### Epiplastin-like proteins in organisms apparently lacking epiplasts.

The diagnostic features of epiplastins—medial ABD domains with high percentages of VI, low levels of AG content, and predicted β-strand/random-coil secondary structure—were used to identify several protein classes in additional radiations that display the same profile. Since these organisms have not been shown to assemble epiplasts (one of our three criteria for epiplastin designation), and since the localizations and functions of these proteins are unknown, we designate them “epiplastin-like.”

#### *Naegleria* and *Percolomonas*.

The Discoba superphylum includes two major radiations, the Jacobida and the Discicristata. The euglenozoans (which include the euglenids) and the heterolobosans are the two major subdivisions of the Discicristata. Given the rich epiplastin endowment of the euglenids, we queried the genomes of two heterolobosans, Naegleria gruberii and Percolomonas cosmopolitus. Of the eight *Naegleria* candidates with medial ABD domains recovered in an LCR search, only two lacked major predicted α-helical content, and both were predicted to be fully random coil (Text S10.2 to S10.4); of the five recovered in *Percolomonas*, one is also predicted to be random coil in its ABD domain (Text S10.5). All three meet the epiplastin criteria of being rich in hydrophobic and charged amino acids and poor in A and G residues. It is not known whether either organism constructs an epiplast during the course of its life cycle.

#### *Caulobacter*.

Although prokaryotes almost invariably produce cell walls and are therefore not expected to assemble epiplasts, Al-Khattaf et al. (36) noted articulin-like proteins in the genomes of the alphaproteobacterium *Caulobacter,* and we pursued this lead. The NCBI database includes 28 proteins, annotated as articulins, from several species, eight of which (some orthologous) are listed in Table S2 and parsed in Text S10.6 to S10.14. All exhibit classic ABD strings with numerous VPV modules, and those queried are predicted to adopt a β-strand/random coil secondary structure. They all also carry numerous VYV modules, a motif otherwise encountered only in the cryptophytes and a marine ciliate (see above). Interestingly, *Caulobacter* cells have been shown to form consortia with cryptophytes (143), adhering to their surfaces via their inducible holdfasts (144); hence, they may have acquired (the prototype of) their epiplastins via eukaryote → prokaryote horizontal gene transfer (HGT). Conceivably, since mitochondria derive from an alphaproteobacterium (145), there could have occurred endosymbiotic epiplastin-like gene transfer to the original protoeukaryotic host. Although *Caulobacter* articulins have not yet been localized, one possibility for a location is a submembranous plaque visualized beneath the tip of the holdfast (see Fig. 1 in reference 146).

Notably, the protein crescentin, considered an intermediate filament analogue in *Caulobacter* (142), can be parsed into long ABD strings. However, it is predicted to be completely α-helical (Text S10.15) and is therefore not considered to be epiplastin-like.

Epiplastin-like genes, sometimes annotated as encoding a tubulin binding protein for reasons that we have not been able to ascertain, are found by BLAST analysis (see Materials and Methods) in three classes of Basidiomycota, most in the order Tremellales, but in no other sequenced fungal genomes (Text S10.16 and S10.17). Two examples of this group were previously noted for *Cryptococcus* and *Coprinopsis* fungi (22). Each genome apparently possesses a single copy of such a sequence. Although divergent, all exhibit classic ABD domains with short strings and numerous VPV modules, and those queried are predicted to adopt a β-strand/random coil secondary structure (Text S10.18 to S10.23). In unpublished QFDEEM studies of *Cryptococcus* performed in our laboratories, there was no evidence of an epiplast-like layer beneath the plasma membrane.

#### Insecta.

Epiplastin-like sequences, variously annotated as titin, titin-like, or proline-rich protein 4, were found in the genomes of numerous Insecta radiations using the same BLAST analysis (Text S10.16 and S10.17). Their sequences are unrelated to the muscle titin modules which form IgG-like beta-sandwiches (147) (Text S8.19), and they are not found in other arthropods or invertebrates. While divergent, all exhibit classic ABD strings with numerous VPV modules, and those queried are predicted to adopt a β-strand/random coil secondary structure (Text S10.24 to S10.26). Each genome apparently possesses a single copy of such a sequence.

The presence of epiplastin-like proteins in the genomes of Basidiomycetes and Insecta is intriguing. Since these proteins are not found in earlier-branching fungi or in other animals or their direct unicellular forebears, direct evolutionary links to the original epiplastins seem unlikely. The alternatives are two instances of early HGT in these two radiations or convergent evolution. Since (cyto)skeletal proteins are usually encoded by several orthologues/homologues in a given genome, these single-copy genes may well specify novel functions that future mutation and/or protein-localization studies will help to identify.

## DISCUSSION

### Overview.

Several investigators have previously pointed out similarities between what we are calling epiplasts/epiplastins present in the euglenids and in the three alveolate radiations (21–23, 45, 46, 49, 67, 90, 106, 133, 139, 148), and Santore (124) notes similarities between the epiplasts of euglenids and cryptophytes. In this study, we subjected these similarities to comprehensive scrutiny and also analyzed two additional lineages—the glaucophytes and cryptophytes—that had not been previously recognized as epiplastin producers.

Tagged epiplastins have been repeatedly localized to epiplast domains in euglenids and alveolates (references are cited in the introduction), whereas such experiments have not yet been performed with glaucophytes and cryptophytes since the necessary genetic tools are lacking for those organisms. Therefore, the proposal that all four systems are related, while based on strong correspondences in ultrastructure and protein organization, awaits confirmation via localization studies in glaucophytes and cryptophytes.

In listing the number of epiplastin genes per species, we recognize that some may have been missed or misclassified, with transcriptomes particularly likely to yield incomplete data. Moreover, while we single out some examples of likely orthologues or splice variants, we have not rigorously evaluated these parameters. That said, and with the exception of the glaucophytes and *Tetrahymena* (low copy number) and *Eutreptiella* (high copy number), most genomes/transcriptomes that were deeply probed encode ∼10 to 20 epiplastins (see Table S1 in the supplemental material), almost all of which differ from one another in head, ABD, and tail domain sequences, both within species and between species, while sharing common amino acid usage, domain architecture, predicted secondary structure, and documented or presumed cellular localization.

### What are alveoli for?

In four of the six radiations, epiplasts associate with alveolar membranes (Fig. 1B). Plattner (149, 150) has made a strong case that alveoli engage in Ca sequestration and release to aid in the coordination of ciliary beating in ciliates, but he also notes (151) that there is little evidence that the alveoli of apicomplexans are involved with Ca flux. Cellulosic plates have been visualized in the alveolar cisternae of some but not all dinoflagellate groups (98). Disruption of alveolar integrity disrupts the gliding motility of parasitic apicomplexans (see below), a form of motility unique to the parasites. Exocytic/endocytic pores have been visualized in the alveolar membranes of *Plasmodium* (79), but their cargo is unknown. Hence, the ubiquitous presence of alveoli in several key lineages remains largely unexplained.

### What are epiplasts for?

A great deal more can be said about the possible functions of epiplasts. We consider below four functional categories that often overlap: roles in secretion, in flexibility, in gliding motility, and in cellular organization.

#### (i) Role in secretion.

Radiations endowed with epiplasts are also endowed with large organelles that store soluble or structural material, “dock” at junctions between the epiplast and the plasma membrane, and rapidly release their contents in response to agitation and/or the proximity of prey. These organelles are designated ejectosomes in cryptophytes (117) (see Text S11.1A in the supplemental material); trichocysts in ciliates and dinoflagellates (150, 152) (Text S11.1B); and mucocysts in glaucophytes, ciliates, and euglenids (Text S11.1C). The apical secretory organelles of apicomplexan parasites, called micronemes, rhoptries, and dense granules, that play crucial roles in the penetration of host cells are also proposed to belong to this class of organelles (153). Importantly, in most cases where the process has been carefully studied, the secretory contents do not directly cross the plasma membrane but rather pass through a junction created by the epiplast with the alveolar sutures or the plasma membrane (Fig. 4A, 5A, and 10B; see also Text S9.2, S9.3, and S11.1C). Hausmann (116), who provides excellent TEM images of this process in a cryptophyte, suggests that the epiplast may function, in part, to protect the plasma membrane from disruption during the explosive discharge of these organelles. Notably, however, cryptophytes also possess large ejectosomes in their gullet domains (15) where the plasma membrane is subtended by striated bands and not an epiplast (124); hence, this suggestion may be germane only to the cell body organelles.

10.1128/mBio.02020-18.11 TEXT S11 Evolutionary scenario. Download Text S11, DOCX file, 3.1 MB. Copyright © 2018 Goodenough et al. 2018 Goodenough et al. This content is distributed under the terms of the Creative Commons Attribution 4.0 International license.

#### (ii) Role in flexibility.

The importance of a membrane skeleton in endowing cellular flexibility has been most intensively studied in erythrocytes, which undergo dramatic shape changes as they squeeze through capillaries that are narrower than the cells in their normal disc shape. Biophysical analyses (154, 155) have shown that under these circumstances the proteins in the spectrin-based membrane skeleton lose their interconnectivity, allowing the cells to stretch into a bullet shape.

Such viscoelastic properties have also been documented in epiplast-based unicellular organisms. *Euglena* undergoes dramatic shape changes, called metaboly (13, 156), when it encounters an obstacle, as illustrated in the movie available at https://www.youtube.com/watch?v=IWyol3u-OL8. *Paramecium* engages in similar twisting (https://www.clipzui.com/video/83o426p3p4q5z4u2h5k413.html), and while *Toxoplasma* glides, it also engages in a sinuous twirling motion during host invasion (81), as illustrated at https://www.youtube.com/watch?v=Y5YxpOrUpdQ.

The structural basis for euglenid metaboly has been analyzed in the colorless euglenid Astasia longa (9, 157). Its 40 longitudinal pellicular strips are organized such that each ridge on one strip fits into the groove of its adjacent strip, linking the strips together along the cell’s longitudinal axis. The strips are also linked together horizontally by a system of filaments that extends from the epiplast underlying one groove to the epiplast underlying its adjacent groove. When the cell is straight, these filaments are perpendicular to the long axis, but when the cell assumes a round shape, the filaments instead adopt an oblique angle as each strip slides incrementally past its neighbor to generate a global torque. The sliding itself, which is ATP dependent in detergent models of *Euglena* (158), is apparently dependent on a microtubule-based system, in which case the filament/epiplast system would confer flexible constraints on the extent of sliding. While it is not known whether the filaments themselves are constructed of epiplastins, they are clearly anchored in the epiplast.

The epiplasts visualized in freeze-dried preparations of *Toxoplasma* (Fig. 6) illustrate an analogous arrangement at a much smaller scale. Longitudinally aligned filaments, spaced 30 nm apart, are regularly cross-bridged by filaments that are ordinarily perpendicular to the longitudinal axis but in some fields are instead oriented obliquely, suggesting that they are capable of exerting flexible constraint. This filament system appears to align IMPs in the overlying alveolar membrane, much as the filaments in *Chroomonas* appear to align IMPs in the overlying plasma membrane in lip domains, representing interactions that presumably participate in generating the topography of the cell surface.

These examples of global cellular flexibility are doubtless accompanied by more-localized instances where epiplasts absorb impacts, such as those from microparticulates, that are buffeted by cell walls in walled organisms. Indeed, the imposition of an alveolar system between the plasma membrane and the epiplast may serve as an additional “cushion” to maintain cellular integrity. Epiplast-based lineages are propelled by flagellar/ciliary motility (restricted to gametes in the parasitic apicomplexans), and while the mechanical stresses incurred by that activity are presumably largely absorbed by its attendant microtubule/fiber systems, an additional epiplast-based flexibility would likely be adaptive.

#### (iii) Role in gliding motility.

Parasitic apicomplexans engage in actin/myosin-based gliding motility (reviewed in references 76, 159, and 181), and evidence is accumulating that the parallel epiplastic filaments visualized in Fig. 6, and their associated microtubules, provide a system of “tracks” that anchor this system. The 30-nm spacing of the filaments corresponds to the 30-nm spacing of IMPs in the overlying alveolar membranes, and the integral GAP and GAPM proteins of these membranes coimmunoprecipitate (co-IP) with both the MyoA motors and several alveolin proteins (84). In *Plasmodium*, deletions of IMC1b and IMC1h negatively affect gliding motility and reduce infectivity (47, 54, 56). In published models of this system, the alveolin component is illustrated either as coiled-coils (see Fig. 6 in reference 84) or as a disordered meshwork (see box 1 in reference 76); our analyses suggest that these models merit refinement.

#### (iv) Role in cellular organization.

The lineages considered in this study assemble microtubule-based cytoskeletons which strongly contribute to the maintenance of cell shape and integrity. In cryptophytes, microtubules are restricted to the basal-body/rhizostyle cytoskeletal complex (124, 156), whereas in other groups, “cortical” microtubules also subtend the epiplast. The cryptophytes adopt a tapered cell shape without an epiplast-associated microtubule endowment, and in many parasitic apicomplexans the microtubule cytoskeleton extends only to the cell’s midline and not to its tapered tail (Fig. 6). Moreover, microtubule depolymerization does not disrupt the integrity of the epiplast in apicomplexans (81) or *Euglena* (140). These observations, coupled with the examples offered below, support the hypothesis that in epiplast-assembling organisms, cellular organization is highly influenced by the epiplast layer.

One example of “global” epiplast organization is given by the gullet region of cryptophytes and the reservoir region of euglenids, where phagocytosis of prey takes place. The epiplast surrounding most of the cell is absent in these regions (124, 134), allowing the phagosome membranes to invaginate. Such an arrangement would not, of course, be possible for cells surrounded by a wall.

A second global example is the patterning of the cell surface into parallel structural units. As Lefort-Tran et al. (10) note: “The cortical complex of *Euglena* displays a highly repetitive structural pattern which is closely comparable to the cortex of ciliates such as *Paramecium* and *Tetrahymena*.” While cortical microtubules are prominent in both of these cases, studies reviewed in Results indicate a prominent role for the epiplast in setting up these patterns. The parallel arrays of plates in the cryptophytes, and the patterned spacing of *Chroomonas* lips, moreover, are by definition organized by their epiplasts since they lack an underlying microtubule-based cytoskeleton.

A more local example relates to the placement of extrusomes. In cryptophytes, the ejectosomes “dock” at the corners of the sutures that interconnect the epiplast plates (Fig. 10B; see also Text S9.4). Glaucophytes display a similar arrangement except that docking also involves alignment with the sutures that interconnect the alveolar cisternae (Fig. S11.1C). Two ciliate studies demonstrated that these docking sites preexist and are not created by the docking event. (i) *Pseudomicrothorax* can be grown under conditions where trichocysts are assembled but fail to dock; nonetheless, trichocyst docking sites form in the epiplast at each cell division (18). (ii) *Paramecium* mutants that fail to form trichocysts nonetheless continue to form epiplast thickenings and overlying arrays of IMP particles at the correct docking locales (161, 162).

A fourth example, detailed in Results, is given by the euglenids and by the cryptophyte Chroomonas mesostigmatica, where not only their global cell surface patterning is under apparent epiplast control but also the shape of their plasma membrane ridge/groove and lip units, where the epiplastins appear to dictate the patterning of their constituent IMPs. The parasitic apicomplexans similarly appear to entrain the placement of alveolar membrane IMPs using epiplastin filaments.

The most extensively documented examples come from two sources: (i) the parasitic apicomplexans, where it has been possible to localize tagged epiplastins to particular regions of the cell, follow the timing of their expression, and evaluate their abundance in the various cell types that differentiate during the life cycle, and (ii) the ciliates, where such localization studies are facilitated by large cell size and a patterned cortex. These studies, many summarized by Francia and Striepen (52) and cited in the introduction, leave little doubt that the epiplast is a highly differentiated structure that plays a key role in cellular organization. Indeed, Kudryashev et al. (31) suggest that all of the organelles in *Plasmodium*—including mitochondria, apicoplast, and microtubules—are tethered to the epiplast.

This leaves us with a meta-question. In lineages with a microtubule cytoskeleton, does it organize the epiplastic membrane skeleton or does the membrane skeleton organize the cytoskeleton or are they independent of one another? To our knowledge, this question remains unanswered.

### How do epiplastins form epiplasts?

The epiplastin class is unlike other protein classes with which we are familiar. Its salient shared feature—a medial ABD domain with restricted amino acid composition and a predicted β-strand/random coil secondary structure—displays no conserved motifs except VPV in the articulin subclass. Its head and tail domains are dissimilar to one another and contain very few conserved motifs. The proteins vary greatly in size and in ABD length (Text S1 to S7 and Tables S1 and S2). While the construction of epiplasts from these proteins remains far from being understood, the studies considered below single out some of the parameters involved.

#### (i) Targeting studies.

Two localization studies performed with alveolates have addressed whether an ABD domain is involved in targeting its protein to its cellular location. El-Haddad et al. (24) found the endogenous ALV2 protein of *Tetrahymena* to localize diffusely over the entire cell surface, but cloned subdomains generated various patterns associated with basal bodies. They also constructed a synthetic charged repeat motif with an amino acid composition resembling an ABD domain and found that it localized to a broad range of cytoskeletal structures, perhaps because the synthetic sequence is in fact predicted to be wholly α-helical (our analysis). In similar studies with *Toxoplasma,* Anderson-White et al. (32) found that the medial domains of IMC3 and IMC8 target to the cell surface correctly but noted that the probes might have been (hetero)dimerizing with endogenous proteins that have the correct addresses. We have not found studies that examine the targeting capabilities of solo head or tail domains. Clearly, more analysis is needed.

Targeting studies have also been performed with *Euglena* (140). When a sonicated pellicle preparation is treated with 4 M urea and then 10 mM NaOH, the plasma membrane is stripped of its epiplast, with two articulins (Text S6.2 and S6.4) making up 60% of the extract. When the membranes and extract are mixed and dialyzed, an epiplast layer is restored to the membrane inner (but not outer) surfaces, having the same width (17 nm) as the original (20 nm), and 17 nm-long filaments with globular termini are arranged perpendicularly to the membrane, reminiscent of the arrays visualized in *Chroomonas* (Fig. 12). A 17-nm-wide layer is also observed if the ratio of extract to membrane is increased 6-fold, indicating that the binding sites can be saturated. In a second study (160), major integral membrane protein p39, which carries claudin domains (163), was shown to serve as an epiplastin binding site. These observations suggest that the epiplastin terminal domains possess specific membrane-targeting information and that the ABD domain participates in filament formation, where the additional proteins in the extract may well participate in the process.

#### (ii) Filament formation studies.

Studies of the small epiplasmin protein subclass of *Paramecium* (25, 26) provide additional insights. The proteins in an 8 M urea extract that was subjected to SDS/PAGE migrated as three subsets—representing high, medium, and low molecular weight (HMW, MMW, and LMW, respectively)—and combinations were subjected to repolymerization assays following dialysis. The HMW fraction formed abundant 5-nm-wide filaments on its own; the MMW and LMW fractions failed to do so on their own but did so when combined. When the HMW sample was divided into three subfractions enriched in particular epiplasmins, each subfraction failed to form filaments on its own but readily formed filaments when recombined. These results indicate that the epiplasmin filaments are assembled from protein combinations but do not indicate whether these filaments, and epiplast filaments in general, are chains of single globular domains or whether they also associate side to side as protofilaments. The ultrastructure of *Toxoplasma* cell ghosts (Fig. 6) and *Chroomonas* (Fig. 12) indicates that epiplastins can also align end to end to form long filaments and that these can be cross-bridged by shorter filaments.

Epiplastins associate as a thin meshwork in alveolates and euglenids and as thin plates or sheets in cryptophytes and glaucophytes. Images from Chroomonas mesostigmatica analyses (Fig. 12) document that the plate edges adopt an open-meshwork topology where they interact with the plasma membrane or ejectosomes and that some *Cryptomonas* strains alternate between discrete plates and a continuous sheet at different life history stages (7). Nothing is known about how filament length and layer thickness are controlled.

#### (iii) β-strand/β-sheet studies.

The predicted secondary structure of the ABD domains as a mixture of β-strands and random coils awaits confirmation by biophysical techniques, but support for this prediction comes from studies (164) of the amino acid composition of β-strand-containing proteins in the Protein Data Bank. V and I, the dominant amino acids in ABD domains (Tables S1 and S2), were found to be the most highly represented in the β-strands (17% and 13%, respectively); L, ranking third (12%), is for some reason uncommon in ABD domains (Table S2, column R). The authors also note a high score for doublets (VV, VI, IV, and II) and triplets (VVV and VIV), units that are common in ABD domains. Even without biophysical confirmation, the consistent prediction of β-strand structure in epiplastin ABD domains from six radiations using two prediction tools indicates that, at the least, the proteins all adopt a related conformation that does not include α-helices (which are far easier to predict than β-strands).

We are aware of three examples in which β-sheets are involved in filament formation. The members of the β-keratin superfamily (165, 166) (not to be confused with the α-keratins in the intermediate-filament family) in reptiles and birds assemble into filaments via β-β interactions. Each monomer carries several short β-sheet domains, rich in V, I, and P (165) (Text S8.21), and these serve as contact regions for dimerization. The dimers then polymerize to form long filaments, 3 to 4 nm in diameter, that are both viscoelastic and detergent insoluble, all features of the epiplastins.

A second example is the protein titin, which is the viscoelastic component of striated muscle (167) and is also implicated in mitotic-spindle dynamics (168). The enormous titin polypeptide (25,000 amino acids) adopts a beads-on-a-string topology: modules of 100 amino acids, each containing 7 to 8 β-sheets (147) (Text S8.20) and lacking ABD domains, fold into hundreds of IgG-like β-sandwiches that collectively generate a single filament, 43 nm long and 3 to 4 nm wide (169). Given that an average ABD domain contains ∼200 amino acids (Table S1), which might fold into 2 to 3 of such modules, and that epiplastin filaments can be 17 nm long (140), the titin model *per se* is not applicable to epiplastins, but possibly individual epiplastin monomers associate end to end to form filaments in the fashion of titin.

A third example is that of the many filamentous forms adopted by β-sheet-containing fragments of degraded proteins, such as amyloid-β, which participate in the etiology of degenerative diseases (170–172). The monomers are rich in V and I and charged amino acids (170). It would be of considerable interest if epiplastins were to adopt analogous conformations but with nontoxic consequences.

A striking feature of the predicted epiplastin β-strands is their length. Whereas the PSIPRED algorithm predicts, consonant with published biophysical data, that the mean β-strand lengths are ∼4 amino acids in β-keratins and ∼6 amino acids in IgG (Text S8.19) and titin (Text S8.20), it predicts a mean length of 13 amino acids in queried *Toxoplasma* epiplastins, with some strands longer than 20 amino acids. The other epiplastins in our survey also displayed long predicted β-strands (Fig. 3; see also Text S1 to S7). The one other natural protein with such long β-strands that we have encountered is porin (Text S8.22), which lacks ABD/VI-rich domains. It folds into β-barrel monomers that insert into the outer membranes of bacteria and organelles but that are not known to form filaments. Long β-strands also characterize the monomers of pathogenic peptides such as amyloid-β.

#### (iv) Head and tail motifs.

Since the images from *Euglena* suggest that filaments bind to membranes via globular domains (140), we examined head and tail domains for the presence of known membrane-interaction motifs, but we found only a few predicted coiled-coil domains (30 in 219 proteins, spread across lineages [Table S2, C-C] and concentrated in *Goniomonas*) and PDZ domains that are restricted to cryptophyte proteins (Table S2). Head and tail domains commonly (and ABD domains occasionally) include C residues (green highlights in Fig. 2 and Text S1 to S7), and in some cases (e.g., *Symbiodinium* [Text S5.24], *Guillardia* [Text S4.43], and *Toxoplasma* [Text S1.45]) they are abundant. Mutagenesis of the C residues in the IMC1c protein of *Plasmodium* affects sporozoite shape and infectivity (62), and the residues have been shown to undergo palmitoylation (173), possibly contributing to membrane associations.

#### (v) Perspective.

Future research deepening our current understanding of epiplast construction has three possible applications. First, there could emerge novel approaches to interfere with epiplast assembly and hence the infection cycles of apicomplexan parasites. Second, the information might be applicable to the formation of pathogenic amyloid-β-like filaments, constructed from β-sheet-containing fragments (170–172), that contribute to several diseases. Third, the principles governing the assembly of such proteins into nanometer-thick films that are both viscoelastic and detergent insoluble could have applications in bioengineering.

### Evolutionary scenario.

When similar traits are encountered in highly divergent lineages such as those considered here, one alternative is to propose that while some may share evolutionary continuity, others may have arisen by convergent evolution (174)—that is, some lineages may have independently come up with/converged upon the “idea” of protein domains encoding ABD- and VI-replete β-sheets for assembling membrane skeletons. The apparently independent inclusion of such domains in the epiplastin-like proteins of Insecta, Basidiomycete, and *Caulobacter*, whose function(s) is as yet unknown, supports thinking along these lines.

The alternative, particularly for a lineage-restricted trait such as epiplast construction where the constituent proteins are structurally related, is to propose that the trait was present in a very deep ancestor and persisted in a subset of subsequent radiations. The evolutionary scenario offered in Text S11 is framed using this second premise. Tests of this and alternative hypotheses will require an approach, such as intron-retention analysis (175), which is capable of detecting long-preserved relationships.

## MATERIALS AND METHODS

### Strains.

The following strains were grown in the laboratories noted in Acknowledgments and shipped live overnight to St. Louis for quick-freezing: Cyanophora paradoxa CCMP329 (Pringsheim strain); Guillardia theta CCMP2712; Chroomonas mesostigmatica CCMP1168 (note that the genus *Chroomonas* has recently been reevaluated [176]); Neospora caninum Nc-1; Toxoplasma gondii RH; Vitrella brassicaformis gen. et sp. nov.; Chromera velia CCMP2878; Glenodinium foliaceum CCAP 1116/3; *Symbiodinium* sp. CS-156.

### Bioinformatics.

We first used Hmmer to conduct a Pfam search of 58 eukaryotic genomes and 14 transcriptomes for the IMCp motif generated from P. falciparum alveolin homologs (https://pfam.xfam.org/family/imcp) and recovered strong candidates from glaucophytes, euglenids, cryptophytes, and alveolates, whereas none were recovered from the other eukaryotic groups queried (see Table S1 in the supplemental material). The collected sequences were manually inspected for modules enriched in EKDR amino acids (22), generating the Pfam set. Proteins recovered in this search are designated IMCp in Table S2, column G.

To search for additional epiplastins that deviate from the alveolin-type motif, we scanned all available genomes/transcriptomes for low-complexity regions (LCRs) using SEG software (177) with a window size of 30, an initial low-complexity cutoff value of 3.0, and a low-complexity extension cutoff value of 3.1. Collected LCRs were analyzed for amino acid composition, and those enriched for EKDR by >20% or 25% were chosen; the threshold was determined by the inclusion of most high-confidence sequences. Manual inspection of the chosen LCR domains was then performed, and sequences enriched in acid/base dyads and in V and I residues were considered epiplastin candidates. The full amino acid sequences of these proteins were obtained and subjected to the evaluative criteria described in Results, generating the LCR set of epiplastins.

The proteins in the combined Pfam and LCR sets (a total of 219; Table S2) were searched for additional homology domains using INTERPRO scanning. Coiled-coils were predicted using COILS v2.2.

Finally, we conducted a BLAST search using the ABD domain sequence of the *Chroomonas* MMETSP0047_c25199_g1_i1_g48336 protein (see Text S4.8 in the supplemental material) and recovered two additional epiplastin-like classes, one restricted to Basidiomycetes and the other to Insecta (Text S10.16 and S10 to S26).

### Secondary-structure predictions.

The predictions reproduced in this report were generated by PSIPRED (http://bioinf.cs.ucl.ac.uk/psipred/). Many of the predictions were confirmed using YASPIN (http://www.ibi.vu.nl).

### Quick-freeze deep-etch electron microscopy.

(QFDEEM) was performed as described previously (178, 179).

### *Cyanophora* ghosts.

Cells were suspended in cold HMEK (10 mM HEPES, 5 mM MgSO_4_, 2 mM EGTA, 25 mM KCl, pH 7.4), to which was added NP-40 (Particle Data Inc., Elmhurst, IL) to reach the final concentrations given in Text S10.7 and S10.8. After 3 min, half of each sample was fixed by adding drops of a 4% solution of glutaraldehyde (Electron Microscopy Sciences) in HMEK to reach a final concentration of 1%; the other half was first brought to 1 M glycerol–5% sucrose and then fixed as described above. Phase microscopy images of fixed cells were taken using a Zeiss Axioscope microscope and a 40× objective.
