# Supplementary material for: Epiplasts: Membrane Skeletons and Epiplastin Proteins in Euglenids, Glaucophytes, Cryptophytes, Ciliates, Dinoflagellates, and Apicomplexans
Source: mBio. 2018 Oct 30;9(5):e02020-18. doi: 10.1128/mBio.02020-18 (PMC6212826; doi:10.1128/mBio.02020-18)
Supplement: TEXT S1 [file mbo005184120s1.pdf]

1

Key to Gene Annotations

- **General organization:** The medial acid-base dyads (ABDs) are “parsed” into “strings that initiate with a dyad. N-term domain is denoted the head, C-term the tail.
- **Highlights**: Yellow, tyrosine; Green, cysteine; Pink, VPV; Gray, ABD in predicted head and tail domains.
- **Colored font:** Red, glycine residues in ABD domains; Bold-faced red, last G residue in head and first G residue in tail; Blue, alanine residues in ABD domains; Green, repeated string domains.
- **Underscores:** Acid-base triads or tetrads.
- **Commentary**: Notes on distinctive gene features (e.g. orthologues, localization patterns) in green font at top of some pages. Predicted homology domains (e.g. PDZ, coiled-coil) are given in Supplement Table 2.
- **Secondary structure**: PSIPRED predictions for a subset of proteins. Yellow, amino acids predicted in β-strand; pink, amino acids predicted in α-helix; no highlight, amino acids predicted in disordered (random coil) domain.

2

Babesia bovis XP_001609023.1

MSSNNPEQLYEGQGPMDQTVFQGNLQSYTMPNNISMAPPTDFANYMAPPPEL
QWKQRTVTKKNKSRFPCNLCCCCYGEEETVPVKSNLVGDANRSIVLKPIR

RERIVEVMKEEIQERVVNVPQIQYV
DKFVEVPKPIFKYKIKEVKRPVIV
EKIKRVPKIVEE
EKIIEVPEIKYV
EKEVEVPHIVK
KEKVVEVPLPIV
RERRIPVLRLK
KDEKYQEVENINYEDFE

TNIMSNRYGSEAPNNTPIKLQARDIPNNTQEMSKTLKGVYSSNENDNFNRWEEQY
DNGSKVDIDHHRPVMYYNNPVITQVTDNEKVSTSRRTVTKDNTLREQYKVSSPTT
ERKDIGVNNNGHYDQRSNNESIVTEIISVRGISNRSSPKMNQYDVEPLIEDIDEQLV
NRDIIDEDNYVSIYPNIDAMSIKDGSSVETKKQQTRTSQEAQNSESGTDAKSMKKG
DHRSVEEQITHVRITK

3

Babesia bovis XP_001609658.1

Articulin

MSTSPLPHSYISKTPSAMSDSAGLKTPPADAIILEPIVRERIIEVERPEIQERIVDVPEI
QYVQRVTEVPEPVIQENIVRVAKPVLQ

ERIKKVVKPIIQ
EKVVEVPVVEIV
EKVVEVPQYVYQ
EKVIEIPKVVVQ
ERVVNIPKKVT
KERIVEVPKIQY
KEVI
KERIVEVEQEIPEIVH
KDVRVVHYI
DRPVEV
EKIVEVPQIQHKY
KDVITPQYRNVPTPVEVPVKQIRNVPVTKII
ERDVPVPVEIDVVQEF

TCRNIEARYHEIPVPVHVQRIIEHPLPQEAFQNTNLVPMYYCAVQDNLRQMQQHGISY
PNNMPIQNIKNQF

4

Cryptosporidium parvum XP_625571.

MNSCNSLGLCSAESESPILQHNYDSVDTNGNTSFGRTNMSRRDDFCCMSDRN
VEDNENDSFIPQDCCSSNPRIVEDSNQDSVSSPGGNAQIVTVPLVQEVNVR

DRIIEVPEVHITQKIRQKVIV
KDVIRKVPKQEIQYV
DKFVEVPEVRIV
DKFVSKPVTKYV
ERHVPKVEV
REIV
KEIPKIEIQYV
EKIVEVPEIRVV
DKIVEIPTIKHVI
KEVPKIEI
KEIQV
EKIVKVPKIEIKQI
EKERKVLGPVEYIDIPI
EKIILKPNPQII
EKIVQVPIP
KEVEIEVPVYNPDI
RDTVEIEVDNYYTV
EKEVEVPIP

GRIIPVPVEVEVEKIVEVPVQVPQEHIRIVQKQVPQYIEHIRTVEIPQYQDEFVEVPQYV
PMVHHTIIKPVVSQEYKELPPVVEQGDVRYVKEPPQYLETEYIHGEPIELDCNTPLPAP
PSPRYTTIKVNSANNRDS

5

Cryptosporidium parvum XP_626594.1

MAEVPVSEDISASQQPPSSSYSLPPQFSNEPDVRTMASIKATLSKKAEELVK
AGQPYVMTSL**G**PIPLPDNVRNQIP

EKFVAAPVLE
EREVIVAR
REVQ
ERIIEVPQIQYEHKFVEVPQKVVV
EKIIPIP
KDVV
REVEIPRYTATVE
EKVIEVPQGVKFVEVPVEVPIAYPPRIVPVPKPYIV
ERTVELSRPVIE
ERLLEVPQKVYRQVPYY
KDVEVPFVVPRYV
EKLVEIPFHP

GMMYDENMLNQSIPPIPPQSQPPFMMGGPGSAPTSAPLLSMPPPMYGTNDP
EDVASDGLPSHPAIPPYHMLPQPQPGVIPQMIPLANSYAAFPLPQGVGMSPHM
LNSGNTAHIPGKTPVPFNPPQQPPQNLLNGVNINQIQFPPNQRQQFPYQFPPQQ

6

Cryptosporidium parvum EAK87793.1

MPAKKNNGGQTDRDSESGLTDRQSNKVESNIVEVPELHFH

EKYSAVNIPVVQ
EKIRYET
KEVIQ
EREVHVVKPVV
KEKIVEVPKYKVV
EKVVEVPQVVVQ
EKLIEV
ERVEVTSRSRNQSRPISPV
EKKQLDIVTQYRQVPKPVEVPIAFYSAIPVPILI
DRAVPVPMELQIIQDILCPKIEAIY
KDIPIPIPVKRTI
EKPVPI

GLYDQPELLQKYLHDPNEPLPNPVAALEQMAQQASMAAALNQQ
NMMQMMQQQGSVQQEPQE

7

Cryptosporidium parvum EAK87794.1

Articulin
Homologue of OII72157.1 in *Cryptosporidium ubiquitin*

MSNSERSGQTSGREHHARFAEDEVQEQRYVVPSLQIPEESSTSGVAE**GG**IPL
VKLVETVVPDVEVKEVVRTVPRIVPEYR

EKVVEVPVIKTV
EKIIEDPVVRYNVKYVPKEEIV
EKVVTKPVVEYKTV
EKIV
EKVTYVEEP
EKPFINAPESLPIFPPNPIEIDENTVVSITRVAVGDAVPSLIELPVPYMVPKPFVVPV
HVPVLEF
RDHFVPIPVRKRVVPKFKMTEEVYEVECV
REVPYFVYEDIIKPVPVDVEFGK
REREMDVHLMNPAELSQADFHAMWMRVNADLLE
ERRVLVGEMEI
DRMITPY
REAMDGIDSNQEPSL
ERMQSF
ERQMNLQRQLWDP

LPMSP**G**NPLMMTYLQNQWILTPTIQTQEMYTQEFFMLQQQAVYNLVTGAPQQVNL
APHQISLCGCEAAAGVVDIDSLPSGAQNLIRGLESKEVVNVDTLGPMDQTPIVTYPV
QQCIPMAVLGAAPLSMPKDIIPIKPEAIEASHHDGFSITSMCCNASENPKEEVNSESLEQ

8 *Cryptosporidium parvum* Pfs77 EAK89629.1.

MQQPQQPNDMFHTSVGDGVTATWVDPAQMGQVNNNCFPNQAHPSSGYYRLEV
DQAPLITVPVVQEIQR

RDKIIEVPQTII
KDKILPKIYHQEVIHEVPKLEVEVQ
EKEVEVPNVTVV
EKVVDLEHIVGFNPKYVPTWEV
REVPKLVPKFVGEQKVLYIEISQIQYV
DKVT
EKEVVVDVV
DKVVPKVVEIEEPIEVIRYKW
KEVYDDIPVVKYVPKFDVEVVCPAPVIVPYVQPEI
KDLPPVKVTV

GPDGRETNMAQDYNIPNNVVSSPNSIPNMNANLAQINDINSSPFPLPEGSQPVEPRM
PQFMKGNMPVNVRVMPPHPNNQ

9

Cryptosporidium ubiquitum OII72158.1

MSGKNNNGGNTDRDSDLGSNEYQSTKKVESNIVEVPELHFH

EKYSAIDIPVVQ
EKIRYET
KEVIQ
EREVHVVKPVV
KEKIVEVPKYKVV
EKVVEVPQVIVQ
EKLIEV
ERVEVTTKSRNPSRPISPV
EKKQLDIVTQYRQVPKPVEVPI

AFYSAIPVPILIDRAVPVPMELQIIQDILCPKIEAIYKDIPIPVPVKRTI
EKPVPIGLYDQPELLQKYLHDPNEPLPNPVATLEHMAQQASMAA
ALNQQNMMQMMQQQNSVQEPQE

10

Cryptosporidium ubiquitum OII72157.1

Articulin
Homologue of EAK87793.1 in *Cryptosporidium parvum*

MSNERSDQTSGREHHARFAEEEVHEQRYVVPSLQIPEDSSTS**G**AAE
AEIPLVKLVETVVPDVEVKEVVRTVPRIVPEFR

EKVVEVPVIKTV
EKIIEDPVVRYNVKYVP
KEEIV
EKVVTKPVVEYKTV
EKIV
EKVTYVEEP
EKPFINAPESLPIFPPNPVEIDENTVVNITRVAVGDAVPSLIELPVPYMVPK
PFVVPVHVPVLEF
RDHFVPIPVRKRVVPKFKMTEEVYEVECV
REVPYFVYEDIIKPVPVDVEFGK
REREMDVHLMNPAELSQADFHAMWMRVNADLLE
ERRILVGEMEI
DRMITPY
REAMD

GIDSNQEPSLERMQSFERQMNLQRQLWDPLPMSPGNPLMMTYLQNQWILT
PTIQTQEMYTQEFFMLQQQAVYNLVTGAPQQVNLAPHQISLCGCEAAAGVV
DIESLPSGAQNLIRGLESKEVVNVDTLGPMDQTPIVTYPVQQCIPMAVLGAAP
LSMPKDIVPIKPEAIEASHQDGFSITSMCCNASENPKEETNSENLDQ

11 *Cyclospora cayetanensis* XP_022589452.1

MSQGNSGCGTSMFCGSVTCGDGEV

EREPV
ERTHIRLQADQPVVA
VPVYQEIQK
RDKYIEVPQVEV
RDSIVPKVYNQSAVHDVPRVQVACG
ERGVAI
EKETIV
EKDVDVPVRVGYAPHFVPKWDI
REVPRPVPKYEGEQQVIEVEVPQIEY
KDTYV
EKEVVVDIQ
EKIVPKVTEVV
KEVEVMQYEW
KEKYQDVPVYKYVPKFDVELE

CPPPLIVPYPETRYVHDEPQTSSPFCRWSACCTQVHQEPHIRTVETAVRTEQ
QRLYPGSAQLTEAKLNYPMDQNFAADFQKQYTQEHVLRSGSGVQGPIRQAT
LSQLTPGPVYPRPTPQVGAADQQKSGARLNEKKPSFWSWLTGKKEEEPETT
SAAGKFGYPEHMPSDFASAFQNQGTSETGAATPTPKDLEEISEAKDKASDEL
EPSVVYRGSVNKPPEYGGELDPISFKLHAIEIHQFVPLPNVETPEFVKALSEGI
TTSDVSGLEKFFGGQVPAGWADPDVTGIPAPTMSDILTGNAQNVAMMNPLV
CQLSSQFAKQGFVPGGTQEVTQPGGSFRRNSSQAQA

12

Eimeria necatrix CDJ68256.1

MSQEASGCGTVPFCGSLTCDAVSCEAEQVQRAHIRLQADQPVVPVPVYQEIQK

RDKYIEVPQVEV
KDSIVPKVYNQSAVHDVPKVHVECG
EKNVAIES
EKIV
EREVDVPIHVGYAPQFVPKWDI
REVPRPVPKYEGEQQVIEVEVPQIEY
KDTYV
EKEVVVDI
KEKIVPKVTEVV
KEVEVMQYEW
KEKYQDVPVYKYVPKFDVE

LDCPPPLIVPYTETRYVQDEPETSNPYCGWSACCTKVHQEPHIKTVETAVRSEQQ
RLYPGSAHLSEANVKYPGNQDFSTHFQKNTEERVLRGSSVIDGPVRQATLSQLTP
GPVFPRPTPQVGASEKPKSSGARLSDKKPSFWSWLTGKKEEPETPTQVSKFGYPE
NMPSDFAAAFQNQGSADAGEPQPNPTELGGVPDEKDKQSDELEPTVVYRGAVNKP
PEYGGELDPISFKLHAIEIHQFVPLPNMEAPEFVKALSNGIMTSDVSGLEKFFGG
HVPPGWADPDITGIPATTMSDILSGNAQNVAVMSPLVCQLSSQFAKQGFVPGGTQ
EMTQVGSFKRNTSQPQPQS

13

Hammondia hammondi IMC8 XP_008881827.1

MYSSRPYPGAASAPGVPPMSSQYATSLPPNSFICAGPPPEGATLLDPVLE

ERIVEVI
KERVENRFIEVPEVHYV
EKVVEVPHPVIE
EKVVHVVKPVKQ
ERFKYVKKPVYL
DKVVEVPQIQYV
DKYVDVPRYNH
REKIVEVPKVLVV
ERIIPVLK

TVRRETVVYVDEDGSQTRVPSQPCAYDERYQTHVSTASSLTPFPPTPM

14

Plasmodium IMC1a SBT79437.1

Lower repeated region of ABD domain is G-rich, VI-poor and predicted α-helical (next slide). Possible insertion.

MFNACQLKSNCCNEEAEEMRQSMNGKYEDLYGEHIINENSDLKKII
EGKPDLDKAVEIGQHTEREYVAITAYQPVDIVTRTVEVPFVRTIETTV
PKIVYE

EKI
KEVPRYIAKYV
EKVVEVPEVKFV
DKIVDVPDIQYCLKYVPKVEIQENIIKRPVFHKKFV
EKIVEVPKV
KEMKRFHEVETVEYVIKYIP
KDASKKTK
KENETTACINETNDETNNEEEGRNV
ERGYIEHPNLNIRNGARLIMNGPPVVQQEGIIIEHPTTEMHTSITSSGT
RENVSCFCNKNVQGNRQTNCYSLSGSCMCNPRTMGELK
EKTNTMLHNPRIEQVFKPKIVKNIEVQKHVPISVDVPVPYMVPKPVVV
NVEVPVLKF
RDTFVPVPVRRKIIPKIKWISDVYQVDCIK
EKPYLKIQDVIKPIPCDVDIKY
RKYM

EKACAVNPNELAQDDVHAMWMRVNAHLA
EKKK
KEYGEFYPYYKNEEENGNKMEENESTNEEIC
KEEEIIYLEEDE
EREFINKNENINNTEI
KEDSNG
KEINENINN
KEINENINNK
EINENSNG
KEINNNSNG
KEINNNSNG
KEINNNSNG
KEINNNSNG
KEIHKNLSVT
KENHNIYKNEINNDFAHINND
EKEKVIAEEHIFVRNKSNIET
EKAYDIFNVS
KELRF
KDDNKMLQTNYDINLNLNEN
EKTYLFENSDIFMNEM
KEDI

EKNKNNNTSNKYIEEQLTASLYPSHPLAMTYLQNKWIQTDTLKTHELYN
NDFIRASVNANYNLQSGNIVMSDIMRNNEFLKSANPIISPFSPGNIRNLE
NYYNNLIIQNLDQQNKQSNYYEHMNNQINYDNYNNYENFENHQIKLYE
QIIKEEQQNEKSNSKCCNYFCDK

15

Plasmodium IMC1a SBT79437.1

Predicted secondary structure

1 M F N A C Q L K S N C C N E E A E E M R Q S M N G K Y E D L Y G E H I I N E N S D L K K I I E G K P 50

51 D L D K A V E I G Q H T E R E Y V A I T A Y Q P V D I V T R T V E V P F V R T I E T T V P K I V Y E 100

101 E K I K E V P R Y I A K Y V E K V V E V P E V K F V D K I V D V P D I Q Y C L K Y V P K V E I Q E N 150

151 I I K R P V F H K K F V E K I V E V P K V K E M K R F H E V E T V E Y V I K Y I P K D A S K K T K K 200

201 E N E T T A C I N E T N D E T N N E E E G R N V E R G Y I E H P N L N I R N G A R L I M N G P P V V 250

251 Q Q E G I I I E H P T T E M H T S I T S S G T R E N V S C F C N K N V Q G N R Q T N C Y S L S G S C 300

301 M C N P R T M G E L K E K T N T M L H N P R I E Q V F K P K I V K N I E V Q K H V P I S V D V P V P 350

351 Y M V P K P V V V N V E V P V L K F R D T F V P V P V R R K I I P K I K W I S D V Y Q V D C I K E K 400

401 P Y L K I Q D V I K P I P C D V D I K Y R K Y M E K A C A V N P N E L A Q D D V H A M W M R V N A H 450

451 L A E K K K K E Y G E F Y P Y Y K N E E E N G N K M E E N E S T N E E I C K E E E I I Y L E E D E E 500

501 R E F I N K N E N I N N T E I K E D S N G K E I N E N I N N K E I N E N I N N K E I N E N S N G K E 550

551 I N N N S N G K E I N N N S N G K E I N N N S N G K E I N N N S N G K E I H K N L S V T K E N H N I 600

601 Y K N E I N N D F A H I N N D E K E K V I A E E H I F V R N K S N I E T E K A Y D I F N V S K E L R 650

651 F K D D N K M L Q T N Y D I N L N L N E N E K T Y L F E N S D I F M N E M K E D I E K N K N N N T S 700

701 N K Y I E E Q L T A S L Y P S H P L A M T Y L Q N K W I Q T D T L K T H E L Y N N D F I R A S V N A 750

751 N Y N L Q S G N I V M S D I M R N N E F L K S A N P I I S P F S P G N I R N L E N Y Y N N L I I Q N 800

801 L D Q Q N K Q S N Y Y E H M N N Q I N Y D N Y N N Y E N F E N H Q I K L Y E Q I I K E E Q Q N E K S 850

851 N S K C C N Y F C D K

16

Plasmodium IMC1b XP_001348100.1

MSHYNPEQLNFVNNHRNNVKEGYFYKNNLLKENNNKMESSKNCIMVPLIN
NPSHSENNFSKNN

RDENINE
EKSKYQFEYQNKIIQVPELKYV
DKMVYDPVII
EKVKYVP
KEVIKYNIIKKPVIKNIIT
EKKVDVLQV
KEKISF
KEEEIVEDVYNYV
DKDLNTKWNESQYDNEMY
KDLTKKKNYI

GQNHLLPNNINKMGHINDRTYKHINNNITNLLEPFGPQIDVEENKIIENVFVPN
VEKVIEVNKKIDIPINLPVPYIVPKPKIIDVDVPVFKFNDKYVPVPVSKKIIPKITW
TDKIYQVDCLIEKPYLVYHNIIKMVPTDSKITVREYPKGIKKINPEELYEVDNLA
LWMRVNADLKQEHDQMKNEKYETNKKKGKGETEQLDDNISSDHTCECSES
YETYEKLSNEEFNSSNEETTIKSSNENILDTLPLHPGHPLEFIHLQNKWINQDT
TNIPDMYDQKYLDAHRNAVFNLTTQMPREAEVEAKQLLYIQKKLQQEETL

1 M S H Y N P E Q L N F V N N H R N N V K E G Y F Y K N N L L K E N N N K M E S S K N C I M V P L I N
51 N P S H S E N N F S K N N R D E N I N E E K S K Y Q F E Y Q N K I I Q V P E L K Y V D K M V Y D P V
101 I I E K V K Y V P K E V I K Y N I I K K P V I K N I I T E K K V D V L Q V K E K I S F K E E E I V E
151 D V Y N Y V D K D L N T K W N E S Q Y D N E M Y K D L T K K K N Y I G Q N H L L P N N I N K M G H I
201 N D R T Y K H I N N N I T N L L E P F G P Q I D V E E N K I I E N V F V P N V E K V I E V N K K I D
251 I P I N L P V P Y I V P K P K I I D V D V P V F K F N D K Y V P V P V S K K I I P K I T W T D K I Y
301 Q V D C L I E K P Y L V Y H N I I K M V P T D S K I T V R E Y P K G I K K I N P E E L Y E V D N L A
351 L W M R V N A D L K Q E H D Q M K N E K Y E T N K K K G K G E T E Q L D D N I S S D H T C E C S E S
401 Y E T Y E K L S N E E F N S S N E E T T I K S S N E N I L D T L P L H P G H P L E F I H L Q N K W I
451 N Q D T T N I P D M Y D Q K Y L D A H R N A V F N L T T Q M P R E A E V E A K Q L L Y I Q K K L Q Q
501 E E T L

17

Plasmodium IMC1c XP_001347324.1

MADSIKSSNSFQKLDNIDAKETSTVDRKWVALTAYQPVDVVTKTVEVPIIKTV

EKYVPKTIIQ
EKIIHVPKNVTHIV
EKIVEVPEVKYI
EKIVEVPHIHYKNKYVPKIEIV
EKVV
ERQKII
EKWH
DKIVEVPQI
KEVVRFKQIED

SEEIIKYVPRNSKNIDWEDEYKKYTESK**G**LQRYSLDQNNIYQQANSFNQFNENAYN
QNAFNRSYELLNKQSSVKSQNNASGENFSQMNFYNQYSGANFEQERSIQASNFE
PSGSMQLKRLSSEEIKPAGCCSAACT

18

Plasmodium IMC1d CAD50838.1

MELYQDNKINNVGNIEINQCDGVIKKENDLHVSVIKPITKKIIHCDNMENKM
KINAFYKPVELVEEIKNYVHKGDTHLVKLYEDFKNKKDKKVKMKNEYMTKL
KNLHILNMINNKKKENECGVVNKNNIVHFNLFNENEDVTPKIETVYIPKL

EKNIEVVSHL
KENVNVDYTYMVPKPVVIPIEVPILKF
RDHFKIIPIRKKIIPVIKYTDNIIYVDCCV
EKPYIVYENVIIPVPCDIPI
EQKKYI
DKVPPI

19

Plasmodium IMC1e XP_001351116.1

MVKCNDSNQSKNCTRNFCNVNEEDRKEEGMMSQKDNENMYNPNDIHNNNYNN
YYTNNYSV

EKNKFSQNSMNQENYENFVDCPPQNARILKPLIQ
EKIVEIMKPEIE
EKIIEVPQVQYI
EKLVEVPHVILQ
EKLIHIPKPVIH
ERIKKCSKTIFQ
EKIVEVPQIKVV
DKIVEVPQYVYQ
EKIIEVPKIMVQ
ERIIPVPKKIV
KEKIVEIPQIELKNIDI
EKVQEIPEYIPEVVQ
KDIPYTQIV
DRPYHV
EKIVEVPHVQHIYRNIVSPQYRHIPKPVEIPMAHYRTFPV
EKIV
DRNVPVPVELQIVQEFLCPKIEARY
KEIPVPVHVQRIIEHPIP
KDAMNNPHLLPLYYQED

NNIEMSTNKKNTMEQNKNKNCFIFNLNRNDSRNSSHASPSVELLLHNDKSQKQFYNN
HTNKKTSIISNPNVFNNYNQNGTLNQFNPNDINNNMEHIIHHNNNNIPSNVNFKSFHD
NVITHNKINNNMPYENNTVLKNNYSVNIPQTYTTKEFANNIYSSPSPVLSSSHTEHA

1	M V K C N D S N Q S K N C T R N F C N V N E E D R K E E G M M S Q K D N E N M Y N P N D I H N N N Y
51	N N Y Y T N N Y S V E K N K F S Q N S M N Q E N Y E N F V D C P P Q N A R I L K P L I Q E K I V E I
101	M K P E I E E K I I E V P Q V Q Y I E K L V E V P H V I L Q E K L I H I P K P V I H E R I K K C S K
151	T I F Q E K I V E V P Q I K V V D K I V E V P Q Y V Y Q E K I I E V P K I M V Q E R I I P V P K K I
201	V K E K I V E I P Q I E L K N I D I E K V Q E I P E Y I P E V V Q K D I P Y T Q I V D R P Y H V E K
251	I V E V P H V Q H I Y R N I V S P Q Y R H I P K P V E I P M A H Y R T F P V E K I V D R N V P V P V
301	E L Q I V Q E F L C P K I E A R Y K E I P V P V H V Q R I I E H P I P K D A M N N P H L L P L Y Y Q
351	E D N N I E M S T N K K N T M E Q N K N K N C F I F N L N R N D S R N S S H A S P S V E L L L H N D
401	K S Q K Q F Y N N H T N K K T S I I S N P N V F N N Y N Q N G T L N Q F N P N D N I N N N M E H I I
451	H H N N N N I P S N V N F K S F H D N V I T H N K I N N N M P Y E N N T V L K N N Y S V N I P Q T Y
501	T T K E F A N N I Y S S P S P V L S S S H T E H A

20

Plasmodium IMC1f CAD52638.1

Articulin

MLKNTTKCDNIIKREQNRVLLQKKLSKDSVTSTKFCDSSEEREGSSNTSSNV
SFKILEESNITKNLNEDKSYSFECDKEETLRKDYKKLNKKNSNKINKSNSLNQ
TNEEIIRDKTSVQNKKIHHEVLEEQEKDNSIKLNKNKVGIYFDNTTNSINNNIQK
DNSDTLGTESYSTHKSNLNEVLNVSGDNSKHLLAPISSNDIIKKEDSNIYYNLN
ENSTYYYPNNNSKNYINKFLNNYNTPNVVDSFSYCGTNFHSGNISTSYNDRY
LNGYSNNYMINNSINSSINSSINNSINSSINNSINNSIDNSINNSINNSISHFNTSY
PNSNNNNNNNNNNNLNYSMSNNFNSYPNNILNYNLNNQFINAYPINNLNYNSTS
HFNMYPNKNMLINDTLNNTNSYINNMKYSGNNIMSEQNNILNETEFIGSYHNSS
APIISNQENNISNLGNTSAYVNDNTTYGNISGRNMYSGVINYENITDDNKMIKSD
NNVVPTINTFYSSKNIGESTNGNVVVRKNYVDNTYDNNSNEEHSIEN

KEIYKSKSVSVSENETIKNDNVSVSVS
EKVSSHTDYSRAKHSSHVV
DKRIVHEGFDTIKIPKY
REVEIV
EKIVEVPVVHKVNKYVNKYEI
KEV
EKVVKKHINKYVETKIEVPELHYQ
DKIVEVPELQEVIKII
EKPEIK
ERIIYKNKIETKIIPKYIEVPVVKIVNRYEEYDDVGEVVKAVPVKKIVEIPNEII
KKVKVPVKKII
ERPNYVPVIKY
RDIPI
EKIRYVPKIETVELVKKIPKIIDVPVPVKVPKIKVI

DKPFYVNKYVDKPVVIPVSKTIKPVYKYEGKKIIEIPIHKPYLVTHDTVVNKNVENNMN
SGRYSVYAKRLDLNSFDIKRNELFNLVNKDNINFQRSMSVDDFVNNNSYVDGKEKY
TDKLNENSVFNNYNGIGNKDNLDNMNKIKFTQSLNNNNNMSTTKHSNMEVSSQGY
GYINDGKENMYNNYNNMNNLNIKGSEDASFHTKKSIFDNLNNDNINNINNNNNINKN
YEENRKSSYGTSIHNTPNILPNRHNSMYPSNGNHMGEFISLKSFQNKKYSNSTIVDS
EKAMNYTYGNFLSPTGVYNNGLYMENKDKNNMVYENNKKDMNTINYNNNNNNNNN
NNNIYYSANNKSPNNIHKLNMSNSMMKTNTFGNYPNIYRNSYMENKPEDMYLNRYT
TKSNIINNNNNNNNNNNNNSNNSNNSNYFYNGRNSSINNAFAYKQYYDSNKNGNNQH
RLSHPSNMVHNINNSRTRSPSTCSADGISAYVVEYVGDENIMYNNGNTPFSSNGLLN
NLGKTMEGSNYSFNGVPVNQK

21

Plasmodium IMC1g CAD51621.1

MCSTNKNLACCKGDNVFDGQINGNESYPQVVNKQLPPKVLEPIIQNKIVEIP

KEVYL
EKIVEVPQIKTV
ERIVEQIRPVIKYKNVYKPKIVYV
EKVKNV
DKIIYQ
EKIVEVPQIKTV
EKIVEVPVYVN
RERIITVPRYMVV
EKVIPVLKTSKRESIMEVPEVNCPHIDIS
KEVE
DKEEIPINEL
KENQ

TISLADEKEIQILNDLTSQKVDSNATINMEGEQDTTVDTITQENFCGTVSCNFL
PNYPNFSKIGNPLCKGGPEKEKRFSSISIYKSKDSGFPSIRIAKTPQMFQRNLY
CSYA

22

Plasmodium IMC1h CZT99374.1

MSYQDVQEKKNEDINYDEIASSMLEKMDKHINGEKQPTIHYENLNVQEQEGA
HAYYSNQDINNINSSMSFLKNVKELNSSNILDHRTTTNDVGYPRRVMTALGP
LPLPEEFKKNIP

EKFVAKPIIE
EREIYVSK
KERKQ
REIEIPHVKYEHTF
EKVKKQLKVNKLVPNVTQVI
KEVP
KEILKPVIE
EKIIEVPQGVKYVEVPVEVPCLYPPKIMPKVVTQYV
ERIVETIKPVVQ
EKIIEVPQTVIKQVPKIKTVEVPYYVPRYV

EKIIEVPFKPNGEMPKATHLPISISDSLPPLKPTHILSAQNEKNITKHNNNMNSNNMN
NMNPFQTTNFMMPLMNCFQQNPDPNTLKNSENGIPNMNMSPTILRPINSRDTPNN
NMNPQEHIKNNITPYPNNSLNSNFTNYNMPQNLYSQIENLPHAHNLPDGFQWQYP
DGVSNNPLCNTRNMPSLVVTCPPQIGRVTCTRKDIQPDILTKTGVYEFDGFKKKGT
SQSLFPPLAHHNRVPFLPAPAAPGTPDIENIEKNM

23

Plasmodium IMC1i CAD51138.1

MQGETINVPSSSVPYTFEHSKHLKSKILKPIRQEKVVKVPVTQYVEKIVEKEE
IKYVNKYVDVIKPIITYKTKHISKPIYLDKIKYQPKVVEKEKIIHIPKIEYRN
KIVEPVYVH

KENII
EKKVPLIV
ERVVPVLKVNRV
EKEILTNSFEIPEICEMT
KDENNIHIGKNVHETNQQISCNVS
EKGNSFVSEINK
EKSDIYNIETYRHMQTTNASPKVYSQLEISSSNMDNQNVETLK
DKNNVVDSNDTSDEEEYNEGNLEHKNETRYHEATNEYSNYDFTS
EKNQKINDEENNS
KELINNNNMEENYYDID

ERFKDSNISHVSIHLPEEKGIEQQSYEQNYMNSCNNNYNIIESNLGDTSGSVYN
NINTYGTYYSDNMNPQFDKTFVNQSYNGSKGYIQKIMQRNHIPNYSTNTMEHLK
IVEEKHPNSSNLHISNERFSTLPSVYGARSVTGKSNFVPSYANSNGQAIVSVRP
ATIVEYVPKSKKYKASLCNFINKCCGNM

24

Plasmodium IMC1j XP_966198.1

MEDKKHCTLTFNDWCKKKEKEDDGLNTGYINKYNEIYFNKDGKHNIYENINE
RPIINLEGDQPVFNVPVYQDKYIRDKIVECVNYEIQDVVQPTFYSQETKHDVP
TVELLYK

EKKVNIPQ
EKII
EKPVEVDMPIGYTPVFSPTWDV
REIPRVIPKYEGEQKIIQVEIPQIKYV
DKFV
EKEIIVDI
KEKIIPRINEV
EKEIDVVKYKW
KEKYQDVPVCKYVPKIDVELDCPPPLIVPYP

AVHFHNTSEVMNPHQKALDIPSEVLLKNNNIFGYDINMNGNHRNVYNDSNMHN
NNNNNNNIYNSSNNYNGMSNFHVDESVKKSLLDVARLTGVQKDNDAKYNEFM
KNKNLERRKKKKNWPFCYFKKDTIKENDEMYNGQNYSNYQNNCCNSYKGEDE
NNYDIDPNTGYPKSMPKDFASFFKQDLNSVKKQMEKKTNTKSSYSSDFIEKSPV
NPSIEYLGKVDKPPIDAGKLDSISFKLHAIEVHQFIPVPSLPKPRFLDLVPSQQYEQ
NDISSLQNVFGQVPEGWVDPQITGFIAPMMNDVLHGNIQPQSPLFNNLSTEGYD
SSSKNRRTPRINAPSNIHNNNDSSNVFSFDHDNYHNEGGEGSFDQAYYDEYTN
EKYDNEENEYHTEYIENNNDEDTNGYIKSPNDLTYNTDSSINEGKQNEDIN

25

Plasmodium IMC1k CAD52548.1

MDEKSEINYDEGADPSYSMHMSESHSAYEEVNKTSSDSEFVDDDYSKVISENN
LESDNVKYNPDLYNLGKARQYLCPKVQEYGIPGQVSPYHFPYNEEDYLSNVDT
EYDENGFPIRTNIDDNDNNEDNKKLWSWTNDGKLKLLCSQPIIPVSVVQ**G**ILR

RDKIILIPQVEVTDVVIPKVYNQNIKHDVPTLNIEIMKSNVNIPNVKYV
DKEIIIPIITGYTHKFVPKWDIHEVPRPVVKYIGEQKIVEVEVPEIKYI
DKFV
EREVIVDTV
EKRVPKIIEVPKYVDEVKYVWKPI

EKIVYVQKLIPKFDVNLECPPPLIVPYPVQRIKQISPVMVKKNNTEIPDYNYYDLNSP
E**G**SLPIPEEYIHHYSRKQKMNKGLLSTCCDKKIVTEEEKNVQFVSVPLSNEHESVC
VDEEIKKNINMSQENINTQLNNNMDNFIYDSDIKKNKDKMNSSINGKQQLYINS

26

Plasmodium IMC1l CZT99881.1

MSIPFHSSCFSKAPKYALKQVHENIGIPVFHKTPKIVEIPEIREITKFVESVKVVDVPIE
QVRIVPKLKVREVEKIRHVP**G**PIEYIDIPQ

ERIIHKVYKEI
REKIHEIPQIEDIEIEVPIYVPTPIGPP
KDIHVNIPLPYDIPQFYYTPDNRYSHILPCDILPHGTQMPDINFE
EKSFNDYSE
DKNICMTHNDNIIHSNRRMEYNKNENNICNNNTFDNNMN
DKNIYDSNIFDNTTYYNNTIDNNIYDDNTYGNNMYDNITTYYEDQENNIHS
KEKDKYINNNYNNKYINDYYKTIPTK

GILKKENISTSPGIKEQYYYSYSKHNSKNAGKAHHYDKDTYDDMSTWDNYAENMNYI
YEDDENENEYKNRKKRVTELIVKKKK

27

Plasmodium IMC1m CZT98544.1

MCDNICEDKKNYMFGRPPFLIPQNVADSPSTTVELDYPLYETAPN

REIKNIIQETTINVPKVEYKNKIVEIPRVEYRTYPLI
KEVETPIYQ
DKYVYKNVEVPNKRLRIKPVY
KEVKVPQIEYINKYIKRKYKRYKYIP
KEIKVPFRPR
REIYNEIPIPRYIPENIEN

CVEKKKMLDTAYRGELPLFQGYDDNIINMMNPFCTYNQSDNLYNNQMCEGKRKKEK
IWNYDILNCLCINNNENGTYDIDPNNIYPSYDNRLNEQVYDHNKIYSSSPYEQLTYTPP
YHIILDSDQKKEENIFCHKVIEVTSSLLAFTGIAIIMMGKLSLHGISILMKNIKSRNNNTQV
EKIINEIKDDEDTLCPPVKKNNNMI

28

Plasmodium IMCn XP_001350612.1

MSYQDVQEKKNEDINYDEIASSMLEKMDKHINGEKQPTIHYENLNVQEQEGA
HAYYSNQDINNINSSMSFLKNVKELNSSNILDHRTTTNDVGYPRRVMTALGPL
PLPEEFKKNIP

EKFVAKPIIE
EREIYVSK
KERKQ
REIEIPHVKYEHTF
EKVKKQLKVNKLVPNVTQVI
KEVP
KEILKPVIE
EKIIEVPQGVKYVEVPVEVPCLYPPKIMPKVVTQYV
ERIVETIKPVVQ
EKIIEVPQTVIKQVPKIKTVEVPYYVPRYV
EKIIEVPFKPN

GEMPKIATHLPISISDSLPPLKPTHILSAQNEKNITKHNNNMNSNNMNNMNPFQT
TNFMMPLMNCFQQNPDPNTLKNSENGIPNMNMSPTILRPINSRDTPNNNMNPQ
EHIKNNITPYPNNSLNSNFTNYNMPQNLYSQIENLPHAHNLPDGFQWQYPDGVS
NNPLCNTRNMPSLVVTCPPQIGRVTCTRKDIQPDILTKTGVYEFDGFKKKGTSQS
LFPPLAHHNRVPFLPAPAAPGTPDIENIEKNM

29

Plasmodium PIP3 CZU00005.1

MAESSYRDTQLFKPNGVNIFDSNLGMNNKISENISAEELFQSKNIY

RDVKSKVIPGPIKTTTV
EKVTKIPKIIF
RERQ
KDIVK
KEKKIIH
KEVEI
EKIVEVIEN

KDEIVYNEVKVPKYIDVPILQPRQEVHYQQISKNVPRGVELVITQTLEVPRIKP
KYVEIPVPIYVPCYIEVPIPAQYIPIEQNDDKEHFTSGISNASKLSKHPHMQNN
SMEFPHHYNNPYVMNPNLNMNDDKSVCGSSKASIQNISSEQKNVEKMVIAG
NL

30

Plasmodium PIP2 CZU00008.1

MDKDKDKSKNTPLLYQDTMNDLGDGLQNMDTLRLNRNGNTGNGNKNKTNGSK
GVK**G**EY

EKK
KETNGHDDYV
KESLNRMLESHIDQYFKGLDYYKNNPKCKIDPKIHLHGSEEIENYIV
KEKIFVTTYFQYIDVYENAINVDEDL
EKEEIVYVEVPKYVTKYKPKIVTNII
EKTIEIPSGEEIKQPKYNTVNVPYVIPNIVENEILVVLKKIIQPEIEITNEELEIEV
EKYIPRLVPVNVYVPRYFGISAKAKGEPEESVRYVDLTQDQIDELM
KELNPHLNE

LKVFNETQLKRMDEYMRESQMQARAHNFEPPQPQLITYDES**G**HCQSYDYSEFHRF
KETCIKELTH

31

Toxoplasma IMC1 XP_002368045.1

.

MFKDCADPCSDCCQPAEQQRGQATLPSHVVLPQTSDSPAIHVTAEASQRLSS
PRALQQLHGTQESVRSVTLEERADRSRTVPIGEET

ERQWVAITAYQPIDTVTKTVEVPVVRTVETYVPKVVVE
EKIVEVPKYIPEYV
EKVVEVPEVQYV
DKIVEVPEYQYEYKYVPKIET
KENIIQRPKYETKYI
EKVVEVAQV
KEVVRYQDVEEVEEIIRYVP
KDSVVPEEWKRIQEEQ
<u>DKRE</u>AEL
<u>KEKE</u>A
KEEEELKGLLEE
ERKLHE
ERV
REQIKVQQQIIQQQLSARAEQ
ERLL
<u>KERRE</u>AE

MVHFPAVEGAPPLPTIPKVEQVFKPKVIKQVEIQKHVPISVDVPVPYMVPKPVVV
SVQVPVLKFRDHFVPVPVRRRVVPRIRWTNEVYEVECIKEKPFLQVQDVIKPVPC
DVEIRVHEFVERAAPINPAELSQADIHAMWMRVNADLAEKRKQELGDKYPYVK
HPAGTVFGEPGCSEEEGEVESAQAEEGGQPAGAEPLALHPGHPLNMTYLQNEW
IKKSTVTTHEMYTPEWFEAHQKALFNLTMQQPTQVQLSAEQAAKLQQQEAQF
GDAPWIEEAHAGVPMKPAAPEPEAAQCCVMCAVCGGDGVCRCQC

1 M F K D C A D P C S D C C Q P A E Q Q R G Q A T L P S H V V L P Q T S D S P A I H V T A E A S Q R L
51 S S P R A L Q Q L H G T Q E S V R S V T L E E R A D R S R T V P I G E E T E R Q W V A I T A Y Q P I
101 D T V T K T V E V P V V R T V E T Y V P K V V V E E K I V E V P K Y I P E Y V E K V V E V P E V Q Y
151 V D K I V E V P E Y Q Y E Y K Y V P K I E T K E N I I Q R P K Y E T K Y I E K V V E V A Q V K E V V
201 R Y Q D V E E V E E I I R Y V P K D S V V P E E W K R I Q E E Q D K R E A E L K E K E A K E E E E L
251 K G L L E E E R K L H E E R V R E Q I K V Q Q Q I I Q Q Q L S A R A E Q E R L L K E R R E A E M V H
301 F P A V E G A P P L P T I P K V E Q V F K P K V I K Q V E I Q K H V P I S V D V P V P Y M V P K P V
351 V V S V Q V P V L K F R D H F V P V P V R R R V V P R I R W T N E V Y E V E C I K E K P F L Q V Q D
401 V I K P V P C D V E I R V H E F V E R A A P I N P A E L S Q A D I H A M W M R V N A D L A E K R K Q
451 E L G D K Y P Y V K H P A G T V F G E P G C S E E E G E V E S A Q A E E G G Q P A G A E P L A L H P
501 G H P L N M T Y L Q N E W I K K S T V T T H E M Y T P E W F E A H Q K A L F N L T M Q Q P T Q V Q L
551 S A E Q A A K L Q Q Q E A Q F G D A P W I E E A H A G V P M K P A A P E P E A A Q C C V M C A V C G
601 G D G V C R C Q C

32

Toxoplasma IMC3 PIL96575.1

MSDAGTPPAVQGELSQPQERVEVVVQPISESPDASSEAEQAGVAAGAFHAEV
PYPYPPVSTGMPAPEQPAEMHLPANGQLPEIAEGAETEGLAPVARPPPQVMTA
LGPMPLPPEVRQKIP

EKFVAKPIVE
EREIYVSK
KEV
RERTIEVPHVHYEHKFAEVARSLKIKKLVPRV
REVV
KEIP
REVYKPVIE
EKVIEVPQGVKYVEVPVEVPCLYPPKIVPKPKVQVI
ERIVETIKPVV
KEKIVEVPQTVVKQIPKIKTVEVPYYVPRYV
EKVIEIPYQPPD

AATLPPLMVGGLPSRVSATMPTPFQLGPAPAGHANEVNLPFEARVDVTVSQSPA
GPHSPLSASPQTGTAASLKLGRPATTLVNPDRSAPAIDLPEGFTWGPPPNAPPGT
FPAPAMPGLPGAAGLPVGAADGLNMPPMMITCPPEVGNVKFAGFLADPDILTRR
VMAYQGFKKEGTFQMLFPPLAPQPGIPLDQTGAPDVNTIVSVNEQMAGQSRHHL
IEDLQQQVESEFDQKVNASAMSCTPWASPAVQEREVVYEQ

1 M S D A G T P P A V Q G E L S Q P Q E R V E V V V Q P I S E S P D A S S E A E Q A G V A A G A F H A 50
51 E V P Y P Y P P V S T G M P A P E Q P A E M H L P A N G Q L P E I A E G A E T E G L A P V A R P P P 100
101 Q V M T A L G P M P L P P E V R Q K I P E K F V A K P I V E E R E I Y V S K K E V R E R T I E V P H 150
151 V H Y E H K F A E V A R S L K I K K L V P R V R E V V K E I P R E V Y K P V I E E K V I E V P Q G V 200
201 K Y V E V P V E V P C L Y P P K I V P K P K V Q V I E R I V E T I K P V V K E K I V E V P Q T V V K 250
251 Q I P K I K T V E V P Y Y V P R Y V E K V I E I P Y Q P P D A A T L P P L M V G G L P S R V S A T M 300
301 P T P F Q L G A P A G H A N E V N L P F E A R V D V T V S Q S P A G P H S P L S A S P Q T G T A A S 350
351 L K L G R P A T T L V N P D R S A P A I D L P E G F T W G P P P N A P P G T F P A P A M P G L P G A 400
401 A G L P V G A A D G L N M P P M M I T C P P E V G N V K F A G F L A D P D I L T R R G V M A Y Q G F 450
451 K K E G T F Q M L F P P L A P Q P G I P L D Q T G A P D V N T I V S V N E Q M A G Q S R H H L I E D 500
501 L Q Q Q V E S E F D Q K V N A S A M S C T P W A S P A V Q E R E V V Y E Q

Toxoplasma IMC4 PIM03237.1

MFSECCQPCAPCGEGEPGAAQPTQVPGSRLVSGVVATQGEARILEPLVQ

ERVVEVL
KEEIQ
ERVIEVPQVQYVDRIVEVPQHVVH
EKVTHVAKPIIQ
ERVKHVHKPVYQQKIIEVPQVKVV
DKIVEVPQYVYQ
EKIIEVPRVVVQ
ERVIPVPRKVV
KEKIVEISQVDY
RDVVV
EKPLEAVEILQEEEVVEIPKPVVV
EKVVDV
EKVVEVPHIQHTYRNVMTPQYRHIPKPVEVPMTHYRPIPV
EKIV
DRNVPVPVELQIVQEYLCPKIEPRY
KEVPVPVHVQRTIEHPVP
KEAM

GNPKLLPLYYQGTQEGVEGIVTPGHPCYSLSACCMPRREQQPIELGTMQGTV
EVMAQQGYPTMPVPQGWDGAAPPAGWEGWSKEHIPTALRSDQHSSGAMTP
VSAGAAVGSAPASPARGPEDNQGQQAPHHQAGGEGPFVQVSVTPLAPGQQ
TQVNIN

1 M F S E C C Q P C A P C G E G E P G A A Q P T Q V P G S R L V S G V V A T Q G E A R I L E P L V Q E
51 R V V E V L K E E I Q E R V I E V P Q V Q Y V D R I V E V P Q H V V H E K V T H V A K P I I Q E R V
101 K H V H K P V Y Q Q K I I E V P Q V K V V D K I V E V P Q Y V Y Q E K I I E V P R V V V Q E R V I P
151 V P R K V V K E K I V E I S Q V D Y R D V V V E K P L E A V E I L Q E E E V V E I P K P V V V E K V
201 V D V E K V V E V P H I Q H T Y R N V M T P Q Y R H I P K P V E V P M T H Y R P I P V E K I V D R N
251 V P V P V E L Q I V Q E Y L C P K I E P R Y K E V P V P V H V Q R T I E H P V P K E A M G N P K L L
301 P L Y Y Q G T Q E G V E G I V T P G H P C Y S L S A C C M P R R E Q Q P I E L G T M Q G T V E V M A
351 Q Q G Y P T M P V P Q G W D G A A P P A G W E G W S K E H I P T A L R S D Q H S S G A M T P V S A G
401 A A V G S A P A S P A R G P E D N Q G Q Q A P H H Q A G G E G P F V Q V S V T P L A P G Q Q T Q V N
451 I N

34

Toxoplasma IMC5 PIM03867.1

Localized to basal complex (Anderson-White et al. 2011)

MVQFAHGPESVLSSQRGSAATWSSVQVPTSHLAHPLSSNVRPTALQGIVSPQ
PVQRSLVVYSRRQQQPRLTDSLSSDSLQLSQLTGSVAQRPLRYRCQSDTVAL
DSRSQSELSDTEAASTRAFAFHELRSSPQGHPLSVSSPAACVAHRTACHVPA
RLHFPSLASETFPENSDRSSAPSGSEDLDLTRGISGDRSSSTVSEASFAPHPQ
APQAEAAAASSTFSPASLSLILAAAASFQKPSKSPSTRTKRPAPAPRPPVRVMS
RLLAPPMTPVKAAGPTHGVSALPGVHAPSVPAHITPSAPGIGGPEGPLIHHGIA
GSGIVVAPPTPRSGPSPMGAPPTNNHLLPPQAPRTGPPVETSSTH

DRQWVAVTAYRPVDVVTKTVEVPVTRTVDVLVPRPVIQ
EKIVEVPKFVPHYV
EKILEVPEIEWV
DRVIEVPEYFYSTKYVPKVEI
RENII
ERPLYQ
DKWV
EKIVEVPRVEEIVRY
RDIVEAEEVIKYIPK

GHSEEEWRGAPIFSVPPEHAPPLPPKWVSPGVAPDRIPGYCPPGDCFAAADGSV
KGRTKLVEHTSPAFPSSPVNGVCHAGPFWTLDCRNVGE

```
  1 M V Q F A H G P E S V L S S Q R G S A A T W S S V Q V P T S H L A H P L S S N V R P T A L Q G I V S
 51 P Q P V Q R S L V V Y S R R Q Q Q R L T D S L S S D S L Q L S Q L T G S V A Q R P L R Y R C Q S D T
101 V A L D S R S Q S E L S D T E A A S T R A F A F H E L R S S P Q G H P L V S S P A A C V A H R T A C
151 H V P A R L H F P S L A S E T F P E N S D R S S A P S G S E D L D L T R G I S G D R S S S T V S E A
201 S F A P H P Q A P Q A E A A A A S S T F S P A S L S L I L A A A A S F Q K P S K S P S T R T K R P A
251 P A P R P P V R V M S R L L A P P M T P V K A A G P T H G V S A L P G V H A P S V P A H I T P S A P
301 G I G G P E G P L I H H G I A G S G I V V A P P T P R S G P S P M G A P P T N N H L L P P Q A P R T
351 G P P V E T S S T H D R Q W V A V T A Y R P V D V V T K T V E V P V T R T V D V L V P R P V I Q E K
401 I V E V P K F V P H Y V E K I L E V P E I E W V D R V I E V P E Y F Y S T K Y V P K V E I R E N I I
451 E R P L Y Q D K W V E K I V E V P R V E E I V R Y R D I V E A E E V I K Y I P K G H S E E E W R G A
501 P I F S V P P E H A P P L P P K W V S P G V A P D R I P G Y C P P G D C F A A A D G S V K G R T K L
551 V E H T S P A F P S S P V N G V C H A G P F W T L D C R N V G E
```

35

Toxoplasma IMC6 PIL99959.1

MAQTAPNQSAPTSGFSPMASMGSFAVPSAPPSPLFPTPAAFPGAAPQSQTVP
GVATMGSVAGSEVTPFPMYNWHQNRVPVNSGGFFGQCCGPAQVGSDSSSE
TYDRSYEENMWRWTRDGKLQLRCGQPVVPVPVIQEIHRR

DKIIEVPQVDVVDAVRPKVYNQGVEHEVPVMQINADHEDFDVEQIKYV
EKEVVVPIVTGFTHKFVAKWDI
REVPRPVVKYVGKQEEIEVEVPQVKFV
DKVVEHEVVVDTI
EKKVPKIIEVPKYVDEVKYVWTPV
EKIVHV
ERFVPVFDVSLECPAPLIVPYPMQAV
KEMPAVMVR
KEVPEAAITEQ

GFDIVSPPGTLRVSDEYVRAHHQFAEENAALCGAGTACGAPQKEPEIEFRTANQM
KEARAAAQEGHLERGGSFVSQVGTESQVPRPAATDEQAEGSADAGDKQGSRGS
SFNSEGEVH

36

Toxoplasma IMC7 PIL98906.1

Expressed in “mature” cortex and not in budding daughters.

MEFTADNAAPLTEQNAPQDVEAVKPDVIENAAETANEESAAPTLKRLVSK
TTHIKTVTESLSRKQT

KEITTNTLHMGW
KET
KEAFLAPRSTITHADGSESEIARFVPSVQVVDLPLDLVYTVPEVKTRMVNY
IFECPARGHSRLVP
REFPVDTPFVVPQYQDVSVPVVMSQTFVPELQETSKVVQVPVARYVPKL
VPVDVFVPRPVAIPIKAGEVTQVSKNTVI

SDDLMRQLSVEMNPHLEALNQFNAQQAQVMNNVVARAQELATQMDCPVP
SREKIEVNASAGSGAGATIVDEQGNKQLQLDMGGKELRSVEMVFKRLHDDI
NTSKCVEENMQITEDIILCVHRDLNGGRLESFPSGNAGIPMIFRAPDQKPSV
ANGLPERVPLRSLTNDLSHAAAAQNESATTTGEASLPTALNAEEQTTAVPIAA

1 M E F T A D N A A P L T E Q N A P Q D V E A V K P D V I E N A A E T A N E E S A A P T L K R L V S K

51 T T H I K T V T E S L S R K Q T K E I T T N T L H M G W K E T K E A F L A P R S T I T H A D G S E S

101 E I A R F V P S V Q V V D L P L D L V Y T V P E V K T R M V N Y I F E C P A R G H S R L V P R E F P

151 V D T P F V V P Q Y Q D V S V P V V M S Q T F V P E L Q E T S K V V Q V P V A R Y V P K L V P V D V

201 F V P R P V A I P I K A G E V T Q V S K N T V I S D D L M R Q L S V E M N P H L E A L N Q F N A Q Q

251 A Q V M N N V V A R A Q E L A T Q M D C P V P S R E K I E V N A S A G S G A G A T I V D E Q G N K Q

301 L Q L D M G G K E L R S V E M V F K R L H D D I N T S K C V E E N M Q I T E D I I L C V H R D L N G

351 G R L E S F P S G N A G I P M I F R A P D Q K P S V A N G L P E R V P L R S L T N D L S H A A A A Q

401 N E S A T T T G E A S L P T A L N A E E Q T T A V P I A A

37

Toxoplasma IMC8 PIM03868.1

Localized to basal complex (Anderson-White et al. 2011)

MYSSRPYPGAASAPGVPPMSSQYATSLPPNSFICAGPPPEGATLLDPVLE

ERIVEVI
KERVENRFIEVPEVHYV
EKVVEVPHPVIE
EKVVHVVKPVKQ
ERFKYVKKPVYL
DKVVEVPQIQYV
DKYVDVPRYNH
REKIVEVPKVLVV
ERIIPVLKTVR
RETVVYVDED

GSQTRVPSQPCAYDERYQTHVSTASSLTPFPPTPM

1 M Y S S R P Y P G A A S A P G V P P M S S Q Y A T S L P P N S F I C A G P P P E G A T L L D P V L E
51 E R I V E V I K E R V E N R F I E V P E V H Y V E K V V E V P H P V I E E K V V H V V K P V K Q E R
101 F K Y V K K P V Y L D K V V E V P Q I Q Y V D K Y V D V P R Y N H R E K I V E V P K V L V V E R I I
151 P V L K T V R R E T V V Y V D E D G S Q T R V P S Q P C A Y D E R Y Q T H V S T A S S L T P F P P T
201 P M

38

Toxoplasma IMC9 KYF43270.1

Articulin. High expression levels. Localized to basal complex. Predicted secondary sequence next slide.

MEHGGFLSSRGLRSEPTQPPRLPAENGRSYIPSAPHLGGENESTWQPTAPVA
VQSPCVRPRLHSEATVYPPFPEAAFPPSTCVPTFQQRVSRLSADSSCFPPHGG
MASSSAPVDGSGFSGDLHSHSFGAHPLQGPASRMQPWRRQHGPEAPAFLAE
RTRVWGSVAETPARAEGTKRQEVTTFASMEETGLCSAPQEDMRPPSAPFQP
RDPGIKGGALCPEEQEQPYRETSPPPVSFGSLSALRQYARDQEASGCLGATR
SLPEASRGAAEGTNEARGPASGPQENSEKGRANAVDPRVSSSISGSRKYDSV
SQVYIP

EKLQCEPQVVETVITVPRVMYEQKITEVPQVMV
RERVLEVPNIVR
KEKVVTIPKIEYQ
EKIVEVPVVKYV
DKVVEVPQCVVA
EKFVTTDEVIRQ
EKIVPIPKIEIV
EKIIQTPKVIQV
EKIVDVPRIEY
REVEV
EKIVEVPQIEIKYV
EREVPVPQKVIRHVPV
DKIVEVRQKKVV
EKIVKIPVPRYVEVPKYIEQPVP
REKIVRV
EKKI
ERKVPTPQPQDTYQEV
EKPVYVTKYI
ERRVPVPT
ERIVEEHVQVEVP
REVIVKKPYDVVRLVQKQVEV

AVPFVSGDPLILTAEGYIPFQEFAQKSQVLTAKDRQFYERQSEALGSVHASNVDTQ
PSSPSPSLSLTSTPEADGFRGAQARLPEANAAKGTSSVAGLSQTTQQSASPLSPR
NSPTSYSTCSVKPNLSQAVN

39

Toxoplasma IMC9 KYF43270.1

Predicted secondary sequence

```
  1 M E H G G F L S S R G L R S E P T Q P P R L P A E N G R S Y I P S A P H L G G E N E S T W Q P T A P
 51 V A V Q S P C V R P R L H S E A T V Y P P F P E A A F P P S T C V P T F Q Q R V S R L S A D S S C F
101 P P H G G M A S S S A P V D G S G F S G D L H S H S F G A H P L Q G P A S R M Q P W R R Q H G P E A
151 P A F L A E R T R V W G S V A E T P A R A E G T K R Q E V T T F A S M E E T G L C S A P Q E D M R P
201 P S A P F Q P R D P G I K G G A L C P E E Q E Q P Y R E T S P P P V S F G S L S A L R Q Y A R D Q E
251 A S G C L G A T R S L P E A S R G A A E G T N E A R G P A S G P Q E N S E K G R A N A V D P R V S S
301 S I S G S R K Y D S V S Q V Y I P E K L Q C E P Q V V E T V I T V P R V M Y E Q K I T E V P Q V M V
351 R E R V L E V P N I V R K E K V V T I P K I E Y Q E K I V E V P V V K Y V D K V V E V P Q C V V A E
401 K F V T T D E V I R Q E K V P I P K I E I V E K I I Q T P K V I Q V E K I V D V P R I E Y R E V E V
451 E K I V E V P Q I E I K Y V E R E V P V P Q K V I R H V P V D K I V E V R Q K K V V E K I V K I P V
501 P R Y V E V P K Y I E Q P V P R E K I V R V E K K I E R K V P T P Q P Q D T Y Q E V E K P V Y V T K
551 Y I E R R V P V P T E R I V E E H V Q V E V P R E V I V K K P Y D V V R L V Q K Q V E V A V P F V S
601 G D P L I L T A E G Y I P F Q E F A Q K S Q V L T A K D R Q F Y R Q S E A L G S V H A S N V D T Q P
651 S S P S P S L S L T S T P E A D G F R G A Q A R L P E A N A A K G T S S V A G L S Q T T Q Q S A S P
701 L S P R N S P T S Y S T C S V K P N L S Q A V N
```

40

Toxoplasma IMC10 XP_002367942.1

MSQFQQPQSPAGGSMFITGTTENAEV

ERVNVRLQADQPVVPVPVYQDVYK
RDKYIEVPTVELNDTIIPKVYNQSAVHEVPKMDIAFQ
EKEVAVHT
EKLV
DRDVEVPVLVGYAPQFVPKWDV
REVPRPVPKYEGEQEVIEVEVPEIEY
KDTYV
EKEVVVDV
KEKIIPKVTEVV
KEVEVVQYEWKQQYQDVPVYKYVPKFDVELDCPPPIIVPYPETRFV
KEDP

SVVQPYCSPLTACCPGTATHKVVTTDRLYVRGTDGAGASQPSAPLVTVGGAF
HSMSPDVQMQSEAIPSAHGVLPADYVAQGQLIGDAPGYSSEGQLPNIQLAPG
PVFPMPSPQVEQPASSFGGSSSGSEAEEAKPTASEKQSFWNWLFGKKEETG
KVAEQKKQEAIDYTAHFQQLHQQGTPSHKAEGQAGKDDTATNLEPSVVYKGV
VSRPAVFAGNLNPISFKLHAIEIHQFIPLPNVRVPEFVKALPDGLMATDVSGLDR
FFGSVPTGWADPDVTGIPAPMMSDILAGNIQASTTSPLINNLSEKFVQQENTVQ
QAQTDARAASGVGLGEEAQISA

1 M S Q F Q Q P Q S P A G G S M F I T G T T E N A E V E R V N V R L Q A D Q P V V P V P V Y Q D V Y K
51 R D K Y I E V P T V E L N D T I I P K V Y N Q S A V H E V P K M D I A F Q E K E V A V H T E K L V D
101 R D V E V P V L V G Y A P Q F V P K W D V R E V P R P V P K Y E G E Q E V I E V E V P E I E Y K D T
151 Y V E K E V V V D V K E K I I P K V T E V V K E V E V V Q Y E W K Q Q Y Q D V P V Y K Y V P K F D V
201 E L D C P P P I I V P Y P E T R F V K E D P S V V Q P Y C S P L T A C C P G T A T H K V V T T D R L
251 Y V R G T D G A G A S Q P S A P L V T V G G A F H S M S P D V Q M Q S E A I P S A H G V L P A D Y V
301 A Q G Q L I G D A P G Y S S E G Q L P N I Q L A P G P V F P M P S P Q V E Q P A S S F G G S S S G S
351 E A E E A K P T A S E K Q S F W N W L F G K K E E T G K V A E Q K K Q E A I D Y T A H F Q Q L H Q Q
401 G T P S H K A E G Q A G K D D T A T N L E P S V V Y K G V V S R P A V F A G N L N P I S F K L H A I
451 E I H Q F I P L P N V R V P E F V K A L P D G L M A T D V S G L D R F F G S V P T G W A D P D V T G
501 I P A P M M S D I L A G N I Q A S T T S P L I N N L S E K F V Q Q E N T V Q Q A Q T D A R A A S G V
551 G L G E E A Q I S A

41

Toxoplasma IMC11 XP_018637647.1

MSGCQQNDWQRRKYILGAHGLSHCRSWKQDFVDSRVLKPSVVGEKC

DRDWVRVLVLQPVDIKTKFVQVPVVKKV
DRFVPKVTYE
EKLVEVPRLIHKTV
EKFV
EKPYIKYV
DKYEEVPETRYMYKYVP
KEVIHERVSYKRKPVTF
DKVTY
KEMPQIKYV
DRYVDVPMFEEVVRYEP

APHIPPLHQDDVSESTIQEHSPGTRQIPTDDQYRVR

1 M S G C Q Q N D W Q R R K Y I L G A H G L S H C R S W K Q D F V D S R V L K P S V V G E K C D R D W

51 V R V L V L Q P V D I K T K F V Q V P V V K K V D R F V P K V T Y E E K L V E V P R L I H K T V E K

101 F V E K P Y I K Y V D K Y E E V P E T R Y M Y K Y V P K E V I H E R V S Y K R K P V T F D K V T Y K

151 E M P Q I K Y V D R Y V D V P M F E E V V R Y E P A P H I P P L H Q D D V S E S T I Q E H S P G T R

201 Q I P T D D Q Y R V R

42

Toxoplasma IMC12 PIM04551.1

Expressed in “mature” cortex and not in budding daughters

MATEFVVPPSMLAVPNAGNVQTGTVGNIVPPSAGTRVVDVPFVHYRQTMCV

KEVKVPVEVTKVAVKKVEVEGVHEVPIIKP
REVNVIQAVRRNVPAPVDVYTIQKYSMPRIQPKYYDVEVPIYVPRYVEVPVPS
HFVTLQ
KEEIPAVVPV

GPPLQGLNSAPVLKPEQAYSLAPAADASDEAAGPVAPGIVEELPLHRQGSG
CSALGYTRTMQNETLTAEVIVQPSGVSYETADVARHAEKEAAVGSLASSPR
VFSRVSEAENDNASERAESVDSMPQ

1 M A T E F V V P P S M L A V P N A G N V Q T G T V G N I V P P S A G T R V V D V P F V H Y R Q T M C
51 V K E V K V P V E V T K V A V K K V E V E G V H E V P I I K P R E V N V I Q A V R R N V P A P V D V
101 Y T I Q K Y S M P R I Q P K Y Y D V E V P I Y V P R Y V E V P V P S H F V T L Q K E E I P A V V P V
151 G P P L Q G L N S A P V L K P E Q A Y S L A P A A D A S D E A A G P V A P G I V E E L P L H R Q G S
201 G C S A L G Y T R T M Q N E T L T A E V I V Q P S G V S Y E T A D V A R H A E K E A A V G S L A S S
251 P R V F S R V S E A E N D N A S E R A E S V D S M P Q

43

Toxoplama IMC13 PIL97952.1

Localized to basal complex (Anderson-White et al. 2011)

METMAQQSGFCQPPGSPVNGAPSNTIYVPLINQTIRRNRIVEIPEIHVV

EKLVPKIQVQDVIRKVPRTDIQWV
EKIVEVPQIQVI
EKIVEVPQVHEIR
REVPRVEVQEIVHRVPRYHVQNV
EKYVEIPQIQYV
EKYVEVPQIQEVVKF
RERVEIVEVPV
EKI
REVPRIEVKVV
EKIRHVPGPIEYIDVPQ
ERII
EKPVYETI
EKIVEVPEVHDVVV
EKPIMVPVP

GPEVEVPVEVPVYYDVPEYYPGPTRVIPVEKEKLYEKLVEVPVHVAQESIRV
VRVPHEVPVDVYKEVPVPRYIDQVVEVPIQVPVPQPPPVIHPVLQNQIIEEEP
IYEQGPPRYIYETPVWAPPVYDDSRPPPIESPSYLPPGGLVPEPPQVGSES
QFLSQKCNGHSSPGFEK

1 M E T M A Q Q S G F C Q P P G S P V N G A P S N T I Y V P L I N Q T I R R N R I V E I P E I H V V E
51 K L V P K I Q V Q D V I R K V P R T D I Q W V E K I V E V P Q I Q V I E K I V E V P Q V H E I R R E
101 V P R V E V Q E I V H R V P R Y H V Q N V E K Y V E I P Q I Q Y V E K Y V E V P Q I Q E V V K F R E
151 R V E I V E V P V E K I R E V P R I E V K V V E K I R H V P G P I E Y I D V P Q E R I I E K P V Y E
201 T I E K I V E V P E V H D V V V E K P I M V P V P G P E V E V P V E V P V Y Y D V P E Y Y P G P T R
251 V I P V E K E K L Y E K L V E V P V H V A Q E S I R V V R V P H E V P V D V Y K E V P V P R Y I D Q
301 V V E V P I Q V P V P Q P P P V I H P V L Q N Q I I E E E P I Y E Q G P P R Y I Y E T P V W A P P V
351 Y D D S R P P P I E S S Y L P P G G L V P E P P Q V G S E S Q F L S Q K C N G H S S P G F E K

44

Toxoplasma IMC14 PIM05297.1

Expressed in “mature” cortex and not in budding daughters

MELCESPCCEPRERLPAIPVLPLATVGGLPASQTLIEEKVTYTTKPIVAEQI
IEVPQVEY

RDHLVE
ERQTVFV
EKVIHVPRTEIQ
ERIVEVPRHVT
REVVVDVPQVQVV
EKFVEIPTPIYQ
EKIIPVARPVIKQKTTHVPKPVLH
EKFVEVPRVYYKPVTV
EKIVEVPEYRIEYKY
RDVPVPQKVFRVVPF
DRIRYVPQIRYV
DKIVKKPEIR

TNSVPVFESTQGRSVKVASPRRVPISGPATPVLMEREQREVAYCDSLMEGL
FGCFMPATPVEKQACRGVSRRRMRLRSPRSPPINMDVVVMSRSPRNRRS
RRVFGDCCSSDVAEVRTPRLMHLPAPTPVAGLAVSSVQHPSLKRPQPVPY
PLPSPPSLPTMVSPCPPPEAMIETKLGAMTFRQFEKLNNEDILGKHLRPYDI
PTSNGQVDSCDTSKCSTLPRPEETTITSNHRQIHPGQLFGEGALSSSAVSP
RGGTRSSDISEVLQRQREELKLRIEHERIHAERLRLACLAEEKRMQLLELEE
QQRQVRLQAHKQQTEYEAQQQLFAREQEHLQKEALAGDVMIQTRYGPMK
FQEFEKLNNEDFMKSPRGLQISTCNTMAYSPSLATPRTQAGETDYRTHTSV
AYVGGTASVPQQVPIYQEAPVILSPRVVVPNRGPVYLQEHQPMYLPRTFQC
SHHGDCPVGGLHPTVGESHDEDLLDERYTIQLQQLTKIAFRNAYQAGLNVY
EGVRSLVNWGKSG

1 M E L C E S P C C E P R E R L P A I P V L P L A T V G G L P A S Q T L I E E K V T Y T T K P I V A E
51 Q I I E V P Q V E Y R D H L V E E R Q T V F V E K V I H V P R T E I Q E R I V E V P R H V T R E V V
101 V D V P Q V Q V V E K F V E I P T P I Y Q E K I I P V A R P V I K Q K T T H V P K P V L H E K F V E
151 V P R V Y Y K P V T V E K I V E V P E Y R I E Y K Y R D V P V P Q K V F R V V P F D R I R Y V P Q I
201 R Y V D K I V K K P E I R T N S V P V F E S T Q G R S V K V A S P R R V P I S G P A T P V L M E R E
251 Q R E V A Y C D S L M E G L F G C F M P A T P V E K Q A C R G V S R R R M R L R S P R S P P I N M D
301 V V V M S R S P R N R R S R R V F G D C C S S D V A E V R T P R L M H L P A P T P V A G L A V S S V
351 Q H P S L K R P Q P V P Y P L P S P P S L P T M V S P C P P P E A M I E T K L G A M T F R Q F E K L
401 N N E D I L G K H L R P Y D I P T S N G Q V D S C D T S K C S T L P R P E E T T I T S N H R Q I H P
451 G Q L F G E G A L S S S A V S P R G G T R S S D I S E V L Q R Q R E E L K L R I E H E R I H A E R L
501 R L A C L A E E K R M Q L L E L E E Q Q R Q V R L Q A H K Q Q T E Y E A Q Q Q L F A R E Q E H L Q K
551 E A L A G D V M I Q T R Y G P M K F Q E F E K L N N E D F M K S P R G L Q I S T C N T M A Y S P S L
601 A T P R T Q A G E T D Y R T H T S V A Y V G G T A S V P Q Q V P I Y Q E A P V I L S P R V V V P N R
651 G P V Y L Q E H Q P M Y L P R T F Q C S H H G D C P V G G L H P T V G E S H D E D L L D E R Y T I Q
701 L Q Q L T K I A F R N A Y Q A G L N V Y E G V R S L V N W G K S G

45

Toxoplasma IMC15 PIL96377.1

Enriched in apical cap and basal complex. High expression levels. Distinctive expression patterns (Anderson-White et al. 2011)

MRICLPPVPQGREAVPDASANVPRQAFSERFRQCEHERLPSGVPRPQTPRCSG
TILIEPAPTGTNNRQQAPTGRCMDMSGSCASVAESAAACCLWQTSPRRWRPTP
REPKMCFFEEPSQSLRFRTPSPRFSCAETTTRMCTTPCSSTDDRCTSRRSIYGT
PQIQTFSASSAGTQAYIRPLGQSSRSVSPWQSSLAPASSAVSSTETTHGIGIYAT
QSQQASHVRVTGGVDSILGPSAPAPTVSSPSHIPAFSQSSCGSTNSAPGNGQS
TWCQTSPVTVGGQASASAQVKIAQAPVAASSQPNYSRAKTTQAVVEQKMVPEGY

DRVEHMVPRLRPVSVVENRIEIPVVKVVDTIVHKQKIE
ERIRFV
DKPVVQEV
EKFVEVPEVIYSDVIVEVPEIVEVIKHVP
KEEI
RENITYVPRFETKIIPKYVDVPIIKIV
DRYEEVHEIHEVLKPVAKVKVVDVP
KEFTRIVPKYSVHKTKSKVSVPDIQY
KEVPRYRMRYEPKIL
RDTVINHIPQYLDIEVPYEVPKVTVVDQPFFVTTY
RDHQYPVPVSHSVTPVLLPGDHTQVVDVPV
EKPYVVVH
DKFTPRPPIPELIP

GQAVLRGVTHLDKERLTAEQQEQLARQTTPSRPTCPDHRFWEREREAELRAPLSPVHI
RPPRQRKFFAGMPRQSCGPMPSSVDAQCCQSPWTQHVQQTSTLSYPN

1 M R I C L P P V P Q G R E A V P D A S A N V P R Q A F S E R F R Q C E H E R L P S G V P R P Q T P R
51 C S G T I L I E P A P T G T N N R Q Q A P T G R C M D M S G S C A S V A E S A A A C C L W Q T S P R
101 R W R P T P R E P K M C F F E E P S Q S L R F R T P S P R F S C A E T T T R M C T T P C S S T D D R
151 C T S R R S I Y G T P Q I Q T F S A S S A G T Q A Y I R P L G Q S S R S V S P W Q S S L A P A S S A
201 V S S T E T T H G I G I Y A T Q S Q Q A S H V R V T G G V D S I L G P S A P A P T V S S P S H I P A
251 F S Q S S C G S T N S A P G N G Q S T W C Q T S P V T V G G Q A S A S A Q V K I A Q A P V A A S S Q
301 P N Y S R A K T T Q A V V E Q K M V P E G Y D R V E H M V P R L R P V S V V E N R I E I P V V K V V
351 D T I V H K Q K I E E R I R F V D K P V V Q E V E K F V E V P E V I Y S D V I V E V P E I V E V I K
401 H V P K E E I R E N I T Y V P R F T K I I P K Y V D V P I I K I V D R Y E E V H E I H E V L K P V A
451 K V K V V D V P K E F T R I V P K Y S V H K T K S K V S V P D I Q Y K E V P R Y R M R Y E P K I L R
501 D T V I N H I P Q Y L D I E V P Y E V P K V T V V D Q P F F V T T Y R D H Q Y P V P V S H S V T P V
551 L L P G D H T Q V V D V P V E K P Y V V V H D K F T P R P P I P E L I P G Q A V L R G V T H L D K E
601 R L T A E Q Q E Q L A R Q T T P S R P T C P D H R F W E R E R E A E L R A P L S P V H I R P P R Q R
651 K F F A G M P R Q S C G P M P S S V D A Q C C Q S P W T Q H V Q Q T S T L S Y P N
