# Supplementary material for: Epiplasts: Membrane Skeletons and Epiplastin Proteins in Euglenids, Glaucophytes, Cryptophytes, Ciliates, Dinoflagellates, and Apicomplexans
Source: mBio. 2018 Oct 30;9(5):e02020-18. doi: 10.1128/mBio.02020-18 (PMC6212826; doi:10.1128/mBio.02020-18)
Supplement: TEXT S2 [file mbo005184120s2.pdf]

1

Key to Gene Annotations

- **General organization:** The medial acid-base dyads (ABDs) are "parsed" into "strings that initiate with a dyad. N-term domain is denoted the head, C-term the tail.
- **Highlights**: Yellow, tyrosine; Green, cysteine; Pink, VPV; Gray, ABD in predicted head and tail domains.
- **Colored font:** Red, glycine residues in ABD domains; Bold-faced red, last G residue in head and first G residue in tail; Blue, alanine residues in ABD domains; Green, repeated string domains.
- **Underscores:** Acid-base triads or tetrads.
- **Commentary**: Notes on distinctive gene features (e.g. orthologues, localization patterns) in green font at top of some pages. Predicted homology domains (e.g. PDZ, coiled-coil) are given in Supplement Table 2.
- **Secondary structure**: PSIPRED predictions for a subset of proteins. Yellow, amino acids predicted in β-strand; pink, amino acids predicted in α-helix; no highlight, amino acids predicted in disordered (random coil) domain.

2

Chromera >Cvel_1331.t1-p1

MGNEASSAQGGGGTVQTQYMYMQGGSTKREDIMELLSRADHQSVNISPQ
VVMNGELNQ**G**LEDAALTQKEAETKAKQ

KDLDLPLEE
EKPVEAEPEEEEDADLLPV
DKKPL
RDSGPIPPEGEGRILNRWLDYENRRIVG
ERLI
DRYHVQ
RELENTKVDEYVLDIIEQEVEVPEIIV
KERIIHNPRIEFQ
ERRVEVPVVKV
KEEI
REIPRVTTIEEVIEIEEEEVTEHVVKMPKYEY
RERPVFVPGPVEVTVKIVEVPHVEY
REVLAEPELQLVSSGPA
KEVII
EKEVEVPLVEYRKVDVEQVF
ERPVPV
DKVVEVPYPVYQDIWHVTPHFV

GVPFEVKREVPYPVTTAVPYVIPVHQEHGGELEPSMSVGGIFGNWRPSLET
GDPTTTQLAQPPDPRGIPTSLALRPQEMAVKPAYTINVQPEEGQGAGPTSP
PLLASAMAGGTGAAPSPGGDREGGREVQEGVAHPYNQRPRVVEEKSTLA
APKVPEPNQAEVSLWDDLAGFFSGGGEVGEPDEEDEEFNLTF

1 M G N E A S S A Q G G G G T V Q T Q Y M Y M Q G G S T K R E D I M E L L S R A D H Q S V N I S P Q V
51 V M N G E L N Q G L E D A A L T Q K E A E T K A K Q K D L D L P L E E E K P V E A E P E E E E D A D
101 L L P V D K K P L R D S G P I P P E G E G R I L N R W L D Y E N R R I V G E R L I D R Y H V Q R E L
151 E N T K V D E Y V L D I I E Q E V E V P E I I V K E R I I H N P R I E F Q E R R V E V P V V K V K E
201 E I R E I P R V T T I E E V I E I E E E E V T E H V V K M P K Y E Y R E R P V F V P G P V E V T V K
251 I V E V P H V E Y R E V L A E P E L Q L V S S G P A K E V I I E K E V E V P L V E Y R K V D V E Q V
301 F E R P V P V D K V V E V P Y P V Y Q D I W H V T P H F V G V P F E V K R E V P Y P V T T A V P Y V
351 I P V H Q E H G G E L E P S M S V G G I F G N W R P S L E T G D P T T T Q L A Q P P D P R G I P T S
401 L A L R P Q E M A V K P A Y T I N V Q P E E G Q G A G P T S P P L L A S A M A G G T G A A P S P G G
451 D R E G G R E V Q E G V A H P Y N Q R P R V V E E K S T L A A P K V P E P N Q A E V S L W D D L A G
501 F F S G G G E V G E P D E E D E E F N L T F

3

Chromera >Cvel_3694.t1-p1

MARRGYKTLKKR

EKPWRNYSSLYLSEV
KEVEVPQVQYAVKPVQNIVGIPKGQIQQVV
KEVEALRLQKIARPVPV
EKVVQVPQQQVVY
KDVPMEALKY
REMPRAVHNPVQQVRQVP
REMVFPRSIPVPC
ERIVQVDQPVDNHVPFQ
REVPIAAPQIIHSDTPLPAPVRQHPVAGAVPVQPEVIPPTATSYAVATAAWVQ
EREGPQSQPEDG
REEVQVPQKVARPHTDSNNAPVKVIQNRHLEETGAVRVPVQQ

GEGDQVSGLQDFRNPLMELPSLSLKGTLASGKKGVTVFRGIFNGETVAAKRAEV
GGDEEKARHQLQEAKTWRGLTEKMKGSTQISQFAKGLSDSSFVSAIGLDPCFLP
FLSDAFRLRWTERVQTWSNAGLNLLPLLSPALDGTETSSETPHVPIQREIPVAPP
QISHGDTSVRPASVEPEEKRREVPKCL

1 M A R R G Y K T L K K R E K P W R N Y S S L Y L S E V K E V E V P Q V Q Y A V K P V Q N I V G I P K
51 G Q I Q Q V V K E V E A L R L Q K I A R P V P V E K V V Q V P Q Q Q V V Y K D V P M E A L K Y R E M
101 P R A V H N P V Q Q V R Q V P R E M V F P R S I P V P C E R I V Q V D Q P V D N H V P F Q R E V P I
151 A A P Q I I H S D T P L P A P V R Q H P V A G A V P V Q P E V I P P T A T S Y A V A T A A W V Q E R
201 E G P Q S Q P E D G R E E V Q V P Q K V A R P H T D S N N A P V K V I Q N R H L E E T G A V R V P V
251 Q Q G E G D Q V S G L Q D F R N P L M E L P S L S L K G T L A S G K K G V T V F R G I F N G E T V A
301 A K R A E V G G D E E K A R H Q L Q E A K T W R G L T E K M K G S T Q I S Q F A K G L S D S S F V S
351 A I G L D P C F L P F L S D A F R L R W T E R V Q T W S N A G L N L L P L L S P A L D G T E T S S E
401 T P H V P I Q R E I P V A P P Q I S H G D T S V R P A S V E P E E K R R E V P K C L

4

Chromera >Cvel_7120.t1-p1

MLFCCCDAPSRDKEAASELWIVAEDKDAYARDLPTGQPALGASTFPFSALP
AFNPLEGLE**G**RTLSVPLPK

KEPPNFPS
DRVVQSKVDLSFPQDGSAQVKCVQVIAVK
KEVPQKI
ERPVPV
EKVIEVPS
EKKVEEESEEEGEEGDVEVV
ERHVEVPQIHII
DRFVEVVQVQEMV
REVPSYVL
KDDVLRQEIEDAQRRVDTLQKQCDALWAASGISLSSV
ERSWETEEVEVLVPVPEFVDVPVEVIKKVQQKVETAVAQKKQTKPKPPKT
ATAVPGQKRVV
EKIVHVPKPVYQ
ERITEVPRIEIV
EKIVEVV

TPQATPEEVERLENLLKRVSALEAEIEALR**G**RSMPRGATLMT

5

Chromera >Cvel_8894.t1-p1

MSSSPNYTRRSESGDGSISCGGTPGQIRRV

EKIVHVPKRYIQ
EKIRYIPKIITQ
EREVRVPKIITRTKIVEVPHVQYI
DKPV
EKIVEVPQVHIRRV
EKVVKVPVRITRHVPIVPHPQVVHFLQALPQVGRCTGFPPPFPATSPHPVP
EREK

KKKGKATEEEQLTRNEASPLSQPATDGLTTFQKGGAEDLLAISPASFVSISAL
SGPKLSPDLSHATPQAKAQLEPVTAKCTTAPLASSLPPAIPVSPESNRFSTR
QASVSAQEVKRSRSREPPISVSHSSASASGSQAIKELLPSVLTPPTSPLKAS
GLPSTGKGGH

6

Chromera >Cvel_8942.t1-p1

MGTPQGPLPLTPPMGPHDNLIVTGTAMPIHTGQTPTAAGMYSSMPPAFPPQ
GAIMQAGSPLRGQLSPGATSIHGP**G**EALSASLRSVAHRIPSKFTAEPQLE

ERVIRMPVVSYE
DREIPE
ERMEY
REKIV
EKLQRVTH
EKVVHVPKVVVQ
EKLIEIPKPEFVEEVVEVPEIRYVQKFVEVPTPVYQ
EKVVHVPKTEVQW
REVLRPKVEVV
EKTVEVPRYEY
REVFV
EKIVEAPVVQHQVVF
KDIPVPQKVHRQ

LDR**G**MPMPPPVPMPPPTATIVATPPPRPIEGPFPPLKTIAPPPPHQFILPPQQLQ
PLQVPFPPPGPKVFAESPVPSPGMMAIAAPPRGLPPPEAHMLPPPTMPTPVPP
GSPPRVPGSPIPMPYPPEVTAGGEATPIPPMQFPMGQPMPGPMPMPGPMTM
PGPMPMPGPMTMPPPMSMPGPMLMPPPMSMPGPMPPPMSVTGPMPMPPP
AAFMGPPPPSAQTPLPPPSPPAPGSPQLPPQQPFAPPPMMMMPPPTIATMKT
LPFPPPPPPPAAPLMVGPPPTAQAPPPMPPMPLQGTGSPVPPPRGITPPPXXX
XXXXXXXRGITPPPTPPAVQTMAPPPQGAPLMMPPPGPPLHMTAPMLMRPQS
FQPLPVGPPPPQHPPMPPPGQVSRAAPPPPASPMMTPPLSPPRTFMHTMPPP
QPPIVDEGPVIDIPVPVYKDVPVKVKVPRKVMRPVPVAPPHQVADPLIETAKGW
VSLEEFQRMNPSMTMHFDHVPPSAEATPAMQGIAGPGPEGFPSIAP

7

Chromera >Cvel_10398.t1-p1

MQLALRFFAFFIVIPEGAGFHLVARPHSLVSPTRLEAVGGNGPPLPSSPEPLS
TASEGRWKGTPELGFSFHERTRTCLNIFVKQRPTCSVLVLYGKKQVGKRRLA

KEAV
EKWR
KDGRLVVEVDL
EKV
KEEDLNV
DRLPEIFWTLWKAADFDLLAYQRLENELDVMRQLWQLMVPDFE
EKYSKA
KERDREVRVFV
EKEL

GGRHQLTVEEAFGVIGLVIRKFAPVFEETAKRETDEENVAARVCAALVDVLEEKA
VAESDTEEAPVLVIRGVERVVLNSAVKKVFDALCWRLVYRQDMDENVPVIMDLS
GEPWDERFFLKVKQKRPNSF

8

Chromera >Cvel_16344.t1-p1

MASPQPTETPMQGPPPPGTTSFNPPQGAFGLPMMPPPAGFPMGPGVPFH
TMSGGPMPMPPPMLAATNASLPMGPPLPPPPMMATSPPPGPPLSMTQQ

EKTEVYRVPV
DKTIQR
RDRVIEYPQIHV
REKVQPTVKVQEVI
KEIPKIEVEWQ
EKVVEVPQVHV
REKYIEVPQVHVVKKHVPKIEYQ
EKIVEIPKIEVQWQ
EKIVEVPHVEV
REKYTDVVQQQEVI
REIPKVEVIEVPI
EKIKHVPKVLIQTI
EKIRHVP

GPIEYIDVEVTRALPPPQPVLMKTLPPPPPPPPPMMVKEVAPPPVMAPLPPPM
IFQKPLPPPPSPPMKLMAPPPMPPPPLMKTVEMPPPSPQPMPLMVKPLPPP

9

Chromera >Cvel_20948.t1-p1

MEPGTGMLPNKFVTAPGHVE

ERLVRVPQVQYE
DREVPGE
ERIEV
RERIV
EKIQKVVQ
EKVVYVPKPVIE
ERVVEVPKPEFIEEIIEVPQIQYTT
REVEVWTPQWM
EKVKYVPKVEVQW
REVPRPRLMVS
EKTVEVPRQEIRHVYV
EKEVEVPVVQHQYVY
KDVPVPQRVHR

TTASMPELPPMPPMLPPPPQQVLVKTLPPPPMPPPTTVLPGPVRELPPPPMP
MPPPTMMAPPPAKIETEIPPPMMAFPPPQPMPMMMAPPPPGPAPLLSTLGG
NPNPLSTSQAPPLPMLAPPLLNKSQLVVGEPRALPSPLPAPEGPPATVSYMP
PPPMSTFAPPPGPPMPLMASLPPPSMPPPPMLEKPPMPPMIMTGPPMEPPK
MPMPTSPPPPMMMPPPPVPPPVPMPMPMPPPSMVMTGPPAEPPKMPPPM
PVSPPPPIMMPPPPMPPMPTMSPMPPPEFEK

1 M E P G T G M L P N K F V T A P G H V E E R L V R V P Q V Q Y E D R E V P G E E R I E V R E R I V E
51 K I Q K V V Q E K V V Y V P K P V I E E R V V E V P K P E F I E E I I E V P Q I Q Y T T R E V E V W
101 T P Q W M E K V K Y V P K V E V Q W R E V P R P R L M V S E K T V E V P R Q E I R H V Y V E K E V E
151 V P V V Q H Q Y V Y K D V P V P Q R V H R T T A S M P E L P P M P P M L P P P P Q Q V L V K T L P P
201 P P M P P P T T V L P G P V R E L P P P P M P M P P P T M M A P P P A K I E T E I P P P M M A F P P
251 P Q P M P M M M A P P P P G P A P L L S T L G G N P N P L S T S Q A P P L P M L A P P L L N K S Q L
301 V V G E P R A L P S P L P A P E G P P A T V S Y M P P P P M S T F A P P P G P P M P L M A S L P P P
351 S M P P P P M L E K P P M P P M I M T G P P M E P P K M P M P T S P P P P M M M P P P P V P P P V P
401 M P M P M P P P S M V M T G P P A E P P K M P P P M P V S P P P P I M M P P P P M P P M P T M S P M
451 P P P E F E K

10

Chromera >Cvel_29922.t1-p1

MPSPLPMKFVTAP**G**SIE

ERLIRVPNVVYE
DREIPQ
ERIEY
REKVV
ERV
EKIV
REKVKYVPKQIIE
ERV
REVPKVEIVEEIVEVPEMRFV
ERVVEVPQIQWM
EKV
G

11

Chromera >Cvel_1899.t1-p1

Articulin

MPGPANAMVSSANLAASGMMQS**G**YIQHDQPIIQ

EKVVEVARPVYQ
ERIVEVPQLVQSV
KDVPVDQVVTQEVVKHVPKIVTQ
ERTVHVPKPVVQ
EKVVEVPQVQYV
EKIVNVPKKYVQ
EKIVHVPKVVTQ
ERTVHVPKVVTQV
KEVEVPQVQYVVKPV
EKVVEVPQVQIQHVV
KEVQVPQKIARPVPV
EKVVEVPQQQVVY
KDVPVEVVQY
REVPRAVQVPVQQVRQVP
REVVVPRHIPVP
RERIVQVDQPVVKHVPIQ
REVPVAVPQIKHVETPVPVPVMQQPV

AVAQPVAVQPQVMPTMATSYAVPTAAY**G**STAAYGYGYGYPTAAYGGYGT
YGYPAATSATPTPAK

12

Chromera >Cvel_9819.t2-p1

Articulin

MAALTEEQRQHFFNKFRATQPQTAHPDCQPHAHYPYYRFHGPLLPIPPFPRH
CLSSAAVEGPQVQRV

EKIVYVPKRYVL
ERTVYVPKIVTQV
KEIEVPQVQYVVKPV
EKIVEVQQVQIQHVV
KEVQVPVKIARPVPV
EKIVEVPQQQVVY
RDVPVEVLQY
REVPRTVHIPNQQVRQVP
REVFVSRFIPAPR
ERIVQVYQPGVKQVPIR
REVPV

AAPHVKHVETPVPAHVMQQPVAVAVHPPLTMRPPGPPMMTPNGWTDSRTQM
PRKMKHSYESAIPSLPLPAQGSSAKLPSESVNVMADVFTPLIDKSSPDGALDEL
PPLEAFTDFLSVDEGGTTKRSLERWLDSREATVAAAEFSASLAEKESPPTLASL
LLDQAFPDNAQRLEKIQSLVSALPVTEGERVRAEMFVLLIERLEAIKPISSDDGDQ
RRRSVSIKKTEGVGTLLGALVGESAASAVRRRLKEAIGGPHLYLCVPLALSFAKK
VGVQGLSSEDVVFLLSLLRGLGNFPCLAGRVGIPVNLFFKNNPSIVFLEKESQRE
ALQTFLSSKIVKGTPDFRAPPASAPKAPPPYAPSPVVPAVPTKESLAQKWLSWS
IPPADLPFLLYVVSSLAESLERLASPQQLHPNAGMRAIFAKMLRRNISLMEDFLTK
AKEEGRRPEVPCDSEIGMLLKCLEREWKSKASTADNDLSESASTASGSAAEHP

13

Chromera >Cvel_10009.t1-p1

Articulin

MDPSSVSPVFRAAGGRQAPTPVFQSLPSHQVPPAPTGPPPQQIRMSSNLV
SMPQMQDPNRSAPLTPRQRLLAQQQHPQAVSASMPFYQTATAFPNQPT
PAALYGSNPMVMQSQQGQQTPHFPSQTYQPTGNVMQQQYGSGTRLVPA
AAALTLQQQVGAQSMSFGSAAYPQYSQYGVFPQTATPVSSHVVPQAPTA
EKDKAPTPPQGVPSPSASAPIQVADPKPGDPIAIKPPPVSASVGPVPESGKE
ETDAKKKEKEAPAPTQARGVAASAPLRTIAVPTRPMGVTASAPLPTTMPAA
SFQGGAAAAAGGDAGGPSEEPPPPPSVVLPGYSVGYSMHERMKGLPQGP
GASKWSSVSKQVDQYRHEVQYVV

KEVRKPVIVYQ
DRLVDIPVVKVI
DRLVPVHKTETVV
KDVF
KDVTETVT
REVKKVVPRIKTRVEYETEVIYV
DRPIEVPRQKVVV
REVPKVEVVEVPVQRTQTVPMPLE
ERQVIV
EKPVPKKMVVHVPNVIR
KEVEVPF
ERVI
EKPVPVVHQRFHEY
ERLIQVPKPII
KDVPVPYRIED

AAAERLPVFADDYWTHMHPASPEANRIEVVPDMLPEGGGEGGVPVSVYPEA
PAGGGQRLEVQTPN

1 M D P S S V S P V F R A A G G R Q A P T P V F Q S L P S H Q V P P A P T G P P P Q Q I R M S S N L V
51 S M P Q M Q D P N R S A P L T P R Q R L L A Q Q Q Q H P Q A V S A S M P F Y Q T A T A F P N Q P T P
101 A A L Y G S N P M V M Q S Q Q G Q Q T P H F P S Q T Y Q P T G N V M Q Q Q Y G S G T R L V P A A A A
151 L T L Q Q Q V G A Q S M S F G S A A Y P Q Y S Q Y G V F P Q T A T P V S S H V V P Q A P T A E K D K
201 A P T P P Q G V P S P S A S A P I Q V A D P K P G D P I A I K P P P V S A S V G P V P E S G K E E T
251 D A K K K E K E A P A P T Q A R G V A A S A P L R T I A V P T R P M G V T A S A P L P T T M P A A S
301 F Q G G A A A A A G G D A G G P S E E P P P P P S V V L P G Y S V G Y S M H E R M K G L P Q G P G A
351 S K W S S V S K Q V D Q Y R H E V Q Y V V K E V R K P V I V Y Q D R L V D I P V V K V I D R L V P V
401 H K T E T V V K D V F K D V T E T V T R E V K K V V P R I K T R V E Y E T E V I Y V D R P I E V P R
451 Q K V V V R E V P K V E V V E V P V Q R T Q T V P M P L E E R Q V I V E K P V P K K M V V H V P N V
501 I R K E V E V P F E R V I E K P V P V V H Q R F H E Y E R L I Q V P K P I I K D V P V P Y R I E D A
551 A A E R L P V F A D D Y W T H M H P A S P E A N R I E V V P D M L P E G G G E G G V P V S V Y P E A
601 P A G G G Q R L E V Q T P N

14

Chromera >Cvel_13540.t1-p1

First half of sequence

Articulin

MQNSKPRLPTRAPAAKAKAAPKAATTTPTSTSSRVSAPAGARTQSRRFSAD
STGSAASRATPQTKANLSTKGRTGQINGSTATLNLHPSMGASSPSGRRRDS
MESHTSASPKTPGVRAAASTGASSLSGRRRESMDSQSSASPKTPAVRKLAL
KRGETNHSEATNTLGMSTHYDSHERPEPPLSESFSACAPGPSLTTPRGGN
GEMEKLKREVKERGEAIERLEQEFEHLEADKRAAQAKADAKEKELEQKNVEM
KEMEERFHEELSKVKEQHRKALGDERRSMTDLKRKGANLSMSDVSSGLRVA
DPAAAGDALTLLSPRTEMARNEREYMTTRAENELLKQQIEAMKKREDEYKKQ
INDLQSHLHEKTVNRMTTHNRRGSTVSQGSLFCGRAYGGLHIPRAPSVVSQT
SQPIAAMLSQPPSQEPTVLRKPRGRGSGAKKSVTIASEEPRSSLASPPMRPL
GVSLQNPRISALGDELAQAAEDLSDHSGSSYPDPQQSEAPSRSVSRAVSRGR
SELEDDQKSLLPPPKPLTRVSLSMHSVPETQSELATSMAPSGQQVLKMAKSLL
SEAKSLISSRAESLMESLHEQPSQPLSSPEEEPSPVGSRRTSDGAPHVPPEA
VPVAIARVSSRLSESLLDVSREQELNRTKKLYDRMNNIISHLEEVEEEQEREKER
QTVEENELNGSFVSEYPTFGLRRLSRKSSWYPGMKIDLAAPPLVTLKHIQTVV

ERQVEVPV
EKIV
EKTVHVPV
EKIV
EKPVHVPV
EKIV
EKTV
EKIV
EKIV
EKPVHVPV
EKIV
EKTV
EKIV
EKIV
EKPVHVPV
EKIV
EKPVETIV
EKIVHVPVTV
EKIV
EKPVETIV
EKIVHVPVTV
EKIV
EKPVETIV
EKIVHVPVPV
[illegible]

15

Chromera >Cvel_13540.t1-p1

Second half of sequence

Articulin

EKPVETIV
EKIVHVPVTV
EKIV
EKPVETIV
EKIVHVPVPV
EKIV
EKPVETIV
EKIVHVPVPV
EKIV
EKPVETIV
EKIVYVPVPKIEYVH
RDIPVPEIV
DRILQYL
DRDVPVV
KEREVVHEIVEV
EKEVPVPVDNYV

AVPLPLVIPAPTMSPFELNSKKLDRLPSKDLSLMMAWLSRFKEAETTPCSIEGLE
HLFTFADMYEFLTGKRISRRRLFFDYLLSQRRWGVEDAQKALYDRNCAWLLEAK
QRKAVEIVPNLDAFSHHPEGHCGSPEMFLQLVCDVGAVEVHTNEDWPKVEEVL
SNVAATRKRLLCDATTSTEGDAVRAAFATGGLIDLISTALSGDETQAHERKEAKET
AARMEAKTEVLPPLATDDESGKLSRQLIMQAKVLGSPFIAALEKGPQEGWVSIRG

16

Vitrella >CEL92206.1

MSGPAPSGSPLAAAFAQAASPPAGQPVSVRDALGQQQQGGEGEMTQSQL
VRLGRTTTPAGQVQT**G**TRRVEVEVPVYSDVHYEDVVA

ERQVPVYTQKIVPKPEVHEVVRQVPELSHSWI
ERKVEVPVIQQV
DRVVEVPQVQVVNKYVPKVEVKTI
ERVVPKTVVKTV
ERIQEVPVVEY
KDKVVEEFLQR

AISEAMQHYRPTVRI**G**EMEVDLRDQVMATRGGGASLRVLGNLAAVASRKVA
VAESFIDGL

17

Vitrella >CEL92508.1

MGAGSSTTVDYDGQVYEISRGAHIRHHEDLSVLQQSAGGDRYRIAERLIEEG
YKDGISTDREAYRPEAQQQQKIQAGTRLIKEMQLQQQQQRIHIRQPPAICRL
CPYQKEIMAFYQKEGEMADIEDHIICGICSKPIRISKEGEVTVPCYLPEYK
EKEEREEAGFEYGKVLSRRVDYESRTVVADNFIDSYRVQRMGDDGKVVDEF
LLEM

REHVVEVPEIITM
EKFVQIPKLEIQ
EREVVVPVRHF
KEKIVEVPQVQIV
EKIVEVPEYIG
EREEVIVIPEVETV
EKVMAIPGPTEI
RERIVEVPHIEY
REVPV
ERVEYQVETVYE
EKV
REVKRTEIQYKEVLV
EKIVEVPHVQYET
KDVYV
EKPIPKYEVKPVYVEVPFVVKRQVPV
ERVVEVPYEVLTV
KDKMVEPQM

SGIDWRSVNLPSAYEIEGGAGSVATKTTGARMAFQSTAEQAEKAGIINLTEAGP
AERVGREPAGFVHPEMTHLRPQPGTEIDGGLVVAAVGQSLAGRKEERYVKYSG
SLTSSPPGGRVETSSSSAAVAQPSSAAAAAAAAVPIDREGEASGAGLGVGE

1 M G A G S S T T V D Y D G Q V Y E I S R G A H I R H H E D L S V L Q Q S A G G D R Y R I A E R L I E
51 E G Y K D G I S T D R E A Y R P E A Q Q Q Q K I Q A G T R L I K E M Q L Q Q Q Q Q Q R I H I R Q P P
101 A I C R L C P Y Q K E I M A F Y Q K E G E M A D I E D H I I C G I C S K P I R I S K E G E V T V P C
151 Y L P E Y K E K E E R E E A G F E Y G K V L S R R V D Y E S R T V V A D N F I D S Y R V Q R M G D D
201 G K V V D E F L L E M R E H V V E V P E I I T M E K F V Q I P K L E I Q E R E V V V P V R H F K E K
251 I V E V P Q V Q I V E K I V E V P E Y I G E R E E V I V I P E V E T V E K V M A I P G P T E I R E R
301 I V E V P H I E Y R E V P V E R V E Y Q V E T V Y E E K V R E V K R T E I Q Y K E V L V E K I V E V
351 P H V Q Y E T K D V Y V E K P I P K Y E V K P V Y V E V P F V V K R Q V P V E R V V E V P Y E V L T
401 V K D K M V E P Q M S G I D W R S V N L P S A Y E I E G G A G S V A T K T T G A R M A F Q S T A E Q
451 A E K A G I I N L T E A G P A E R V G R E P A G F V H P E M T H L R P Q P G T E I D G G L V V A A V
501 G Q S L A G R K E E R Y V K Y S G S L T S S P P G G R V E T S S S S A A V A Q P S S A A A A A A A A
551 V P I D R E G E A S G A G L G V G E

18

Vitrella >CEL96659.1

MRELSQSPTESRTEKVLPSMQSQPLPPPQTRCLHTNPGPHAQTAPPSAGE
PHVLHIPVHQEVQVR

DRIVEIPEIHLVEVIKPKVTIQEVI
KEVPKYEPVYT
EKIVEVPQVHTV
DKFVEVPQIQEVI
REVPKFEVQEIVRGIPKVQVQYV
DKPMGVAQVQEVI
KEVPK

ADGVLPVDGATRSVPMGMTKVVEKIRHVPGPVQYVGVPVPKVVHSGKVEIV
EKIKYVEVPSSQIVEVEKPYPVPVPGPVIDVPVPMGSAGLGENRSVLGSKPP
GSGRIIQIPVEVPVEKEIIVHKPIPVPVGHDGKPAAVAKPVEQIRHVEVPEFVD
EYVDVPVPGDAPIIIQPYPMVEVTHLPPIIEKAEPRYVHDPPVHLPPEFTTQQV
PAEAKTTPLNNPPSGLPSEGP

19

Vitrella >CEM00883.1

MASDAPA

RDEVHNLRV
ERKYVV
REVRKPVYEYV
ERVVEVPIVQTV
DRII
ERHVDEEVI
KEVPKLDIQYETKIIPKPTLKVVPRYEYV
KEVVTV
DRPVVVPQVEEVVKSVPKIEVVEVPV
EKVKLVPV
RETKYV
EKIVEVPHTLVI
EKPIQKIV

TVPKVETKIVENVVEVPKVYLHELSQIVQVPKPVYEDELVDVLVEKAVPKFVEHNVS

20

Vitrella >CEL92204.1

Articulin.
92204, 98590, 12952, and 15816 are orthologous

MSGPAPPGSPLAAAFAQAASPPAGQPVSVRDALGQQQQGGEGEMTQSQLV
RLGRTTTPAGQVQTGTRRVEVEVPVYSDVHYEDVVA

ERQVPVYTQKIVPKPEVHEVVRQVPELSHSWI
ERKVEVPVVQHV
DRVVEVPQVQVVNKYVPKVEVKTI
ERVVPKTVVKTV
ERIQEVPVVEY
KDKVVEVPHVVEVV
KEVPKAVPIDIHVPVIKYVPKVEVKTI
ERVQEVPVPHYVDVPVEVV
KEVPKYIV
KEEVRHVPVPGEVVPIW
KEVVV
EKEVVV
DREVEVVKY
KDI
EKPYEVPVYEYKYNKYPVVIPHR

TTTMLPPSPTHVVVPPIIKATLNIDIPPGVSIS

21

Vitrella >CEL98590.1

Articulin.
92204, 98590, 12952, and 15816 are orthologous

MSSPAPSGSPLAAAFAQASPSAGQPVSVRDALGQGQQGQQGGEGDLTYS
QLVRLGRTTAPAGQVQTGTRRVEVEVPVYSDVHYEDVVA

ERQVPVYTQKIVPKQEVHEVVKYVPELSHSWI
ERKVEVPVVQHV
DRVVEVPQVQVVNKYVPKVEVKTI
ERVVPKTVVKTV
ERVQEVPVVEY
KDKVVEVPHVVEVV
KEVPRAVPVDVRVPVIKYVPKVEVKTI
ERV
EKMPVPQYVDVPVQVV
KEVPKYIVQEQF
RDVPVPGPVVPVW
KEVVV
EREEVI
DREVPYITY
KDI
ERPYEVPVYEYKYNKYPV

TLPHRTTKMLPPGPTNFVVVPVIKCNVTIDIPPGFPITHAQLVELFAARGVDLNQ
FKMLSEPIPLPPGEAPTTIEARVETTGPQYPEGAIIYPGVLPPPHMMRDGQTWC
GPPEKVGELIETILDGPPAGWIPTQEQLEIMRAHKLTPEQCGYKGPAMTPPPPV
MTMTHSVPVLEPSPPVQHSVPVLEPSPPVQPPIQPHGRHIHLPAGGMPFPEAIQ
AAPGMIQCCR

22

Vitrella >CEM12952.1

Articulin.
92204, 98590, 12952, and 15816 are orthologous

MSGPAPSGSSPLAAAFQAASEPVSVRDALAMGGQQQQGGEGEMTQSQLI
RLGRTTPAGSIQTGTQRIEVEVPVYTDVHYGDVVA

ERTVPLHMQKIVPKPEVHEVVRQVPELSHSWI
ERQVEVPVIQHV
ERIVEVPQVQVVNKYVPKVEVKTI
ERVVPKTVVKTV
DRVEEVPVVQY
KDRDKIVEVPHVVEVI
KEVPKPVSVEVQVPVIKYVPKVEVKTI
ERIEHVPVPQYVDVPVPVIKQVPKIITE
ERVVDVEVPGPVIEVW
REVPV
EREEVI
EREVEVVKYVDVPKPYEVPVYDYKHNKYPVEIPH

EKTTTLPPGPTNFVIPSIIKGNFTFDIPPGMRITREELVHYVRATKGIDLNQCKE
IAPPMPLPEGTAPTSIEAHFENEPLPEYVKNAKPWPVDEWGPLPDPSQMQD
GHSWTGPPVKVGEPIKTTLQGPPQGWIPNQHQLKIMAEHKLTPEQCGYQGP
PINMPMLMAPPPPFPGAGRPVAPPPAAPAGPPVVTMMRPQMVPPPPAHMIA
PKKKSSTKKSCGVGFFGWFTGSKK

23

Vitrella >CEM15816.1

Articulin.
92204, 98590, 12952, and 15816 are orthologous

MSGPAPSGSSPLAAAFQAASEPVSVRDALAMGGQQQQGGEGEMTQSQLI
RLGRTTPA**G**SIQTQRIEVEVPVYTDVHYEDVVA

ERTVPLHVQKIVPEPEVHELVRQVPELSHSWI
ERQVEVPVVQHV
ERIVEVPQVQVVNKYVPKVEVKTI
ERVVPTTVVKTV
DRVEEVPVVQY
KDKIVEVPHVVEVI
KEVPKPVPVEVQVPVIKYVPKVEVKTI
ERIEHVPVPQYVDVPVTVI
KEVPKIITE
ERVVDVEVPGPVIEVW
REVPV
EREEVI
EREVEVVKYVDVPKPYEVPVYDYKHNKYPVVIPH

EKTTMLPL**G**PTNFVIPSIIKGNFTFDIPPGMRITREELVHYVRATKGIDLNQCKEI
APPMPLPEGAAPTSIEAHFESEPLPEYVKNAKPWPVDELGPLPHPSQMQDGH
TWTGPPVKVGEPIKTTLERPPQGWIPNQHQLKIMAEHKLTPEQCGYQGPPIRM
PMVMAPPPPFPGAGRPFAPPPAAPAGPPMVMMMRPPMMAPPPAHMIAPKKK
ISTKKSCGVGFFGWFTGSKK

24

Vitrella >CEM15033.1

Articulin

MYGGPPPPSVPPGVPPYPGAMPPGAVPYGFPPGAMPGPPGAMPPGAAP
PFGAPGAPGAPGYPGGLPAKEGSVFRPTPHQLGQSYKE**G**

DRIVQQRIVGEVLRPVPVVTEVEVPQVTYQV
KEQPVSHMIVH
EKVVPVPKIELQ
ERVKHVPKYMTE
ERLIEVPQYKYE
EKLIEQVEEVTQENVVYVPKPVVE
EREVHVPKPVYEHKLVEVPHYALQMVPY
EKRIEVPQVYTQVLTKKVDVPYRV
ERQVPV
EKLVEVPQLAVSIT
ERQVPVPQY
KEM
EKTIEVPKHVY
RDKPV
EKIV
EREVPVPHEVKVQ
KEVDV

ADPIHTQYEQQMKAAEFRYFIDYAQIPSVV**G**PQGEIGQVYYHHVPQLQLGY
TVRGELATTGPSHLFYPSQAQAVIPPGGGAPVVMPSGPLPGVTMGGVPAV
PPQMVGAVQGMYPLGGPYVPSPGAPMAPYGLPGQQQGAEGAAGPAPVM

25

Vitrella >CEM30227.1

Articulin

…VPHVVEVV

KEVPKAVPIDIHVPVIKYVPKVEVKTI
ERVQEVPVPHYVDVPVEVV
KEVPKYIV
KEEVRHVPVPGEVVPIW
KEVVV
EKEVVV
DREVEVVKY
KDI
EKPYEVPVYEYKYNKYPVVIPHR

TTTMLPPSPTHVVVPPIIKATLNIDIPPGVSISQMQLVELFATNGIDLNLFKYIQEPIP
AAFPEQLKDAKPWPTETFGPLPPTTHMRDGHTWTGKPEPVGDVIETKLEGPPA
GWIPNQEQINIMNEHKLTPEQCGYTGPQLVATAVPVPAQPVQRPPVPARACCTS
RCR

26

Vitrella >CEM38470.1

Articulin

MSGPAPSGSPLAAAFQAASEPVSMRDALAMGGQQQQGGEGEMTQSQLI
RLGRITPAGSIQTGTQRIEVEVPVYTDVHYEDVVA

ERTVPLHVQKIVPKPEVHEVPQVQVVNKYVPKVEVKTI
ERVVPKTVVKTV
DRVEEVPVVQY
KDKFVEVPHVVEVI
KEVPKPVPVEVQVPVIKYVPKVDVKTI
ERIEHVPVPQYVDVPVPVI
KEVPKIITE
ERVVDVEVPGPVIEVW
REVPV
EREEVI
EREVEVVKYVDVPKPYEVPVYDYKHNKYPVVIPH

EKTTMLPPGLTNFVIPSIIKGNFTFDIPPGMRITREELVHYVRATKGIDLNQCKE
LAPPMPLPEGAAPTSIEAHFENEPLPEYVKNAKPWPVDEWGPLPHPSQMQD
GHSWTGPPVKVGEPLKTTLEGPPQGWIPNQHQLRIMSEHKLTPEQCGYQGP
PINMPMVMAPPPPFPGAGRPVAPPPAAPAGPPMVMMMRPPVVPPPPAHMIA
PKKKSSTKKSCGVGFFGWFTGSKK
