# Supplementary material for: Epiplasts: Membrane Skeletons and Epiplastin Proteins in Euglenids, Glaucophytes, Cryptophytes, Ciliates, Dinoflagellates, and Apicomplexans
Source: mBio. 2018 Oct 30;9(5):e02020-18. doi: 10.1128/mBio.02020-18 (PMC6212826; doi:10.1128/mBio.02020-18)

1

Key to Gene Annotations

- **General organization:** The medial acid-base dyads (ABDs) are “parsed” into “strings that initiate with a dyad. N-term domain is denoted the head, C-term the tail.
- **Highlights**: Yellow, tyrosine; Green, cysteine; Pink, VPV; Gray, ABD in predicted head and tail domains.
- **Colored font:** Red, glycine residues in ABD domains; Bold-faced red, last G residue in head and first G residue in tail; Blue, alanine residues in ABD domains; Green, repeated string domains.
- **Underscores:** Acid-base triads or tetrads.
- **Commentary**: Notes on distinctive gene features (e.g. orthologues, localization patterns) in green font at top of some pages. Predicted homology domains (e.g. PDZ, coiled-coil) are given in Supplement Table 2.
- **Secondary structure**: PSIPRED predictions for a subset of proteins. Yellow, amino acids predicted in β-strand; pink, amino acids predicted in α-helix; no highlight, amino acids predicted in disordered (random coil) domain.

2

Paramecium PTET.51.1.G0190258; XP_001429876.1

Articulin. Orthologue of PTET.51.1.G0130289.

MNNQSSKSVRFNEGHKILQPVNTNTTSTGSSKLTKVTSAIQELRTSFNNQAQ
KLTSSRVSDQLNESVATVTRSPGHSCAPSYCQPCQPMCQPMCQSVCQPQF
GFQQQQMCSPQAYVVQGGSDQRHLEKEIKKLKKKNKKLKESKEEVIVSTIAE
TPKRQRPQQHVQDPEIDTIKREIQEMERIIKQMEQQPKQQSNSEIELYLEDNK
KCLKKMCKKIKSLEKELYEVGRQRDEALIIKQQLERENQEMFDRIGELESLLKV
ADKKVFDLTVQLERQNGYVKQLEDEVERLRKKKKKQIEIQ

ERVV
EKIV
EKPVEVIKTVHVNQPHQVQEVKPVEII
KEVI
KEVPSEPKIV
EKIIEIPKIEYVYQQVPQYIEVPKLQTIEVPVVQRIEVPYEVPYY
RDVPYEVI
KEVPYEVV
REVI
KEVPYEVI
KEVI
KEVPYEVI
KEIPVYIEVPV
DRIV
ERRVEVPV
ERIVEVPV
DRVVEVPVPYEVPYPY
ERVVEVPY
ERIVEVPR
DRYM
DRYI
DRPV
DRYVEVPV
ERRVEVPY
ERIVEVPY
EKIVEVPV
EKIVEVPV
EKIVEVPV
DRFV
ERYV

RDDAELEMLNIENRELQRIIGIWEDRANKLENEIIKERRISDKLRFDIEELEYMVEDGR
SFNNQQQEQFRRYLKELKSKYESKIIEARKGVVIKHHVIPTEVQQTVIQQPISTGISQNL
PQFGGQMLNQ

3

Paramecium PTET.51.1.G0130289

Articulin. Orthologue of PTET.51.1.G0190258
Secondary structure next slide

MNNQSSKSVRFNEGHKVLQPVNTNTTSSGSSKSSKVTSAIQELRTSFNNQAQ
KLTSSRVSDQLNESVATVTRSPGHSCAPSICQPCQPMCQPMYSSVCQPQFIC
QQQPVCPPQAYVVQGGNDQRHLEKEIKKLKKQNKKLKQSKEEIIVSTIAETPK
KHRPQQHVQDPEIDTIKREIQEMERIIRQMEQQPKQQSNSEIELYIEDNKKCLK
KMCKKIKSLEKELYEVGRQ

RDEALIIKQQL
ERENQEMFDRIGELESLLKVA
DKKVFDLTVQL
ERQNGYVKQLEDEV
ERLRKKKKKKQIEIQ
ERVV
EKIV
EKPVEVIKTVHVNQPQQVHDVKPVEII
KEVI
KEVPSEPKIV
ERIVEIPKIEYVYQQVPQYIEVPKVQTVEVPVVQRIEVPYEVPYY
RDVPYEVI
KEVPYEVI
KEVI
KEVPYEVIKQVI
KEVPYEVI
KEVPVYIEVPV
DRIV
EKRVEVPV
ERIVEVPV
DRVVEVPVPYEIPYPY
ERVVEVPY
ERIVEVPY
EKIVEVPR
DRYV
DRYV
DRYM
DRYI
DRPV
DRYVEVPV
EKRVEVPY
EKIVEVPV
EKIVHVPV
EKIVEVPV
EKIVEVPV
DRYV
DRYV
RDESELEMLNIEN
RELQRII

GIWEDRANKLENEVIKERRISDKLRFDIEELEYMVEDGRAFNNQQQEQFRHYLKELKSK
YESKIIEARKGVVIKHHVIPSEVQQTVPTFIQQPISTGISQNLPQFGGQKINQ

4

Paramecium PTET.51.1.G0130289

1 M N N Q S S K S V R F N E G H K V L Q P V N T N T T S S G S S K S S K V T S A I Q E L R T S F N N Q
51 A Q K L T S S R V S D Q L N E S V A T V T R S P G H S C A P S I C Q P C Q P M C Q P M Y S S V C Q P
101 Q F I C Q Q Q P V C P P Q A Y V V Q G G N D Q R H L E K E I K K L K K Q N K K L K Q S K E E I I V S
151 T I A E T P K K H R P Q Q H V Q D P E I D T I K R E I Q E M E R I I R Q M E Q Q P K Q Q S N S E I E
201 L Y I E D N K K C L K K M C K K I K S L E K E L Y E V G R Q R D E A L I I K Q Q L E R E N Q E M F D
251 R I G E L E S L L K V A D K K V F D L T V Q L E R Q N G Y V K Q L E D E V E R L R K K K K K K Q I E
301 I Q E R V V E K I V E K P V E V I K T V H V N Q P Q Q V H D V K P V E I I K E V I K E V P S E P K I
351 V E R I V E I P K I E Y V Y Q Q V P Q Y I E V P K V Q T V E V P V V Q R I E V P Y E V P Y Y R D V P
401 Y E V I K E V P Y E V I K E V I K E V P Y E V I K Q V I K E V P Y E V I K E V P V Y I E V P V D R I
451 V E K R V E V P V E R I V E V P V D R V V E V P V P Y E I P Y P Y E R V V E V P Y E R I V E V P Y E
501 K I V E V P R D R Y V D R Y V D R Y M D R Y I D R P V D R Y V E V P V E K R V E V P Y E K I V E V P
551 V E K I V H V P V E K I V E V P V E K I V E V P V D R Y V D R Y V R D E S E L E M L N I E N R E L Q
601 R I I G I W E D R A N K L E N E V I K E R R I S D K L R F D I E E L E Y M V E D G R A F N N Q Q Q E
651 Q F R H Y L K E L K S K

5

Paramecium PTET.51.1.G0660154

Articulin

MSQIHNDLCIKSIDELRQAFKQVQLPNKQPQQQQQSQQQSPQKEVERVIETT
APTSSHQQIHCHPICFPSHICHPINYQPFIIVQEQPVNERKSNKKKKKYVKEVQ
EEIQVQIPVKKSRQKIEQPNFNVQREVDHLEQLIRQVKIQEQPAQVLEYHISVP
EQEKPKDNTEMIAMLSALKQEPIQLPKDDYLKIENARLQEEIKNTKISYHELSV
KYEELQKRKRK

EKIV
KEIEY
KEIPKIVEVP
RDVV
REVVKPFEVI
KEVI
KEV
EKPSQMNT
KEIPIYVPQY
KEVTVN
KEVPVY
KEVPV
EKEVKVY
KDIPVYKVQPVYYDVPVY
RDVPIYQKVPVY
KEVPVYSEPINVY
REIPII
KEV
DRVPE

TIIEHPNLSRTRRVISPSQN**G**RSYIRSPYSRIEY

6

Paramecium 4223 epiplasmin family1 CAI39404.1

MSQRPPVPQNQAPQPQQFQPAPTYAPQYAPQYAPTYAPAYAPTYAPTYAPQY
APAPIAPLTYSVARPVAPVAPVAPVVAQPVIAQPVVAQPVLQQSVIAQPVVQQS
VHATIK**G**ESRIEYIPYQKAVMEYEEQEVVQYVPR

ERKVTDYYAVEYQTEYVPQVFQ
EKYTEYVPV
DRYQ
ERVEYYPV
ERQVVH

QQVVQQPVVQQVVQQPVVQQVVQQPVVQQVVPQPVVQQVVQQPLSVVQPVQT
VPLTYAPQYAAPIVSSRVIPSYPQYPSYPQYQQAPQQHQAPQQPPRSNLNVH

7 *Paramecium* EPI41 epiplasmin family 1 CAI39386.1

MSQRPPVPQNQAPQQPQAPQYHPQAPQYAPQYAPQYAPQYSPAPLATYPAQY
APATYAPAYAPVAPLTYSVARPVAPVVAQPVVQAPVLQQSVIAQPVVQQPVHAT
IK**G**ESRIEYVPYQKAVMEYEEQEVVQYVP

RERKVTD**YY**AVE**Y**QTE**Y**VPQVFQ
EK**Y**TE**YVPV**
DRYQ
ERVE**YY**PV
ERQVVH

QQVVQQPVVQQVVQQPVVQQVVQQPVVQTVAPQPVVQQFVQQPVSIVQPVQT
YPVQYAAPIVSSRVIPSYPTYPQYPQYHPAPQQVQQQPPRSNLNNNI

1 M S Q R P P V P Q N Q A P Q Q P Q A P Q Y H P Q A P Q Y A P Q Y A P Q Y A P Q Y S P A P L A T Y P A
51 Q Y A P A T Y A P A Y A P V A P L T Y S V A R P V A P V V A Q P V V Q A P V L Q Q S **V I A** Q P **V V** Q
101 Q P V H A T I K G E S R I E Y V P Y Q K A V M E Y E **E Q E V V Q** Y V P R E R K V T D Y Y A V E Y Q T
151 E Y V P Q V F Q E K Y T E Y V P V D R Y Q E R V E Y Y P V E R Q V V H Q Q V V Q Q P **V V Q** Q **V V** Q Q
201 P **V V** Q Q V V Q Q P V V Q T V A P Q P V V Q Q **F V** Q Q P V S I V Q P V Q T Y P V Q Y A A P I V S S R
251 V I P S Y P T Y P Q Y P Q Y H P A P Q Q V Q Q Q P P R S N L N N N I

8

Paramecium EPI2 epiplasmin family 2 CAI28404.1

MSNIPQSQHPQQAQQPPQAPQPYYGQPSYAQPYGAPLSPLRYSYAPPVVQQV
VPQTYVPQQVVPQTYVPQ QVVAQPVVAQPVVAQSVVAQPTIKGESRIEYVPY

EKTVLEYEEVRQRIQVP
REKFVTDYYAVEYQTEYVPQVFQ
EKFTEYVPV
DRYQ
ERVEYYPV
ERQVVH

QQQVQQVVAQPVQQVVTQSVVQPVQYAPQPVQYVQQPVQYAPQPVQYASQPV
QYAPAPLQQTTYVPAPVASLPLAQTQVPTRTVPQARPQQPLDRTQAQNPRPQPA
QQPQQKQKSFLDRLFDRD

9

Paramecium EPI34 epiplasmin family 2 CAI39393.1

MSNHPASTKPPQKTAPAPQQPIPYSPPRTYAPPVAFASPSYLPIQQSFVGAP
VQYVPQPVAVQPVAVQPVAVQPAQQVIKGESRIEYIPY

EKSVIEYEEVRQKIQVPR
EKYITEYQAVEYQTDYIPQVFY
DKVTEYVPV
DRFQ
DRVEYYPV
ERQVVH

QPVQQVVAQPVVQSVVQQVPQYVAPVQSVVQPVYQQPQISYAPYVQPNFAPS
RIAPVSYAPPLSYGAPVSHPRRYVNHIVINQSPPAKPQPAQKPQPQKEKKTFLD
NIFS

1 M S N H P A S T K P P Q K T A P A P Q Q P I P Y S P P R T Y A P P V A F A S P S Y L P I Q Q S F V G
51 A P V Q Y V P Q P V A V Q P V A V Q P V A V Q P A Q Q V I K G E S R I E Y I P Y E K S V I E Y E E V
101 R Q K I Q V P R E K Y I T E Y Q A V E Y Q T D Y I P Q V F Y D K V T E Y V P V D R F Q D R V E Y Y P
151 V E R Q V V H Q P V Q Q V V A Q P V V Q S V V Q Q V P Q Y V A P V Q S V V Q P V Y Q Q P Q I S Y A P
201 Y V Q P N F A P S R I A P V S Y A P P L S Y G A P V S H P R R Y V N H I V I N Q S P P A K P Q P A Q
251 K P Q P Q K E K K T F L D N I F S

10 *Paramecium* EPI38 epiplasmin family 2 CAI39389.1

MSNRPPTQQPGQPATTAPQYQPQWNPTFSPPRAYQAPVQLQSPAYIPQYYTQ
PVAQSYVAPPVVQQPIVYQQPVVTQSVVAQPVVTQPVVQQQPIIKGESRVEYIPY

EKSVIEYEEVRQKIQVP
REKYVTEYQAIEYQTEYVPQVFY
DKVTEYVPV
DRYQ
ERVEYYPV
ERQVVH

QPQIQTVAQPVVQQSYVAQPVVTQSYVAQPVVQQSYVTQTVPVVSQPIQYAQYP
QYAQPATQTIYGPPVPIGNGPIPASQLPAAVKTGQFQQTPQK

1 M S N R P P T Q Q P G Q P A T T A P Q Y Q P Q W N P T F S P P R A Y Q A P V Q L Q S P A Y I P Q Y Y
51 T Q P V A Q S Y V A P P V V Q Q P I V Y Q Q P V V T Q S V V A Q P V V T Q P V V Q Q Q P I I K G E S
101 R V E Y I P Y E K S V I E Y E E V R Q K I Q V P R E K Y V T E Y Q A I E Y Q T E Y V P Q V F Y D K V
151 T E Y V P V D R Y Q E R V E Y Y P V E R Q V V H Q P Q I Q T V A Q P V V Q Q S Y V A Q P V V T Q S Y
201 V A Q P V V Q Q S Y V T Q T V P V V S Q P I Q Y A Q Y P Q Y A Q P A T Q T I Y G P P V P I G N G P I
251 P A S Q L P A A V K T G Q F Q Q T P Q K

11

Paramecium EPI20 epiplasmin family 2 CAI39407.1

MSNVPNSHHPPQAPKQGPPYQPTQFQPGFAPQYAPAPVTYGPPLTSSPLRYS
QPLYQPSVVAQPVYAPPVVQQPVYAPSVVQQPVVTQSVVAQPVVAAQPIKGE
SRIEYVPY

EKTVLEYEEVRQKIQVP
RERYVTDYYAVEYQTEYVPQVFQ
EKYTEYVPV
DRYQ
ERVEYYPV
ERQVVH

QPAQQVQQVVAQPVVQQPVQVVQQPVQYVQQPVVQQPLVQSIPVQTVRPPVYAPG
SLPLGQTVSPRLPPQAQTKPQAQLPQTKQPQQQQKTKSFLDRLFDRD

12

Paramecium EPI18 family2 CAI39409.1

MSNIPPSQHPPQAPQPGPYQQPTFQPGFAPQYAPAPVAYGPPLTQSPLRYSQ
PLYQAPVVQQPVYAQPVVQQPVYTQPVVQQPVYAQPVVQQPVYAQPVVQQP
IVTQSIVAQPQPVVAAQPIQ**G**ESRIEYVPY

EKTVIEYEEVRQKIQVP
KEKYVTDYYAVEYQTEYVPQVFQ
EKFTEYVPVDRYQ
ERVEYYPV
ERQVVH

QQVQPVQQVVAQPVVQQPVQYVQQPVQVVQPQPVQVVQQQPVYQQQPVYQQ
PLVQSIPVQTVRPPVY**G**PATTLPLGQTVSPRPLGTTVPAKPLDKTQGPKQPAQQQ
NKQKSFLDRLFDRD

13

Paramecium EPI43 family 4 GSPATP00017377001

MAEYQPQGIVQQPQLYGTPQTITGQPQFVRPIVLGNSTYQQQLVTNQNKGQS
GFYQGGGVLVNQPVLGASAISSNIVSTGQAVK**G**ESRIEYIPY

EKTIMEYEEVRRQYQVPITRQITEYQAIQYETEYIPQVIQ
EKVIEYMPV
EKFA
ERVEYQ

TVTRQNVLQNTVQQVQQTQIQPIVTTQTYQTTTPIVQTVQQPIVTQVAQPINVPQ
TYYSTYQ

14

Paramecium EPI40 epiplasmin family 4 CAI39387.1

MANLPPQQYSPPSNLPTNNREPQTFQQPTIGQPQVARSNLPIQSLPDTALQYLPQQIAQQ
PYSSQPLQQPLYNQLQPTKLGPQQPIFGQSQIQQIAQPPQEGNVVKGQSRIEYIPYERTI
TEYEEVRRQVQVPITKQVTDYYAVQYDIEYIPQVIQ

EKQIEYVPV
ERVAERTEYYTV
EKQNVIQQPIGYQSQIQTNYIPEQFQYNQII
ERQSALAYQPYQQQTQIQT
REVTQYPV

AQQQQYIQQLPVQQSVPLPAPQAQYLPTQYQQYASPIGIQQPQYQIQQTVPLNYGQQFQA
SQIQPAPKQPIATATVPQGYQIPTQTVPQQYINQQVEQDPTIHSKRPQQQLAQTAQYNPQL
QQTVGPNQFAQSVPQQQSLQQDLGRTRPYQQQQQPQPQQQQLPQQQQGVPQKPNKEK
SFLEKLFD

1 M A N L P P Q Q Y S P P S N L P T N N R E P Q T F Q Q P T I G Q P Q V A R S N L P I Q S L P D T A L [illegible]
51 Q Y L P Q Q I A Q Q P Y S S Q P L Q Q P L Y N Q L Q P T K L G P Q Q P I F G Q S Q I Q Q I A Q P P Q [illegible]
101 E G N V V K G Q S R I E Y I P Y E R T I T E Y E E V R R Q V Q V P I T K Q V T D Y Y A V Q Y D I E Y [illegible]
151 I P Q V I Q E K Q I E Y V P V E R V A E R T E Y Y T V E K Q N V I Q Q P I G Y Q S Q I Q T N Y I P E [illegible]
201 Q F Q Y N Q I I E R Q S A L A Y Q P Y Q Q Q T Q I Q T R E V T Q Y P V A Q Q Q Q Y I Q Q L P V Q Q S [illegible]
251 V P L P A P Q A Q Y L P T Q Y Q Q Y A S P I G I Q Q P Q Y Q I Q Q T V P L N Y G Q Q F Q A S Q I Q P [illegible]
301 A P K Q P I A T A T V P Q G Y Q I P T Q T V P Q Q Y I N Q Q V E Q D P T I H S K R P Q Q Q L A Q T A [illegible]
351 Q Y N P Q L Q Q T V G P N Q F A Q S V P Q Q Q S L Q Q D L G R T R P Y Q Q Q Q Q P Q P Q Q Q Q L P Q [illegible]
401 Q Q Q G V P Q K P N K E K S F L E K L F D

15

Paramecium EPI11 epiplasmin family 4 CAI39415.1

MNNPRPSQLPAQKATPSQLPPTSQQQVPETYQAQPNWVPASSQIQPGVLSQP
IPLQQTGLIQNPQYLQQPVYAQQQLIQSSAPVVTQGNAIK**G**ESRIEYIPYT

KEVTEYVTQEVVEYVP
RERKITDYYAVEYVTEHIPQVIQ
EKYIEYVPVETI
KERTEYQAVTKQSVVQAPIDY

QQIQKTQQYQVAAPIQYAQTYTQPIQYAQTTVQPIQYAQTTTQFVPTTQSQYVSAP
VTTSQFVSQPYPTTTY**G**APITNYGQLTQNTAYLPAATYAQPGIGQVTTTTQQYSTG
WQQVYPASTGNFQPLQQQQVGQLQIQGQSPKYA

16

Paramecium EPI51 epiplasmin family 5
GSPATP00033927001

MSYYKPFYQSAYDRTAPVYPPSQYGPAYYGPYDRPYSYQSRAPTRBEQWSEY
IPVEQRYTDYVPETKVEYRPV

EKSYTDYIEVKHETDYVPVPRL
EKRVEYIPV
DRYDEHVDYVPVQ

NSHVVKGPQSRAGYGYQSQYLPPPPPAPTSYSNYRYSPSRVSGYRPGATFYGY
RYL

17

Paramecium EPI48 epiplasmin family 5
GSPATG00018713001

MSYLUSPURSYARPYQYSPTRSYVSSPYYTSYPPRGNSWV

ERIPVEQRYTEYVPEQRIEY
KPV
ERRYTDYVEIEHY
RDYVPVPRL
ERRVEYVPI
ERYDEAVDYVPV
ERSSVVRQPL

SNSLAYSRYSRYPSRYYY

18

Pseudocohnilembus persalinus KRX06821.1

Articulin. First half of sequence

MKKNTKHKQNYRKNSGSSMEVPVEIPYQVEI

EKIV
EKIVQKPVVQ
EKIV
EKIVEVPVEIPYQVEI
EKIV
EKPVIQ
EKIV
EKVVEVPVEVPYYVEV
EKIV
EKIV
EKPVIQ
EKII
EKIV
EKVVEVPVEVPYEVQV
EKIK
EKPVIQ
EKIV
EKVVEVPVEV
EKIV
EKPVIQ
EKII
EKIV
EKVVEVPVEVPYEVEV
EKII
EKIV
EKPVVQ
EKIVKQIV
EKVVEVPVEIQI
EKIV
EKPVIQ
EKIV
EKIVEIPVEVPYQVEI
EKIV
EKPVVQ
ERIV
EKIV
EKIV
EKVVEVPVEVPYEVEV
EKII
EKIV
EKPVVQ
EKIVKQIV
EKVVEVPVEIQI
EKIV
EKPVIK
EKIVEVPVEVPYEI
EKIV
EKPVIK
EKIVEIPVEVPYEI
EKIV
EKPVIK
EKII
EKIVEVPVEVPYQVLV
EKIV

19

Pseudocohnilembus persalinus KRX06821.1

Second half of sequence

EKPVIK
EKIVEVPVEVPYQVEV
EKIK
ERPVQQ
EKIV
EKIV
EKVVEVPVEVPYKVEV
EKIIKKIV
EKVVEVPVEIII
EKPA
EKIV
EKVVEVPVEVIV
EKMVEVPQKKQQEFKKNQPNQQLS
EKAPQII
EKIVEVPVEVPYKVEV
EKIV
EKVVEVPVE

LKLKKKRQSKSPQNIQYI

20

Pseudocohnilembus persalinus KRW98423.1
First half of sequence

Articulin. VYV motifs.

MV

EKLV
EKEIIV
DRPVEVIKNVYV
EKEVEVPVYI
EKIV
DRPYEVIKTV
EKAIEVPIEVPIEVPVEVPYEVIV
EKRVEVPYETKVYI
DKLVEIPIEVPVEVPYEVRVEVPV
EKRIEIPV
EKTVYV
DKLVEIPIEVPVEIPYEVVV
EKRVEVPV
EKTVYV
DKYV
DRLVEIPIEVPVEIPYEVRVEVPV
EKRIEIPV
EKTVYV
DKYI
DRLVEIPIEVPVEIPYEVVV
EKRVEVPV
EKRIEVPV
EKTVYV
DKYI
DRLVEIPIEVPVEIPYEVIV
EKEVFRTV
EKEIEVPVYI
EKIV
EKPV
EKIV
EKIVEV
EKFIEV
EKPVRQQPPQ
EKIVEVFV
DREIEVPVYI
EKIV
DRIVEV
EKRVEVPV
EKIV
ERIVEVPIEVPVEVPYQIETAKQVPTIQYQ
DRIV
EKIV
ERIVEIPIEVPVEVPYEIETARRAQPQEIQ
ERIV
EKIV
EKRVEVPV
ERIV
ERIVEVPIEVPVEVFV
DRIV
EKIV
EKRVEVPV
DRIV
EKIV
ERIVEVPIEVPVEVPYEIETARRAQPQEIQ

21

Pseudocohnilembus persalinus KRW98423.1
Second half of sequence

ERIV
EKIVEVPV
EKIV
ERFIEIPIEVPVEVPYEIEAARRAQPQEIQ
ERIV
EKIV
EKPVYI
EKEVEVPVYV
DRIV
EKIVEI
EKIV
EKIVEVPVEI

FKEVESKQAANFQTQYVEVEVEVPVYVDRIVEVPVEKIVEVPVEKIVEVFRE
YPNNQIQQAPQVIKEYVEVEVEVPVYVDRIVEQTIENQIFSDNSERITQLQQQ
NGQLQAYSDELKKLNSQLSQKISSLYEQIETSKTSINQTQSKCENCQDMTRL
VLAGAEIESLRIFDQTEVVLIEPRARSSQYVMTGMTGEIDGRASQYLQPGKD
YKSPIPRASFTNNRYSQYNNNQRVVVSPNPRDSSRERLLMGSQVQRTSHTI
TRSQQANMKNSQQANVFVSHTNLTNSNIQQQQRQQFQRNL

```
  1 M V E K L V E K E I I V D R P V E V I K N V Y V E K E V E V P V Y I E K I V D R P Y E V I K T V E K
 51 A I E V P I E V P I E V P V E V P Y E V I V E K R V E V P Y E T K V Y I D K L V E I P I E V P V E V
101 P Y E V R V E V P V E K R I E I P V E K T V Y V D K L V E I P I E V P V E I P Y E V V V E K R V E V
151 P V E K T V Y V D K Y V D R L V E I P I E V P V E I P Y E V R V E V P V E K R I E I P V E K T V Y V
201 D K Y I D R L V E I P I E V P V E I P Y E V V V E K R V E V P V E K R I E V P V E K T V Y V D K Y I
251 D R L V E I P I E V P V E I P Y E V I V E K E V F R T V E K E I E V P V Y I E K I V E K P V E K I V
301 E K I V E V E K F I E V E K P V R Q Q P P Q E K I V E V F V D R E I E V P V Y I E K I V D R I V E V
351 E K R V E V P V E K I V E R I V E V P I E V P V E V P Y Q I E T A K Q V P T I Q Y Q D R I V E K I V
401 E R I V E I P I E V P V E V P Y E I E T A R R A Q P Q E I Q E R I V E K I V E K R V E V P V E R I V
451 E R I V E V P I E V P V E V F V D R I V E K I V E K R V E V P V D R I V E K I V E R I V E V P I E V
501 P V E V P Y E I E T A R R A Q P Q E I Q E R I V E K I V E V P V E K I V E R F I E I P I E V P V E V
551 P Y E I E A A R R A Q P Q E I Q E R I V E K I V E K P V Y I E K E V E V P V Y V D R I V E K I V E I
601 E K I V E K I V E V P V E I F K E V E S K Q A A N F Q T Q Y V E V E V E V P V Y V D R I V E V P V E
651 K I V E V P V E K I V E V F R E Y P N N Q I Q Q A P Q V I K E Y V E V E V E V P V Y V D R I V E Q T
701 I E N Q I F S D N S E R I T Q L Q Q Q N G Q L Q A Y S D E L K K L N S Q L S Q K I S S L Y E Q I E T
751 S K T S I N Q T Q S K C E N C Q D L M T R L V L A G A E I E S L R I F D Q T E V V L I E P R A R S S
801 Q Y V M T G M T G E I D G R A S Q Y L Q P G K D Y K S P I P R A S F T N N R Y S Q Y N N N Q R V V V
851 S P N P R D S S R E R L L M G S Q V Q R T S H T I T R S Q Q A N M K N S Q Q A N V F V S H T N L T N
901 S N I Q Q Q Q R Q Q F Q R N L
```

22

Pseudocohnilembus persalinus KRX09694.1
First half of sequence

Articulin. VYV motifs.

MRSSIINGQQQQLVSSVNRGSFFQRQTLVSQSPKRLVSATNYTVVPSQQQIL
TGSVKSVTQNTSSQQLVQIISPVKKVQQVQQLQQSSPNTITHSHT

EKVI
EKPVYY
EKEV
EKVI
EKPVYY
EKEV
EKVI
EKPVYY
EKIV
EKPVEV
EKIV
EKIVEVPV
EKIVYV
EKPVEI
EKIVYV
DKIV
EKIIEVPIEVPVEVPYEVPVEVQKRIEVPV
EKIVYV
DKIVEVPV
EKTVYV
DKYI
EKLVEIPIEVPVEVPYEVIV
EREKRVEIPV
EKTVYV
DKVVEVPQYI
EKLIEIPIEVPVEVPYEVIV
EREKRVEIPV
EKTVYV
DKVVEVPQYI
EKLIEIPIEVPVEVPYEVIV
EREKRVEIPV
EKTVYV
DKYI
DKLVEIPIEVPVEVPYEVMV
EREKRVEIPV
EKTVYV
DKIVEIEIPKYI
EKLVEIPIEVPVEVPYEVIV
EREKRVEIPV
EKTVYV
DKYI
DRPV
EKTVYV
DKYV
DRLVEIPIEVPVEVPYEVIV
EREKIIEIPV
EKTVYV
DKYV
DRLVEIPIEVPEVPYEVIV
ERERLV

23

Pseudocohnilembus persalinus KRX09694.1
Second half of sequence

EKRVEIPV
EKTVYV
DKYI
DRLVEIPIEVPVEIPYEVVV
EKERRVEIPV
EKTVYI
DKFI
EKLVEIPIEVPVEVPYEVIV
EREKLI
EKRVEIPV
EKTVYV
DKYV
DRLVEIPIEVPVEIPYEVVV
EKERRVEIPV
EKTIYV
DKYV
DRLVEIPIEVPVEIPYEVLV
EKRVEVPV
EKTVYQ
ERIVEIPIEIPVEVPYEV

LIERNIVDENRVKQVQAELEEWRRKFDMKLVEENRYREQIRLLELEVKDWMRK
YEEEVNTETHYEVVSVKSRKSIYNSSTSQLNLRNSQVRAPEQVIDAVKRASLY**G**
DMKNSHTYNSNSNLHQQQQYTNSQVQNGVTTLSYQNSSQSQTTYVNGGSNIK
QMSPNYRTTQSRQFIVGNNQ

24

Pseudomicrothorax dubius L41557

Articulin

MIPNTALQAGQIGSGVGPFGAGQFQGVPGFASPQYNFALQGQLQAALQAAGTPVGAIPGLL
GSAAAGLGFANLIPQQVANPALSVSAPVPNQSHQFFAENQRLQGQLQR**G**NALISELKALVTQ
LASQPPQVVNIPQVV

DRVVPVQQVIDQIVP
REV
EKPFPVDVPV
EKIV
ERQVPFPV
ERKVDVPYVVT
RDVEVPRYV
DKEITVMKYVDVPVDVPIYVPVYQ
DKVVEVPVYV
DKIMEVPV
DKPVYVQ
RDIIV
ERPIII
EKKVEIPV
ERQVVV
EKPIIVEV
ERLI
EKPVY

STKTVDIPIEHERDVVLTSVVDQPVNYQVVVPKVVDTPVEVPVNVPVDVPIEVPVPVDRDVPV
PFQLNIDVPVDVPVARPVPVERIIQQPIPLEQPRLVEQHVPIPHPVPVAQEVIVQQPFAVPQPY
TVQQEVPIPHPVPVPQPYAVPQPVPVPTPVAVPQPVTVQQPVTIQQAVPVAQPV**G**VQSTVQI
PLQQSGYVQRSIGLAPQGFSPVGVQGTFQLPQNAVYAGNVGFGQGGFVGGPAAFNVGQAG
LIGTPGRMTAQNVAGFGQFGGAQLQNGSF

25

Pseudomicrothorax dubius Articulin 1 AAC15830.1

Articulin

MAQVVGTPGRVIGGNAFGANLIGNRVGVAPANVLGAQQLGLNRFGTPLGAGV
VNANAFQLNGLIGARGLANSVVQASSASAGNIAAAQLQRSLVGAGSRVGIAPLA
ANQALLTNEFANWRARASTVNGLIATPPAQLGLVDLQARALPYGAFQAQVPQQ
VVNLPQVV

DRVVPVPHVI
DRIVPRAVDTPYQVDVPV
ERIV
DRPVQFPV
DRPYDVPYVVT
RDVEVPRVV
DKPIAVPRYVDVPFDVPIVVPRYNDIIVEVPVYV
EKRVEVPV
DKPIYVQ
KDVVV
EKPVII
ERKVEVPV
ERQIVVPKPVYVEVERIVEVPVY

SQRVVDVPIEHERSVLLTSIVDQPVNRPVTVPKVVDTPFEVPVNVPVDVPVQIPIQV
DVERPVPVPFNVDVPVDVPIQRPIPVERVFHNPVPIEQPRIIDQPIPFQHPVPVPQP
VTVQQGVPVPQPVRVPQPVGIPQAVPVPHPVAVPQPVAVPQPYAVEQPYAVQQQ
VRVQEPVAVPNPVAVPQPYAVPQPYAVQQPVRVQEPVPVGVQTVVQPQFAVGV
QTVGLQQGVIGVSPRIGVQNINAGRFPVVNGGLGLAGVAGVQNLAGIQNFAGIQNL
AGIQNFAGLQGVAGVQNLASLRAVNGLAGQVNLASSRVAAAPGLINNSLVLAGGVL
GNSRVL

26

Pseudomicrothorax dubius Articulin 4 AAC15831.1

Articulin

MAFVNNNQLLNSRLLNGQVVNGQVLNAAARPGFVGAQVLPGQNVFGSNVLGA
RAQFGVGVGSVDSPRIGQQVLPQGFVGSRVGLQVPLHPQFFNAPVPQTLVQQ
TVVENIVEQPQIVENPVEQIF

ERPVPVAVP
REVTIQRDVPVPV
DRPV
ERPVPVEQIIQ
KELLI
ERPAFFEEVI
ERPVPRPVYVEQII
DRQI
EKPVVIEQIVEVPV
ERPVYIEVENPI
EKEVVV
EREIPVPV
ERQVRIEQPVYHFI
EKPVEVPVLFENIVQ
RDIHI
DRPIY

SQQAVPRPVDFPVHAEQVVQRPVEVPRQYPVQVPRPVPAPVQVPRDVAVPVPVER
QIPIERPVEVPFAVDRYVDVPVPVDVPVPIGRPVPQPVQVPQPYQVIQPVAVPQPYHV
PEPVPVAQPYQVPQPVPVPQAVPVPHPVPVPQPTQYIEQVPVVERVPVPHNVPVPQ
PVAVPHPVPVVEQVPVVEKVPVPVYNNFYAQPQVVGANFRVNQVAGAFPVQGYGIQ
GVQGIQGIQGIQGFGTPVGVAGLNRAVGLNVANAGLLGSSIIGRAL

Tetrahymena ALV1 EAS06387.1

Predicted secondary structure next slide

MNQDQADHIPVKEGSNLANDNQDQNKQPQRVSKIQQALSSFRSGVEQIYSES
RERSLGRLKESEDEMQRKFSIQKVINNDVGELQNIGQDNLTRFLSDEHNEIT
EAQKLDCYVIREQIKSSKNREEEEERIYRQQIEKLHKKNQELEEQIQQKKRQ
QRINSKNSSLNSFVKSSIDVVPKNFIPTALPFPSIEQKMQQFDISVYNNPLQ
GYQDQVVNTNQFQGQQQDIKQAKNQKGKTQQLDFKPIQTQIDNIPVYAAGYE
QEQQDIQVVYEGEKRDLNKCLRKFAKKIFYLQRMVQDLTAERDKWKTQFEGV
DSQY

REYRLKYEEVIEHNKLQ
EKQMSENKVNL
EKYKTMAVALEEEVN
KLRLYNHGSGDPEVQIV
EKLI
EKPIEIVKHVVL
EKIV
EKPIEIIRTIEAPPIEI
EKIV
EKIVEVPV
ERIV
EKVVQVPFETIV
EKIIEVPKIEIV
EKEKIIEVPRPEYI
EKLVHV
EKPVYI
DRPQIV
KEVIE

TKDHGLERELMMVREDYHKLQMIQENQFKELEDWKLKCIRLEDQFRTNEADK
SVMIERDRSVLMDEIKNSHQTIDNLRDRIEMLYKEISMLTDNNNRKNIEMSNQIR
FLEEQLAISAPRIISTTSYQGLPAYQPVPPPIVENTTAYYKTTAPAYPNNGYSSL
QQPISNPQFNNSQSYQQTFQQTNNQENLPYSNSRYQLQKDGQIDPNYIKFISK
IYELIIGQPDSINTPESRKNQKILKYIKKNNLALAKKYLQIILMSLQIPQQQFNSLSS
KALNSISNNNDNLSSTERLNTSSNQNQQKQQALLFTPNSQNTQKSMTPQPHLQ
QQFQQVGLVSPNIYTNSSSLQNNNANINTSNMRFQKNLNSLAFNNHNDIKNQQ
TQLIVHHQNNSSNNSESLCSDLRSFFQDNISGINSQFTGTLSVNKNNNNNYSSII
QGSKNGSMNSNQHRIQVMTPSTNDDKSILSQNNHFISTNPKQVDLSSSSQSHH
NQHLLHSQVHSKNVSQTTQMSVFNNSGEISQFNYSSLGNNQNFQSQNQMSNIS
SVINNINFENDTLSSSQQQNTSISNISNTLQEAKCRFHPYKKVKYQMSQNENEKF
CTACALEYAKQGLDVEPIIKTQQKEINLYMNEMLNVKNINDMLMNSLDSKQEFIKE
HSRKQSQIVQDLFLQIEKLIYEQKTQLLSQINNKMWEQINLIELKKNDSRLFEQKLL
LKQQDIEKNYQQITTAMGVSEVREILFFYNSELDQIKTSISDMFKNTSFDLINLNQN
QIAQQIQILQKFLQEEVFKPTITQSTLQESINLLSNKSNSPSKNDKNQLSRFMSNQ
DKKGSGKKQQYQQQQIQNKTLAQQLQNYHIEQSQLQPNVQKVIELNCAMNKQNT
QSQNLQKQNYQQNEQMNFNQQKEQQQMFATQLQQKQLQQQQQQQQYNEIV
DTSISEKQHTQESDDLSSAQGIYLNIQNQSKISNKSNISSHKIINYSDIHDEQNLNILK
DSSSPSTSAKQIVRDSKATPVTQEKKPFFPFQCLSPQQTSKGEGTLRQQYSISKDN
QLNQRQKQFIQTEPTHLPSNKTKQGISKPGNYQRNLLMNDEIATSNNRFKFNQNPT
NNFSLLTHFNKQTNSNIQNLVAVPQINNKQQEQAQNFTNQSKLNAINNIRQLQINLV
DQDSNQKSLTSRPIIMNQINNNQQFANSQNQQPNFVAQLIQQKQQNTLKNTPNY
QQLQNNDYNSMSSLKN

Tetrahymena ALV1 EAS06387.1

28

Predicted secondary structure

```
   1 M N Q D Q A D H I P V K E G S N L A N D N Q D Q N K Q P Q R V S K I Q Q A L S S F R S G V E Q I Y S
  51 E S R E R S L G R L K E S E D E M Q R K F S I Q K V I N N D V G E L Q N I G Q D N L T R F L S D E H
 101 N E I T E A Q K L D C Y V I R E Q I K S S K N R E E E E E R I Y R Q Q I E K L H K K N Q E L E E Q I
 151 Q Q K K R Q Q R I N S K N S S L N S F V K S S I D V V P K N F I P T A L P F P S I E Q K M Q Q F D I
 201 S V Y N N P L Q G Y Q D Q V V N T N Q F Q G Q Q Q D I K Q A K N Q K G K T Q Q L D F K P I Q T Q I D
 251 N I P V Y A A G Y E Q E Q Q D I Q V V Y E G E K R D L N K C L R K F A K K I F Y L Q R M V Q D L T A
 301 E R D K W K T Q F E G V D S Q Y R E Y R L K Y E E V I E H N K L Q E K Q M S E N K V N L E K Y K T M
 351 A V A L E E E V N K L R L Y N H G S G D P E V Q I V E K L I E K P I E I V K H V V L E K I V E K P I
 401 E I I R T I E A P P I E I E K I V E K I V E V P V E R I V E K V V Q V P F E T I V E K I I E V P K I
 451 E I V E K E K I I E V P R P E Y I E K L V H V E K P V Y I D R P Q I V K E V I E T K D H G L E R E L
 501 M M V R E D Y H K L Q M I Q E N Q F K E L E D W K L K C I R L E D Q F R T N E A D K S V M I E R D R
 551 S V L M D E I K N S H Q T I D N L R D R I E M L Y K E I S M L T D N N N R K N I E M S N Q I R F L E
 601 E Q L A I S A P R I I S T T S Y Q G L P A Y Q P V P P P I V E N T T A Y Y K T T A P A Y P N N G Y S
 651 S L Q Q P I S N P Q F N N S Q S Y Q Q T F Q Q T N N Q E N L P Y S N S R Y Q L Q K D G Q I D P N Y I
 701 K F I S K I Y E L I I G Q P D S I N T P E S R K N Q K I L K Y I K K N N L A L A K K Y L Q I I L M S
 751 L Q I P Q Q Q F N S L S S K A L N S I S N N N D N L S S T E R L N T S S N Q N Q Q K Q Q A L L F T P
 801 N S Q N T Q K S M T P Q P H L Q Q Q F Q Q V G L V S P N I Y T N S S S L Q N N N A N I N T S N M R F
 851 Q K N L N S L A F N N H N D I K N Q Q T Q L I V H H Q N N S S N N S E S L C S D L R S F F Q D N I S
 901 G I N S Q F T G T L S V N K N N N N N Y S S I I Q G S K N G S M N S N Q H R I Q V M T P S T N D D K
 951 S I L S Q N N H F I S T N P K Q V D L S S S S Q S H H N Q H L L H S Q V H S K N V S Q T T Q M S V F
1001 N N S G E I S Q F N Y S S L G N N Q N F Q S Q N Q M S N I S S V I N N I N F E N D T L S S S Q Q Q N
1051 T S I S N I S N T L Q E A K C R F H P Y K K V K Y Q M S Q N E N E K F C T A C A L E Y A K Q G L D V
1101 E P I I K T Q Q K E I N L Y M N E M L N V K N I N D M L M N S L D S K Q E F K E H S R K Q S Q I V Q
1151 D L F L Q I E K L I Y E Q K T Q L L S Q I N N K M W E Q I N L I E L K K N D S R L F E Q K L L L K Q
1201 Q D I E K N Y Q Q I T T A M G V S E V R E I L F F Y N S E L D Q I K T S I S D M F K N T S F D L I N
1251 L N Q N Q I A Q Q I Q I L Q K F L Q E E V F K P T I T Q S T L Q E S I N L L S N K S N S P S K N D K
1301 N Q L S R F M S N Q D K K G S G K K Q Q Y Q Q Q Q I Q N K T L A Q Q L Q N Y H I E Q S Q L Q P N V Q
1351 K V I E L N C A M N K Q N T Q S Q N L Q K Q N Y Q Q N E Q M N F N Q Q K E Q Q Q M F A T Q L Q Q K Q
[illegible]

   1 V D T S I S E K Q H T Q E S D D L S S A Q G I Y L N I Q N Q S K I S N K S N I S S H K I I N Y S D I
  51 H D E Q N L N I L K D S S S P S T S A K Q I V R D S K A P V T Q E K K P F F P F Q C L S P Q Q T S K
 101 G E G T L R Q Q Y S I S K D N Q L N Q R Q K Q F I Q T E P T H L P S N K T K Q G I S K P G N Y Q R N
 151 L L M N D E I A T S N N R F K F N Q N P T N N F S L L T H F N K Q T N S N I Q N L V A V P Q I N N K
 201 Q Q E Q A Q N F T N Q S K L N A I N N I R Q L Q I N L V D Q D S N Q K S L T S R P I I M N Q I N N N
 251 Q Q F A N S Q N Q Q P N F V A Q L I Q Q K Q Q Q N T L K N T P N Y Q Q L Q N N D Y N S M S S L K N
```

29

Tetrahymena ALV2 ACO05021.1

Predicted secondary structure next slide

MSIQSSNFGSLNKNTLDLQFREYGNIDDVLKYQKSRIIQSSDIEQQQGSGSN
NALILSCP ISHECDSLNNRIIIPNAQTILQETIQTDTANLLGSLEAGNIPQKSGL
QKNQSEFLEQKLNSMKQKHKQLQQQLSEVSKIDSVSNKKKNVVHSQSTQG
FFKGGVSTSNLDMFNFGSLKNQIKQMDVISNILSSIQLKPNNNQQ

KDIEVII
EKNDLYRILKILIDQLVSYI
EKLNQVVS
ERDIIDIQRQNA
EKLNNEL
KETIRILEEELKIYKNQSTETKIEL
EKQKALYDIL
EKKYLDLLKRKQEVKIIN
KETV
KEIVI
EKPIEIL
KEINNVRIVKQK
KEVRKIIRPQAEVKHTI
EKKLHIL
EREKIVPQIQVI
EKV
KEIPKIEFV
DKEKVI
EKPFFV
EKLV
DRFVEVPRFIP
KEVLV
DRPVYV
DRPV
EKIVKVPQIV
EKEKLV
ERPVFI
EKPV
DRFI
EKETFI
DRPVYIQEPPVHI
EKVIEVPKII
EKQKII
DRPIYINQPRI
ERVI
EKPVFINKYEPIN

SQLVNSYSTPTISTQFNSPAANYQRFIPEQISRQNNFLSPFNSIQQTFSQDL
TSPSYIKQYV

Tetrahymena ALV2 ACO05021.1

Predicted secondary structure

```
  1 M S I Q S S N F G S L N K N T L D L Q F R E Y G N I D D V L K Y Q K S R I I Q S S D I E Q Q Q G S G
 51 S N N A L I L S C P I S H E C D S L N N R I I I P N A Q T I L Q E T I Q T D T A N L L G S L E A G N
101 I P Q K S G L Q K N Q S E F L E Q K L N S M K Q K H K Q L Q Q Q L S E V S K I D S V S N K K K N V V
151 H S Q S T Q G F F K G G V S T S N L D M F N F G S L K N Q I K Q M D V I S N I L S S I Q L K P N N N
201 Q Q K D I E V I I E K N D L Y R I L K I L I D Q L V S Y I E K L N Q V V S E R D I I D I Q R Q N A E
251 K L N N E L K E T I R I L E E E L K I Y K N Q S T E T K I E L E K Q K A L Y D I L E K K Y L D L L K
301 R K Q E V K I I N K E T V K E I V I E K P I E I L K E I N N V R I V K Q K K E V R K I I R P Q A E V
351 K H T I E K K L H I L E R E K I V P Q Q V I E K V K E I P K I E F V D K E K V I E K P F F V E K L V
401 D R F V E V P R F I P K E V L V D R P V Y V D R P V E K I V K V P Q I V E K E K L V E P V F I E K P
451 V D R F I E K E T F I D R P V Y I Q E P P V H I E K V I E V P K I I E K Q K I I D R P I Y I N Q P R
501 I E R V I E K P V F I N K Y E P I N S Q L V N S Y S T P T I S T Q F N S P A A N Y Q R F I P E Q I S
551 R Q N N F L S P F N S I Q Q T F S Q D L T S P S Y I K Q Y V
```

31

Tetrahymena XP_012656168.1

This may be the same gene as ALV1 or it may be an orthologue.

Predicted secondary structure next slide

MNQDQADHIPVKEGSNLANDNQDQNKQPQRVSKIQQALSSFRSGVEQIYSESR
ERSLGRLKESEDEMQRKFSIQKVINNDVGELQNIGQDNLTRFLSDEHNEILTEAQ
KLDCYVIREQIKSSKNREEEEERIYRQQIEKLHKKNQELEEQIQQKKRQQRINSKN
SSLNSFVKSSIDVVPKNFIPTALPFPSIEQKMQQFDISVYNNPLQGYQDQVVNTNQ
FQGQQQDIKQAKNQKGKTQQLDFKPIQTQIDNIPVYAAGYEQEQQDIQVVYE**G**
EKRDLNKCLRKFAKKIFYLQRMVQDLTA

ERDKWKTQFEGVDSQY
REYRLKYEEVIEHNKLQ
EKQMSENKVNL
EKYKTMAVALEEEVNKLRLYNHGSGDPEVQIV
EKLI
EKPIEIVKHVVL
EKIV
EKPIEIIRTIEAPPIEI
EKIV
EKIVEVPV
ERIV
EKVVQVPFETIV
EKIIEVPKIEIV
EKEKIIEVPRPEYI
EKLVHV
EKPVYI
DRPQIV
KEVIE

TKDH**G**LERELMMVREDYHKLQMIQENQFKELEDWKLKCIRLEDQFRTNEADKSVMI
ERDRSVLMDEIKNSHQTIDNLRDRIEMLYKEISMLTDNNNRKNIEMSNQIRFLEEQLA
ISAPRIISTTSYQGLPAYQPVPPPIVENTTAYYKTTAPAYPNNGYSSLQQPISNPQFNN
SQSYQQTFQQTNNQENLPYSNSRYQL

32

Tetrahymena XP_012656168.1

Predicted secondary structure

```
  1 M N Q D Q A D H I P V K E G S N L A N D N Q D Q N K Q P Q R V S K I Q Q A L S S F R S G V E Q I Y S
 51 E S R E R S L G R L K E S E D E M R K F S I Q K V I N N D V G E L Q N I G Q D N L T R F L S D E H N
101 E I L T E A Q K L D C Y V I R E Q I K S S K N R E E E E E R I Y R Q Q I E K L H K K N Q E L E E Q I
151 Q Q K K R Q Q R I N S K N S S L N S F V K S S I D V V P K N F I P T A L P F P S I E Q K M Q Q F D I
201 S V Y N N P L Q G Y Q D Q V V N T N Q F Q G Q Q Q D I K Q A K N Q K G K T Q Q L D F K P I Q T Q I D
251 N I P V Y A A G Y E Q E Q Q D I Q V V Y E G E K R D L N K C L R K F A K K I F Y L Q R M V Q D L T A
301 E R D K W K T Q F E G V D S Q Y R E Y R L K Y E E V I E H N K L Q E K Q M S E N K V N L E K Y K T M
351 A V A L E E E V N K L R L Y N H G S G D P E V Q I V E K L I E K P I E I V K H V V L E K I V E K P I
401 E I I R T I E A P P I E I E K I V E K I V E V P V E R I V E K V V Q V P F E T I V E K I I E V P K I
451 E I V E K E K I I E V P R P E Y I E K L V H V E K P V Y I D R P Q I V K E V I E T K D H G L E R E L
501 M M V R E D Y H K L Q M I Q E N Q F K E L E D W K L K C I R L E D Q F R T N E A D K S V M I E R D R
551 S V L M D E I K N S H Q T I D N L R D R I E M L Y K E I S M L T D N N N R K N I E M S N Q I R F L E
601 E Q L A I S A P R I I S T T S Y Q G L P A Y Q P V P P P I V E N T T A Y Y K T T A P A Y P N N G Y S
651 S L Q Q P I S N P Q F N N S Q S Y Q Q T F Q Q T N N Q E N L P Y S N S R Y Q L
```

33

Tetrahymena XP_001012752.2

This may be the same gene as ALV2 or it may be an orthologue.

Predicted secondary structure next slide

MSIQSSNFGSLNKNTLDLQFREYGNIDDVLKYQKSRIIQSSDIEQQQGSGSNNAL
ILSCPISHECDSLNNRIIIPNAQTILQETIQTDTANLLGSLEAGNIPQKSGLQKNQS
EFLEQKLNSMKQKHKQLQQQLSEVSKIDSVSNKKKNVVHSQSTQGFFKGGVST
SNLDMFNFGSLKNQIKQMDVISNILSSIQLKPNNNQQ

KDIEVII
EKNDLYRILKILIDQLVSYI
EKLNQVVS
ERDIIDIQRQNA
EKLNNEL
KETIRILEEELKIYKNQSTETKIEL
EKQKALYDIL
EKKYLDLLKRKQEVKIIN
KETV
KEIVI
EKPIEIL
KEINNVRIVKQK
KEVRKIIRPQAEVKHTI
EKKLHIL
EREKIVPQIQVI
EKV
KEIPKIEFV
DKEKVI
EKPFFV
EKLV
DRFVEVPRFIP
KEVLV
DRPVYV
DRPVNQFI

NNNRIQQKIKQKRLKKQLKCLKQQKKKNQSRDQYLLKNQLTDLQKRKLLLIDQSIFK
NLQYIQKKLQKFLRLQKSKKQLIDLFTSINHVQKELLKNLYSQINMSQQILSQLIHILLQ
LFQHNLTHLLLIIRDLYLSKLVDKITFCHLLILYSKHFLKIQLHLHTLNSMFEKYLFVIVK
QYSLIKISLFLKFQISLYLIISQSFTFQKINLFCKIKNYQKCIFVFQQTILQPLINLLLSDKF
IQQKQFMILFIVSKQKIDILIFYNQYVNFLF

34

Tetrahymena XP_001012752.2

Predicted secondary structure

```
  1 M S I Q S S N F G S L N K N T L D L Q F R E Y G N I D D V L K Y Q K S R I I Q S S D I E Q Q Q G S G
 51 S N N A L I L S C P I S H E C D S L N N R I I P N A Q T I L Q E T I Q T D T A N L L G S L E A G N I
101 P Q K S G L Q K N Q S E F L E Q K L N S M K Q K H K Q L Q Q Q L S E V S K I D S V S K K K N V V H S
151 Q S T Q G F F K G G V S T S N L D M F N F G S L K N Q I K Q M D V I S N I L S S I Q L K P N N N Q Q
201 K D I E V I I E K N D L Y R I K I L I D Q L V S Y I E K L N Q V V S E R D I I D I Q R Q N A E K L N
251 N E L K E T I R I L E E E L K I Y K N Q S T E T K I E L E K Q K A L Y D I L E K K Y L D L L K R K Q
301 E V K I I N K E T V K E I V I E K P I E I L K E I N N V R I V K Q K K E V R K I I R P Q A E V K H T
351 I E K K L H I L E R E K I V P Q I Q V I E K V K E I P K I E F V D K E K V I E K P F F V E K L V D R
401 F V E V P R F I P K E V L V D R P V Y V D R P V N Q F I N N N R I Q Q K I K Q K R L K K Q L K C L K
451 Q Q K K K N Q S R D Q Y L L K N Q L T D L Q K R K L L L I D Q S I F K N L Q Y I Q K K L Q K F L R L
501 Q K S K K Q L I D L F T S I N V Q K E L L K N L Y S Q I N M S Q Q I L S Q L I H I L L Q L F Q H N L
551 T H L L L I I R D L Y L S K L V D K I T F C H L L I L Y S K H F L K I Q L H L H T L N S M F E K Y L
601 F V I V K Q Y S L I K I S L F L K F Q I S L Y L I I S Q S F T F Q K I N L F C K I K N Y Q K C I F V
651 F Q Q T I L Q P L I N L L L S D K F I Q Q K Q F M I L F I V S K Q K I D I L I F Y N Q Y V N F L F
```

35

Tetrahymena >XP_001017441.1

MSSNENQSEQEKAQVLQGTKPVYQYPAYTSQYVQQQPLIQSQLVTQPVQYV
QQPVQYVQQTVQGVHQSALSQSQAVASGLVATQIQ

REVVKGESRIEYIPY
EKSVIEYEAVQRVDYVPK
EKKITDYYAVEYQTEYLPQVYQ
DRYIEYIPT
ERIQ
ERVEYQAIQKQIVHQPV

QQQQQVLQTVVQPATTYLQSSQVTVPVVQQQFVPTASVVHTTIPATQVVQGA
THVVQGASTVVQNVPTAQFVQTVPTAFSTQTLGVPVVRAGLTTSGVRTLGPV
PYSVVEEAIKKSQKKDE

Tetrahymena EPI21 XP_012653403.1, TTHERM_000481318

MSEYSFFAQLYAQMNEQNLQNSWIDYGLAEENKIYFCQLKGFKKEEISEYFI
QQILINKQKIFRKEKENFKEETNNLQLLANITKMSDQPEQAQQQANLQQQQQ
PPVQTQVLQYPFQYVQQPLVQSQVVAQPIYTNGQVPTTQFLGSTLIQGATQ
QVSAGTVLHQSGVQVSGVPVQTTLQ

REVVKGESRIEYIPY
EKTVIEYEAVQRIEYVPK
EKKVTDYYAVEYQTEYIPQVYQ
DRYIEYVPT
ERVQ
ERVEYQAI
EKQVVHQP

AQEVSQVSQVPVSSVSQVPVAQQLTSSQVHVLPPTQTVISGGQQVAYQNQS
FAYAPVQQSVVYTTPAQLGQSVYRGPTVLTNSIAQKPVQQNAKKEEKTFLER
IFE

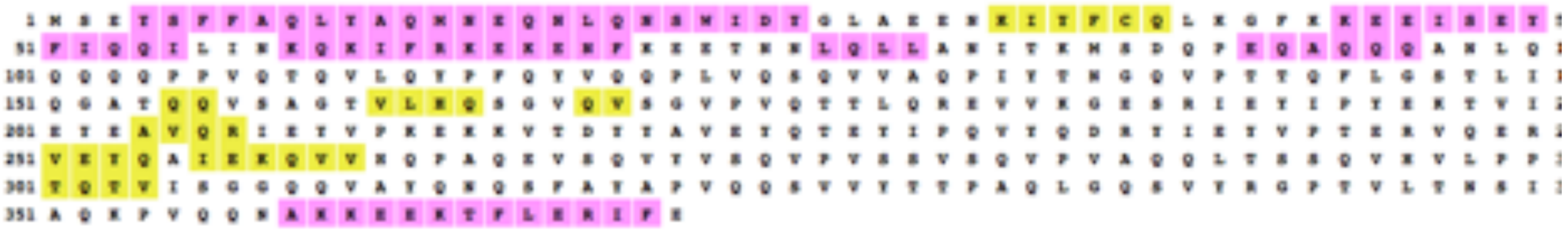

Supplement: TEXT S3 [file mbo005184120s3.pdf]
