# Supplementary material for: Epiplasts: Membrane Skeletons and Epiplastin Proteins in Euglenids, Glaucophytes, Cryptophytes, Ciliates, Dinoflagellates, and Apicomplexans
Source: mBio. 2018 Oct 30;9(5):e02020-18. doi: 10.1128/mBio.02020-18 (PMC6212826; doi:10.1128/mBio.02020-18)
Supplement: TEXT S4 [file mbo005184120s4.pdf]

1

Key to Gene Annotations

- **General organization:** The medial acid-base dyads (ABDs) are “parsed” into “strings that initiate with a dyad. N-term domain is denoted the head, C-term the tail.

- **Highlights**: Yellow, tyrosine; Green, cysteine; Pink, VPV; Gray, ABD in predicted head and tail domains.

- **Colored font:** Red, glycine residues in ABD domains; Bold-faced red, last G residue in head and first G residue in tail; Blue, alanine residues in ABD domains; Green, repeated string domains.

- **Underscores:** Acid-base triads or tetrads.

- **Commentary**: Notes on distinctive gene features (e.g. orthologues, localization patterns) in green font at top of some pages. Predicted homology domains (e.g. PDZ, coiled-coil) are given in Supplement Table 2.

- **Secondary structure**: PSIPRED predictions for a subset of proteins. Yellow, amino acids predicted in β-strand; pink, amino acids predicted in α-helix; no highlight, amino acids predicted in disordered (random coil) domain.

2

Chroomonas MMETSP0047_c33894_g1

Sequence truncated mid-ABD domain; scoring as articulin since all other *Chroomonas* epiplastins are articulins

…EIYV

DRVVANDVPTPY
DRLVDNFVEIEV
ERII
EKPVEVTV
ERVAVTEVPVEV
ERIV
EKTVWTEIPV
EKIVMQEVHVPV
DKII
ERSIPV
DRIVEIPI
DRMIPVHSP
EKIVHVQHHDV
ERM
ERDN

ADLWRVNTRLLAELREVKMSSFFSAKFECVEAGASSATLEWGPVHWAGGYTL
QCQEAERDE

3

Chroomonas MMETSP0047_c3950_g1

Articulin

...EII

ERVVEVEVI
KEVPV
ERIVEVPV
DKVV
DRYVEVPV
EKIV
ERF
REVPV
DKVVTQEVPVEV
ERVV
EKIVY
KEVPYDV
EKIV
EKVIEVPF
EKIVYQ
DKIVYQ
DKVVYQ
DKVVEVPV
ERVV

GNQYTSSNSSMMMSSSMREYRGEISGEVRSSPMAGGMYRSYGEARDVSHIMG
EGRMYGSTRIENGSGARMEGSRIEYGPNSRIEYGQRYSAEMYPQ

4

Chroomonas MMETSP0047_c6912_g1

Articulin

...VVI

KEVPVEI
ERVVY
KDRVVDVPV
ERII
EKMIEIPI
EKTIV
KEVHHEIPV
EKVIVQ
ERVVEVPV
ERVVY
KELSVEVPV
ERVVV
REVPVEVPV
EKVLI
KEVHVEVPV
EKIVM
KEVPITV
EKVVI
KEVPINVEPV

RMEPMASS**G**PPANRSWAPPEPRSPGPMTMRSSGASMRAPSPSPSARSGYMAM
AAPSQRSPSPAHSHLYSSQGGASTAGGRKVGLGLALMRAPDGSTCIHEAIPGFAA
FQSGQVHAGDVIVAIDDLSVAGYSLDDVKRLTQGEEGSFCALGLRRGDYEYKVVL
QRRNPPPAEDWESTSVQAPRRERSGSIRSNASMHSERPFAPGAL

5

Chroomonas MMETSP0047_c8616_g1

Articulin

…PISAPPPPPPPPI

DRIIEVT
KEVAV
DRPIEVT
KEVPI
DRYVEMQV
DRVVF
KEVPIEV
EKIHV
KEVAVPY
EKIVY
KDVQVPVEIPV
EKIV
EKVVV
KEVPV
EKITI
KEVPLPV
ERIVVQEVPVHI
DRVV
EKVVY
KDVPV
DRVQI
KEVPVPV
EKVVV
KEVKVPV
ERIV
DRVV
EKPV
DKVVYQ
DRIVYQ
DKIV

6

Chroomonas MMETSP0047_c9292_g1

Articulin

...KVVV

KEVPVPIEVPVQVIT
EKEVP
KEVTV
EKVVTQEVPVYV
DKIVT
KEVPVPI
ERIV
EKVV
EREVV
KEVPV
ERIITQEVPKYV
DRII
EKIVEVPV
EKIVT
KEVPITV
DRVV
EKVV
ERI
KEVPVYI
ERVET
KEVPVYV
EKVVVQEVPVNVEVIV
ERVI
EKIVEVPV
DRLV
ERIVEVPV
DRIVTNEVPVTVEHVV
EKIIT
KEVP
KEVYV
DRVCVNEVPVHV
DRVV
EKFIEVPI
ERIV
EKYVEVPV
ERVAITEVPVPV
DRIV
EKTVVQEVPV
EKIVI
KEVSVPV
EKVIYQEVPV
EKIVEQFRI

EHKESPELMTEVLGLRQANQRLLTEVRELKVSNIFPGRLFCASTTATSAKLQ
WSGVAWATAYTLHYAVGDEGQEPRYDSESWRRLYSGGETSYSATNLSSQ
HGYWFRVRAEGDGVHSRWSTPSLFVCTGDRISYGHTSLSHTHHSMISSDM
LTQGIC

7

Chroomonas MMETSP0047_c10313_g1

Articulin

…**G**TRS

ERSEPRTQTI
ERERIVEVPV
DRIVEVQRVV
ERVVEVEV
EKIVEVEV
EKVKYVKVEVPIEIQKIVYQ
DRWLEPEHKNV
EKVVEVPVEIT
KEIEV
EKRVEVPV
EKIVYQ
DRIVEVPIEVPIMVPGP
ERIVHVPGP
ERFIEIPGP
ERIIEVHVPVEVI
KEVIV
ERIVYR
DREPMESP

8

Chroomonas MMETSP0047_c25199_g1_i1_g48336

Articulin

...PPPPPKPEIR

DRYV
DRTIEVPV
DRIIEIPI
EKYI
DRFVEVPV
DRVVY
KEVVVEI
DREVI
KEVPQPV
ERIQY
REVPVPQEVAV
ERIV
ERIEL
KEVPV
EKII
EKQMPYTV
DRTVT
KEVPVFV
DKVV
EKVVP
REVPI
DKVLV
KEVPVEV
ERVVV
REIPEPF
DRVV
DRVVEVPI
ERIIYQ
DKIVYQ
DKVVYQ
DKIVEVPV
ERFVN

AEGPAARSEYSARIETGRGIIGQSIGYGSMGPRKVGLGLLLKTNNEGMTYVKE
TIKGFAASNQGTVQSNDIIIAVDGRSVEGWDLDSIKQLTFGDEGTQCTLTLRRG
ATTETVTLTRILPATQDLDSVAKNYSQY

9

Chroomonas MMETSP0047_c25974_g1
First half of sequence

Articulin

...FASRYALRTHRDQREGSPGHRQVIVCAAMIRTDKRILHGPWMSPSSPARG
RSMSPQRVQTTPFASDMSYVEQAPGPQERAFSPIPGARGPVAPELIQVHNNT
YQTMQRVQQQPQQIMELVEVHQVQQQPQYQPQVTQIVDVY

ERQVHVPVEVPVPQVH
EKVVYQ
DRPYEV
EKLV
EKHIDVPV
ERLVY
KEVPVPV
EKIVY
REVPVPVEVPVQQFYETTQI
KEVPV
DRVVMNEVPVQY
DKVVHHEVAKFV
DRIVANEVAVYV
DKIV
KEPFEVFS
ERIV
EREVPVTV
ERIV
KEEVAVTV
DRVVANEVPVYV
DRVVEVFN
EKIIEVPM
DKVVI
KEVPVTVEVPIQEVV
ERVQI
KEIPV
DRII
ERPVEIPV
DKII
EKVVV
KEV
EKVVTT
EKVVYV
DKVV
EKIVEIPV
DKLV
ERIVEVPV
ERIV
ERVVEVPV
EKIV
ERI
REVPVEHVI
EKIV

Chroomonas MMETSP0047_c25974_g1

Second half of sequence

10

ERI
KEVPVEHVV
ERVVEVPV
DRIV
ERVVEVPV
DRVVVQEVI
KEI
EKPVYI
EKIVEV
ERIVRQEVPVYV
DRVV
EKLV
EKFVEVPV
ERIV
EKIVEIPV
DRVVRTEVPVQVNV
DRIIETPIEIPV
EKVVT
KEVPVHV
DRII
EKMIEVPI
DRVI
EKTHHVEVPV
DRVITQEVPIHVPV

QHMVERPMPQMQMQMIHHHMEPVRQIEHVRHERRQQVHHVENPEALVLPY
EKEVQRIEVTPPRPEAYIVAEQHLRAENSRRSMSPHRSMSPPPPPG

1	F A S R Y A L R T H R D Q R E G S P G H R Q V I V C A A M I R T D K R I L H G P W M S P S S P A R G
51	R S M S P Q R V Q T T P F A S D M Y V E Q A P G P Q E R A F S P I P G A R G P V A P E L I Q V H N N
101	T Y Q T M Q R V Q Q Q P Q Q I M E L V E V H Q V Q Q Q P Q Y Q P Q V T Q I V D V Y E R Q V H V P V E
151	V P V P Q V H E K V V Y Q D R P Y E V E K L V E K H I D V P V E R L V Y K E V P V P V E K I V Y R E
201	V P V P V V P V Q Q F Y E T T Q I K E V P V D R V V M N E V P V Q Y D K V V H H E V A K F V D R I V
251	A N E V A V Y V D K I V K E P F E V F S E R I V E R E V P V T V E R I V K E E V A V T V D R V V A N
301	E V P V Y V D R V V E V F N E K I I E V P M D K V V I K E V P V T V E V P I Q E V V E R V Q I K E I
351	P V D R I I E R P V E I P V D K I I E K V V V K E V E K V V T T E K V V Y V D K V V E K I V E I P V
401	D K L V E R I V E V P V E R I V E R V V E V P V E K I V E R I R E V P V E H V I E K I V E R I K E V
451	P V E H V V E R V V E V P V D R I V E R V V E V P V D R V V V Q E V I K E I E K P V Y I E K I V V E
501	R I V R Q E V P V Y V D R V V E K L V E K F V E V P V E R I V E K I V E I P V D R V V R T E V P V Q
551	V N V D R I I E T P I E I P V E K V V T K E P V H V D R I I E K M I E V P I D R V I E K T H H V E V
601	P V D R V I T Q E V P I H V P V Q H M V E R P M P Q M Q M Q M I H H H M E P V R Q I E V R H E R R Q
651	Q V H H V E N P E A L V L P Y E K E V Q R I E V T P P R P E A Y I V A E Q H L R A E N S R R S M S P
701	H R S M S P P P P P G

11

Chroomonas MMETSP0047_c33934_g1

Articulin

…IIQQQQTMQVPIHFEDHSARRRLDISASPQPAP

REVV
RDVV
REVPV
DRIVQVPV
DRIV
DRVHEVV
KEVPV
DRVHEII
KEVPV
DRIIHHDVI
KEVPV
DRVHEVI
KEVPV
DRIVHVPVDVV
REVV
KEVPV
DREVV
REIPV
DRYHEII
KEVPI
DRV

12

Chroomonas MMETSP0047_c35882_g1

Articulin

…FTSRLFVSLQEPAAMAYTPQSNRGSRSRTPLREKTTANSADLRSFGDYDTG
AHTGDVLLQAT

ERVVSQ
ERLVDHAV
ERPINVPA
ERIV
EKVV
EKPKIV
EKNTVTEQPVIQQV
EKVVYVT
REVPVENII
EKQVVRKVVV
ERIVEQV
REVI
KEVPV
ERIVEQVVEVV
KEVPI
EKVV
ERIQI
KEIPVVETE
ERVVEVV
REVPVEIV
KEVPVYVKVD

AAEWDARHGLTHKSRAQYGGVGMLLGKFEGNHDRAGNIYVLELVPGTPAEQCH
LISLQDILISVEGEVVHGMHLSTVHQKVRGPEGTPVVLEFVRGSSGERYTVTLYRM
AAVSEGNAVYQHSNNVKNIRVVSAGFSPTTTRGTYDFYSQGEPNGSGYMANEQ
KERAYL

F T S R L F V S L Q E P A A M A Y T P Q S N R G S R S R T P L R E K T T A N S A D L R S F G D Y D T
G A H T G D V L L Q A T E R V V S Q E R L V D H A V E R P I N V P A E R I V E K V V E K P K I V E K
N T V T E Q P V I Q Q V E K V V Y V T R E V P V E N I I E K Q V V R K V V V E R I V E Q V R E V I K
E V P V E R I V E Q V V E V V K E V P I E K V V E R I Q I K E I P V V E T E E R V V E V V R E V P V
E I V K E V P V Y V K V D A A E W D A R H G L T H K S R A Q Y G G V G M L L G K F E G N H D R A G N
I Y V L E L V P G T P A E Q C H L I S L Q D I L I S V E G E V V H G M H L S T V H Q K V R G P E G T
P V V L E F V R G S S G E R Y T V T L Y R M A A V S E G N A V Y Q H S N N V K N I R V V S A G F S P
T T T R G T Y D F Y S Q G E P N G S G Y M A N E Q K E R A Y L

13

Chroomonas MMETSP0047_c37846_g1

Articulin

...AGDMSGHQDAGSGGYQVRTFPQLLDKLSPAVSPLPHSIQKSPPGGYEEA
YRSALSKSPSSPPK**G**DTQDVQQHSQQQAAEAPVQRSLMQQMSPYASQQA
AQQLPHYTTTTQSSAR

DRSGEVPV
ERIIF
KEIPVPV
EKIVY
KEVPTPYEVPGPIV
ERI
REIPV
DRVVLQEHAVPV
DRIV
KEDVPVHI
ERLVT
KEIPVEVPV
DKIVFQEVPVQVEKYV
EKHVEVPL
ERVFT
KEVPVEVPVMIQKVEI
KEV
EKVVYQDVV
KEVI
KEVPV
DRIVEVPV
DRVVEVPV
EKLIEVPV
ERVV
ERLVEIPV
DKII
EKPVPVHV
DRFV
DRPLQVKTVEVEV
EKII
ERVVEVPV
DKVVI
KEVP

*Chroomonas*_MMETSP0047_c341_g1

Articulin. Predicted secondary structure next slide

…
REVI
KEVPVI
KEIEVPRPYEVKVPYEVT
REVPVEVI
REVT
REVPVEVI
REVPV
EKIVYQEIAVPV
EKVVV
KEVTVPV
EKPVEVIVH
KEVPV
EKIVI
KEVPVPV
EKVVV
KEVQVPV
DRIV
EREVF
REIPI
ERVVV
KEVPVTV
EKVVT
KEVPVPYETVVT
KERTVEVPV
DRIV
DRERIVYQEVPV
EKIVV
KEVPVEV
EKII
ERIVYQEVEVPVEIKVPVTV
EKII
EKEVV
REVQVI
KEVAVPTETLV
EKVVI
KEVPV
ERVVY

KDIQHECNVCGRKDMELREYSGLKTRLEAEIRRLTRTLEEAEGRNSNISHNEVEISQLRSERSR
LEQEIRRLTALLDEAGRRASSSSASKERESQQLRDALQRAEAEIRRLQALLEAERSRGPEVVYT
ESPQQVVEKVVYRESPPQVVEKIVYRDSPQKVMPVQQMPVARHSPPPELKKRVGLGLALERSSD
DRTTYVSEIIPQFAAAKSGQFQLGDVVMAIDQEPVEGYELDAIKQLTIGEEGSFCTLQMLRGNQ
YYAVTLQRIAPAHLDDSNYEAAMRVMDQRSFNSTASLSPRSTRMSGSYGQPY

15

*Chroomonas*_MMETSP0047_c341_g1

Predicted secondary structure

1 R E V I K E V P V I K E I E V P R P Y E V K V P Y E V T R E V P V E V I R E V T R E V P V E V I R E
51 V P V E K I V Y Q E I A V P V E K V V V K E V T V P V E K P V E V I V H K E V P V E K I V I K E V P
101 V P V E K V V V K E V Q V P V D R I V E R E V F R E I P I E R V V V K E V P V T V E K V V T K E V P
151 V P Y E T V V T K E R T V E V P V D R I V D R E R I V Y Q E V P V E K I V V K E V P V E V E K I I E
201 R I V Y Q E V E V P V E I K V P V T V E K I I E K E V V R E V Q V I K E V A V P T E T L V E K V V I
251 K E V P V E R V V Y K D I Q H E C N V C G R K D M E L R E Y S G L K T R L E A E I R R L T R T L E E
301 A E G R N S N I S H N E V E I S Q L R S E R S R L E Q E I R R L T A L L D E A G R R A S S S S A S K
351 E R E S Q Q L R D A L Q R A E A E I R R L Q A L L E A E R S R G P E V V Y T E S P Q Q V V E K V V Y
401 R E S P P Q V V E K I V Y R D S P Q K V M P V Q Q M P V A R H S P P P E L K K R V G L G L A L E R S
451 S D D R T T Y V S E I I P Q F A A A K S G Q F Q L G D V V M A I D Q E P V E G Y E L D A I K Q L T I
501 G E E G S F C T L Q M L R G N Q Y Y A V T L Q R I A P A H L D D S N Y E A A M R V M D Q R S F N S T
551 A S L S P R S T R M S G S Y G Q P Y

16

*Chroomonas*_MMETSP0047_c3210_g1

Articulin

…GYQLQEVQQMQMIPVQQQQLVEVQQVQMVEQIPVQQVQMVEYQQPVVTQVQ
EVIHYSPPPMVEPPPPPPPPPPKPRPPPAPRPPNVRIQEQEVNVPI

DRIIEVPT
EKYI
DRYVEVPV
DKVVY
KDVVYEVA
REVI
KEVPQPV
EKIQY
REVPVPVEVVV
EKVV
ERVVL
KEVPY
EKVI
EKQVPYTV
DKIVH
KEVPVYV
DKIV
ERVVA
REVPV
ERIVTMEVPVEV
EKVIV
KEVPVPF
DRIV
DREIEVPI
ERIVYN
DKIVYQ
DKIVYQDRMVEV

TSSREVNSELGHIGGYEKFEGAFQGQQMMQTGYTTTGYTTVGYGSMAGRKVGLGLL
LKTNASNQVFIKETIKGFAAAVQGSVMPNDVIVAVDGKTVEGWDLDSIKQLTFGEEGSV
CTLTLRRGNDHFQVSLTRILPTGGDAEMARSLTRDY

17

Chroomonas _MMETSP0047_c8081_g1

Articulin

…GGQRFSSPMTPTEYLQHKGLSPRQHAHTHHTHSGTVVSQRHSGSSFAHSKP
MHYHHTHGSHLVHHDRVHYQHLVHPMEIREVHPVEVREVRPVEMHHVQHSPG
PSPVFVAPAVPVYNTVAIQ

ERVVTQEVPVVVQ
DRIVT
KEVTVPH
DRIVEVPV
EKVVF
RDVP
RDIPVEI
EKVVY
RDVPV
DRVV
EREVPV
ERVVT
KEVPV
ERVV
ERVVT
KEVP
RDV
ERVVEV
ERRVEVPVEV
EKLVEVV
KEVT
REVPV
ERRVEVPV
DRVVEVPV
DRVV
ERVLQ

APNSPPAPTGEVVGEFATVLDIVTTNVGPLRPVDDPLVYRNAEELRRMCHDCERC
EKCRNNIPHTGNSIRGSVSPMRQSFNAERPSYGGYAERASYSGQASATHSPVVTA
SASTEHRAASLSIDQNVAASPSVPKQVEEVGVGLRITQDLGTDSLFYVEEIIAGYAAA
RSGQFRVGDQVLSIQGRRITHIDEITGLTVGPSGTRCRISVRREGQQPFDIELIRSAP
GDIVASPYYANAEPSPKAGGYRASTTYSAMSARTTRQMDSEDENDYV

18

*Chroomonas*_MMETSP0047_c46070_g1

Articulin

...PPPPPPIEQIIYQQAPQPPPQI

RERVVEV
EKLVEVPV
DRIVEVPV
DRIV
DRYVEVPV
EKVTEQM
REVPV
DRVIT
KEVPVEI
ERLV
EKVVV
KEVPVPV
EKIV
EKIVEVPV
ERIVYQ
DRIVYQDQVVYQ
DRVVEVPV
ERVVQY

NQPREGPAIATGMAFVREYSPQNITTMTGNMYRSQSRVLSSVGLGLLLRKND
QNMTCIKEVFSGYAAARSGKVHPGDVIMSVDGRQVDNMDLEYIKNLTIGEEG
SMCTLTVKRGNEVVDLPLQRINPEGGMSDGRAGEVYRSMSRQSYGGGEVHY
SGGGGAPFMSSPQHVYGAPMYMQ

Goniomonas >CAMPEP_0175813662

Articulin. VYV motifs

...KVIYI

DREVPVT
REVPV
ERVV
EKEV
EKVV
ERPVYIEVPV
DREVFV
EREVPV
ERIV
EKEV
EKEVKVFV
DRPVYV
DREV
EKVV
EKRVEVPV
ERVVQV
DRPVYV
EREVPYVEPPRVM

SHRSEV**GG**VGLVLRDDSNQRDFEIERMLPNSPAERCGQIRVGDKVIGVDTYSVENRAL
SLVKEYIRGNVGTEVTLHCRRPTLFGGAGEIFSVTLRRDHEPEVASEYPQEIV
STIVPPNYASFQNSTILRASNPTYASNIIPLSSLSSSEPVSPLDDRX

1 K V I Y I D R E V P V T R E V P V E R V V E K E V E K V V E R P V Y I E V P V D R E V F V E R E V P
51 V E R I V E K E V E K E V K V F V D R P V Y V D R E K V V E K R V E V P V E R V V Q V D R P V Y V E
101 R E V P Y V E P P R V M S H R S E V G G V G L V L R D D S N Q R D F E I E R M L P N S P A E R C G Q
151 I R V G D K V I G V D T Y S V E N R A L S L V K E Y I R G N V G T E V T L H C R R P T L F G G A G E
201 I F S V T L R R D H E P E V A S E Y P Q E I V S T I V P P N Y A S F Q N S T I L R A S N P T Y A S N
251 I I P L S S L S S S E P V S P L D D R X

20

Goniomonas >CAMPEP_0175818310

VYV family. This and several other genes are either orthologues or splice variants. See slide 4.26 for alignments.

…DDLQRLVDDPRLHDNRRDLAGVGIVFVEGLDQKRHIAVMTLVRHSPAERDGTIQ
VGDVIVEIDGQSVRGFRLDIIRGMILGAPGTTVTLGFHRHHASPHRHQHASPAGRS
SLIYATLTREADPSDHLKGRLVTMEREMLSYREQYEFERETRTTVETRFSNLQDQL
LASQQEITKYQEELLSERSARERVEMELRQALGKERATRASLESRVESLTAQLNEQ
AVRERLEAELRELQGAAGKVSTLEQRVSMLQQQLRVAQEELTQERLARERMERD
HAEQTALFERMELRLRQSQEEVVQQRSGRERAEGDLENVAGELRQATASYQTM
ERQVNEERQERSQLQSDYERLISQTESEVQRRERLEIRIRELTTELDQMHDLKQAL
SESRNKVSSLQSKLRSLQDGAEAAQAEIARERRERERLKRWNALXTQERSIRQALE
XEMRSLGGRFEDLLXENNGLREQLRRAESEAAQLPELQKTIRELEAEIQELRQRNE
AL

EREF
ERVQDFLRRQGPP
KDRVVYV
DRPVEVEV
ERIV
ERV
KEVPYEVVV
EKPV
EKTVYV
EKEV
ERPVYI
EKEVPRPVYV
EKEVPRPVYVQQEI

PPPPPPPPQEFEQVVSRHQGPEMGGIGLLLKDDKNGYEFEIERLIPGSPADRCGQIHV
GDAVIGVDSYSVRDRPLALVKEYIRGNVGSTVTIHLRRAGTRDEMFSLSLTRGHEPEV
ASQYPAPAVEEVVSIRPRSTHNSWRGTLXSTSNLISSSLDSRIVGQPLASRPIGNQVG
ASXVGYNSISTASPVHLSTSNLVTGPSLVSNVSGVRAPLSRVIG

21

Alignment of *Goniomonas* epiplastin family

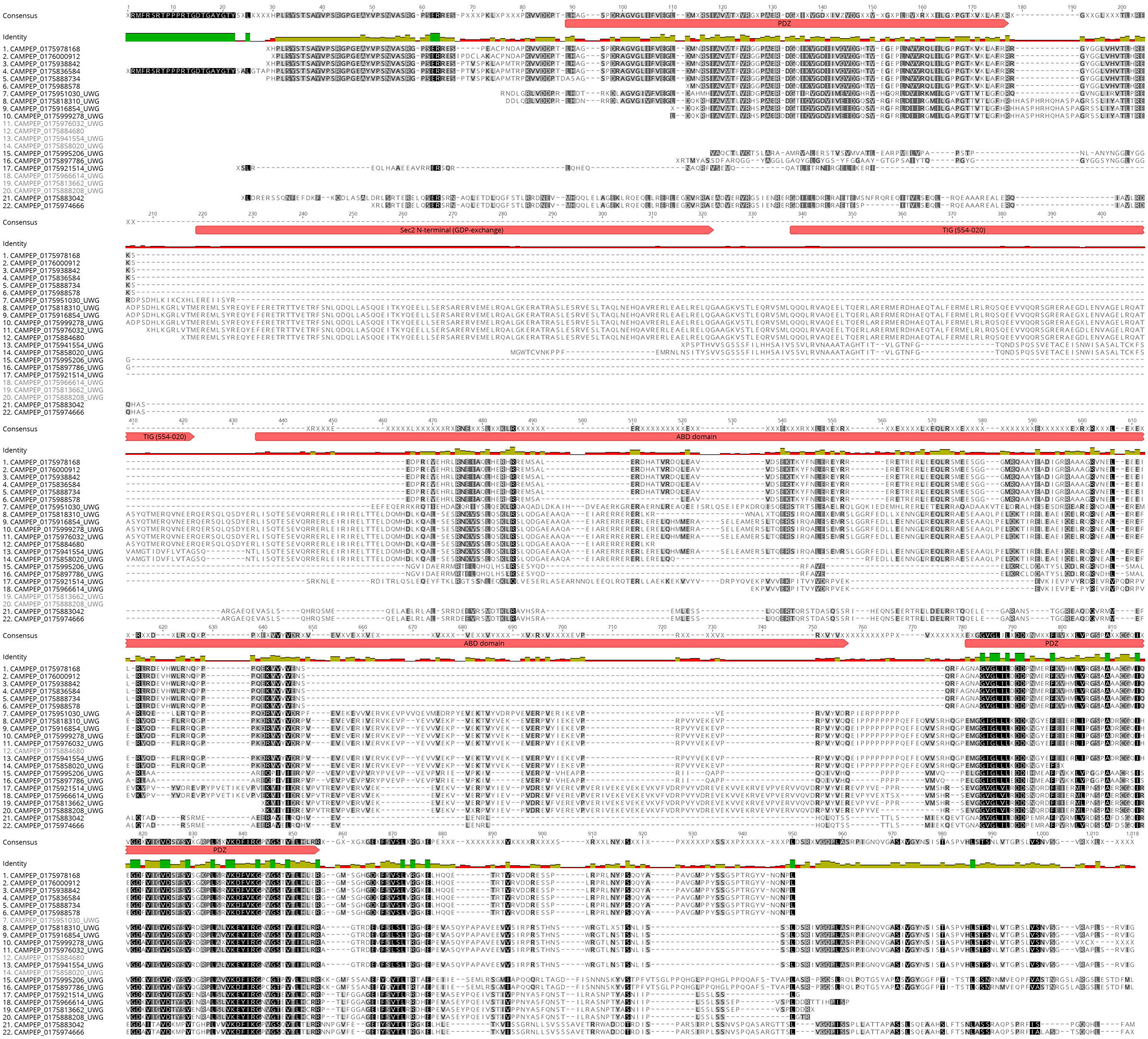

Goniomonas >CAMPEP_0175916854

VYV family. This and several other genes are either orthologues or splice variants. See slide 4.22 for alignments. Predicted secondary structure next slide

…DQKRHIAVMTLVRHSPAERDGTIQVGDVIVEIDGQSVRGFRLDIIRGMILGA
PGTTVTLGFHRHHASPHRHQHASPAGRSSLIYATLTREADPSDHLKGRLVTM
EREMLSYREQYEFERETRTTVETRFSNLQDQLLASQQEITKYQEELLSERSA
RERVEMELRQALGKERATRASLESRVESLTAQLNEHQAVRERLEAELRELQ
GAAGKVSTLEQRVSMLQQQLRVAQEELTQERLARERMERDHAEQTALFER
MELRLRQSQEEVVQQRSGRERAEGXLENVAGELRQATASYQTMERQVNE
ERQERSQLQSDYERLISQTESEVQRRERLEIRIRELTTELDQMHDLKQALSES
RNKVSSLQSKLRSLQDGAEAAQAEIARERRERERLERELQHMMERASELEAM
ERSLTQERSIRQALESEMRSLGGRFEDLLEENNGLREQLRRAESEAAQLPEL
QKTI

RELEAEIQELRQRNEAL
EREF
ERVQDFLRRQGPP
KDRVVYV
DRPVEVEV
ERIV
ERV
KEVPYEVVV
EKPV
EKTVYV
EKEVERPVYI
EKEVPRPVYV
EKEVPRPVYVQQEI

PPPPPPPPQEFEQVVSRHQGPEMGGIGLLLKDDKNGYEFEIERLIPGSPADRCGQ
IHVGDAVIGVDSYSVRDRPLAVVKEYIRGNVGSTVTIHLRRAGTRDEMFSLSLTRG
HEPEVASQYPAPAVEEVVSIRPRSTHNSWRGTLNSTSNLISSSLDSRIVGQPLASR
PIGNQVGASIVGYNSISTASPVHLSTSNLVTGPSLVSNVSGVRAPLSRVIG

23

Goniomonas >CAMPEP_0175916854

Predicted secondary structure

1 D Q K R H I A V M T L V R H S P A E R D G T I Q V G D V I V E I D G Q S V R G F R L D I I R G M I L
51 G A P G T T V T L G F H R H H A S P H R H Q H A S P A G R S S L I Y A T L T R E A D P S D H L K G R
101 L V T M E R E M L S Y R E Q Y E F E R E T R T T V E T R F S N L Q D Q L L A S Q Q E I T K Y Q E E L
151 L S E R S A R E R V E M E L R Q A L G K E R A T R A S L E S R V E S L T A Q L N E H Q A V R E R L E
201 A E L R E L Q G A A G K V S T L E Q R V S M L Q Q Q L R V A Q E E L T Q E R L A R E R M E R D H A E
251 Q T A L F E R M E L R L R Q S Q E E V V Q Q R S G R E R A E G X L E N V A G E L R Q A T A S Y Q T M
301 E R Q V N E E R Q E R S Q L Q S D Y E R L I S Q T E S E V Q R R E R L E I R I R E L T T E L D Q M H
351 D L K Q A L S E S R N K V S S L Q S K L R S L Q D G A E A A Q A E I A R E R R E R E R L E R E L Q H
401 M M E R A S E L E A M E R S L T Q E R S I R Q A L E S E M R S L G G R F E D L L E E N N G L R E Q L
451 R R A E S E A A Q L P E L Q K T I R E L E A E I Q E L R Q R N E A L E R E F E R V Q D F L R R Q G P
501 P K D R V V Y V D R P V E V E V E R I V E R V K E V P Y E V V V E K P V E K T V Y V E K E V E R P V
551 Y I E K E V P R P V Y V E K E V P R P V Y V Q Q E I P P P P P P P P Q E F E Q V V S R H Q G P E M G
601 G I G L L L K D D K N G Y E F E I E R L I P G S P A D R C G Q I H V G D A V I G V D S Y S V R D R P
651 L A V V K E Y I R G N V G S T V T I H L R R A G T R D E M F S L S L T R G H E P E V A S Q Y P A P A
701 V E E V V S I R P R S T H N S W R G T L N S T S N L I S S S L D S R I V G Q P L A S R P I G N Q V G
751 A S I V G Y N S I S T A S P V H L S T S N L V T G P S L V S N V S G V R A P L S R V I G

24

Goniomonas >CAMPEP_0175999278

VYV family. This and several other genes are either orthologues or variants. See slide 4.22 for alignments.

…LDQKRHIAVMTLVRHSPAERDGTIQVGXXDGTIQVGDVIVEIDGQSVRGFRL
DIIRGMILGAPGTTVTLGFHRHHASPHRHQHASPAGRSSLIYATLTREADPSDH
LKGRLVTMEREMLSYREQYEFERETRTTVETRFSNLQDQLLASQQEITKYQE
ELLSERSARERVEMELRQALGKERATRASLESRVESLTAQLNEHQAVRERLE
AELRELQGAAGKVSTLEQRVSMLQQQLRVAQEELTQERLARERMERDHAEQ
TALFERMELRLRQSQEEVVQQRSGRERAEGXLENVAGELRQATASYQTMER
QVNEERQERSQLQSDYERLISQTESEVQRRERLEIRIRELTTELDQMHDLK
QALSESRNKVSSLQSKLRSLQDGAEAAQAEIARERRERERLERELQHMMERA
SELEAMERSLTQERSIRQALESEMRSLGGRFEDLLEENNGL

REQLRRAESEAAQLPELQKTI
RELEAEIQELRQRNEAL
EREF
ERVQDFLRRQGPP
KDRVVYV
DRPVEVEV
ERIV
ERV
KEVPYEVVV
EKPV
EKTVYV
EKEV
ERPVYI
EKEVPRPVYV
EKEVPRPVYVQQEI

PPPPPPPPQEFEQVVSRHQGPEMGGIGLLLKDDKNGYEFEIERLIPGSPADRC
GQIHVGDAVIGVDSYSVRDRPLAXVKEYIRGNVGSTVTIHLRRAGTRDEMFSLS
LTRGHEPEVASQYPAPAVEEVVSIRPRSTHNSWRGTLNSTSNLISSSLDSRIVG
QPLASRPIGNQVGASIVGYNSISTASPVHLSTSNLVTGPSLVSNVSGVXC....

25

Goniomonas >CAMPEP_0175941554

VYV family. This and several other genes are either orthologues or splice variants. See slide 4.22 for alignments.

…PSPTHVVSGSSSFILHHSAIVSSVLRVNAAATAGHTITVLGTNFGTQNDS
PQSSVETACEISNWISASALTCKFSVAMGTIDVFLVTAGSQNTLISQTES
EVQRRERLEIRIRELTTELDQMHDLKQALSESRNKVSSLQSKLRSLQDGAE
AAQAEIARERRERERLERELQHMMERASELEAMERSLTQERSIRQALESE
MRSLGGRFEDLLEENNGL

REQLRRAESEAAQLPELQKTI
RELEAEIQELRQRNEAL
EREF
ERVQDFLRRQGPP
KDRVVYV
DRPVEVEV
ERIV
ERV
KEVPYEVVV
EKPV
EKTVYV
EKEV
ERPVYI
EKEVPRPVYV
EKEVPRPVYVQQEI

PPPPPPPPQEFEQVVSRHQGPEMGGIGLLLKDDKNGYEFEIERLIPGSPA
DRCGQIHVGDAVIGVDSYSVRDRPLALVKEYIRGNVGSTVTIHLRRAGTRD
EMFSLSLTRGHEPEVASQYPAPAVEEVVSIRPRSTHNSWRGTLNSTSNLI
SSSLDSRIVGQPLASRPIGNQVGASIVGYNSISTASPVHLSTSNLVTGPSLV
SNVSGV*RAPLSRVIG

26

Goniomonas >CAMPEP_0175966614

Articulin. VYV motifs

…
EKPVVV
EKPITVYV
DRPV
EKEVKIEVPEPY
REVRVPQ
DRPVEVKVPVYV
DREVPYPVETI
KEVPV
EKVIYI
DREVPVT
REVPV
ERVV
EKEV
EKVV
ERPVYIEVPV
DREVFV
EREVPV
ERIV
EKEV
EKEVKVFV
DRPVYV
DREV
EKVV
EKRVEVPV
ERVVQV
DRPVYV
EREVPYVEX

TSXVMSHRSEVGGVGLVLRDDSNQRDFEIERMLPNSPAERCGQIRVGDKVIGVD
TYSVENRALSLVKEYIRGNVGTEVTLHCRRPTLFGGAGEIFSVTLRRDHEPEVAS
EYPQEIVSTIVPPNYASFQNSTILRASNPTYASNIIPLSSLSSSEPVSPLDDRTTIHP
ISP

27

Goniomonas >CAMPEP_0175951030

VYV motifs

…RNDLGRLVDDPRLHDTRKDLAGVGIVFVEGLDAHMHIAVMTLVRDGPAERD
GTIRVGDVIMEVDGHRVHGQRLDVIRKMILGPVGTTVTLGFDRQGVNGLIRVTL
PR

ERDPSDHLKIKCXHL
EREIISY
REEFEQ
ERRKRQTIEHDAKQRIYSLQEQLRQAQADL
DKAIHDVEA
ERKGR
ERA
ERNL
REAQEEISRLQSEIEP
KDRQISQ
ERSTRTSLEAELRQLGQKIEDEMHL
RERLETELRRAQADAAKVTEL
DRALHESES
DRREAR
ERNDAL
EREMARLQELLRTQPPPQ
DRVVYV
DRPFEV
EKEVVV
ERV
KEVPVVQEVMI
DRPYEV
EKTVYV
DRPVEV
ERPV
ERI
KEVPV
ERVVYV
DRPI
ERPPPPPP

```
  1 R N D L G R L V D D P R L H D T R K D L A G V G I V F V E G L D A H M H I A V M T L V R D G P A E R
 51 D G T I R V G D V I M E V D G H R V H G Q R L D V I R K M I L G P V G T T V T L G F D R Q G V N G L
101 I R V T L P R E R D P S D H L K I K C X H L E R E I I S Y R E E F E Q E R R K R Q T I E H D A K Q R
151 I Y S L Q E Q L R Q A Q A D L D K A I H D V E A E R K G R E R A E R N L R E A Q E E I S R L Q S E I
201 E P K D R Q I S Q E R S T R T S L E A E L R Q L G Q K I E D E M H L R E R L E T E L R R A Q A D A A
251 K V T E L D R A L H E S E S D R R E A R E R N D A L E R E M A R L Q E L L R T Q P P P Q D R V V Y V
301 D R P F E V E K E V V V E R V K E V P V V Q E V M I D R P Y E V E K T V Y V D R P V E V E R P V E R
351 I K E V P V E R V V Y V D R P I E R P P P P P P
```

Goniomonas >CAMPEP_0175921514

Articulin. VYV motifs. Predicted secondary structure next slide

...SLREQLHAAEEAVRRERSQRLQHEQNAQRFVSEMQQATLETRNIRGEL
EKERISRKNLERDITRLQSLEQEYFTKLRGTSSNLEQKLQLVESERLASEA
RNNQLEEQLRQTERLLA

EKK
EKVVYV
DRPYQV
EKPVVV
EKPITVYV
DRPV
EKEVKIEVPVY
RDREVRVPQ
DRPVEVKVPVYV
DREVPYPVETI
KEVPV
EKVIYI
DREVPVT
REVPV
ERVV
EKEV
EKVV
ERPVYIEVPV
DREVFV
EREVPV
ERIV
EKEV
EKEVKVFV
DRPVYV
DREV
EKVV
EKRVEVPV
ERVVQV
DRPVYV
EREVPYVEPPRVM

SHRSEVGGVGLVLRDDSNQRDFEIERMLPNSPAERCGQIRVGDKVIGVDTYS
VENRALSLVKEYIRGNVGTEVTLHCRRPTLFGGAGEIFSVTLRRDHEPEVASE
YPQEIVSTIVPPNYASFQNSTILRASNPTYASNIIPLSSLSSLG

29

Goniomonas >CAMPEP_0175921514

Predicted secondary structure

```
  1 S L R E Q L H A A E E A V R R E R S Q R L Q H E Q N A Q R F V S E M Q Q A T L E T R N I R G E L E K
 51 E R I S R K N L E R D I T R L Q S L E E Y F T K L R G T S S N L E Q K L Q L V E S E R L A S E A R N
101 N Q L E E Q L R Q T E R L L A E K K E K V V Y V D R P Y Q V E K P V V V E K P I T V Y V D R P V E K
151 E V K I E V P V Y R D R E V R V P Q D R P V E V K V P V Y V D R E V P Y P V E T I K E V P V E K V I
201 Y I D R E V P V T R E V P V E R V V E K E V E K V V E R P V Y I E V P V D R E V F V E R E V P V E R
251 I V E K E V E K E V K V F V D R P V Y V D R E V E K V V E K R V E V P V E R V V Q V D R P V Y V E R
301 E V P Y V E P P R V M S H R S E V G G V G L V L R D D S N Q R D F E I E R M L P N S P A E R C G Q I
351 R V G D K V I G V D T Y S V E N R A L S L V K E Y I R G N V G T E V T L H C R R P T L F G G A G E I
401 F S V T L R R D H E P E V A S E Y P Q E I V S T I V P P N Y A S F Q N S T I L R A S N P T Y A S N I
451 I P L S S L S S L G
```

30

Goniomonas >CAMPEP_0175945020

Articulin

…TLVVRHTPLNMSTS**G**ENLSSRDPAYDARTDWHNQKVLEQPFVEYQNVE
YEEPSVETQRKTITNVETLEVPTTVTELVSRSMTRNI

EKPVTRMQVTEVEVPETKSFVKTIEVPVVSTQRVKV
DRIVQRT
DKRTIQVPEAYTKTVQIDEPQTRVV
KEEIEVPETTMRNVTVEDVVTRTVKVQARAPA
EKRVLVDVEVPVTEIQKRVIEEPVTRYRT
KEVEVPETRI
ERRIVETPVTTVVRRQIEVE
ERTRMLDVTENELXPATGESRLSRRSFNDEGTVPQSLGTIRTRTVDEPVTR
MIQQEVEEPVVTMVKKAIQEPYIEMVP
REVEEPETRVTIKQE
ERKQLVMEP
REWDVPESTVVPRQVSVPVSRIIKQVVEVEVTDFVPRTIEVQLTRMVA
REVEVVTTEVVPEEVEVPLVRMVSKQVECQETTFRRAQVETPVTEMVPQQ
VTYTVT
ERVPKRVTMPQTKQTS
REIDFQTTKMLRKMAQVPV
EKRVRKVVEQ
RERVV

GVQELWPDNHNAAAASGRGSALDTRGSYA

31

Goniomonas >CAMPEP_0175932590

Articulin. Predicted secondary structure next slide

…QYGQQPHGQQPYGQQAYGQQAYGQQYGQYGRTQYGMQ
TAQVNVPVV

REQVPV
ERTILH
RENVPVPRQVPVPVPVQ
REVHHTQRVEVPVEVPRMVV
RDRVIDQPVETPVPRPVPVEVVKPV
ERPYQVPVQ
RDVIHNQVVEVPVEVP
REYYTDVVRTQPVEVPYEVAVPV
DRFV
EKEVPIEQT
REVIRNQIVEVPVEVPVEVT
KDVEVIQPVEVPVPV
EKPYPV
DREVIVEVPVPV
EKEVIRW
ERVEIP
REIQIPI
DKEVVRW
ERVEIPIEVPVHTEVI
KEVP
REIP
REVL
KEVI
KEVPIEIVRWERVEVPTEVP
REVVTEVV
REVPKIIEVPVEVPHEVVRYQRVEVPIEVEVP
REVVRWEND
DREILRL
REDYRLLEQRYHNL

ARAYEELKRVEQRTFTREKVIEVAQVRQVTRETASQRRASPPRR
APAPRQSTSPPRQTTSPPRRPAAMASSSSSSSSSSSSSSPRNN
SSAPRNNLVSNNNAQDYNAYEV

32

Goniomonas >CAMPEP_0175932590

Predicted secondary structure

```
  1 Q Y G Q Q P H G Q Q P Y G Q Q A Y G Q Q A Y G Q Q Y G Q Y G R T Q Y G M Q T A Q V N V P V V R E Q V
 51 P V E R T I L H R E N V P V P R Q V P V P V P V Q R E V H H T Q R V E V P V E V P R M V V R D R V I
101 D Q P V E T P V P R P V P V E V V K P V E R P Y Q V P V Q R D V I H N Q V V E V P V E V P R E Y Y T
151 D V V R T Q P V E V P Y E V A V P V D R F V E K E V P I E Q T R E V I R N Q I V E V P V E V P V E V
201 T K D V E V I Q P V E V P V P V E K P Y P V D R E V I V E V P V P V E K E V I R W E R V E I P R E I
251 Q I P I D K E V V R W E R V E I P I E V P V H T E V I K E V P R E I P R E V L K E V I K E V P I E I
301 V R W E R V E V P T E V P R E V V T E V V R E V P K I I E V P V E V P H E V V R Y Q R V E V P I E V
351 E V P R E V V R W E N D D R E I L R L R E D Y R L L E Q R Y H N L A R A Y E E L K R V E Q R T F T R
401 E K V I E V A Q V R Q V T R E T A S Q R R A S P P R R A P A P R Q S T S P P R Q T T S P P R R P A A
451 M A S S S S S S S S S S S S S S S P R N N S S A P R N N L V S N N N A Q D Y N A Y E V
```

33

Goniomonas >CAMPEP_0175932456

…IAMPVNSSASFVSGDNWTGQRVVEQPYTENQVVEYEEPDVVMGTQSVQ
NIETVQV**G**ATIHELVATQKQRTI

EKPVTRMHITEVEMPENRTFVKTIEVPVVTTQRVQV
ERPVQKV
EKKIIEVPEAYTQSVTIEEPVTRIVQEEIEVPDTVMRTATVEDVVTRNVVVQAQ
TPA
ERSVSVDVEEPVTRMQRRIIEEPVTRFRT
REVEVPETTMQRRIVETPVTTLVRKQVEVE
ERVRVLDVSDGGDGRRYVDDQFGNDRNSRVLSTRTVDEPVTRMIQQEIEEP
VVTMVRKTIEEPVTEVIQ
REIEEPITSSVMRQE
ERRQVVMEP
REYEVPESTVVARQVSVPVSRLVRQVVDVEVTEFVPRTIEVQLTRMVQ
REVEIVTTDFVTEEVEVPLVRMVP
KEIECQETQFRIAQVETPVSELVPHVVNYTVT
ERVPKQVTMPQTKQVSRQVDFETTKMVRKVANLPV
ERRVRKVVEQ
RERVV

GVQELWPEQDGAVSALRASQGGRGSWA

34

Goniomonas >CAMPEP_0175825140

...RAVDDDAIVSRVVTDI**G**VDTVRTTRPVVVDSSMVETRTVEYQEPATEVQRQT
LRDFESVDVEVEVQELQTRTESREVQAPVTTIKIVECXIPETRTYYKTIEEPVVST
QQVAVLEPVQKTYTQRVAKPVARTKVVDVYEPVTKVETFEVDVIETSHQPATVE
EIVTKLVEIETEAPLMQRQLVD

REVPVTQRVRRTV
REPVTRVI
EREVEVPQTMMQRRVVE
ERTRLVDEQGRYIDS
RDGSDRIVQ
REYDTPITTMTRQVVQEPYEEIV
EREIEEDVQSVEIQQQEVQTIG
RERRVFQVPESSVVTRQIEAPVSRIIRKAVEVETTDFVQRQVEVTEVEMQYEEVQC
VKTEFVESLHEVPLVRLVQKQVEYQQTDMRVSEIECPHTELVP
KEYVYSVTNKVNRTVNLPQT
KELT
KEVDVRSTKFVTKSYDVPV
ERRVRKVVEQR
ERIVEEVPVSY

GYTSQPADVQYVRSSRYS

35

Goniomonas >CAMPEP_0175897786

Articulin. Only example of tandem VPV units

...RTMYASSDFARQGGYAGGLGAQYGLGYGSYFGGAAYGTGPSAIYTQPG
YGGYGGSYNGGLYGGGN**G**VIDA

ERRMRTELQHQLHSLRSESYQ
DRFAVEELKRCLDDATYSLQDLRGRNDHLSMALARLAAA
REQPIVI
ERPVPVEVPVEVPVRYPVEVPVEVPVRIEVPKIVEV
ERPVVHEAPPRIIQAPPQQIVVHQPPPPVMVQPEL

GGIGLLLEDDIHMEAFPVKKLVPGGPAAACRSISVGDCVIGVDVYSVQGRPLA
VVKDFIRGPCGTPVVLHLRRKKGMFSSANEFYQVTLTRTAEPEIIESEMLRSG
MIAPQQQRLTAGDFISNNNSKYVSTPFVTSGLPPQHGLPPQHGLPPQQAFST
VAPLASRPGQSLRQLPQTGSYAPASVGYGGFPTITSTLGSNRNMVEQPFVAS
TNRGSLARGSLESTDFML

36

Goniomonas >CAMPEP_0175989294

Articulin. The tail domains of 4.37-4.40 are homologous.

…QYGQQPHGQQPYGQQAYGQQAYGQQYGQYGRTQYGMQTAQVNVPVV

REQVPV
ERTILH
RENVPVPRQVPVPVPVQ
REVHHTQRVEVPVEVPRMVV
RDRVIDQPVETPVPRPVPVEVVKPV
ERPYQVPVQ
RDVIHNQVVEVPVEVP
REYYTDVVRTQPVEVPYEVAVPV
DRFV
EKEVPIEQT
REVIRNQIVEVPVEVPVEVT
KDVEVIQPVEVPVPV
EKPYPV
DREVIVEVPVPV
EKEVIRW
ERVEIP
REIQIPI
DKEVVRW
ERVEIPIEVPVHTEVI
KEVP
REIP
REVL
KEVI
KEVPIEIVRW
ERVEVPTEVP
REVVTEVV
REVPKIIEVPVEVPHEVVRYQRVEVPIEVEVP
REVVRWENDDRXDP

SAPXXLXAXEXXKRVEQRTFTREKVIEVAQVRQVTRETASQRRASPPRRAPAPR
QSTSPPRQTTSPPRRPAAMASSSSSSSSSSSSSSSPRNNSSAPRNNLVSNNNA
QDYNAYEV

37

Goniomonas >CAMPEP_0175998318

Articulin. The tail domains of 4.37-4.40 are homologous.

…QRDVIHNQVVEVPVEVP

REYYTDVVRTQPVEVPYEVAVPV
DRFV
EKEVPIEQT
REVIRNQIVEVPVEVPVEVT
KDVEVIQPVEVPVPV
EKPYPV
DREVIVEVPVPV
EKEVIRW
ERVEIP
REIQIPI
DKEVVRW
ERVEIPIEVPVHTEVI
KEVP
REIP
REVL
KEVI
KEVPIEIVRW
ERVEVPTEVP
REVVTEVV
REVPKIIEVPVEVPHEVVRYQRVEVPIEVEVP
REVVRWEND
DREILRL
REDYR

LLEQRYHNLARAYEELKRVEQRTFTREKVIEVAQVRQVTRETASQRRASPPR
RAPAPRQSTSPPRQTTSPPRRPAAMASSSSSSSSSSSSSSPRNNSSAPRN
NLVSNNNAQDYNAYEV

38

Goniomonas >CAMPEP_0175962262

Articulin. The tail domains of 4.37-4.40 are homologous.

…EVPYEVAVPV

DRFV
EKEVPIEQT
REVIRNQIVEVPVEVPVEVT
KDVEVIQPVEVPVPV
EKPYPV
DREVIVEVPVPV
EKEVIRW
ERVEIP
REIQIPI
DKEVVRW
ERVEIPIEVPVHTEVI
KEVP
REIP
REVL
KEVI
KEVPIEIVRW
ERVEVPTEVP
REVVTEVV
REVPKIIEVPVEVPHEVVRYQRVEVPIEVEVP
REVVRWEND
DREILRL
REDYR

LLEQRYHNLARAYEELKRVEQRTFTREKVIEVAQVRQVTRETASQRRASPP
RRAPAPRQSTSPPRQTTSPPRRPAAMASSSSSSSSSSSSSSPRNNSSAP
RNNLVSNNNAQDYNAYEV

Goniomonas >CAMPEP_0175844352

Articulin. The tail domains of 4.37-4.40 are homologous.

…QT

REVIRNQIVEVPVEVPVEVT
KDVEVIQPVEVPVPV
EKPYPV
DREVIVEVPVPV
EKEVIRW
ERVEIP
REIQIPI
DKEVVRW
ERVEIPIEVPVHTEVI
KEVP
REIP
REVL
KEVI
KEVPIEIVRW
ERVEVPTEVP
REVVTEVV
REVPKIIEVPVEVPHEVVRYQRVEVPIEVEVP
REVVRWEND

DREILRLREDYRLLEQRYHNLARAYEELKRVEQRTFTREKVIEVAQVRQVTRET
ASQRRASPPRRAPAPRQSTSPPRQTTSPPRRPAAMASSSSSSSSSSSSSSP
RNNSSAPRNNLVSNNNAQDYNAYEV

40

Goniomonas >CAMPEP_0175835782

Articulin

…RRLVDARMSGYGNYGNYASNRATGYGAGYGSNYASGYGANYATQYGA
ATQYGAAASAYARTGSVVGERIGQPT

REVMHQTIGVQVPVV
REQVPV
ERHIVH
REPVEVPVPVEVPVPVQ
RDVVHQQRVEVPVEVPVEVP
RDVVRYQKVPVEVPVPYPV
ERVQEVF
REVPVEVT
RDVVHTQRVEVPVEVPVEVP
REVVRYQPVEVPVEVPV
ERI
KEVF
REVPVEVT
RDVVHTQRXEVPVEVPVEVP
REVVRYQYVEVPVEVPVPV
ERIQEVI
KEVPIEIP
REVVRYQPVEVPVEVPIEVP
REVVRYQYVEVPVEVPVV
KEVI
KEVPVEVPYEVI
KEVI
KEVPYEVVRYEQVEVPVEVP
REII
KEVI
KEIPVEVPVEVPVEVVRYQKVEVPVEVPIEVP
REVVKWRTD
DRELQRL
REEYYMLE
ERYRKLR
RDYEELYRVKQRV
REV
EKVVEVPKVV
EKVVEV
DRPQPVVQQPQM

SYQVVSQPVVQSRLVTQPVYTSNLVSSPVRYTTGYTSGIANQGYTTGYTTGYQ
QYPVTGYTTGAVATSGIATSGYGATTGYGVTTGYTSGYGAGGMYASGINTGSP
VRPGSSRGAVYA

41

Guillardia 1|147041|f|71|-0.32887323943662|-0.145774647887324

Articulin

MAEAAGKNLLDEMLEEATEAQKNSAFGAGSGYSQEQLSHASPSECMAADNYG
LRPPSLTGNSEFFTCKYSCGFSGEFEAVDAHERQGDCVQRVVASESAAEGYVM
SPQSGTFGGLFSDSFTVDRSFTSYVTRMPQLPPKPCTDCSYAKAQSEALSKRLE
DLINAKYQNQIIV

KEILVPVPQVKVV
ERVV
DRYIEVPV
ERIV
KEMIEVPVPV
EKIVI
REVPV
EKIV
EKVIV
KEV
EKIV

ERIGLGLGLEETVDGRIYVADIVEGFAASRNGQIRVNDTIIGIDGMDVIANGLNLKTLIN
HTLGPEGSSCSLSLSRDGYQFTVKLTRVDGSQMLRSPDTSIYGPNSCTMDATPHTH
LFSCSNSGSPQQPETFFTTLPPILSQESVHACEWSSSGWMVGLLRGCEHGEQGAG
AAGQHDGFLVL

1 M A E A A G K N L L D E M L E E A T E A Q K N S A F G A G S G Y S Q E Q L S H A S P S E C M A A D N
51 Y G L R P P S L T G N S E F F T C Y S C G F S G E F E A V D A H E R Q G D C V Q R V V A S E S A A E
101 G Y V M S P Q S G T F G G L F S D S F T V D R S F T S Y V T R M P Q L P P K P C T D C S Y A K A Q S
151 E A L S K R L E D L I N A K Y Q N Q I I V K E I L V P V P Q V K V V E R V V D R Y I E V P V E R I V
201 K E M I E V P V P V E K I V I R E V P V E K I V E K V I V K E V E K I V E R I G L G L G L E E T V D
251 G R I Y V A D I V E G F A A S R N G Q I R V N D T I I G I D G M D V I A N G L N L K T L I N H T L G
301 P E G S S C S L S L S R D G Y Q F T V K L T R V D G S Q M L R S P D T S I Y G P N S C T M D A T P H
351 T H L F S C S N S G S P Q Q P E T F F T T L P P I L S Q E S V H A C E W S S S G W M V G L L R G C E
401 H G E Q G A G A A G Q H D G F L V L

42

Guillardia <135760

Predicted transmembrane domain highlighted in yellow.

MCGTVSCHKDPGHPQCYCDPGYTGPVGSKNCTICPAGSFKPSSGPQACSVCT
NKPANAVYLQESLPVSSNCEWTCAAGYYHDMGSCLPCEAGTFSVQGSSSCTV
CPDGSYSTPHSSSCSPCPQGTFSLSGFGTCFIAIPV

EKVIYI
EKPVYI
EKIVEIP
KEIQIQQTYQYNVNVQTQPANQTNTTVNINGEDCQAEIRYMDG
KEYVTC
KEIMCQAPQTNVTLNINGEDCQGEIRYMDG
KEYVTC
KEIMCQAPQ

NQSNVTVNINGEDCQAEIIFNHGRLFVTCKPHLNSFLAVNLFGIADALCRYFQSFPD
FDTSDISYKTLCSNLSYYVSLDKMVFPKFMSDFTVLPQMAFLNRTVEIPKVIYINRTH
LVEKLIYYVNQSQDSTSSQLSPSSSGVTPNIQVQMQVTLPIALQSFDNAKRVQFRTS
VAATAAVDPSTVIINKVQAARRSSSNVQVGFSIVTATQTDARAVAGNLNLKNLNEQF
RRHGLPEASATTNPQITSFSASTSSGVPLSYTAMIAACALGGVLLAVIVFYCLSYSGV
RRRREKVAPRLSVVQYFNSDVSRQRRNSINERDFALAFSLARRDRSRSGVGEVQPN
IARKRSGSATAIEIGNDFVRVWNPEHPRRNSASTAQHPNAANITVEDLE

1 M C G T V S C H K D P G H P Q C Y C D P G Y T G P V G S K N C T I C P A G S F K P S S G P Q A C S V
51 C T N K P A N A V Y L Q E S L P V S N C E W T C A A G Y Y H D M G S C L P C E A G T F S V Q G S S S
101 C T V C P D G S Y S T P H S S S C S P C P Q G T F S L S G F G T C F I A I P V E K V I Y I E K P V Y
151 I E K I V E I P K E I Q I Q Q T Y Q Y N V N V Q T Q P A N Q T N T T V N I N G E D C Q A E I R Y M D
201 G K E Y V T C K E I M C Q A P Q T N V T L N I N G E D C Q G E I R Y M D G K E Y V T C K E I M C Q A
251 P Q N Q S N V T V N I N G E D C Q A E I I F N H G R L F V C K P H L N S F L A V N L F G I A D A L C
301 R Y F Q S F P D F D T S D I S Y K T L C S N L S Y Y V S L D K M V F P K F M S D F T V L P Q M A F L
351 R T V E I P K V I Y I N R T H L V E K L I Y Y V N Q S Q D S T S S Q L S P S S S G V T P N I Q V Q M
401 Q V T L P I A L Q S F D N A K R V Q F R T S V A A T A A V D P S T V I I N K V Q A A R R S S S N V Q
451 V G F S I V T A T Q T D A R A V A G N L N L K N L N E Q F R R H G L P E A S A T T N Q I T S F S A S
501 T S S G V P L S Y T A M I A A C A L G G V L L A V I V F Y C L S Y S G V R R R R E K V A P R L S V V
551 Q Y F N S D V S R Q R R N S N E R D F A L A F S L A R R D R S R S G V G E V Q P N I A R K R S G S A
601 T A I E I G N D F V R V W N P E H P R R N S A S T A Q H P N A A N I T V E D L E

43

Guillardia 117614

Articulin. Predicted transmembrane domain highlighted in yellow. VYV repeats in blue. Next 2 slides: alternative parsing based on VY, and predicted secondary structure.

MPCPAGQYSEKGSTSCVDCPAGTFSKGNSSVCTRCQEGYASEVGSSSCCKVI
VRNNTVEKSVYLNVTVEVPKFIKVKEQVPIEVQKKVYVDEESGKKYGE

DRIVYLNRTVEVPVIV
EKLYTLIVLLKKLCTCTKLQWIKRIKARKIMTTKTTNHMAKTHKLTMKKMEAIKKM
KKMTRMVVYVNNTIY
KDRIIDVPRNVYINRTVEVPTLVYVNRTVEVPTVVYVNRTI
REPYEVYINRTV
EKEVVVYINRTVEVP
KEVFVNRSV
DKVVYVNNTIYVPTEVYINRTVEVTKVVYVNQTVEVPTVVYVNRTI
REPYEVYINRTIMEPCETKTVYVNNTVYVPTEVYINRTVEVPTLVYVNRTVVVPV
EVYINRTI
REPYEVYINRTVEVPVEVYINRTVEVPVEVYINNTVYVP
KEVYINRTVEVPVEVYINNTVYVP
KEVYINRTIMKPCETKTVYVNNTVYVPTEVYINRTVEVPTLVYVNRTVEVPTVVYV
NRTI
REPYEVYINRTV
EKEVVVYINRTVEVP
KEVFVNRSV
DKVVYVNNTIYVPTEVYINRTVEVTKVVYVNQTTVYVNNTVYVPTEVYINRTVEVPTL
VYVNRTVVVPVEVYINRTI
REPYEVYINRTVEVPVEVYINRTVEVPVEVYINRTIMKPCETKYECVT

GVFCTGWGRSDQTVYVNNTVYVPTEVYINRTVEVPTLVYVNRTVVVPVEVYINRTIRE
PYEVYINRTVEVPVEVYINRTVEVPVEVYINNTVYVPKEVYINRTIMEPCETKTQGNSSS
ASPSSTGSSPSSPSPNLGNYKVEMSVGLPLSVAQFDETRRTAFRISVATVASVDVSLV
QIDSVQAARRGGSGINVKFGVYDSNEKSAAGVAGKLRMNNLNDEFQKRGLPLGVQNS
DATVSNLAASSPVSSFSLPFQATIALYALAGVLVCGILTYAISWMRSCRKKQKVRPRLSII
QIGSDQVEPFDGRGLRLAFNRASSSSSLRTGQVQRRRSGSVSAIEIGTDFVRVWNPER
GREAPPAPSQLEIQNLDN

Guillardia 117614 VY parsing

MPCPAGQYSEKGSTSCVDCPAGTFSKGNSSVCTRCQEGYASEVGSSSCCKVI
VRNNTVEKSVYLNVTVEVPKFIKVKEQVPIEVQKKVYVDEESGKKYGEDRIVYLN
RTVEVPVIVEKLYTLIVLLKKLCTCTKLQWIKRIKARKIMTTKTTNHMAKTHKLTMK
KMEAIKKMKKMTRMVVYVNNTIYKDRIIDVPRN

VYINRTVEVPTL
VYVNRTVEVPTV
VYVNRTIREPYE
VYINRTVEKEVV
VYINRTVEVPKE
VFVNRSVDKV
VYVNNTIYVPTE
VYINRTVEVTKV
VYVNQTVEVPTV
VYVNRTIREPYE
VYINRTIMEPCETKT
VYVNNTVYVPTE
VYINRTVEVPTL
VYVNRTVVVPVE
VYINRTIREPYE
VYINRTVEVPVE
VYINRTVEVPVE
VYINNTVYVPKE
VYINRTVEVPVE
VYINNTVYVPKE
VYINRTIMKPCETKT
VYVNNTVYVPTE
VYINRTVEVPTL
VYVNRTVEVPTV
VYVNRTIREPYE
VYINRTVEKEVV
VYINRTVEVPKE
VFVNRSVDKV
VYVNNTIYVPTE
VYINRTVEVTKV
VYVNQTTVYVNNT
VYVPTEVYINRTVEVPTL
VYVNRTVVVPVE
VYINRTIREPYE
VYINRTVEVPVE
VYINRTVEVPVE
VYINRTIMKPCETKYECVTGVFCTGWGRSDQT
VYVNNTVYVPTE
VYINRTVEVPTL
VYVNRTVVVPVE
VYINRTIREPYE
VYINRTVEVPVE
VYINRTVEVPVE
VYINNTVYVPKE
VYINRTIMEPCE

TKTQGNSSSASPSSTGSSPSSPSPNLGNYKVEMSVGLPLSVAQFDETRRTAFRISVATVASVD
VSLVQIDSVQAARRGGSGINVKFGVYDSNEKSAAGVAGKLRMNNLNDEFQKRGLPLGVQNSD
ATVSNLAASSPVSSFSLPFQATIALYALAGVLVCGILTYAISWMRSCRKKQKVRPRLSIIQIGSDQ
VEPFDGRGLRLAFNRASSSSSLRTGQVQRRRSGSVSAIEIGTDFVRVWNPERGREAPPAPSQL
EIQNLDN

45

Guillardia 117614

Predicted secondary structure

1 M P C P A G Q Y S E K G S T S C V D C P A G T F S K G N S S V C T R C Q E G Y A S E V G S S S C C K
51 V I V R N N T V E K S V Y L N V T E V P K F I K V K E Q V P I E V Q K K V Y V D E E S G K K Y G E D
101 R I V Y L N R T V E V P V I V E K L Y T L I V L L K K L C T C T K L Q W I K R I K A R K I M T T K T
151 T N H M A K T H K L T M K K M E A I K K M K K M T R M V V Y V N N T I Y K D R I I D V P R N V Y I N
201 R T V E V P T L V Y V N R T V E V P T V V Y V N R T I R E P Y E V Y I N R T V E K E V V V Y I N R T
251 V E V P K E V F V N R S V D K V V Y V N N T I Y V P T E V Y I N R T V E V T K V V Y V N Q T V E V P
301 T V V Y V N R T I R E P Y E V Y I N R T I M E P C E T K T V Y V N N T V Y V P T E V Y I N R T V E V
351 P T L V Y V N R T V V V P V E V Y I N R T I R E P Y E V Y I N R T V E V P V E V Y I N R T V E V P V
401 E V Y I N N T V Y V P K E V Y I N R T V E V P V E V Y I N T V Y V P K E V Y I N R T I M K P C E T K
451 T V Y V N N T V Y V P T E V Y I N R T V E V P T L V Y V N R T V E V P T V V Y V N R T I R E P Y E V
501 Y I N R T V E K E V V V Y I N R T V E V P K E V F V N R S V D K V V Y V N N T I Y V P T E V Y I N R
551 T V E V T K V V Y V N Q T T V Y V N N T V Y V P T E V Y I N R T V E V P T L V Y V N R T V V V P V E
601 V Y I N R T I R E P Y E V Y I N R T V E V P V E V Y I N R T V E V P V E V Y I N R T I M K C E T K Y
651 E C V T G V F C T G W G R S D Q T V Y V N N T V Y V P T E V Y I N R T V E V P T L V Y V N R T V V V
701 P V E V Y I N R T I R E P Y E Y I N R T V E V P V E V Y I N R T V E V P V E V Y I N N T V Y V P K E
751 V Y I N R T I M E P C E T K T Q G N S S S A S P S S T G S S P S S P S P N G N Y K V E M S V G L P L
801 S V A Q F D E T R R T A F R I S V A T V A S V D V S L V Q I D S V Q A A R R G G S G I N V K F G V Y
851 D S N E K S A A G V A G K L R M N N L N D E F Q K R G L P L G V Q N S D A T V S N L A A S S P V S S
901 F S L P F Q A T I A L Y A L A G V L V C G I L T Y A I S W M R S C R K K Q K V R P R L S I I Q I G S
951 D Q V E P F D G R G L R L A F N R A S S S S S L R T G Q V Q R R R S G S V S A I E I G T D F V R V W
1001 N P E R G R E A P P A P S Q L E I Q N L D N

46

Guillardia |106372|a|112|-0.225| -0.180357142857143A

MNMNTYAVSPTNAANNNMMQYNIQPKYATAQMYNNFQQVQPVQTVSMRAMPY
NSMQVQPMTTAVIQNRSVMNCSSLKSENEMLMEEIGHLRRLLDN

ERSKLPM
EKVIV
KEIPV
EKEVI
REIPV
EKVVV
KEVPFEV
EKIVT
RDVEVLI
DKIVI
KEIIQEIPV
EKVVQ
REVPV
EKFF
EKVY
EKHIEVPV
EKVVNVEVPV
DRIVYK
ERESPPP
ERRMV

GIGLMLERLSNEYYTYVEEIVPGFAADLSGQIQVGDIVLYVNDISLEGMPLDDIKQASRMILFA
VNLFRRSAMSLNPNNYDAALRITSFESFQESENNSYQERDHDRSRSSSNNTNYNGQKGFS
LNDPRFTLTVQMIAEYAFRFEPRYDLNI

```
  1 M N M N T Y A V S P T N A A N N N M M Q Y N I Q P K Y A T A Q M Y N N F Q Q V Q P V Q T V S M R A M
 51 P Y N S M Q V Q P M T T A V I Q R S V M N C S S L K S E N E M L M E E I G H L R R L L D N E R S K L
101 P M E K V I V K E I P V E K E V I R E I P V E K V V V K E V P F E V E K I V R D V E V L I D K I V I
151 K E I I Q E I P V E K V V Q R E V P V E K F F E K V Y E K H I E V P V E K V V N V E V P V D R I V Y
201 K E R E S P P P E R R M V G I G L M L E R L S N E Y Y T Y V E E I V P G F A A D L S G Q I Q V G D I
251 V L Y V N D I S L E G M P L D D I K Q A S R M I L F A V N L F R R A M S L N P N N Y D A A L R I T S
301 F E S F Q E S E N N S Y Q E R D H D R S R S S S N N T N Y N G Q K G F S L N D P R F T L T V Q M I A
351 E Y A F R F E P R Y D L N I
```

47

Guillardia 1|162236|e|
59|-0.273728813559322|-0.0533898305084746

Articulin

MAEAAGKNLLDEMLEEATEAQKNSAFGAGSGYSQEQLSHASPSECMAADNYG
LRPPSLTGNSEFFTCKYSCGFSGEFEAVDAHERQGDCVQRVVASESAAEGYVM
SPQSGTF**GG**LFSDSFTVDRSFTSYVTRMPQLPPKPCTDCSYAKAQSEALSKRLE
DLINAKYQNQIIV

KEILVPVPQVKVV
ERVV
DRYIEVPV
ERIV
KEMIEVPVPV
EKIVI
REVPV
EKIV
EKVIV
KEV
EKIV

ERI**G**LGLGLEETVDGRIYVADIVEGFAASRNGQIRVNDTIIGIDGMDVIANGLNLKT
LINHTLGPEGSSCSLSLSRDGYQFTVKLTRVDGSQMLRSPDTSIYGPNSCTMDA
TPHTHLFSCSNSGSPQQPETFFTTLPPILSQESVHACEWSSSGWMVGLLRGCEH
GEQGAGAAGQHDGFLVL

1 M E K E S V C L K D N L I Q M P Y D S T D N F E A P P L L N E S S V A V Y S S P M K T N A M V T Y T [illegible]
51 N T N Q A V S Q V Q S V Q L H S S P N Q L L Y P V Q Q V T G I V P I Q S Q N L Q T I Q T S A V Q L S [illegible]
101 P V S I V R E E A P M V R I P R E E V T Y M Q P V H V Q Q E Q R K I V I E N E E P V F R R I P V Q Q [illegible]
151 D R I V N R H E T L Y V E N V R V K D V D V E V Q R I N L R E E P V A V E K I D V Q E K Q V P Y D V [illegible]
201 P V E N I I V K D E I K E V P V D R L N I Q E I S V P M Q D V P V E K V V V Q E I P V P I E K I I I [illegible]
251 R D V E V P V E R T V E R I I E R Q V D K V V N Q E R I V Y Q D N I I Y K K D R N V E V P V E R L V [illegible]
301 C K G K E K I Q Q S F I Q N V G F V M D S T G Q L E R N S G Y M L S Y S Q P R R V L S N N A G V L F [illegible]
351 K T K K V G L G I L L K R N S Q G E T Y I H A L L P G F A G A E S G E V Q V D D I I V T I D N Q N V [illegible]
401 D N W D L D D I K Q L T V G D E G S A C N L V L R R R G Q T H E Q V K L I R I G S S R G Q L E S F E [illegible]
451 K S P G A T

Guillardia 115012

MAELYSTNPGIYGSFPGTYYGSEQRGTDVI

DRYVVPETTTVPYQQLVYQTQTVQEPRTYMESVQKTVQVPKTVMEDHDVV
YKTPKIEMETRTIQVPKSVME
DKEIQVQVPKRIQVPQTKMVPRTVLVPVTVMVDQIIQETETRTIQVPKTVME
DREIEVPRQVYETVTRTVQRPKTIME
EKVITVQEPRIVQVPKQIPVTQTVLSQRYVT

AGVTQTVPSITSAPLMSTAYAPGGYYASMPSGNAPSQNEGQV

1 M A E L Y S T N P G I Y G S F P G T Y Y G S E Q R G T D V I D R Y V V P E T T T V P Y Q Q L V Y Q T
51 Q T V Q E P R T Y M E S V Q K T V Q V P K T V M E D H D V V Y K T P K I E M E T R T I Q V P K S V M
101 E D K E I Q V Q V P K R I Q V P Q T K M V P R T V L V P V T V M V D Q I I Q E T E T R T I Q V P K T
151 V M E D R E I E V P R Q V Y E T V T R T V Q R P K T I M E E K V I T V Q E P R I V Q V P K Q I P V T
201 Q T V L S Q R Y V T A G V T Q T V P S I T S A P L M S T A Y A P G G Y Y A S M P S G N A P S Q N E G
251 Q V

49

Guillardia 144262

MFAALKERKQIKNGRAVLSAMKHNMSESTSQRLSPDQKMQSWAVSGYNIENRD
VSSKHQTLRQFNTDINVNFQRGYGNSVVTSVNPVSFKRSSDPNKYWNPVIPTEQ
TSVTVV

DKPI
ERIVEVFHRVPM
DKVV
ERNVESIKVE
EKHL
EKDIKITQVVPVIQARTRAEDSVGLQEIK
KEVVY
EKEVL
KEEVQEIVV
DKYV
ERVWKSRIVEVI
REVEVI
KEVPTIRYV
DRVIT
KEVPVTQVVERVV

1 M F A A L K E R K Q I K N G R A V L S A M K H N M S E S T S Q R L S P D Q K M Q S W A V S G Y N I E
51 N R D V S S K H Q T L R Q F N T D N V N F Q R G Y G N S V V T S V N P V S F K R S S D P N K Y W N P
101 V I P T E Q T S V T V V D K P I E R I V E V F H R V P M D K V V E R N V S I K V E E K H L E K D I K
151 I T Q V V P V I Q A R T R A E D S V G L Q E I K K E V V Y E K E V L K E E V Q E I V V D K Y V E R V
201 W K S R I V E V I E V E V I K E V P T I R Y V D R V I T K E V P V T Q V V E R V V

50

Guillardia 1|115464|au.85_g16566

Articulin. VYV module in tail.

MAGRSPLRAYGEARGAPLESFGGSRIDDMDH**G**ARPMETH

ERLLSI
EKPI
EKPI
ERSVPVQVPRLV
EKVV
ERGKHVECVTTH
ERPVVQYVNKVVQIVKEVPV
EKIV
EKPFVRKIV
RERIIEQVVEVIKEVPV
ERIIEQIVEV
EKEVPV
EKVV
ERIHVQEVPVIETE
ERVVEVV
REIPVEIV
REVPVYIKIDP

AEWESRHSLRSSSAI**GG**VGLRLERFETGPQAGNVYVVEVVPGSPAADCGVIK
LHDLLVSVDGRAVERHNLQELHQMIRGPAGTPVVLELKRTGRQNQQVTLYRT
SQPSFMRLSDKAPVGRIVTSQELRQTGEIRNGYSSQQMYSQGMDVTQATL*

51

Rhodomonas >CAMPEP_0172143614

Articulin

…PGTRKMAKNGESFDWNHVQGAAGFATA**G**

ERIISQEVCVEQRV
ERDVNVPQ
ERIV
EKMVPRN
KDV
ERVTVTETPVVNKVYKTCKVI
KEVPV
EKVVKRQVVRKKVI
ERII
EKEVQVV
KEVPI
ERIVEQVVEVV
KEVPV
EKVI
ERTII
KEVPVVETE
EKVVEVI
KEVPVNIV
KEVPVYIK

SDLVEYEKYTIETAEAAYPVEA**G**VGMLLGKSEGFGADPNIHVLELLWGGPAR
ECGKISIQDILVRVDGKVVSHMNLHAVHNLIRGPPDTPVAFDFQRRGQGENG
KYSVTLYRRQVNQEVLGVEGTQQTMGVPLAGQPLGPYTQPGPRRGQEML

```
1   P G T R K M A K N G E S F D W N H V Q G A A G F A T A G E R I I S Q E V C V E Q R V E R D V N V P Q
51  E R I V E K M V P R N K D V E R V T V T E T P V V N K V Y K T C K V I K E V P V E K V V K R Q V V R
101 K K V I E R I I E K E V Q V V K E V P I E R I V E Q V V E V V K E V P V E K V I E R T I I K E V P V
151 V E T E E K V V E V I K E V P V N I V K E V P V Y I K S D L V E Y E K Y T I E T A E A A Y P V E A G
201 V G M L L G K S E G F G A D P N I H V L E L L W G G P A R E C G K I S I Q D I L V R V D G K V V S H
251 M N L H A V H N L I R G P P D T P V A F D F Q R R G Q G E N G K Y S V T L Y R R Q V N Q E V L G V E
301 G T Q Q T M G V P L A G Q P L G P Y T Q P G P R R G Q E M L
```

Rhodomonas >CAMPEP_0172143546

52

First half of sequence

Articulin

…QPKGPVSILRAKYFDDPSKMSTENFPLYGSNRSVTMMGDTMGFGGAKVGGVG
YSTISQPTSMGYGAVTATPTTYSSYSTGGVVTGQTYGASYGQVSTVPVTQPMGTI
TSPVTTPITTQPLTTNVTYGEPMRTITSPVQMPVQMTEQVVTYQPVVEQVPVTVYE
YPSVEVMYTQPVMRMTPPPMMAPVRMTPPPMRAPSPPPPVVRATPPPPPEEEVR
KVVKKEAPVAQRAAAPPMPPPAPVTHEVPV

DKVQLV
DREIAV
DRIHTV
DREVPV
DRVVHV
DREIPV
ERIQMV
DREVPV
DKVQVVYQ
DREIPV
DRVRTVEV
EKVV
EKY
RDVPV
EKLVYQEVAVPV
DKVVV
KEVEVPV
DRIVT
REVEVPV
EKIV
EKF
RDVPV
ERLVYQEVAVPV
DKVAIQEVEVPV
EKIV
EKF
RDVPV
ERLVYQEVAVPV
DKIAI
KEVEVPV
EKIV
EKF
RDVPV
ERLVYQEVAVPV
DKIVKQEVEVPV
ERVVT
RDVV
KEVQV
DRVVYQEVGVEVQKIVKQEIEVPV
DRVVT
REVV
KEVPV
DRVVYQEVGVEVQKIVRQDVEVPV
EKIV
EKLVEVPV
ERVIV
KEVPVVV
EKVVTQEVAVEV
ERVVVQ

53

Rhodomonas >CAMPEP_0172143546

Second half of sequence

Articulin

ERVVEVPV
DRIIYQT
RDVEV
ERIV
EKLVEVPV
ERVIV
KEVPVEVIKTVT
KEVPVEV
ERVVIQ
ERMVEVPV
EKVVVQ
ERIVEVPV
ERIVTQY
KEVPVEVTKIV
EKEII
KEIEVPVEIKVPYPV
DRVVESTIV
KEVEVI
KEVPVAI
EKIV
EKVVT
KEIPV
ERTVY
RDTHVDLGEVI
EKKKYEEAL
ERIRRLELQLA
ERVETRTVV
REV
ERAPVR

TSPPVMRAASPPIMRAAPPPPAVMRAPSPVRPRMVGLGLALERNDRDRTTYVQ
EVIHGFAAHKSGQFQEHDIVVAVDQEPIEGWNLDAIKQLTVGEEGSFCTLQMLRN
DRYFSVTLQRISPERFGSSNYDSQPRYSYQ

54

Rhodomonas >CAMPEP_0172142116

Articulin

...SIV

EKIVELEQEV
EKLVIKYIDIEV
EKPITVNKQVTIEQIIEVPTPEY
EKVYVEVPKYVEVKKTEYIKKRV
EKPIFTYNEEVIDVIRKPVIVYE
DRIVYN
DRSVEVVKTV
EKIV
EKERKEYR
EKIVI
KEVPVEV
EKIV
EKIV
EKMVIEHRTKTI
EKEVPVEV
EKIV
ERVVTKPVIQYVDEVVVQEVPVIV
EKIV
EKIV
EKPVVMYR
DKKVIKKPNITYM
DKIKYVEVPII
REKIVELRQIICV
EKVVP
KEVEIEVIKVIEQPITIYVDNVVV
KEVPVRGQAVIEEAVVTYNDSLIY
REVPRFV
DRIV
DRPV

SRFLDTIVYQPSDETGKGGGD

55

Rhodomonas >CAMPEP_0172138138

…DDRARSLRYQERRGREDRRGGAVRAEGVRDREDRLRHQGGHGPEDVQGCGDR
GGAEDDEVIVINYEEVEEIVEVPVTLAKGRVETTEGDRSIVNTGARIVEVPVIKTR

DKVV
DREVPYTV
EKVV
EKIV
EKPVIEYI
EKIVEQEVPV
EKEVIV
EKIV
EKREVKYTEEVVA
KEVPYEV
EKIV
ERVVEVPQI
EKRIQVVT
KEVPVEV
EKIV
EKIVEVPTTEF
REQVEIIDEHMTQFSGNVKQVEVEVIE
ERIV
EKPVIEY
KEVEVVQEQAVTKVI
EKVIEVPSYQH
RDKIIY
KECL
REDTQYLR
KDILL
ERQKVVY
KDVTVXVVFV
DKDTYQ
ERDVVVQVPRPKVITLD
DRKTV

GLG

56

Rhodomonas >CAMPEP_0172122140

Articulin

…PGTRKMAKNGESFDWNHVQGAAGFATA**G**

ERIISQEVCVEQRV
ERDVNVPQ
ERIV
EKMVPRN
KDV
ERVTVTETPVVNKVYKTCKVI
KEVPV
EKVVKRQVVRKKVI
ERII
EKEVQVV
KEVPI
ERIVEQVVEVV
KEVPV
EKVI
ERTII
KEVPVVETE
EKVVEVI
KEVPVNIV
KEVPVYIKSDLVEY
EKYTIE

TAEAAYPVEA**G**VGMLLGKSEGFGADPNIHVLELLWGGPARECGKISIQDILV
RVDGKVVSHMNLHAVHNLIRGPPDTPVAFDFQRRGQGENGKYSVTLYRRQ
VNQEVLGVEGTQQTMGVPLAGQPLGPYTQPGPRRGQEML

57

Rhodomonas >CAMPEP_0172121026

…DDRARSLRYQERRGREDRRGGAVRAEGVRDREDRLRHQGGHGPEDVQGCGDR
GGAEDDEVIVINYEEVEEIVEVPVTLAKGRVETTEGDRSIVNTGARIVEVPVIKTR

DKVV
DREVPYTV
EKVV
EKIV
EKPVIEYI
EKIVEQEVPV
EKEVIV
EKIV
EKREVKYTEEVVA
KEVPYEV
EKIV
ERVVEVPQI
EKRIQVVT
KEVPVEV
EKIV
EKIVEVP

TTEFREQVEIIDEHMTQFSGNVKQVEVEVIEERIVEKPVIEYKEVEVVQEQAVTKVIEKVIEVP
SYQHRDKIIYKECLREDTQYLRKDILLERQKVVYKDVTVENAVFVDKDTYQERDVVVQVPRP
KVITLDDRKTVGLGLKLNRASRDVTX

1 D D R A R S L R Y Q E R R G R E D R R G G A V R A E G V R D R E D R L R H Q G G H G P E D V Q G C G
51 D R G G A E D D E V I V I N Y E E V E E I V E V P V T L A K G R V E T T E G D R S I V N T G A R I V
101 E V P V I K T R D K V V D R E V P Y T V E K V V E K I V E K P V I E Y I E K I V E Q E V P V E K E V
151 I V E K I V E K R E V K Y T E E V V A K E V P Y E V E K I V E R V V E V P Q I E K R I Q V V T K E V
201 P V E V E K I V E K I V E V P T T E F R E Q V E I I D E H M T Q F S G N V K Q V E V E V I E E R I V
251 E K P V I E Y K E V E V V Q E Q A V T K V I E K V I E V P S Y Q H R D K I I Y K E C L R E D T Q Y L
301 R K D I L L E R Q K V V Y K D V T V E N A V F V D K D T Y Q E R D V V V Q V P R P K V I T L D D R K
351 T V G L G L K L N R A S R D V T

Rhodomonas >CAMPEP_0172111824

Articulin

…RKLPREPGMNDNFAPTLIGGGTNTYNSGYPSLTGSFKGYSTISQPSSMTS
MSYGQTQYGAVTTNPATYSSYTTGPAITGPVTTGAPVTTPITTQPVYSTYTEP
VMQTITSPVQMPVRMATEQIVTYQPVVEQVPVTVYETRPVEVMYTPPPXPLH
AAALAAAARQEGTGAGGTNGEEGTTKADTGSEGRACAAARASSRTASPGE
VPI

DKIMQV
DREVPV
DRIVSV
DREVPV
DRVVHV
DREVPV
ERVVYQ
DREVPV
DRVVTVET
ERVV
ERHVEVPT
EKLVYQEVAVPV
EKVVI
KEVEVPV
ERII
EKEVI
KEIPV
ERVVY
REVGVEV
DKIVKQEVEVTV
EKIV
EKYVEVPV
ERVVV
KEVPVVV
EKVVI
KEVPVEV
EKIVIQ
ERVVEVPVEHVIYQT
KEVPVEVIKIV
EKEVI
REVEVPVEIKVPYPV
EKIVEQQVV
KEVEVIXEVPVTV
EKMI
EKVVT
KEIPV
ERTVY
RDTHL
DRDMV
EKSTYQQALLEI
ERLKRQLEQ

RETTTYREVERAPMMRAAPPPPQQVVRAAPPPPVVRQVSPVQSTRSMPPTRPRMVGLG
LALERNANDRTTYVQEIIRGFAAHKSGQFQVHDIVVAVDQEPIEGWDLDAIKQLTVGEEGS
FCTLQMLRGDRYFAVTLQRISPQSLDSSGYGDSARWDRAAPLRVAPDAVGGRGVGAGSR
QGECVQGSKARVEVTEGEXGASVLREKESSGVAPSRRFLPLLCAWNEGGTECVRRSGG
VVLHVADAAQRPLLLGHAPAHFAGEVRVLQLRLAAPLLVPVSSSVVGTARSERPTWRSAV
TGTGFAPSLPLPGSLSPSPTLAPASLSISAPPLTPCSLPSISRTHLSSLPRHPPAGLSPPX

59

Rhodomonas >CAMPEP_0172109810

Articulin

…DSPVATKTGDMQHQDAYGIIQGSTMPSYGTIGIEGAFEHRDHVIGGGYTSP
TVGPVTFPGNSFASPSKPVTYTSPMNYAPVQTMQTVEYVPVTEYVPITEYYEA
PMREMPPPPPQQRQAPPPPPPEPEPEPEPARKTVVQAPVAVRAAAPPPAPE
PEIIT

REV
EKIVHVPYEV
EKIVEV
EKLVEV
EKIVFK
DREVPVEVQRVVPV
ERRVEVPVEV
EKIVTIEVPV
ERRVEVEV
ERRVEVPVTQYVEV
ERTVEVPHAYPVEV
EKRVEVPVPYAV
ERVVEVPV
ERRVEVPV
DRIVTQYV
DKPYPV
EKRVEVPVPYPV
EKRVEIPV
DRVVTQRVEVPV
DRVVTQYVEVPV
EKIV
EKRVEVPV
EKIVYV
DRPV
EKIVYV
DKRVEVPVPYPV
ERLI
EKIVEVPV
EKII

TKQVPVPVEVYSNVEHRVLHNEAREVGRREIGTRAGAPMYSQAQFQSWSGQAQS
SQTFQTSQAFHSSPPQASFQGQSLESVAPALASFARGGSLTAPMYKPGDYTTNAR
SNWGPSLYGSGRPVGGNVGLGLMLEQTENGITIKEVVPGYAAHKSGKIQAGDVIVS
VNGESTVNLDLDRVRSMTWGEEGTDVTVEVMRDGEKLSAMLTRSTPPAVAEDSG
RM

60

Rhodomonas >CAMPEP_0172107742

Articulin. Predicted secondary structure next slide

…RFLLSVAWRHSAKMQTQGQMYENAFSSGTFVPNANGSSQPQYSTSPAQP
LPTYNAPLSPSYGAVTAPVGNMMPMPTYTPPLSPSYGSVPAPVMTSPVQTYT
GGYSAPLSPQYGVSTAPVTMMAPTTYPVSPTTYPVGSPTTYPVTSPVTQYNQ
VQYMQPQLQTVAL**G**EVEEVTNWVPMQVQTQIVENVPITAPLTYVEPTPIYEAM
PMPIMESPPPPPPQRRVAEQPVQVQRAAPPPEPEPVRVRKTPPPERKQRAAA
PPPPAEI

EKIVEV
EKIVHVPVVEEV
ERIVEVPVEV
ERIVYQ
DREVPVTVEV
EKVVTV
DRTIEVPIEV
EKIVQVPV
DKIVQVPV
ERIV
EKIV
EKEVPV
ERRIEVPVPYPV
DRIV
EKIVEVPV
EKIVY
KEVPVPVQMSP
ERVVV
KEVPVPIEVV
REVPVPV
EKVVY
KEVPVGVQM

GQHMEIRQHERVQVGQREHTYTDGPQYALQQFAASHSPQQIQYQTSPGQYQTQ
QFQTSPQQMQQFMQYQPQYQTSPPQALYSTSQMFQSSPGTRISQQQLSSPQAV
YSTLPGDLSNLSLRSPGGMAGTNLYGSQQAAAGSRRVGLGLVLERNDANRSIVIRD
LVPDFAAHRSGKVQRGDILLAVDAEPTTGYDLDQIKYLTSGEDGSQVMLDLLRNGA
RVQVPLVRSGPGGGGGGGXDDADARGAARQLGWGARVLRPVALRRERRRRWP
SISPAMLEQSDERGSPIVRSRRRRVPTHWCEQTPCTPARVWARRARVEQALTKW
RRALGRRVMEPLLRVPEEQHEAVLSRVAWESQTRAPRGGVHFGGRRRAASPASS
TLPVFLVWSEVFMSX

61

Rhodomonas >CAMPEP_0172107742

Predicted secondary structure

1 R F L L S V A W R H S A K M Q T Q G Q M Y E N A F S S G T F V P N A N G S S Q P Q Y S T S P A Q P L
51 P T Y N A P L S P S Y G A V T A P V G N M M P M P T Y T P P L S P S Y G S V P A P V M T S P V Q T Y
101 T G G Y S A P L S P Q Y G V S T A P V T M M A P T T Y P V S P T T Y P V G S P T T Y P V T S P V T Q
151 Y N Q V Q Y M Q P Q L Q T V A L G E V E E V T N W V P M Q V Q T Q I V E N V P I T A P L T Y V E P T
201 P I Y E A M P M P I M E S P P P P P P Q R R V A E Q P V Q V Q R A A P P P E P E P V R V R K T P P P
251 E R K Q R A A A P P P P A E I E K I V E V E K I V H V P V V E E V E R I V E V P V E V E R I V Y Q D
301 R E V P V T V E V E K V V T V D R T I E V P I E V E K I V Q V P V D K I V Q V P V E R I V E K I V E
351 K E V P V E R R I E V P V P Y P V D R I V E K I V E V P V E K I V Y K E V P V P V Q M S P E R V V V
401 K E V P V P I E V V R E V P V P V E K V V Y K E V P V G V Q M G Q H M E I R Q H E R V Q V G Q R E H
451 T Y T D G P Q Y A L Q Q F A A S H S P Q Q I Q Y Q T S P G Q Y Q T Q Q F Q T S P Q Q M Q Q F M Q Y Q
501 P Q Y Q T S P P Q A L Y S T S Q M F Q S S P G T R I S Q Q Q L S S P Q A V Y S T L P G D L S N L S L
551 R S P G G M A G T N L Y G S Q Q A A A G S R R V G L G L V L E R N D A N R S I V I R D L V P D F A A
601 H R S G K V Q R G D I L L A V D A E P T T G Y D L D Q I K Y L T S G E D G S Q V M L D L L R N G A R
651 V Q V P L V R S G P G G G G G G G X D D A D A R G A A R Q L G W G A R V L R P V A L R R E R R R R W
701 P S I S P A M L E Q S D E R G S P I V R S R R R R V P T H W C E Q T P C T P A R V W A R R A R V E Q
751 A L T K W R R A L G R R V M E P L L R V P E E Q H E A V L S R V A W E S Q T R A P R G G V H F G G R
801 R R A A S P A S S T L P V F L V W S E V F M S X

62

Rhodomonas >CAMPEP_0172104778

First half of sequence

Articulin

…QPKGPVSILRAKYFDDPSKMSTENFPLYGSNRSVTMMGDTMGFGGAKVG
GVGYSTISQPTSMGYGAVTATPTTYSSYSTGGVVTGQTYGASYGQVSTVPV
TQPMGTITSPVTTPITTQPLTTNVTYGEPMRTITSPVQMPVQMTEQVVTYQP
VVEQVPVTVYENPSVEVMYTQPVMRMTPPPMMAPVRMTPPPMRAPSPPPP
VVRATPPPPPEEEVRKVVKKEAPVAQRAAAPPMPPPAPVTHEVPV

DKVQLV
DREIAV
DRIHTV
DREVPV
DRVVHV
DREIPV
ERIQMV
DREVPV
DKVQVVYQ
DREIPV
DRVRTVEV
EKVV
EKY
RDVPV
EKLVYQEVAVPV
DKVVV
KEVEVPV
DRIVT
REVEVPV
EKIV
EKF
RDVPV
ERLVYQEVAVPV
DKVAIQEVEVPV
EKIV
EKF
RDVPV
ERLVYQEVAVPV
DKIAI
KEVEVPV
EKIV
EKF
RDVPV
ERLVYQEVAVPV
DKIVKQEVEVPV
ERVVT
RDVV
KEVQV
DRVVYQEVGVEVQKIVKQEIEVPV
DRVVT
REVV
KEVPV
DRVVYQEVGVEVQKIVRQDVEVPV
EKIV

63

Rhodomonas >CAMPEP_0172104778

Second half of sequence

Articulin

EKLVEVPV
ERVIV
KEVPVVV
EKVVTQEVAVEV
ERVVVQ
ERVVEVPV
DRIIYQT
RDVEV
ERIV
EKLVEVPV
ERVIV
KEVPVEVIKTVT
KEVPVEV
ERVVIQ
ERMVEVPV
EKVVVQ
ERIVEVPV
ERIVTQY
KEVPVEVTKIV
EKEII
KEIEVPVEIKVPYPV
DRVVESTIV
KEVEVI
KEVPVAI
EKIV
EKVVT
KEIPV
ERTVY
RDTHVDLGEVI
EKKKYEEAL
ERIRRLELQLA
ERVETRTVV
REV

ERAPVRTSPPVMRAASPPIMRAAPPPPAVMRAPSPVRPRMVGLGLALERN
DRDRTTYVQEVIHGFAAHKSGQFQEHDIVVAVDQEPIEGWNLDAIKQLTVG
EEGSFCTLQMLRNDRYFSVTLQRISPERFGSSNYDSHPATRTSKQLGGRH
GALGAANVAQRCNWHWLRSLSSSPWLLVSLSHPGSRVSVDLRSSTHSLQ
LAIHIQDTSQLPPPPSTSRSFSASRSLPSSPAQRPSLPSEALHPDIKYMX
