# Supplementary material for: Epiplasts: Membrane Skeletons and Epiplastin Proteins in Euglenids, Glaucophytes, Cryptophytes, Ciliates, Dinoflagellates, and Apicomplexans
Source: mBio. 2018 Oct 30;9(5):e02020-18. doi: 10.1128/mBio.02020-18 (PMC6212826; doi:10.1128/mBio.02020-18)
Supplement: TEXT S5 [file mbo005184120s5.pdf]

1

Key to Gene Annotations

- **General organization:** The medial acid-base dyads (ABDs) are "parsed" into "strings that initiate with a dyad. N-term domain is denoted the head, C-term the tail.

- **Highlights**: Yellow, tyrosine; Green, cysteine; Pink, VPV; Gray, ABD in predicted head and tail domains.

- **Colored font:** Red, glycine residues in ABD domains; Bold-faced red, last G residue in head and first G residue in tail; Blue, alanine residues in ABD domains; Green, repeated string domains.

- **Underscores:** Acid-base triads or tetrads.

- **Commentary**: Notes on distinctive gene features (e.g. orthologues, localization patterns) in green font at top of some pages. Predicted homology domains (e.g. PDZ, coiled-coil) are given in Supplement Table 2.

- **Secondary structure**: PSIPRED predictions for a subset of proteins. Yellow, amino acids predicted in β-strand; pink, amino acids predicted in α-helix; no highlight, amino acids predicted in disordered (random coil) domain.

2

Karlodinium veneficum AM931978.1

Articulin
Orthologue of AM931979.1 and AM931980.1

MMYPGAYGAPLVGGTIGGGYGGYPTGAVETIGAGYGGYGGYSTGAVETI
GYGGYGGY**G**ATPIMSAPMVTAAPQVQTV

ERVVEVPQLQVQEIVRQVPRVMTQEVVXNVPVPQIQTV
EKVVEVPQVQTFETIIPVPQVQVQEVVRQVPRVQMQEVVRQMPVPQVQTV
EKVIEVPQTQVM
EKVVPVPQVVTQEVVRQVPRPQTVELTRQVPVPQVQTV
EKTMQVPQVQMV
ERTMPVPQVTVQEVVRQVPRAMQVEVVRAVPVPQTATVTKRVEVPQVQTV
ERTVPVPQLSVQEVV
REVVKVVPQEVVRPVPVPQIQTV
EKVVEVPQVQVV
EKVMPVPQVQVQEVIRQVPRAIPVEVVVQAPVPQVATVTKRVEVPQIQTV
EKVVPVPQVSIQEVVRQVPRVMVQEVLKQVPRVMVQEVVKQVPVPQVQAV
ERVVEVPEVQVV
EKIVEVPQVQTV
EKIVQ

APAPVMAAPMIETFAPAPIMTSSIPMTTMAAPTY**G**AGFGYPAPMVGSISAALP
MTTMAAPTYGAGLGYPAPMVGSISAALPMTTMAAPTYGAGFGYPTTIF"

3

Karlodinium veneficum AM931979.1

Articulin
Orthologue of AM931978.1 and AM931980.1

MYPGAYGAPLVGGTIGGGYGGYSTGAVETIGAGYGGYGGYSTGAVETIGYGGYG
GYGATPIMSAPMVTAAPQVQTV

ERVVEVPQLQVQEIVRQVPRVMTQEVVRNVPVPQIQTV
EKVVEVPQVQTFETIIPVPQVQVQEVVRQVPRVQMQEVVRQMPVPQVQTV
EKVIEVPQTQVM
EKIVPVPQVVTQEVVRQVPRPQTVELTRQVPVPQVQTV
EKTMQVPQVQMV
ERTMPVPQVTVQEVVRQVPRAMPVEVVKAVPVPQTATVTKRVEVPQVQTV
ERTVPVPQLSVQEVV
REVVKVVPQEVVRPVPVPQVQTV
EKVVEVPQVQVV
EKVMPVPQVQVQEVIRQVPRAIPVEVVVQAPVPQVVTVTKRVEVPQVQTV
EKVVPVPQVSVQEVVRQVPRVMVQEVLKQVPRVMVQEVVKQVPVPQVQAV
ERVVEVPEVQVV
EKIVEVPQVQTI
EKIVQ

APAPVMAAPMIETFAPAPIMTSSIPMTTMAAPMVGSISAALPMTTMAAPTYGAGFGYP
APMVGSISAALPMTTMAAPTYGAGFGYPTTIF

4

Karlodinium veneficum AM931980.1

Articulin
Orthologue of AM931978.1 and AM931979.1

MYPGTIGAPLMGGYGATYGSPVVETFGAPLVGGYGIGAPVFETFASAPIMSAP
MVTAAPQVQTV

ERVVEVPQLQVQEIVRQVPRVMTQEVVRNVPVPQIQTV
EKVVEVPQVQTFETIIPVPQVQVQEVVRQVPRVQMQEVVRQMPVPQVQTV
EKVIEVPQTQVM
EKIVPVPQVVTQEVVRQVPRPQTVELTRQVPVPQVQTV
EKTMQVPQMQMV
ERTMPVPQVTVQEVVRQVPRAMPVEVVKAVPVPQTATVTKRVEVPQVQTV
ERTVPVPQLSVQEVV
REVVKVVPQEVVRPVPVPQVQTV
EKVVEVPQVQVV
EKVMPVPQVQVQEVIRQVPRAIPVEVVVQAPVPQVVTVTKRVEVPQVQTV
EKVVPVPQVSVQEVVRQVPRVMVQEVLKQVPRVMVQEVVKQVPVPQVQAV
ERVVEVPEVQVV
EKIVEVPQVQTV
EKIVQ

APAPVMAAPMIETFAPAPIMTSSIPMTTMAAPMFETFAPAPIMASSIPMTTMAAPM
VGSISAALPMTTMAAPTYGAGFGSPTYGAGFGYPTTIF

```
  1 M Y P G T I G A P L M G G Y G A T Y G S P V V E T F G A P L V G G Y G I G A P V F E T F A S A P I M
 51 S A P M V T A A P Q V Q T V E R V V E V P Q L Q V Q E I V R Q V P R V M T Q E V V R N V P V P Q I Q
101 T V E K V V E V P Q V Q T F E T I I P V P Q V Q V Q E V V R Q V P R V Q M Q E V V R Q M P V P Q V Q
151 T V E K V I E V P Q T Q V M E K I V P V P Q V V T Q E V V R Q V P R P Q T V E L T R Q V P V P Q V Q
201 T V E K T M Q V P Q M Q M V E R T M P V P Q V T V Q E V V R Q V P R A M P V E V V K A V P V P Q T A
251 T V T K R V E V P Q V Q T V E R T V P V P Q L S V Q E V V R E V V K V V P Q E V V R P V P V P Q V Q
301 T V E K V V E V P Q V Q V V E K V M P V P Q V Q V Q E V I R Q V P R A I P V E V V V Q A P V P Q V V
351 T V T K R V E V P Q V Q T V E K V V P V P Q V S V Q E V V R Q V P R V M V Q E V L K Q V P R V M V Q
401 E V V K Q V P V P Q V Q A V E R V V E V P E V Q V V E K I V E V P Q V Q T V E K I V Q A P A P V M A
451 A P M I E T F A P A P I M T S S I P M T T M A A P M F E T F A P A P I M A S S I P M T T M A A P M V
501 G S I S A A L P M T T M A A P T Y G A G F G S P T Y G A G F G Y P T T I F
```

5

*Kryptoperidinium*_MMETSP0120_c8558_g1_i1_g26451

MAYSNRQSPSSPTAREPSVQRQTPVATRQDPPQASPVSSSASRKVEYHTVI

REQPQIETV
ERQVDVPTVEFQ
ERVVEMPQVITH
ERIVEVPEVSQTDIIKQVPRVEY
REVT
KEIALPVYHGI
DKVVEVPQQLV
KEKITEVPQVQYVELIRQVPKPQLQYI
DKHV
EKPVIELQ
ERPVEVPVVTT
REEIVEVPRYEHMELV
KEVP
REEIQQVQKQVIRPEVQLV
ERPVEVVLTELH
DRITEVPEVEY
REIIRQVPKVEIRYI
DKEVPITKVEYI
EKVVEVPQIIYE
ERIVEVPEVEV
REVIKQVPVAVTEYV
EKEVPKHIMEYY
EKIVDVPTVLNHETVIEVPQVLSMDLQTRVPRIDYQSVH
KEVPKYQIMAQ
ERVVDVPEVIQH
ERPRHVTEVQTVDAVTQVSKPYTEYV
EKQVPRFETQAVEMVVEVPQVL
REEFIQEVPQVQI

AEAIKQVHQPKFEQAVKQVPKVQMEYVERVVQVESKMDREKEYIMQPVIQKETV
YQPIVQRTIFTAPQEPAVAPPPPQEPRVVSPPPPPQEPTVAPLVVTTVTAPPRTT
ATTTITAPPQYMTQAAPQYAAATTITAPPQYMTAPAATQSLGSPQYMTAPAEPAT
RMVQPMSVFDALDTNHDGKLTREEFNRGVAQQAAPTAPGAFAPPARTTTVVAP
GIVATGPQPMVAKLPPTTVRAQSVTIGQPSVTSIAPRPATQVVTTAAGAPMSVFD
ALDKNHDGKLSREEFAQLRVG

6

*Kryptoperidinium*_MMETSP0120_c13725_g1_i1_g45335

Articulin

...MAMMVAPSVGTTQWYS

REIPQVQVV
ERVVEVPQVQV
KENIVEVPTVLYQ
ERVVEVPKIQDTILTKQVPRVEVQEVT
KEVVRPVNTIT
ERVVEVPMTLTTEVVHEVPVVQQVDVMKQVPKPTV
REVQKQVYKPTPQY
RERIVEVPHVMFSEQIVEVPQVQIEELI
KEVPRHEMQVVQ
KEV
EKPVYQLV
ERVEEVPMTTV
KECILEVPQVVNVDVVKQVPRHEVQVVQ
REVPKTEIQYV
EKIVEIPQIVYE
ERIVEVPVVET
REVVKQVPKTEVQYI
DKRVPKKIVQYV
EKIVEVPQIIYE
ERAVEVPQVVNVEAITSVPRPQTQMVR
KEIPKHIFQAQ
EKIVEVPLTLNH
ERPVEVPVTQYVDVTVQVPSAEVQII
DKQVPVVQIEA
REQVVEVPQAFYE
ERLVDVPQVQMAEVVRQVLVPQVQEI

AKQIPKIVKTEVRQRVVQVPSTLIQETAVEVPKVMMQEVVTQKASAQMQQRIV
QNWTQYQQPISREEIVQGSKEAVVGGVHEASVIGTRSYAVPPDQIERSERVV
TESRLSQEAPVLTTVNMPPDPQPVYRAAPPQMMYAPPPVAQAAPPIVTTTTT
TAAPVYLHPTHAAPPTTAYGGAGSGVPMEPVAVAHP

7

Kryptoperidinium MMETSP0120_c12023_g1_i1_g37885

Articulin

MTQEELVHVPKIIPQ

ERVVQRHVEQVVEVPITMTQEEVVHVPVVVQQARTHHIHVEEIVEVPVPMT
QEELVHVPKVITQQRDHHIHVEEIVDVMVEQRVEEIVHVPVIQTQ
ERIVQNPIEHVVEIPKPQVI
EKTIEVPKIQIE
EKIIKVPKVVQTVIDTTVQNQVQTIEIVKPKIIQKIVQRKKPIIQDEIRQVPKIEKQ
MVPIQKVV
EREVQMPQVQIIDEIVEVPVQKQVQVPMVMKVQKVVDIPQVEIVEQDVHVPV
QKHRHVPMV

SVAQKFVDIPQVEFVD

8

*Kryptoperidinium*_MMETSP0120_c320_g2_i1_g1076

MGSRAGPMIQEVVREVPGREVITHCTVIQYVPEIV

EKEKVVEIPQFNLQYLEEEVVQRLTH
EKIVEVEQEQHFELLTQESRVEYQEVA
KEIQKPVFEP
REVIVEVPHVLH
KEVLVEVPQVQIIDLLKQVP
KEHRQRKVVHV
EKPVVQY
RERRVEVPAVTV
KEQLVEVPVTQELEVVRQIAGPPQVQEV
EKQVN
KEIFEINETTQEVPVIETM
EKVVEFPQVEI
REVVKQVPRIEVQYI
DKEVQKIEYQYV
ERIVEVPHVVYE
ERIIEVPEIEV
REVVRQIPKPMVQYV
EKRIPKQELRYL
ERTVQQPLLLQIEQPVEVPRVEIIETVAQVPRPVPQYV
DKQVQKVVIEP
REIVEEVPVLVQQEQ

AVEIADVQIMDTVTQVSAPRTEYRDKPVPAVETKVQERIQQVPYVLTKDRLVE
VPQVQYVDIVTEEPSYDIREVLKEVPRYTVEYRHTIEEVSQQIAGPGGAPSPR
LSA

9

*Kryptoperidinium*_MMETSP0120_c1965_g1_i1_g5573

…TAPMAMMVAPSV**G**TSQFYS

REIPQVQVV
ERVVEVPQVQV
KEHVIEVPTVLYQ
ERVVEVPKIQDTILTKQVPRVEVQEVT
KEVVRPVNTIS
ERVVEVPLTLTTEVLHEVPVVQQVDVVKQVPKATV
REVQKQVYKPTPQYR

10

*Kryptoperidinium*_MMETSP0120_c5042_g1_i2_g15237

…QVEYV

DRVVEVPQVEYV
DRVVEVPQVEYVDRPIQVPRVEYI
DRPIEVPKVQRI
ERQEVPQVDWVL
EKVMEP

TTMQQVVRPPTYHRAPPIVQVAVAPPRTTAMLPPQHYVMAGTAPV

11

*Kryptoperidinium*_MMETSP0120_c8927_g1_i1_

…IVEVPQVQVQEVVKPVPKVRVQEVV

REVPKYETKIV
ERIVDVPQVEYI
DRVVEVPEVRYV
EKLVEVPRVSYQDVVRHVPKIEVQEVVRTVPKVEIQT
RERVVEIPEVQYV
ERVVEVPEVQIQEVVRHVPKIEIQ
ERIRHVPKYETQLV
ERLVEVPEVQYV
DRIVEVPQIDYQEVVKPVPKIEVQEVIRQVPKYETQIV
EKLVEVPKIQYT
ERVVEVPEVQYV
EKIVE

12

*Kryptoperidinium*_MMETSP0120_c11415_g1_i2_g35718

…NWSVLELVLRARMVCMSDCALRWLAAEVRRLR

REV
DKMVKQKTEEIVHVPMGQQEQKQIVPIRKAVG
REVQTPQVPIFNETVDVPVQKQVVELD
EREVLQILIHDEVDVPVQKQVVELD
EREVLQILIHDE

13

*Kryptoperidinium*_MMETSP0120_c15248
_g1_i1_g55834

…DASLVARALRCIEEAAPRGSMSPLDRSRTIDRQKHEDLLKTATHVEHKGY
K**GGGG**P

DKVVLPPGKSRHVHTAVKQIHVPV
ERTVRVPVVKKHV
ERQTETKVVNGVKMVPVKKYRTV
KETVLET
KEEVVEGF
REEWKKVKVPT
KEVVKKQVPRTVTRQVPYVEYVP
KEVV
REVRVP
RDVIK

EKH**G**IRVDKHLGTKVMTIEEDHHYEMRPVKVKTGDVRVMETGYQHHGKSQ
HGRSQFNP

14

*Kryptoperidinium*_MMETSP0120_c26334_g1_i1_g80820

MSQPAASAQVPMEPLA**G**SPMPPQHAEQHFSMPNAAEEFQL
EPEYY

ERVVEVPRIHVEN
RERIIEVPQPQIV
DRIVEIPQVQEVIKQIPGEIETHIIT
KEVPKIEVKQV
ERIVEVPQIEYQ
DRYVEVEEV
REIVRRVPRIEVHEIPI
ERIVRVPKKIIQEIEQPIYRPVPHLVQQRV
EREIPI

AKTQVQTLEVVQQVPVPMN**G**GSGMFMPMEPGPGMMQQP

15

*Kryptoperidinium*_MMETSP0120_c52233 _g1_i1_g123926

MAYGGGPAYPMQFDGAQQRAAMGPGPSDAYQGNYPKPFAAHASAATTNF
TSGGGGFQGQSSGYQQQQSSGFQQQFGAQQAYGDYDPSVFESLPKELLQ
GAVSFEVRDPTLVSERVVETQEHYFAAVPSSMFHSHDARDREAVRAVVNKV
ASGDGVAVAGYRPVYMM

EKVVEVPHVIT
REY
DRYVPKPEVI
RERLVEAPKIEV
ERVQQLPPRVQY
KEQIVEVPEVVIE
ERIIHVPK
REVQ
ERLIEVPKVTYV
ERIEYEDVIEY
REVPV
DKIVEVPEIEYRV
KEVEHMVPQTYIQEYFV
DRY
KEMPVTQVQEVQRIEQVPV

SVGQQPGMPPPGSSFLVPRGTYQPGHYGFDGSRQG

1 M A Y G G G P A Y P M Q F D G A Q Q R A A M G P G P S D A Y Q G N Y P K P F A A H A S A A T T N F T
51 S G G G G F Q G Q S S G Y Q Q Q Q S S G F Q Q Q F G A Q Q A Y G D Y D P S V F E S L P K E L L Q G A
101 V S F E V R D P T L V S E R V V E T Q E H Y F A A V P S S M F H S H D A R D R E A V R A V V N K V A
151 S G D G V A V A G Y R P V Y M M E K V V E V P H V I T R E Y D R Y V P K P E V I R E R L V E A P K I
201 E V E R V Q Q L P P R V Q Y K E Q I V E V P E V V I E E R I I H V P K R E V Q E R L I E V P K V T Y
251 V E R I E Y E D V I E Y R E V P V D K I V E V P E I E Y R V K E V E H M V P Q T Y I Q E Y F V D R Y
301 K E M P V T Q V Q E V Q R I E Q V P V S V G Q Q P G M P P P G S S F L V P R G T Y Q P G H Y G F D G
351 S R Q G

16

*Kryptoperidinium*_MMETSP0120_c57047_g1_i1_g136537

…IEVQVQ

ERIVEVPQVQVV
EKTVEVPQVQIQEVVRHVPKIHVQEVVRHVPKIEVQVQ
ERIVEVPQIQTV
EKMVEVPQIQVQEVVRHVPKVHIHEVVKHVPKIEVQV
KERIVEVPQVEV
REKVVEVPRIEVQEVVRHVPKHIIET
REHIVEIPCVQIQ
ERIVEHPEIHVNEV

TRHVNKVVEVQEVVKNVTKVDWQ**G**ANAASLGTTFDGAATLPPGSPIHSVASV
QTAMPGAPTMGWGGAPSSPVHAPTMRLPQQRVMMEPSGYS

17

Kryptoperidinium MMETSP0120_c71290_g1_i1_g159348

Repeated strings in lower two-thirds of ABD domain. Homologous to string repeats in *Symbiodinium* MMETSP1367_c42055_g1_i1_g70685 (5.39).

…ELAARRPPDFDNWFDPRAFDDRPPGYAGSRPVTEPIAGSPMEPIACSSRQ
VVEPVACSSPQPDASRSPSSPGSWHYGRNLGYARGELDI

RDSVV
ERSEQFR
REVP
KEVRKRQTVELITEVPKPEVQYV
EKLVEVPEVQYV
EKIVEVPQVQFV
EKIVEVPQVQYQEMVRHVPKIVTQEIVKHVPRVEIQTV
EKIVEVPQVQYV
EKIVEVPQVHIQEVVKHVPRVEVQEIVRHVPKIEVQVQ
ERIVEHRQVQFV
EKIVEVPQVMVQVV
DKHVQKIEVQEIVKHVPKIEVQVQ
ERIVEHPQIQVV
EKLVEVPQVMVQVV
DKHVSKIQVQEVVRHVPKIEVQVQ
ERIVEHRQVQFV
EKIVEVPQVMVQVV
DKHVQKIEVQEIVKHVPKIEVQVQ
ERIVEHPQIQVV
EKLVEVPQVMVQVV
DKHVSKIQVQEVVRHV

18

*Kryptoperidinium*_MMETSP0120_c72315_g1_i1_g162326

...GGGAPAACRALTPPAPLSLPMEALVGPLGGGVLPAVMRVTLPATTAVVPA
HPPTPRHGAGCGGFFVCGTPRALTPPPRAPTPRASTVGTSGVALMPPPRVM
TRPLPSDSSSPIRRRSSGVASSPTRSDMSAPRSAKAVVRLSEPVAVVTCAAA
GACAMEPGGESEATPVVT

RESQPASQMLTDYQTVI
REKPTIQEVVKEIEVPEFHVY
ERVVEVPVTLIE
ERILEVTH
EREVEAVKQVPRVSYQELAIDVQKLVPTPV
ERIVEVPQVLLE
ERLVEMPQPYYVDCLKEVVQPQYQAVQRNVFKPVVQLV
EKHVEVPAPTIKEELVEIPQHVEVELLRQVARHEVQEV
EKRV
EKPEVNVRHLVKEVPQVEFQ
EKVVEIPRVEI
REVVRQVPKVEVVYI
EKRVPK
REVQWV
EKIVEVPQIVYE
ERIIEVPEVEVHEVVRHVPVTTVQYI
DKPVPKFEMRYT
ERQVDVP

TELTLTHEQAVEVPEVSSVEVVTQVARPVVQHVDKQVPRVSLQAEQRFVDCPVP
VRCEQPVEMPEVQYIDTVRQVLRPSYHIQDKPVASVEIQAVERIVEVPQVFLEER
VVEIPQVQIVEALKQELQPIVQEVIKEVPQYQIEYTERVVEVSSSVLQEINPESQLLQ
STATVCSGRTLPPGAHGPMLACQERVGSEMLPQRPVSWHPHYVQPPALPPPPP
LAPPHLGAPGGVREESFC

19

*Kryptoperidinium*_MMETSP0120_c93491
_g1_i1_g203158

…SHLGACLLAIATATPRPPPARARSALRCRTCDSAATQQAMSTSAVSTGAY
RQEQPMHTGSVLSSSSRFANVPPMEPTAMTSMMRPHIQEVVREVPGREVI
THSTVIQYVPEIV

EKEKVVEIPQFNLQYLEEEIVQRVTH
EKVVEIEQEQQFEILTQEARVEYQEVM
KEVQKPVFEP
REVIVEVPHVLH
KEVLVEVPQVQIFELIKQVP
KEHRQRKVVHV
EKPVVQF
RERYVEVPAVTV
KEQLVEVPVAQEMEVVRQVAGPQEVQQV
EKQVN
REVYEINEATQEVPFVETM
EKVIEVPQVEV
REIVKQIPKIEVQYI
DKQVPKVEYQYV
EKIVEV

20

*Kryptoperidinium*_MMETSP0120_c111325_g1_i1_g235499

…SELACGASAGSQPMMMAPQVMQYGGGGPPQEPTVMRPQTPGTTAI

REMPKVELV
EKVVEVPNVQVQ
EKFIDVPTTLYQ
ERIVEVPQIQVAELIKQVPKVEVQEVVKHVAKPVTEVY
ERVVEVPQVMSV
EKLVEVPQVQQVEVVRQIVRPQVQYV
EKQVPKPVYEV
REKIVEVPQATIQEQLVEVPKIEYVELV
KEVPRPEVQVVQKNV
DKPVVQCV
ERIVEVPQVTVQEVIMEVPQVEIQEVVRQVPKVEVQMV
DKQVKK
REIQYI
EKIVEVPQIVYE
ERIVEVPKIEY
REIIKQVPKPQIQYI
DKKVPKHIIEYV
EKIVEVPQVVYE
ERAVDVPQVHHVEAITEVPKPHVQQVQKPIPKYEIRAQ
ERIVEVPTILRVEQPIEVPQVLVAEVVTQVPRPEIQYI
DREIPVIRTEA
REQIVEVPQVQIEELI
KEVPR

21

*Kryptoperidinium*_MMETSP0120_c118147_g1_i1_g247530

MSPMAGGGLQQPVGTAPGASPASSRGFATPVGTSRSGMMMMHTSSLDGQ
SETLMEVPQHHFREKIVEVPTICA

KEVVKAVPRVEVHEVI
KEVPKVEYQVT
ERIVEVPEVHYV
EKIVEIPEVHM
REVVKTVPKVEVQEVIKNVPKVKTVFQ
EKIVEVPQVHQV
EKIIEVPQIHVQEVVKHVPKHMIQEIVKHVPRVELQTV
EKIVEVPQVSFV
EKVVEVPQIQVQEVVKHVPKVHVHEVV
REVPKIHTEY
RERIVEIPQVHIQ
ERIVEVPRIEVQEIVKTVPKVEVQIV
DRMVEVPQVQYV
EKIVEVPQIHQV
ERVIEVPRVHIQEIVKQVPKVEIQEIVHEVPRWETRFV
EKIVEVP

Oxyrrhis marina AM931982

22

MSVSGKPQFSSSSWFVGQPQYVTAQAPQYMTTQAPQYITSQAPQYLTSQAPQ
YVTSQAPQYVTSQAPQYVTSQAFEQVSYSTGVVIDTYQTGERNLVSERVTGSQL
TNDGTIVNIRKTRQVPVMQVE

ERIVEVPKIEIQTV
ERIVEIPQIQYV
DKIVEVPQIQEVI
KEVIKIQTQEQ
REVPRTVIQTV
EKVVEVPQVQTV
ERVVQVPQIQEVVRQVPRVEIQDVYY
EREVQIPRLVC

GDGGSRSGRFLCLRSWMCRWR

```
  1 M S V S G K P Q F S S S S W F V G Q P Q Y V T A Q A P Q Y M T T Q A P Q Y I T S Q A P Q Y L T S Q A
 51 P Q Y V T S Q A P Q Y V T S Q A P Q Y V T S Q A F E Q V S Y S T G V V I D T Y Q T G E R N L V S E R
101 V T G S Q L T N D G T I V N I R K T R Q V P V M Q V E E R I V E V P K I E I Q T V E R I V E I P Q I
151 Q Y V D K I V E V P Q I Q E V I K E V I K I Q T Q E Q R E V P R T V I Q T V E K V V E V P Q V Q T V
201 E R V V Q V P Q I Q E V V R Q V P R V E I Q D V Y Y E R E V Q I P R L V C G D G G S R S G R F L C L
251 R S W M C R W R
```

Oxyrrhis marina AM931981

MSVSGQPTTQQYAGQPQFMGTQQFVGQPQFMGTQQFVGQPQYIASQAPQYVTTQAPQYVT
TQAPQYVTTQAPQYVTTQAPQYVTSQPAQFITSQAPQYITSQAVEQQQVSYSTGVVIDTYQT**G**

ERNLVS
ERVTGSQLTNDGTIVNIRKTRQVPVMQVE
ERIVEVPKIEIQTV
ERIVEIPQIQYV
DKIVEVPQIQEVI
KEVVKIETQEQI
REVPRT VIQTV
EKVVEVPQVQTV
ERVVQVPQIQEVVRQVPRVEIQDVQVQ
REVQIPRLVVETVEQV
REIPVPQIVVPVEVIQEVVVQQAVQQIV
DRPVQVDQPYQV
ERVV
ERPYTVVQEQEVVRQQIVDVPRIQQRQVTV
ERPVYYD

APVDQQYQEQVQY**G**QPQMVQSMAQPQMVQSTMVQQPMMQTVQQPMMQSIQYGAPVQYGA
PMQY GAPVSYGAPVQYGAPVQYGPPGSQGGSVGVRPQP

24

Symbiodinium MMETSP1367_c11934
_g1_i1_g28925

Articulin

MAAAGMCASGGACGAGETTVTGERWEVVGKGKGSYERVTSLKYVGEGHG
SISKEPNVLISGRSPVIGAIVCMLCIPVLLGLGYLLIWSERPVGADDVLVPPVAT
EPDCVSGFDDW

EKSWT
KERQSYCCSHFQRGCAPLV
REVPKWVVHDVNVPVNVPVK
KEVPVPVTKVQTVQVKAPDPYECHDTASVDAWSADHQRWCCYAASVGCKP
IIV
DKTVYKTVTKI
KEVPVPHYIEPKKPKVHIVH
KEVPYEVPGTPKIIPVKLPRKVIY
KDRVQVVPKLIKVPEAGKVQIVHKPVPHPVHGHAIYVKVPVV
KDSYDCDE

GFENWFFGWSSVKKHFCCKTEQKGCPHTWHGSLHLHSHVHVEGEGHATGRIY
DCHAGFSNWQQGWSVSKKKWCCSREERGCEKFYCEGDSKMWHAAKKDWCC
SHFQQGCAATTLSPLGCDATCELKGHSSSCQERIHWTSHHFFSGRENSCALAYS
KVQVECDVCRACSIEAAGCTVQKVATSDAFDCHAAYNNFFRAWSPSKKKWCCTI
KRMGCEGESPPSVDPGVGMMWKHVQVNGYWTWQAVASGGGGFVKLPYDCQA
GLTHRSTGWSPGKKSWCCQHKQIGCMDVSAGAAAGAAAAAAAGGAAAGAAGA

25

Symbiodinium MMETSP1367_c12099_g1_i1_g29664

Articulin

…TFFHTFFPMKKRGGKKAPPPQEQPLAGDHPGIPEVQII

EKVVEVPRVLKQPLEHLVEEIIHVPKVVVH
ERIQHRTVEQFIDIPVPQPQEEIVHVPV
KEYQ
DRHHHVEVEQFVDIPVPVQQEDVVQIPVELPQKRVIQQSVEQLIEVPVSITQE
EIVHVPRVVYQHRHHHAEVEQVVDLHVPHHIEETVSVPKVIQQ
ERLVHHTVEQLVEVPVQIPQ
EKIVQVPKITVQ
ERQNVVHVEHVIEIPVPQTVEEIVQVPIVQTE
ERLVQNPVEITVEVPRPVIL
EKTIEVPKVQIEETIVKVPKIMQTVQHLTVQDQIQKIEVVKPQIIQRTVRRKKPII
QENITEVPQITIEHVPV
EKVVKKPVDVPQIQYV
DKIVEVPVQKQRHVPVIQKVPKIVELPTLEYVDHVVHVPITQHRHVPVVQTVK
KHVEVPVVKYEDEILHMPVQKHVHVPMVTKVPRTVEVEQVEYVDHH
VHIPVQTHRHVTV
EKRMQRTVEVPQIEYV
DKVVPVPVQKHVHVPQVAAV
ERHVEVPQIQYV
DKDLEVPVPVHHHVPVLSKVSKAVEVPV
KETI
EKVIEVPVVNQVDIPQVQTV
EKIVEVPMVQEVQKVVEVPVV

GDTLPGQQQRSCVDLPVLRREAPPEVRMEVVQGPPMPPEYVKGLVTAAPSS
AASHAASTLGGLATPPRTGSFMPPMMGSFAPPMGSGSLTPGSLWANSAGTP
TGSPISRRA

26

Symbiodinium MMETSP1367_c23383_g1_i1_g46312

Articulin

MPIARTPCESRGETEEPCSTSAPVSSARRKVKRSASSRRARSKSRKRDGA
GAESASEGEHIESAQVKDFCAKVEVAVQTDNVNLQDLRLMVPEKVVEVET
DERRQIIKYIPIERVVTV

EKRVPFV
KEVVRTVPVDRIV
EKIVEVPVEGFG
EKIVEVI
KEVPV
EKVVV
KEVV
KEVPVERIV
EKIVEV
EKEVI
KEVPV
EKIV
EKVVI
KEVPVDKTL
EKEVI
KEVI
KEVPV
ERIV
EKIVEV
EKEVI
KEVPI
EKIV
EKEVI
KEVPV
EKIV
EKIVEVEVI
KEVPVQI

ANREVMNEMITDFAEMEPFEEEVEDAQLPEEVHQAVLEAQEILGSGLEDLESF
EEQIVQRLRTETVDHERKRSVIREMVMEALTAWLKNATPETKKALMTWGQGS
KPKKPASWIGAALMKDDETF

27

Symbiodinium MMETSP1367_c26184_g1_i1_g51257

Articulin

…
ERIMEVPEVFEDELVKQVPRIQYLEV
EKPIELPVFQAQQRIQELPTLLEQECLVEVPQVQCIELI
REVP
KEVVKTVQKLV
ERPVVKVVGQPVEVPVTTLQEELVEVPVVEEVEIIKQVPVYELQQV
EKRVEVPAVELQ
ERIVEVPQTEVAENPVEVPEIEICEVIRQVPKYEIHYV
DKEVVKHEIKYI
EKLVEVPHVIYE
ERIIEVPQV
ERRELIRHVPVSRVQVV
DKKVPRHMLQVT
EKTVEVPTVITEEVPQETLEVVVVETITEVPKIQMLPVPVEVPKVVNLQIQ
ERLEEVHTTLIE
ERVVEVPEPQVVEAFTQVVRPVVEYV
DK

28

Symbiodinium MMETSP1367_c5490 _g1_i1_g12303

…FGSSSQPALIKGWLRARNSPRAQRRFSAMAPKLFHVLFALLVLVDGLGHK
RLEPNALVDSEEAIADQLVKEAEANV

ERESPAP
KESNSTKKVPVTPKKVPVEVNVS
KEV
KESSTPSK
KEA
KEDGDEDL
EKEILDMENE

TGNANKTKEVRSGCKAASMSVLAAVVLSATLG

29

Symbiodinium MMETSP1367_c7256 _g1_i1_g16315

MKELPKEIVEHHTIVKQKPEYKTVEKHIEVPEYQVSEQVQEVITRITHEKVVE
VPQTQVIETLRQVPSLEYEEVVKQVHLPLVEG

KETI
REVPTVLH
REIAFEVPQLQIVDCVKQVARVSEQRVM
KEFAKPIIQFQ
ERTVEVPHTLVQEEITEVPMVAHCELMKQVARVEVQQV
ERQV
EKPTVQLM
ERLEEVPQVEV
RENIIEVPRVEV
REVVRQVPKVQVQYV
EKKVPKPVIEYV
ERVVEVPQVLYQ
EKIVEVPEVEI
REVIRKVPKTTVQYV
DKPIKRHELRYY
ERIEEVPVHLQHEQPVEVTQVMAV
ERI
REVPKLQWQDVP
REFPKVQLQAETKAVEVPVSLVHEQPVEVEQVHTVDVISQVLKPQFMP
<u>RDR</u>VVPKISTQVQ
ERVVHVPQVLVEEQAIELPQANIVEVI
REDLAPVYQEVVKQVPKVHLDYI
ERVVEVE

TAAVNETSPAHSQVLPPASRYAQEPSSMAFSGARSSPVPSPVPSQLVTSQFW
PQSCVVPPSGTVLGTASLVSPQRSRSPSPPHLVGVPSTLGTGSMTLWPQEPT
AFGNRGS

```
  1 M K E L P K E I V E H H T I V K Q K P E Y K T V E K H I E V P E Y Q V S E Q V Q E V I T R I T H E K
 51 V V E V P Q T Q V I E T L R Q V P S L E Y E E V V K Q V H L P L V E G K E T I R E V P T V L H R E I
101 A F E V P Q L Q I V D C V K Q V A R V S E Q R V M K E F A K P I I Q F Q E R T V E V P H T L V Q E E
151 I T E V P M V A H C E L M K Q V A R V E V Q Q V E R Q V E K P T V Q L M E R L E E V P Q V E V R E N
201 I I E V P R V E V R E V V R Q V P K V Q V Q Y V E K K V P K P V I E Y V E R V V E V P Q V L Y Q E K
251 I V E V P E V E I R E V I R K V P K T T V Q Y V D K P I K R H E L R Y Y E R I E E V P V H L Q H E Q
301 P V E V T Q V M A V E R I R E V P K L Q W Q D V P R E F P K V Q L Q A E T K A V E V P V S L V H E Q
351 P V E V E Q V H T V D V I S Q V L K P Q F M P R D R V V P K I S T Q V Q E R V V H V P Q V L V E E Q
401 A I E L P Q A N I V E V I R E D L A P V Y Q E V V K Q V P K V H L D Y I E R V V E V E T A A V N E T
451 S P A H S Q V L P P A S R Y A Q E P S S M A F S G A R S S P V P S P V P S Q L V T S Q F W P Q S C V
501 V P P S G T V L G T A S L V S P Q R S R S P S P P H L V G V P S T L G T G S M T L W P Q E P T A F G
551 N R G S
```

30

Symbiodinium MMETSP1367_c7475_g1_i1_g16761

...CRKCGHKRGAVGAAVPAGSGAVPAYAAGPPVTMMAPATSVPVTAPAPV
TTYTRAPVTAPAVAQVAVRPQVQLV

EKIVEVPQVEVQ
EKYVDVPTTLYQ
EKVVEVPRVQVAEVVKQVPKIEVQEVI
KEVAKPVTEVY
ERTVEVTQNLYV
EKLVEVPKVRVVDVVKQVPKPVMKQV
EKQVVKPIYEVV
EKLVEVPISTIEEQIVEVPKVEYMECI
REVPRPELQKAQKQV
EKPVMQCV
EKVVEVPQYTIQESIVEVPVTETHEIVKQVPSIEVQTV
DKEVKK
KEIHYV
EKIVEVPQVIYE
ERIIEVPKIEY
REVIKQVPKPEVQYI
DKKVPKHIIEYV
EKIVEVPQVVYE
ERVVEVPQMHHVEAITEVPKPQMQRV
EKKIPKIQLRAQ
ERVVEVPVTLKVEQPVEVPQVLVAEQVKQVPSTEVQYV
DREVPNITYQALEQIVEVPQVI
KEERLVEVPQVQVAEFIKQVPKQQVQEIPKHIPKVEMRCV
EKIQNVPVKLMQEVAVEVPQVLRHEVISQVSQQTEQRVVQAAEEY
ERFVN
RDEVVV

GEQESQFGGAYEAKVVGVGPISPSTASAEMYERGQVSHVKSTEVRQGTASAQ
ASGLASGCAGYGSAQRAGDLFTALDTNGDGVLSREEMAALRTGPSTQGMRAP
VTMAAPMAYAGAVGAPAAMGQVTYTAPPVYAHPMAMGPVTRAPGPPVTYGAA
CGSGACVTYGQGCQGCQGSSASMRGGDLFSALDTNGDGVLSREEMAALQPA
PRPAQGVSFGGAVTYAT

31

Symbiodinium MMETSP1367_c10057 _g1_i3_g23143

…RGMGGMGMGYGPGGRGGKGKGK**G**NLPVKVIT

REVPKIEVKQV
EKIVEIPNIEYQ
DRLVEV
REV
REVVRRVPRIEV
REIPI
ERVIQVPKKVVQEVEQPVYRPVPHMVKQHV
EREIPVPKPYLQTLEVVKQVSVP

TTED**G**VVIPQSEVTPQAAVQPGQTSPPAQGASPGQPVLSAPVSRTYELPPVP
SGGTPVAPGGTVMYGSGVVQSQTQVAGGDLFSKMDKDGSGAISREEFAQAQ
QVGIATMSSPGAAGPLYGPPQSVSMGSMVMGAGFGSTPQGCAVAYGGAATP
PQAPSSASCIQSQRYPGQVGVVQSMGAMNPYATMSGAPGGAVAYASMPPQ
APCGSGCFTQTQRYASAGPAASGDLFSRLDRDGSGVISREEFEQALRSGVVG
APQSGSSQVATT

```
  1 R G M G G M G M G Y G P G G R G G K G K G K G N L P V K V I T R E V P K I E V K Q V E K I V E I P N
 51 I E Y Q D R L V E V R E V R E V V R R V P R I E V R E I P I E R V I Q V P K K V V Q E V E Q P V Y R
101 P V P H M V K Q H V E R E I P V P K P Y L Q T L E V V K Q V S V P T T E D G V V I P Q S E V T P Q A
151 A V Q P G Q T S P P A Q G A S P G Q P V L S A P V S R T Y E L P P V P S G G T P V A P G G T V M Y G
201 S G V V Q S Q T Q V A G G D L F S K M D K D G S G A I S R E E F A Q A Q Q V G I A T M S S P G A A G
251 P L Y G P P Q S V S M G S M V M G A G F G S T P Q G C A V A Y G G A A T P P Q A P S S A S C I Q S Q
301 R Y P G Q V G V V Q S M G A M N P Y A T M S G A P G G A V A Y A S M P P Q A P C G S G C F T Q T Q R
351 Y A S A G P A A S G D L F S R L D R D G S G V I S R E E F E Q A L R S G V V G A P Q S G S S Q V A T
401 T
```

32

Symbiodinium MMETSP1367_c18518_g1_i1_g41046

…PMVQKVQKEVEVPQIQYEDQVVEVPVTKQVHVPMVETVQKQVEVPQVQY
EDQVVHVPVAKHVHVPMVQTVQKQIEVPQIEYQDEIVEVPVQKQINVPMIQKV

ERSVDVPQIQYEDQVVQVPLAKQVHVPMIQEI
ERTVEVPQVEYVDNVVQVPV
EKQVHVPMIQKI
EKTVEIPQIEYVDNHVHIPVQKTRHVHVHVPV
ERPVEVNVIETL
EKVIDVPVVKQVEVPQVQTI
EKIVEVPFVQVV
EKVVEVPQV

GSTTQGSVREVDVETEPTRQEEPAQVVQQVIAGTPYPVEHAAPEVIGASPVPT
QTEDAPVAEPIQTGA

33

Symbiodinium MMETSP1367_c25489 _g1_i1_g50146

MIQKVQKIVDVPQIEIEEQVVEVPVAKHVQVPMIQKVQKMVEVPQVEYDDQV
VDVPVQKQVHIPFQQVQVPMV

EKVQRSVEVPQVEFVDEVMQIPVAKQVNVPMVSKV
EKVVEIPQVQYV
DKVVPIPVQKQVNVPMITQV
EKIVEIPQVEFVDTHIHIPVQKHRQVPVQVPV
EKPVEVNVIETT
EKVIDVPVVKQIAVPQIQTI
EKIVEIPFVQVV
EKVVEVPQV

GSTTQGSVREEHVEGETKREEHPAQVVQQVLAGPSHPVEYVTETEEPPPVQ
EPIQTAT

34

Symbiodinium MMETSP1367_c26041_g1_i1_g51043

…PRQMSGPVTKVVTAPPVTKVPSMGSPKRKLEYHTILREQATIEEV

EKAVEVPRVEVV
EKVEEVPITLIHESLVEVPQLLQDEIIREVPLPQYQEVV
KEIELPQFEA
RERIIEVPMSLIEETYVEVPRVQRLEVIKQVPKAMARKMPKHV
EKPVIQV
RERQMPVTTATV
KEEMVEVPKVEVVEMVREAAVMEVQNV
EKLV
ERIEVELVT
KEVPVVLQAIHDNIVEVPQVEYIEVV
KEIPKVEVRYV
EKTVEVPKIEYV
DRIVEVPQITYE
ERIVEVVQREVREIIKQVPKPVVQYV
DKKVPKHIYNYYDNVQ
EKLAVLKQEVPVEVPEVHAVELLTELPKPQY
EKVP
KEIPLYQIEVV
ERIESTPVVL
KEERAVPVDQVENLQVMRQVVKPVVEYV
EKTVPKVETQAV
EKEVEVPMVLRQEVIVETPQLVVAEVIREMPQEMTQQVV
KEVPKFQMEYVNKVLEVKP

ARYEAGMMSAPLTLGSSVASGSRYMEPGPVYQPSAGRAVSYGAQSMGSSMM
GGLLGSATS

35

Symbiodinium MMETSP1367_c32331_g1_i1_g57394

…DPSKATMSHWAAQFQAVSVEGEPQYLAGVGSEVQGAPQVMMQRQEQV
VAGQVKQKITEIPTIQEIVEEQEVPEVQYINKIVEVPEIIQQQIEQVVEQVVHVP
QIVEQKRVQHRHVEQFVDVPVAEQAAPQEQVVEIPVPMQEEEIVHVPKIITQ
TRQVQQPVEQVIEVPVPMMQEEVVHVPKIMTASRVQHQHVEELVEVPIPMT
QEEVVHVPKVMTATRQVQQPVEQVVEVPIHQRREEVVHVPKVVTHTRTSH
QHVEQTVEVPVPMQQEEHVHIPKILTQTRQIQQQVEQSVEVPVPMTEEVMV
HVPKIMTQTRVRHEHVEQTVEVPIPHTQ

EKVVHVPKVITQ
ERVTHQPVETT
KEVPIPMVQEQVVHVPKVVQQ
ERQHHFHVEEMVDIPIEQPVETQVHVPLVSKV
ERIVHNPVDLVVEVPRPVVV
EKSVQVPMIQVE
EKTVRVPKVLNTVVDTVVQHQVQNVQIVRPTVVHKVVQRRKPIVQ
ERVQHVPKVMVQHVPVQKVI
EKQVHIPQIQYVDEFVDVPV

ARHRRVAMPAAQAPIVHKHVHVPMVQKVQKTVEVPQIQFEDQIVEVPVAKQVN
VPVVQTMQKQVEVPQMQYEDQIVHVPVQKQVHVPMVQKVQ

36

Symbiodinium MMETSP1367_c33132_g1_i1_g59119

MLASNCKNCGNTFVSDSNFCRRCGEKREVYGASAAPMATGSIRTAAPAATL
MQEPNTQVQIEEQIVEVPTILYQ

EKVVEVPKVQETILLKQVPRVEVQEVTKEVVRPIYEVT
EKIVEVPHTLVQ
ERIEEVPKVEFVPLVKQVPKTQVREVQKQVGVPHYQP
RERIVEVPTTMVAEQMVEVPQVQI
KELL
KEVPRPEVKIVAKPV
EKPVNEYV
ERIVEVPHTTIQECIIEVPKVEVQEIVKQVPRPEVQVV
DRQV
EKLEVQYV
EKLVEVPQIIYE
EKIVEVPTVEVREVIKQVSKPEVKYI
EKRVPKKVIKYV
EKIVEVPQVIYE
ERPIEVPELIHVEAVTQVPRPHVQFVK
KEVPKVVLQPQ
ERIVEVPLTLTQ
ERPVEVPQVHVAELMVQVPKPQVEMV
DKEVPKIIVQAE
EKIVEVPQVFYEECLKHVPQVQVAEVI
KEVPHYQKQVVQKHVPKIVETKVV
EKVVPVPV

NLVRETAVEVPQVVQHEVIRQKASGAMQQRIIQTGWQYQRSARKEEVVSGIAEAQQGG
IYDAPVASVRRVSLPTNEVEVSAVITEQSVSHEAPVVTTVNLKPGMFAQTQVAPA
PVTTYGTAIVGTMGTGTAAAQSIFRAAQACPCRTAWGTGDVSASAAATSGAAAS
TMRTTRAEATACANCGNVLMPDSSFCRKCGAARSVPGAPDPFLVTEPVAPGYG
TINTQSTLPTQPL

37

Symbiodinium MMETSP1367_c38960 _g1_i1_g68168

…EQ

ERISHQHVEEVVEVPVPMTE
EKVVHVPKVVQQ
ERQHHFHVEETVDVPIEQHVEQVVHVPVVSRG
ERIVHNPVDLEVEVPRPIVV
EKCIEVPKIQVE
ERTVRVPKIVNTVV
DRVVRHSMETVEVVKPTVVHKVVQRKKPVVQ
ERVQHVPKVFIQQVQV
EKVV
EKPVEVPQIQFVDEVVDVPMVKHRHVPTVVKQQ
KEVEVPQVEYVD

38

Symbiodinium MMETSP1367_c42055_g1_i1_g70685

Repeated strings in lower two-thirds of ABD domain. Homologous to string repeats in *Kryptoperidinium* MMETSP0120_c71290_g1_i1_G159348 (5.17).

...SDSPGQWFKENPLLQSWNASAQDALAGALPGQTSQLQTPRSSMSIPRAD
MSQEVLIEVPQTLFREKVVEVPTICTQELVRAVPKVEVHEII

KEVPKIEYQVT
ERVVEVPEVRYV
EKIVEVPEVHTQEIVRTVPKIEVQEVVKTVPKVQVQVQ
ERIIEVPKVCHV
ERVVEVPQVQVQEVVRTVPRLEVQEVI
REVPKLEVRTV
EKLVEVPQVAFV
DKFVEVPQVQVQEVLRHVPKLEVHEVI
KEVPKIQMEY
RERIVEVPQLHLQ
ERLVEVPKIEVQEVVRHVPKVEVRTV
DRVVEVPQVQYV
EKLVEVPKVEIQ
ERLHHVPKIHTQVV
EKLVEVPQVQFV
EKFVEVPQVQIQEVVRHVPKIETQEIVRTVPKVEIRTV
EKIVEVPQVQFV
EKIVEVPEVRIQEVVRHVPKIEVQEVVKHVPKIEVQLQ
ERIVEHPQVHQV
EKIVEVPQTVIQTV
DRHVPKIEVQEIVRHVPKIDVKIQ
ERIVEHPQIHQV
EKVVEVPQTVVQEVVRHVPKIQVQEIVKHIPKIEVQVQ
EKIIEVPQVQVV
EKIVEVPQKQVHEVIKHVPKLQIQEVVRHVPKVEVQVQ
ERIVEVPHIQTI
EKVVEVPKVQIQEVVRHVKKPVIET
RERIVEIPVVQVQ
ERVVEHPEIHVQEVI
KEIPKVMEVQEIVKNVQKVD

WTGADVSAMSGGSIEAPTLPPATPNAASTRYVPMSAAGDCTPSGRSVATVDIN
AATLTPSTLNTQLNKLPTLKASCELEAPVIRLPEEASRQGSEVPVFEVPRDRVTV
TSAELQSRPSGSPAVAAASLVAGFCVACGSSYPPGSSFCRQCGRQVDGPSQP
QPTALPQPGMAPGMPMHPPQTAYGAPCTGMGGRPSMPTVFGSAPTQYPHGL
RGPGVDPFSFR

Symbiodinium MMETSP1367_c43021_g1_i1_g72658

MPGMPGMPGMPGMPGMPGMPPGGPEMSGRYGMDLHMPGMPGLPGMP
GMPGMPGFGLDVGLPGMRGFGISGMGMGGFDGMGFGLYGNGVSGIGPG
GLAMDLGGYGGGGIQDILGEGGMELVNGGDLDAALGLLNGPGVDANGEIQ
LPPGVEPGTKEYNDFLQQLEAQ**G**LIEI

REEIVEVPQVVVE
ERVVHVPGKKQIQ
ERLIEVPKVDWV
ERVEYEDYVEY
REVPV
DKIIEVPEIEYKIKQVDQTVPQTYIQEHFV
DKF
REVPVTQVQET
ERVEHVPVMVP
KDWQPPNYE

AL**G**MKSSPANAPAQITVT

40

Symbiodinium MMETSP1367_c44073_g1_i1_g74632

MVNTVQRTVEVPQIEYIDNHVHIPIQKHRHVPVHIPV

EKPVEVTVIETLQKIVDVPVVKQVEVPQVQTI
EKIVEIPLVQTV
EKVVEVPMMGQTTQGTT
REVDIPLAPR
REEHPAQVVQQVIM

GEDHPVQVMAAQAASIAQPGISVTATPAAASASAAPAAIQEPVQTSA

41

Symbiodinium MMETSP1367_c63059_g1_i1_g99513

…SQVWNGFSDRRGLQIHAPHPSASVVPTWRPPWNVGSQTAAAGFHKAYS
CPHCGNVYADDSLFCRKCGKPRAQVPNEVPPVPSVPMQVSKSTGRLGVSQ
TVQGGQIGPQEHVTQMTSNVSNVSRQYSAPQEAYAMRPAAMPQVREVVEVT
QLATAAALEDSIKQCQVVIR

EKPEV
REREKVVEVPTISLSENMVEVPVSLVH
EKVVEVPQVQVVEIMKQLPKVEYQ
ERVVQV
EKIQLEGRVNEVEVPHVVTQECLVELPQPKVVDVL
REVAKVQSKQLAKHFARPVVQW
KESI
REVPQTTL
REQLQEVPQLRTVEVL
REVPKPEVQRV
EKQVEVPTIQLQ
ERLVDVPCVEQ
REEPTEVAKVEI
REVVRQVPKVEVKYV
EKKVPKQVIQYV
EKTVEVPQVVYEEVIVEVPEVEI
KEMVRQVPVPVVQYI
DKPVPRRSLRAV
EKVVPFPQVFHEEQIVEVPQQMAVELAVQVPKVQYTTSP
KEFPKVLLEA
REVAVEVPVVL
REERVVEVPQVQSVPLVRQRLCPSMQAV
DKPVPYIETRVQ
ERQVQVPYVLQEEAIAEVPQQQLVELR
REELQPMAEEVLKQVPKYQVEYV
EKVVEVQ

SQVAQETSPSRESRSQAGGTAVAVSTISTQKSASLHREIQGLGLGQFHESTCPK
CGRSRVQNSRFCHGCGYQYEA

42

Symbiodinium MMETSP1367_c47066 _g1_i1_g79256

MEDAAEVNDDKGPPKCSASANGEAEVTQRTEAEGTKDEDNVEDEDVERS
QKVETSEDVAKVE

EKAVVEE
EKAVAEE
DKLEE
KTVVAE
EKTDEE
EKVV
EKVIEE
