# Supplementary material for: Epiplasts: Membrane Skeletons and Epiplastin Proteins in Euglenids, Glaucophytes, Cryptophytes, Ciliates, Dinoflagellates, and Apicomplexans
Source: mBio. 2018 Oct 30;9(5):e02020-18. doi: 10.1128/mBio.02020-18 (PMC6212826; doi:10.1128/mBio.02020-18)

Key to Gene Annotations

- **General organization:** The medial acid-base dyads (ABDs) are “parsed” into “strings that initiate with a dyad. N-term domain is denoted the head, C-term the tail.

- **Highlights**: Yellow, tyrosine; Green, cysteine; Pink, VPV; Gray, ABD in predicted head and tail domains.

- **Colored font:** Red, glycine residues in ABD domains; Bold-faced red, last G residue in head and first G residue in tail; Blue, alanine residues in ABD domains; Green, repeated string domains.

- **Underscores:** Acid-base triads or tetrads.

- **Commentary**: Notes on distinctive gene features (e.g. orthologues, localization patterns) in green font at top of some pages. Predicted homology domains (e.g. PDZ, coiled-coil) are given in Supplement Table 2.

- **Secondary structure**: PSIPRED predictions for a subset of proteins. Yellow, amino acids predicted in β-strand; pink, amino acids predicted in α-helix; no highlight, amino acids predicted in disordered (random coil) domain.

2

Euglena 80K articulin Gene.55749_G

Articulin
Predicted secondary structure next slide

MSYLAGAPVTAAASYPVVPGAAAYPVGASYPAVAQASVLRANAQFA**G**QRVIR

KEVVGNVEYIPVQHNHEVELI
EREFIVPV
EKIVQRHVPVPV
ERIVQRRVPVPRQVPVPQRVEIPVPV
ERIQHRQVPYPVEQIV
EKRIPVPVTQTVEQAVEVPVPVHRRVIQQVPVPHAVV
REVIRHEPYPVT
KEVTRQVPVEVP
REVVRQVTVDVPVQVPQHVQVPYPV
EKVVHRQVPYPV
EKVVQRQVPYPVQKIV
ERQVQVPYEVLVP
ERVEIPVPHEVITH
RDVPVPQEVIRTVQVPVPVEQIVH
RDVPYPVEQIV
EKVVQVTRQVTVPEIVQVPVPHEVIV
ERRVPVPV
ERITHKAVPYPVEQIV
EKIVQVPVPQYQKVPVQVPVPV
ERIVT
RDVPYPVEQIV
DKVV
ERQVPVPTPVQVPVPTPVQVPYPV
EKIV
DRPVPHEVVRVV
ERRVEVPYDVPVPVIETVQVPHEVIRTVEVPFPVEQIV
EKIV
EKIVHVPVPV

TRQEHVTRQVVQNTLQQTRAPPQQL**G**TQVLPGRDLGVTSGIVRGGPAAYGAYGA
TPVAAAPVTYGSVAAPVTYGAAAPVTYGAAPVTAYGAPPVYAGAPAVVTAPSSVT
RYGYGPPRPAYPATVPVAYAAAPTELRNDDFDWPPVPSVCGVQRQPQ

3

Euglena 80K articulin AAB23240.1

Predicted secondary structure

```
  1 M S Y L A G A P V T A A A S Y P V V P G A A A Y P V G A S Y P A V A Q A S V L R A N A Q F A G Q R V
 51 I R K E V V G N V E Y I P V Q H N H E V E L I E R E F I V P V E K I V Q R H V P V P V E R I V Q R R
101 V P V P R Q V P V P Q R V E I P V P V E R I Q H R Q V P Y P V E Q I V E K R I P V P V T Q T V E Q A
151 V E V P V P V H R R V I Q Q V P V P H A V V R E V I R H E P Y P V T K E V T R Q V P V E V P R E V V
201 R Q V T V D V P V Q V P Q H V Q V P Y P V E K V V H R Q V P Y P V E K V V Q R Q V P Y P V Q K I V E
251 R Q V Q V P Y E V L V P E R V E I P V P H E V I T H R D V P V P Q E V I R T V Q V P V P V E Q I V H
301 R D V P Y P V E Q I V E K V V Q V T R Q V T V P E I V Q V P V P H E V I V E R R V P V P V E R I T H
351 K A V P Y P V E Q I V E K I V Q V P V P Q Y Q K V P V Q V P V P V E R I V T R D V P Y P V E Q I V D
401 K V V E R Q V P V P T P V Q V P V P T P V Q V P Y P V E K I V D R P V P H E V V R V V E R R V E V P
451 Y D V P V P V I E T V Q V P H E V I R T V E V P F P V E Q I V E K I V E K I V H V P V P V T R Q E H
501 V T R Q V V Q N T L Q Q T R A P P Q Q L G T Q V L P G R D L G V T S G I V R G G P A A Y G A Y G A T
551 P V A A A P V T Y G S V A A P V T Y G A A A P V T Y G A A P V T A Y G A P P V Y A G A P A V V T A P
601 S S V T R Y G Y G P P R P A Y P A T V P V A Y A A A P T E L R N D D F D W P P V P S V C G V Q R Q P
651 Q
```

4

Euglena 86K articulin Gene.26294_G

Articulin
Predicted secondary structure next slide

MSWVAAQTQQGAFALDAADGRIDGKYFGSNVGVSVPGAPVTYGAAAPVTSYA
APAAVTSFAATAAPVTSFGAYGAYAPACPPCATGPRVVNDPLETRFVDVVKQV
ETIRSVDVPVPHEVVRTVDVPEHYDVPVPHAVHVQVPYPV

DKFVDVPVPHTIQKIVETRVPYPVQQVVQRRV
ERPYDVPVV
ERVNVPYPVEQVV
ERRVPVPVEQIV
ERVVQVPV
ERLV
EKVVQVHRQVPVPVRVEVPVPHEVIRTVDVPVPHEVVRTQDVPVPVEQIV
EKVVQVPVPVQKKVIQHVQVPYPVQKIV
DRPVPYPV
EKIVEQKVPYAVQKVI
DRPVPYPVQKIV
ERRVDVPVEVKVRQEVRVPYPVQKIV
DRPEPYPV
DKVVEVPQPYPVQKVV
ERRVEVPHVIQVREEVRVPYTV
DKVV
DRPVPYPVT
KEVVRYV
DRPVPQPYEVRVPQPYEVKVPVEQIRY
RDVPVPV
ERIV
EKVVQVPVPRQVPVKQIQQVPVPV
EKIVHVQRPYPVQKVVV
REVVKHVQVPHEIIQRVEHVQHVVQPVEVIEEPVIQQIV

TINKRTIQGQPYVANTTTNVIGQEVRGAYAAGAYGATVTGPAVATGYGQSVTGYGQ
SVTGYGYGAAAPVAVAGSQGGALSLDLADGRLDGRFYGAPVVSASPF

5

Euglena 86K articulin AAB23241.1.

Predicted secondary structure

```
  1 M S W V A A Q T Q Q G A F A L D A A D G R I D G K Y F G S N V G V S V P G A P V T Y G A A A P V T S 5
 51 Y A A P A A V T S F A A T A A P V T S F G A Y G A Y A P A C P P C A T G P R V V N D P L E T R F V D 1
101 V V K Q V E T I R S V D V P V P H E V V R T V D V P E H Y D V P V P H A V H V Q V P Y P V D K F V D 1
151 V P V P H T I Q K I V E T R V P Y P V Q Q V V Q R R V E R P Y D V P V V E R V N V P Y P V E Q V V E 2
201 R R V P V P V E Q I V E R V V Q V P V E R L V E K V V Q V H R Q V P V P V R V E V P V P H E V I R T 2
251 V D V P V P H E V V R T Q D V P V P V E Q I V E K V V Q V P V P V Q K K V I Q H V Q V P Y P V Q K I 3
301 V D R P V P Y P V E K I V E Q K V P Y A V Q K V I D R P V P Y P V Q K I V E R R V D V P V E V K V R 3
351 Q E V R V P Y P V Q K I V D R P E P Y P V D K V V E V P Q P Y P V Q K V V E R R V E V P H V I Q V R 4
401 E E V R V P Y T V D K V V D R P V P Y P V T K E V V R Y V D R P V P Q P Y E V R V P Q P Y E V K V P 4
451 V E Q I R Y R D V P V P V E R I V E K V V Q V P V P R Q V P V K Q I Q Q V P V P V E K I V H V Q R P 5
501 Y P V Q K V V V R E V V K H V Q V P H E I I Q R V E H V Q H V V Q P V E V I E E P V I Q Q I V T I N 5
551 K R T I Q G Q P Y V A N T T T N V I G Q E V R G A Y A A G A Y G A T V T G P A V A T G Y G Q S V T G 6
601 Y G Q S V T G Y G Y G A A A P V A V A G S Q G G A L S L D L A D G R L D G R F Y G A P V V S A S P F 6
651
```

6

Euglena Gene.3890_GEFR01000334.1
(First third of sequence)

Articulin. Predicted secondary structure at end of sequence

…FFFELFISTMSKPVTTTSGRVRYDGGSQMRVLGGATSGSASSTPIFVSETSP
ITTTHYGGSVTRTYGASTVGVGGISSHYVGGTTVAGSSLAGSTRTHAVGGTAIA
GSSALAGSSLTGSAAFGTGGSVLVGGSALVGGASSVVGGASVVGGSRVRHIG
GEGPIYVDSGYTTSGYSNVCCAPAGVRSTFVGSRREVLEAATVTQHI

EKVPVEIDVDIDTY
REIVEVPI
EKIVEVPYPV
EKIVQVPV
DRVVEVPV
ERLV
ERIVRVPEVRTVEVPVEHCI
EKLVTVPEIL
EREVAI
EKIV
ERVVRVPEIHEIEVPV
EKLI
ERILRVPEIHEIEVPV
ERIV
EKVIRVPEIRQVEVPV
ERII
EKIIRVPEIHEIEVPV
EKRIETVVRVPEVHQIEVPV
EKII
ERVVRVPEVHQIEVPV
EKIV
ERVVQTHT
RDVIEVPV
EKVV
ERIVRVPQETLIEVPV
EKIV
ERVVRVPQHTTVEVPV
EKVI
ERVVQTHT
RDIIEVPV
EKVV
ERVVRVPQETIIEVPV
EKVV
ERIVTVPQHTTVEVPV
EKIV
ERVVQTHT
RDVIEVPV
EKVV
ERIVKVPQETIIEVPV
EKLI
ERVCRVPQETIVEVPV
EKII
ERVSQTHV
RDIVEVPV
EKLV
ERIVTVP
KETVIEVPV
EKII
ERIVKVPQETIVEVPV
EKLV
ERVCQTHT

7

Euglena Gene.3890_GEFR01000334.1
(Second third of sequence)

RDIIEVPV
EKIV
ERVVKVPQETIIEVPV
EKVI
ERVVTVPQETI
REVPV
EKVV
ERVVQTHT
RDIIEVPV
EKII
ERVVKVPQETIVEVPV
ERVI
EKVCSTHT
RDIIEVPI
EKII
ERIVKVPQETIIEVPV
EKCI
ERVVTVHSTDVVEVPV
EKVI
ERIVRVPQETIIEVPV
EKII
ERVCEVQV
EKIVEVPVQQIVRVPVEVPVEHIV
ERVVKVPVETI
KEVPV
EKIVEIIRPVEVIHHIEVPV
ERIV
ERVVEVL
KEVPV
DRVV
ERLVEVPVTHTVEVPVVSVV
EKLVEVP
KEII
REVPV
EKIV
ERVVTVNVDNYIEVPV
EKII
ERVVTVPV
EKRIEVPVEVVV
ERI
KEVQV
EKIVEVPV
EKII
EKIVKVPV
EKIVEVPVECVV
ERIVQVPVTQTVEVPV
ERIV
EKIVEVQVQQIVEVPV
EKVV
ERVVKVPVDHVIEVPV
ERVV
ERI
REVPV
EKIIQVPV
EKIKIVTV
DRIVEVPVEV

8

Euglena Gene.3890_GEFR01000334.1
(Final third of sequence)

EKIVTVQV
EKIVQVPV
EKIV
EKLVEV
EKIV
ERTVTV
ERVVEVPKVV
EKIV
EKIVEVP
REYI
REVTVTSI
KEVPV
EKIV
ERIVEVPITNVVEVPV
ERVV
ERVVEIPL
ERVV
ERIVEVPV
EKII
ERIIEVPV
ERVVEHIIEVPV
ERLV
ERIVEIPV
EKII
EKIIEVPI
EKII
ERVIEVEVPIEI
EKVVEI
EKIVEVPVVV
EKVVEV
EKIVEVSGGVEIV
EKIVEV
EKIVEVPVVV
EKVIET
EKII
EKGVV

TEIVPEVSTELYSTSYSTGSKGTSSSISASGGASYSVGGGYGVSGGASTGGGYAVS
GGTGGAGYGSIGTSGGTGYSSITTGGSGTGYSSIGGTGAGVGYSSATTGGAGAGA
GTGYSSISGSGAPGGAGGYSSTSYSSGTYGAGGGTYGAGAPGAGGYGSYSSTSY
SGTGGSGSAPGRTGYTATSGSGTRSGSATRSGSGSRA

Euglena Gene. 3890_GEFR01000334.1

9

Predicted secondary structure

```
  1 F F F E L F I S T M S K P V T T T S G R V R Y D G G S Q M R V L G G A T S G S A S S T P I F V S E T
 51 S P I T T T H Y G G S V T R T Y G A S T V G V G G I S S H Y V G G T T V A G S S L A G S T R T H A V
101 G G T A I A G S S A L A G S S L T G S A A F G T G G S V L V G G S A L V G G A S S V V G G A S V V G
151 G S R V R H I G G E G P I Y V D S G Y T T S G Y S N V C C A P A G V R S T F V G S R R E V L E A A T
201 V T Q H I
```

```
  1 E K V P V E I D V D I D T Y R E I V E V P I E K I V E V P Y P V E K I V Q V P V D R V V E V P V E R
 51 L V E R I V R V P E V R T V E V P V E H C I E K L V T V P E I L E R E V A I E K I V E R V V R V P E
101 I H E I E V P V E K L I E R I L R V P E I H E I E V P V E R I V E K V I R V P E I R Q V E V P V E R
151 I I E K I I R V P E I H E I E V P V E K R I E T V V R V P E V H Q I E V P V E K I I E R V V R V P E
201 V H Q I E V P V E K I V E R V V Q T H T R D V I E V P V E K V V E R I V R V P Q E T L I E V P V E K
251 I V E R V V R V P Q H T T V E V P V E K V I E R V V Q T H T R D I I E V P V E K V V E R V V R V P Q
301 E T I I E V P V E K V V E R I V T V P Q H T T V E V P V E K I V E R V V Q T H T R D V I E V P V E K
351 V V E R I V K V P Q E T I I E V P V E K L I E R V C R V P Q E T I V E V P V E K I I E R V S Q T H V
401 R D I V E V P V E K L V E R I V T V P K E T V I E V P V E K I I E R I V K V P Q E T I V E V P V E K
451 L V E R V C Q T H T
```

```
  1 R D I I E V P V E K I V E R V V K V P Q E T I I E V P V E K V I E R V V T V P Q E T I R E V P V E K
 51 V V E R V V Q T H T R D I I E V P V E K I I E R V V K V P Q E T I V E V P V E R V I E K V C S T H T
101 R D I I E V P I E K I I E R I V K V P Q E T I I E V P V E K C I E R V V T V H S T D V V E V P V E K
151 V I E R I V R V P Q E T I I E V P V E K I I E R V C E V Q V E K I V E V P V Q Q I V R V P V E V P V
201 E H I V E R V V K V P V E T I K E V P V E K I V E I I R P V E V I H H I E V P V E R I V E R V V E V
251 L K E V P V D R V V E R L V E V P V T H T V E V P V V S V V E K L V E V P K E I I R E V P V E K I V
301 E R V V T V N V D N Y I E V P V E K I I E R V V T V P V E K R I E V P V E V V V E R I K E V Q V E K
351 I V E V P V E K I I E K I V K V P V E K I V E V P V E C V V E R I V Q V P V T Q T V E V P V E R I V
401 E K I V E V Q V Q Q I V E V P V E K V V E R V V K V P V D H V I E V P V E R V V E R I R E V P V E K
451 I I Q V P V E K I K I V T V D R I V E V P V E V
```

```
  1 E K I V T V Q V E K I V Q V P V E K I V E K L V E V E K I V E R T V T V E R V V E V P K V V E K I V
 51 E K I V E V P R E Y I R E V T V T S I K E V P V E K I V E R I V E V P I T N V V E V P V E R V V E R
101 V V E I P L E R V V E R I V E V P V E K I I E R I I E V P V E R V V E H I I E V P V E R L V E R I V
151 E I P V E K I I E K I I E V P I E K I I E R V I E V E V P I E I E K V V E I E K I V E V P V V V E K
201 V V E V E K I V E V S G G V E I V E K I V E V E K I V E V P V V V E K V I E T E K I I E K G V V
```

```
  1 T E I V P E V S T E L Y S T S Y S T G S K G T S S S I S A S G G A S Y S V G G G Y G V S G G A S T G
 51 G G Y A V S G G T G G A G Y G S I G T S G G T G Y S S I T T G G S G T G Y S S I G G T G A G V G Y S
101 S A T T G G A G A G A G T G Y S S I S G S G A P G G A G G Y S S T S Y S S G T Y G A G G G T Y G A G
151 A P G A G G Y G S Y S S T S Y S G T G G S G S A P G R T G Y T A T S G S G T R S G S A T R S G S G S
201 R A
```

N.B Sequence submitted to PSPRED in 5 sectors. Longer sectors yield predictions of no secondary structure using both PSIPRED and YASPIN.

10

Euglena 15434_G
(First half of sequence)

Articulin

MTVEESRTFSQTPITTYQSGARAVSNTRILSTGATTLSSATSIVDGSRRYAR
SSAAVCCQPFDPCNSVVTRQLQTRYLDPVETTHVEKVPMEIEVDVEGYRE
VIEVPV

ERIVEVPV
ERIRTVPQMQVVEVPVNRHV
ERLVVVPEL
KERQVFIPKTI
ERVVKVPEVYEYEVPVQRTV
EKVVVVP
REVLVEVPV
DKVTTV
DRVV
EKHVPVEHIV
ERLVEQ
ERVVETQVKVQQIVEVP
REVIKNYEV
ERIV
ERVVEVPQL
KEVQVPV
ERVY
ERIVPVPK
RDTVQIPV
ERIH
ERIVPVPQTNIVEVPV
DKLI
ERVCTVPKQCTVEVQV
EKVV
DRIVEVPRTITQEVLIDMVV
EKVVTVPS
EKIIEVPV
EKVVESIVQIPKVVTHDVPV
EKVI
ERVVTVPHEQRIEIPIQRVV
ERVVQVPV
DRVV
ERVVEVPHVVT
REVPVESVV
ERVVTVPVNRVVEVPV
DRYV
DRVVQVPV
EKVVNRVVEIPKVIT
REVELQQIV
EKIVEIPTTKVVEVPVIRYI
EKIVEVPVT
REVEVPVQKYV
EKIVEIPY
ERVVEVQVEVPV
ERIV
EKVVEVPFTV
DRVVEISI
EKVI
EKIIEV
ERRVEVPVEVIV
EKVV

Euglena 15434_G
(Second half of sequence)

EKVVEIEVPVII
EKVVEI
EKIVEV
ERRVEVPFDTIVEV
ERVVEV
EKIVEIPIEVVKNV
ERVV
EKTIE

TSYDVTPVVEQKVSSFSAS**G**GGSYSTSASYSTGGGYASGGGGYVSGGGGGFT
SGGGGYVSGGGYSSGGGTGGSAFIGGGGSGGSAFISGGGSGGSAFISGGGSG
GSAFISGGGGSGSGSRVTGGFSSSGALPGAAGVTGTTQLYSSQTSSLSGSTGA
WGLPKQY

12

Euglena 22739_G

Articulin

…AASPVVDDLPGRPGGGSPELVAMAPALGSGVAGGQAVGDQGEVVDLMQIGSE
QEIVEALEVESFDSRAVHQVALLEEDSPLWRSPVKPNGVV

ERIVPV
ERLIEVPKVIHCVE
ERIVEVPIQRIV
ERLVPMEVTHI
KEVPV
ERIV
EKLVEVPVVTI
KEVPVETVV
ERVEVVTV
EKVV
EKLVTV
EKIVEVPTFVEVIHVKVIEVPV
ERVI
EKIV
ERPVPVESVV
ERLVEVPVQHVVQVPV
EKIV
ERIVEV
EKPVTVTH
EKIVEVPV
DRIV
EKVVTQEVPRIVEVPV
ERLV
ERLVEVPVVTI
KEVPI
ERVV
ERVEVITV
EKVV
EKVVPV
EKIVEV
EKVVRVVE
DRIVEVPVARVIQQVV
ERPVVVETRVETI
REVPV
REIVQIPVYEVI
ERI
KEVQVPYT
REVPVEHVV
EKIVEFVEVPIYV
DRIVEVPP

SVRSTSRAQQKISAHQRQGCGASRRNGSGRW

13

Euglena 54789_G

Articulin

…YVRHYTDLQPTHREYVEMVPVEVSVQEEIIQDIIEVPTFERRRRMVWEEFGG
AAPTVVPFRPVTPERPVPVPLQRPADTFAPVPVPHEGAQTVAPSERHGPVG
PAEHTLQVVRVPQVQRVEVPV

ERVV
EKVVTVPQVQRVEVPV
ERLV
EKVVKVPEVRIVEVPV
DRVI
ERRVEVPV
DRVV
ERVVKVPEVTTVEVPVETI
KEKIVTVPVQRTIEVPI
EKFV
ERIVKIPEVRTVEIPV
ERII
EKIVQVPI
EKIVEVPV
DRII
ERVIEVPVTKVV
EKVIEVPV
DRVVEVPV
EKIV
EKFVEVEVLV
ERIV
ERLVEVEVP
KEKII
ERIIEVPV
ERVI
EKVVEVYVPV
ERVVEV
ERIVEVPV
ERIV
ERIVEV

PYSEDLAGAQVPSEQMAEKGSEEATRSAQAQSGSHPVSHVSSEVSSSALPPVGSI
SSWMPGSSYASTQPSQSVASSAQSAGAVDVSTTWGPSGYRPAAARSGPSLAGY
VPSAGARSEPPGTAAGVVDFRQYSAQLAPEAEQQRF

14

Euglena 61926_G

Articulin

…FFSPFPLSFLSFTLQSADSQRYMSYSGAYRADYPVRSTSPRLSRPYPFSRPA
ERVVSPTRVVDGRYRSPSRLVSESCFPAQSTVVETTSACIRHTQEIVEVPVAQ
PVEVVRTVPMEVPTLEVVERARVQVQEHVIEVP

REHIV
EKIV
EKVVLKTIEVPVEHIV
EKIIEVPV
ERLV
ERIV
ERIVEVPQ
DRLV
ERVI
EKIVEVPVEYPV
ERVV
EKIVEVLVEIPV
ERIV
ERIVEVPVEVPV
ERIV
EKIVHVTTEVPRVQRQ
ERIIEVPVDHIV
ERIVEVPV
DRPISAIQ
ERLVPHTV
ERIVKYEVPV
DRVIEVPV
EREVI
REVHV
DRPVIHQV
ERPVVHTVTRPVIHEV
ERPVIHEV
ERPVIHEV
ERPVVHRV
ERPV

AVPVSVVRQEPAVCYETAPYRQRYVNGTTTYSTRAPR

15

Eutreptiella CAMPEP_0113738536

Articulin

MPIPKTARDVAAALDNADGVADGKFHGLPIVVGDSRPSSRNVYSSPRSTRYV
SDEFIRTGLPATRVGYPATRVGYPATRSRYMSGPVIHHHEPPTIVRHSTAPSA
SRIIRSEGPLVRRERIDQATSRTHIEKRVVPVTRVIEESVEPVRRFVQPVERRV
EVQPVQTVERIVEVPVERRVVDVQQVEHVVEVEVPVPVEVTKEVKVEVPVEK
PVYVEHVVEKIVQVDVNHETQVPIEKIVETLRVEEVVHIQEVPVQQTVEKLVTV
EVPHIIEVPV

EKVV
EKIVEV
EKII
ERIVPV
EKIV
EKEVILKIVE
EKIIEVPV
ERVKHV
EKIVEVPKIIETIV
EKIVEVPVKHVVEVPV
EKIV
EKLVQVPVMYT
KEVPIETVV
EKEIPGPV
REV
EKIVQVPV
EKLVEV
EKIIEVPVQVPV
EKIVEVPVEVPV
ERLV
EKLVEVPV
EKIVEVMV
EKVIEVPVEVPV
ERII
EKVVEVPVHV
EKIVEIPLEI
EKIV
EKIIEVEIPVEIPVEI
ERIV
EKLVEVPVVV
EKVVEVEIPIEI
EKIIEI
EKIVEIPIEI
EKIVEI
EKIIEVEVPIEI
EKVIEV
EKIVEVPIEI
EKVI
EKTIEV

GTEPTTVTKYSSFSTSSAPIETKRFSSQGMSRSSTQSSSTTPGGEIQYSSSAGSAESTGT
GTGWGTPGTNRSAWLGNMNN

16

Eutreptiella CAMPEP_0113740288

Articulin

MTVTITPTMTIQQTPGRFTX

EREVGYRKLH
RDVLQEIHEVLV
EKPVEVIQTEVMEVPVKHIE
ERVVAETH
ERIVEVPV
EKIV
ERVV
EKVVVKTVEVPV
ERIV
EKIVEVPV
ERIVEVYV
EKIVEVP
RDKIV
ERIV
EKFVEVPQIV
ERIV
EKIIEVPVDYPV
ERVV
EKLVQVPVEVPV
ERIV
ERIVEVPVEVPV
DRIV
EKIVEVEVEVPVIQ
DRERVVEVPTDNI
REKVVEVPV
ERLVEVPS
EREVVKQVQVPVEIPVEVEPEVDVVIV
DKAV
EKT
ERPKRITLADQNYVPPRYSL
DKTY
KERSS

ASYQYTAPEKSVEYVTHAPSEKRYTRSSSGQVVHEYAAPAKSVEYVTS
GPERGQVLREYTREYYEDPAHEGDYRISSGRHGTAYEAARALDAADGRLDG
KYQGKDIYVNGEKVRSGGXACCLRRPQPRLHKSQPAGEQQVAVAITRVQAXV
TMCERLSILPRSPSLPPQQGHLVLAGDLHLPLYTQKKQVRDRVNKATGTGWV
MAIGVNQRQVKGQPSSAIRVLRMSSKKHSMGSAEPTGSCRVEGENKHSEVR
GRALESDNSPKHHHLPALTSNQAIFFRNEWLQSSWWANVVSGESLC

17

Eutreptiella CAMPEP_0113741250 (First half of sequence)

Articulin

…GAPHSAPISSNCTIDNMSSRVTTGSASGGRVRYDGGSQMRVRDPKISVTE
TGTPGNEVIVVSAERDVVPTGTVTSSTYQGGSTIVRERGYPVTSVGHSYFRS
GSPVAVGGSSVARSAVRVGQVQAGGAYYRQGGRALYSQSVRRELLDEATE
TTMI

EKVPIEIDVEVDNY
REVVEIPV
EKIVEVPYPV
ERVVEVPVEHTV
EKIITVPEL
REIEIPV
ERIV
EKVVRVPQETLVEVPV
EKVI
ERVVRVPEVTEIEVPV
ERIV
EKVVRVPEVHEIEVPV
EKII
ERVVNTHS
RDIIEVPV
ERVI
ERVVEVEV
EKIVHVPV
EKVVTVPHEVVVNSVV
EKIVTVEVETI
KEVPVESVVEVIRPVEVTRHVEVPVEHIV
ERIVEVV
KEVPV
ERV
REKIVQVPVTNTVEVPVVSVV
EKLVEVP
REIV
REVPV
EKIV
ERIVQVPVDNYIEVPV
EKSV
EKLVQIPV
EKTVEVPI
ERVV
EKLVEVPVETIVEVPV
EKL
RESIKVVTV
EKLIEVPV
ERVV
EKIIQVPVEHSVQVPI
ERIV
EKIVEVQVQQIIEVPV
EKEV
ERVVKVPV
EKVVEVPI
EKIV
ERIVQVPV
EKIIEVPI
EKVVEVIKVVQN

18

Eutreptiella CAMPEP_0113741250
(Second half of sequence)

ERIVEVPVEV
ERLVSVPV
EKIIEVPL
ERIV
EKAVPV
DRII
EKVVEV
ERIVEVPMIV
EKFV
DRTVTV
EKIV
EKIVEVPVTNVVEVPI
ERTV
EKVVEVPV
EKIV
ERIVEVPV
EKIVEV
EKIVENIIEIPI
ERVI
EKIIEVEIPIEI
EKIVEI
EKVVEVEIPIEI
EKIVEI
EKIVEVEIPIEI
EKIVEI
EKIVEIEVPVIV
EKVVEI
EKIVEVPIEI
EKLV
ERTIVE

TAPTVETTVTTETVEVPR**G**AGYSSYSSSSSFSSGSRGAGGGGGGAAGGGSGGSTSYSTWSSQSGX

19

Eutreptiella CAMPEP_0113737346
(First half of sequence)

Articulin. Head domain a perfect repeat, followed by a bit of a third repeat, followed by a novel sequence (blue font)

…NDGLFAEPDHTLSQLALPGPPGGEAMAVATQRLAGKLKATVSPKHLVFS
KLIPALKRACKRSDLLEEMEADGLTTIPPPSDWVLAKMAREWRCWLPSGP
GYFDYASQPVPWELVGGRHWPNSRGDFDERDTGHEGFSTQDFLNRPEAK
AAGLTAAEVIALRLYTGPGYIPINRSLRVNSGRFAVTQWALDCAIGKLALAERE
GLLLRGLRLLPKEEWQQQYEDCRCADDAMDLWISDPAYSSTTTDMAVATGT
DFGGPCTFVFHAQCDLSPADGLIGNAASVQWVSQYPDEVERLLPSNSCFLSL
PQGMRSELPEGMGDRKIFQFFSRFLWDYERCCPPVVTEVEEYVVRINEVMW
HVYRGLGSEKPPPEVTADAELTAFFLAPDPVGVDGMDGGLPEVLEEEM

NDGLFAEPDHTLSQLALPGPPGGEAMAVATQRLAGKLKATVSPKHLVFSKLI
PALKRACKRSDLLEEMEADGLTTIPPPSDWVLAKMAREWRCWLPSGPGYF
DYASQPVPWELVGGRHWPNSRGDFDERDTGHEGFSTQDFLNRPEAKAAG
LTAAEVIALRLYTGPGYIPINRSLRVNSGRFAVTQWALDCAIGKLALAEREGLL
LRGLRLLPKEEWQQQYEDCRCADDAMDLWISDPAYSSTTTDMAVATGTDF
GGPCTFVFHAQCDLSPADGLIGNAASVQWVSQYPDEVERLLPSNSCFLSLP
QGMRSELPEGMGDRKIFQFFSRFLWDYERCCPPVVTEVEEYVVRINEVMW
HVYRGLGSEKPPPEVTADAELTAFFLAPDPVGVDGMDGGLPEVLEEEM

NDGLFAEPDHTLSQLALPGPPGGEAMA

ASSLN**G**LPTFCAVSQEDPVAVETRIEVPTVEKVTAVQAEDIQEVPMVEVPV

ERVIEVPV
EKII
ERI
ERIPVETIREVPVEV
EKIVHVPVEHVV
EKVVEVPV
ERVV
EKVLQVPVEV
EKVV
EKVVEVPV
EKIV
EKVFQVPVEV
EKIIHVPVEHVV
EKVVEVPV
ERVV
EKVLQVPVEV
EKIIHVPVENVV
EKVVEVPV
ERVV
EKVLQVPVEV

20

Eutreptiella CAMPEP_0113737346
(Second half of sequence)

EKVV
EKVVEVPV
EKVV
EKVEKVPVEV
EKIIHVPVEHVV
EKVVEVPV
ERVV
EKVLQVPVEV
EKIIHVPVXXVV
EKVVEVPV
ERVV
EKVLQVPVEV
EKVV
EKVVEVPV
EKVV
EKV
EKVPVEV
EKIIHVPVEHVV
EKVVEVPV
ERV

21

Eutreptiella CAMPEP_0113744366

Articulin. Predicted secondary structure next slide

MAEARTGPSAMDALEDADRRTATRQTRSAAEALDAVDGVMDGKFFGRPIVAT
GPSRVVGGRSVVDSIDAFDGRRYRSSGGVHGSAAEALDAADGVMDGRFYGR
PIVETRSPQRLRGVTAQALDAADGVIDGRFYGRPIVETRGATTRYDDHVEVRR
VH**GG**TRHRSKVVEVPVYHHENRIPVEVQVPVQIPVEVPVRI

ERPLAI
ERVVEVP
REVIKHVEVLVEVPHEVKVPYAV
EKVI
ERIVENVVEVPGPTKVV
EKIVEV
EKIVEVEVQV
ERI
REVKVPYPV
ERVV
EKLV
ERTYPVDRIVEVPV
ERIVHDHVDVP
RERIV
ERIVEVPYPV
EKIV
EKISEVADVRVV
EKVVQIPV
EKVV
EKVTTVPI
EKIVKVPI
EKPVIKLVH
KEVPVEHIV
EKIVEVPV
EKIIEVEVDVPV
EKVV
EKVTRIPVEIPI
EKFVEVPI
EKIVEVPVEHVV
EKIVEVPV
ERII
EKLVKVMVPVPGPPHENIIY
RDREVPI
EKRVY
RDRIW

GGRQRGYEMPPPPPPPPRDVQWISVPEAYPHQTWEWDRWQNWWDGRYGSNMQY
DGGYNAGYPPAAPQSYSNMQYDGGYNAGYPPAAPQSYRYGA

22

Eutreptiella CAMPEP_0113744366

Predicted secondary structure

```
  1 M A E A R T G P S A M D A L E D A D R R T A T R Q T R S A A E A L D A V D G V M D G K F F G R P I V
 51 A T G P S R V V G G R S V V D S I D A F D G R R Y R S S G G V H G S A A E A L D A A D G V M D G R F
101 Y G R P I V E T R S P Q R L R G V T A Q A L D A A D G V I D G R F Y G R P I V E T R G A T T R Y D D
151 H V E V R R V H G G T R H R S K V V E V P V Y H H E N R I P V E V Q V P V Q I P V E V P V R I E R P
201 L A I E R V V E V P R E V I K H V E V L V E V P H E V K V P Y A V E K V I E R I V E N V V E V P G P
251 T K V V E K I V E V E K I V E V E V Q V E R I R E V K V P Y P V E R V V E K L V E R T Y P V D R I V
301 E V P V E R I V H D H V D V P R E R I V E R I V E V P Y P V E K I V E K I S E V A D V R V V E K V V
351 Q I P V E K V V E K V T T V P I E K I V K V P I E K P V I K L V H K E V P V E H I V E K I V E V P V
401 E K I I E V E V D V P V E K V V E K V T R I P V E I P I E K F V E V P I E K I V E V P V E H V V E K
451 I V E V P V E R I I E K L V K V M V P V P G P P H E N I I Y R D R E V P I E K R V Y R D R I W G G R
501 Q R G Y E M P P P P P P P P R D V Q W I S V P E A Y P H Q T W E W D R W Q N W W D G R Y G S N M Q Y
551 D G G Y N A G Y P P A A P Q S Y S N M Q Y D G G Y N A G Y P P A A P Q S Y R Y G A
```

23

Eutreptiella CAMPEP_0113747498
(first half of sequence)
Articulin. Predicted secondary structure next slide

…PQVTE

REVVV
EKVV
EKILRVPEIHEFQVPI
EKIV
EKIVEVPV
EKVVEVPV
KEVVVEVMEV
EKIV
EKLVVV
ERIV
ERPVEV
EKIIEVPKVV
ERVVHKMVEVPVEVT
REVPV
EKIV
ERIVEVPNIT
EKLVVV
EKIVEVPKVV
EKVVVL
EKIVEVPKVVEQVVVV
EKIVEVPKVI
DRVV
EKSVVV
EKIVEVPKVV
EKLI
EKVVVI
EKVIEVPKVV
EKLV
EKIVVV
EKVVEVV
KEVKS
KDLIEVPV
ERVV
EKVMEVSNTHVVEVPI
EKII
EKVVEIPIEVVVEHVVEVPV
EKVVKFPV
EKIVEVPVEIYI
EKII
EKVVEVEVPVEV
ERILEV
EKAVEV
EKLVEVPI
EKTIEVMKTV
EKIVEVPV
EKII
EKIV
EKVVEI
EKVVEVPV
EKVVEI
EKVTH

24

Eutreptiella CAMPEP_0113747498
(second half of sequence)

SITQIPPAIETTFSFERESQSE**G**RHQYADVSSGESVERYHSSMSSSTHGERY
QVVESMTTSSPSIVSGGATSPRVGQSIRVADTTNYGQSSSVMSTTSSERDGP
MEVVEGSPRRYFSSISPRGTSEYKSIKAGAI

```
  1 P Q V T E R E V V V E K V V E K I L R V P E I H E F Q V P I E K I V E K I V E V P V E K V V E V P V
 51 K E V V V E V M E V E K I V E K L V V V E R I V E R P V E V E K I I E V P K V V E R V V H K M V E V
101 P V E V T R E V P V E K I V E R I V E V P N I T E K L V V V E K I V E V P K V V E K V V V L E K I V
151 E V P K V V E Q V V V V E K I V E V P K V I D R V V E K S V V V E K I V E V P K V V E K L I E K V V
201 V I E K V I E V P K V V E K L V E K I V V V E K V V E V V K E V K S K D L I E V P V E R V V E K V M
251 E V S N T H V V E V P I E K I I E K V V E I P I E V V V E H V V E V P V E K V V K F P V E K I V E V
301 P V E I Y I E K I I E K V V E V E V P V E V E R I L E V E K A V E V E K L V E V P I E K T I E V M K
351 T V E K I V E V P V E K I I E K I V E K V V E I E K V V E V P V E K V V E I E K V T H S I T Q I P P
401 A I E T T F S F E R E S Q S E G R H Q Y A D V S S G E S V E R Y H S S M S S S T G E R Y Q V V E S M
451 T T S S P S I V S G G A T S P R V G Q S I R V A D T T N Y G Q S S S V M S T T S S E R D G P M E V V
501 E G S P R R Y F S S I S P R G T S E Y K S I K A G A I
```

Eutreptiella CAMPEP_0113748194

MSIYVGEQRYSSPQQYAAERQRYVSEPRPLDHIRVADAGYKYGTGDSKVVQG
KRVRSQVVNVPVVHHDVRVPVEVEYPVEVPVEVPYEVSIPREVIREVTVPVERI
VESVN

ERKVI
KEIPVPQEFETIV
EKVV
EKRVSVPGPVKYV
EKIVEV
EKIVEV
ERPVWRI
KEIPVPYPV
EKVV
EKIVNVPKYI
DRKVEVPQDRLVQVP
REREYI
REVV
KEVRVPYPV
ERIV
EKVVKVPQ
ERFV
EKKVPFIV
EKVVEHV
EKVEFT
KEVHVTV
EKEVIEEVEVRVPV
EKVI
EKRVPKII
EKVVEI
EKPRIVQKIV
EKTVHMPY
EKIT
EKTIEVPI
ERHVHVPV
ERVI
EKQIEVPI
ERVV
EKVVQVPQEVD

GGDVDKVVWRDIPVFIDKVVYNDYTESGHLFAYHNMPTPPPPLPNFKWVPYSYNALTLGT
GRSAKGGPPLARSLVPPRGPPVPARLPGGRVGTIGRLPLPLGDARXXGNLGQLNIIDRFIF
VDPEGNVVDERADLWTERGLDPNIMHQKVSAAQAAGFHRSDKFWEHHFDELAHAAAQR
PIVDTQHFDHPVELNYHGHVSPTGRAYYPXLLAGGTATGTRVPTSLPAVLTVSLRGAASAL
RVWLPTDEDSGVAGALAGTGLLAAPLGPRAFDSAAPNMDEEAVCGGGAPPHPVGQPEP
REEGSX

26

Eutreptiella CAMPEP_0113750188
(First half of sequence)

Articulin

MHAAAPRTPPRQGGSVTRPGSGVXXXXXXPHPHQLSGGVPCREAGSKNASHDGMV
MQVVQESSQMGSRLAPSYNRGYLTSQEVAVARSRGSTMPSSQSSSLTRSDVIVVDE
GRTYYDSGMLSHPGSRVRHGGYS

ERSVV
EKREIEIEVEVDNI
REIVEIPV
EKIIEVPV
ERVV
EKIVQVPQVT
ERDVLV
EKVV
ERIVQVPVETRVEVPMVQTI
EKVMAVQVENV
REVPV
EKYR
EKLVEVPV
ERVIEVGV
EKLV
ERVVQVHVDNI
REVPV
EKVI
EKLVQIPVETI
REVPV
ERIV
EKVVEVRVPVPV
ERVI
EKIVEYPVQNI
REVVV
EKVV
ERI
REVPV
ERLVEVPV
ERIV
ERIVEVPIHNI
KEVPVVRLV
EKVVQVPVEIPV
ERVI
EKIVKVPVENI
REVPV
EKIV
ERVVEVPV
ERVV
ERVV

27

Eutreptiella CAMPEP_0113750188
(Second half of sequence)

EKQVPVEV
EKIVPVENIV
EKLVTVQVEHI
KEVPV
EKIV
ERIVEVPV
ERVV
EKIV
EKVV
ERVVEIPIEI
EKIV
ERVEVVEVPIEI
EKII
ERTVEVEVEVPVIV
EKVVEI
EKIVEVPIEI
EKIV
EKTIVE

SV**G**SAPSASVTVTTETSSSASGYSSGGSGYVAGSWSSASKEQAAPGGANGYS
AFSNSAHRHANMLAALDAADGKLDGQVFSADVKTILPGSAPTAYVPPPTTRDKL
AXEGQPQCQPIHRQPPVWLQQFRDPNPRIRVDS

28

Eutreptiella CAMPEP_0113750414
(First third of sequence)

Articulin

XVVMSGATQHPSYATYPSLQAPTYVGLASSAHHAAAEYRGPYASLQNTVEAS
GIGALMPATISGPPPLLPVTAQYQPPSGQEVGQGWMEEQSTVTPGNMQVESL
TEISTHASKAKKSNKVSSGSGRS**G**LVRYVEVPV

EKVV
EKVVEV
EKIV
EKAVPV
ERIV
EKVVEV
ERIV
EKAIPV
EKIVTIPQVTT
REEKIV
DRVVKVPQKHEVVVPVEHIV
ERVVQVPTTV
EKVVVV
EKVV
ERPVEIPVATEHIVVV
EKVV
ERVVRVPEVLEVPVEHIVKQI
EKVPEVRTVEVPV
ERII
ERLVEVPQVT
EKEVPV
ERIV
ERIVKVPEVHTVEVPV
ERLL
EKLVEVPLVV
EREVPV
ERLV
ERIQLVP
EVRTVEVPVEVV
RERIVKVPEVHTVEVPV
EKYV
ERLVEVPQIIE
KVVVNTV
ERIQTVPEVCTIEVPV
EKRIETINKVPMETI
KEVPV
EKII
ERIVEIPQLTTIEVPV
EKVV
ERVQQVTVQNI
KEVPV
EKXV
ERIRKVPMVTI
KEVPV
ERIH
EKIVEVPQVLTIEVPV
EKVV
ERVQQVVVQNI
KEVPV
EKLV
ERIQKVPVETI

29

Eutreptiella CAMPEP_0113750414 (Second third of sequence)

KEVPVENII
EKVVQVITQNV
REVPV
EKTV
ERIMKVPVETV
REVPI
EKTI
ERLQKVPMETI
REVPV
EKII
EKIVEVPLVI
ERAIPV
EKVV
EKIVEVPQLTTI
ERPVETII
ERVVQVNTENI
REVPV
EKTI
ERVHKVPMETI
KEVLV
EKVV
EKIVEVPQVI
ERAVPVETIV
ERILEVQVQNI
KEVPV
EKVV
ERVVQVPVEII
KEVPV
EKVMTVEVAKIVHQPVEVI
KEVPI
EKVVKVPEVHV
KEVPV
EKVI
ERIVQVPVETV
KEIPV
EKIVNVEVAKIVNVPVEVL
KEVPV
ERIVKVPEIHV
REVAV
EKLI
EKLVEVPV
EKII
EKHIYIDRHVEVI
KEVPV
EKIV
ERIVEVP
KETVKMVVV
EKVV
ERVVEVP
KETVRSVVV
EKVV
EKVVKVPQLHEIEVAVEQIY
EKIVEVPCETT
KEVPI
EKII
ERIVEVPV

30

Eutreptiella CAMPEP_0113750414
(Final third of sequence)

ERII
EKAVTV
EKIVEVPVRI
ERTVEVPVTEIVEV
EKLVYVNVP

HHAEGPVQMDDRVSPFDWSCAFVDCSNHVVPGYRFCATHQVDHGLSRSANSL
NIPIPHVGVPEPHKRTEKDYQALADMIREDGERRRSANSSSISQAHSSASSFSSIT
RMSGATKTKSYEGIKSEPIIARLTTDDGTRTINIPMHRVVVLGSSSELSLEYRFATF
EPQHCSLSSTSAGIVLRDLSDSGVFVNEKAVGKQVSVMLQDKDRVAIGKARVSF
VFHPT

31

Eutreptiella CAMPEP_0113750944

Articulin

…PQYSVQPLQMKQAEYQPAIYSMPQMQQAPAPVQENRVEYKEVIKEVEVPV
PVEVPVYQDEPEYYEVPEYVRVEVPVPHEVPRYVEVPQPYTV

EKVVEVPEPYEVV
KEVTVPVNVTYEVEVVETVNVPYPVEQVV
EKVVVVP
KEQIV
EKVVEVPV
EKVV
EKVVEVPQSVSVPYVVEIPVPYET
EKIIEVPKPYEVIKYIEVPTPVTKEV
EKIV
EKRIPNYVKV
REEIKVPSRRPKLWIKNVPV
ERV
ERVEVPYKVHKYVDVRKPYEVIKKI
EREV
EKVVEVKVL
KEKAVPHKI
DKIVEIPLPYYV
EKIVDVPQPYTVKKII
EKEVEVPYTVLV
KEEVRKPYEVTKVVNRPVPQKV
DKVVLKYV
EKPFAQHYEVRVPKYYDVPVPYEDIKY
RDVPFPV
EKLV
DKVVEVPVP
REVPFKVFNEIPVPV
EKIIEVPKPEPYDTVV
EKEVIVYQ

HEPHEVIQEIELVEHIVHPIEIIEEQVIKRKIKKLRKKVYGEPVLCDAPFTGQPVDQAPPDGK
SGKSGKSGKSKKGTGATNPQIESGPPLAFPAAAYGMPGMPGMPGMPGMPLFGMPMPY
GVPMAF

32

Eutreptiella CAMPEP_0113750946

Articulin

XFFRLGVSMTTYEELKARREREKRLFEVPSDVLSDRTTERRVVQPAYVQDD
MGALAIRARPWTSQSPSTGELSCLPITLCPXSVLS

REVPIQVPVEVP
REVPI
DRVFKHT
EKIPVPVR
RERKIRVPVTKIVKKPVTKYVDVPVEVIT
EKVLIKRKKKIVEVPV
ERIV
EKPV
EKIIEVKV
EKIVEVPV
ERVV
EKKVNVPYEQVV
EKVVNVPVEILVNKVV
EKVV
EKIVEVPVEQVV
ERTVEVTI
ERIIQKNIEIPV
ERVQENIITIPIIKII
EKVVHVPV
EKII
EREVPVQV
EKIV
EKYDT

SDPIYVDKTVNVVVEPTVHTTTTYETEYNYDVDGDWGGYGDNTEVTTEVITTTEY
DGDWGGDAGVTTTTTEYTYGDDGGYGGDDGYGGY

33

Eutreptiella CAMPEP_0113753042

MPEPE**G**SPADLQAADDIEIDVSVETLVHVDQRQVVDKPFEVSIEHLEEVPVE
QVLESVN

ERLVL
REVRVPI
ERVV
ERVVGVAIEVPY
ERVV
ERVVEVPV
EKVVP
KEIVKVI
EKIVEVPVTRVLKTKKITPV
ERIV
EKRVEVPTETTVIRRVEVPYTRIV
ERFEEVII
DKIV
EKRVEVFV
DKIV
EKIVHVPHDVII
EKVVEIEEEEEIVEVPV

THHETTYLDVPQRITLQDQ**G**YKPPKAVILNHRQPVPLPTQHQPHMHHHQPTQHPIT
PIMLQQPYPPTPVTALHTDWLRDRVQAAEEKNHWLQGRRYEPLSHDVEAPVRRLS
APDPGPGSGHRMHGSTAVHEPPYAAPSSVSSRTRSRSADQYAA

34

Eutreptiella CAMPEP_0113754010
(First half of sequence)

Articulin

...PEVTADAELTAFFLAPDPVGVDGMDGGLPEVLEEEMNDGLFAEPDHTLSQL
ALPGPPGGEAMAVATQRLAGKLKATVSPKHLVFSKLIPALKRACKRSDLLEEM
EADGLTTIPPPSDWVLAKMAREWRCWLPSGPGYFDYASQPVPWELVGGRHW
PNSRGDFDERDTGHEGFSTQDFLNRPEAKAAGLTAAEVIALRLYTGPGYIPINR
SLRVNSGRFAVTQWALDCAIGKLALAEREGLLLRGLRLLPKEEWQQQYEDCR
CADDAMDLWISDPAYSSTTTDMAVATGTDFGGPCTFVFHAQCDLSPADGLIG
NAASVQWVSQYPDEVERLLPSNSCFLSLPQGMRSELPEGMGDRKIFQFFSRF
LWDYERCCPPVVTEVEEYVVRINEVMWHVYRGLGSEKPPPEVTADAELTAFFL
APDPVGVDGMDGGLPEVLEEEMNDGLFAEPDHTLSQLALPGPPGGEAMAASS
LNGLPTFCAVSQEDPVAVETRIEVPTVEKVTAVQAEDIQEVPMVEVPV

ERVIEVPV
EKII
ERIE
RIPVETI
REVPVEV
EKIVHVPVEHVV
EKVVEVPV
ERVV
EKVLQVPVEV
EKVV
EKVVEVPV
EKIV
EKVFQVPVEV
EKIIHVPVEHVV
EKVVEVPV
ERVV
EKVLQVPVEV
DKVA
EKVVEDVV
EKVVEVPV
ERVV
EKVLQVP

35

Eutreptiella CAMPEP_0113754468
(Second half of sequence)

XKVLQVPVEV

EKIIHVPVENVV
EKVVEVPV
ERVV
EKVLQVPVEV
EKIIHVPVEHVV
EKVVEVPV
ERVV
EKVLQVPVEV
DKVA
EKVVEVPVEQAVI
RDVPV

EKTVEMIVEGPNMLDAAIGLAEEGKPFVFEAANGYGLTARHVEAGQQLKNKR
ADNDRWRLHRMSPGEFTVESLSAPGLRLGVGNQSGHGFKAVLVPEGDQRA
LLRLVPARDGSQQRVSFESVSQPGSLLNHCNGLMWFFDRPANQHFSNDSSW
VLLSSEKENSKTSRRGGTAQLRLEGKYLNLDESSGFTIRPRAGQTIVQPVTFT
SAQDVYPGHNPHTWTLKGTNDXGGMGITSGWGLSLEGSGFREYIRFENWKA
YRMYRVTPPPGPELHPRKVDADRAVRRPRPGRRQPPAKKKRERKREEGLSQ
GWX

36

Eutreptiella CAMPEP_0113754992

Articulin

MAEARTGPSAMDALEDADRRTATRQTRSAAEALDAVDGVMDGKFFGRPIVAT
GPSRVVGGRSVVDSIDAFDGRRYRSSGGVHGSAAEALDAADGVMDGRFYGR
PIVETRSPQRLRGVTAQALDAADGVIDGRFYGRPIVETRGATTRYDDHVEVRR
VH**GG**TRHRSKVVEVPVYHHENRIPVEVQVPVQIPVEVPVRI

ERPLAI
ERVVEVP
REVIKHVEVLVEVPHEVKVPYAV
EKVI
ERIVENVVEVPGPTKVV
EKIVEV
EKIVEVEVQV
ERI
REVKVPFLTXV
ERVV
EKLV
ERTYPV
DRIVEVPV
ERIVHDHVDVP
RERIV
ERIVEVPYPV
EKIV
EKISEVADVRVV
EKVVQIPV
EKVV
EKVTTVPI
EKIVKVPI
EKPVIKLVH
KEVPVEHIV
EKIVEVPV
EKIIEVEVDVPV
EKVV
EKVTRIPVEIPI
EKFVEVPI
EKIVEVPVEHVV
EKIVEVPV
ERII
EKLVKVMVPVPGPPHENIIY
RDREVPI
EKRVY
RDRIW

GGRQRGYEMPPPPPPPPRDVQWISVPEAYPHQTWEWDRWQNWWDGRYGSNMQY
DGGYNAGYPPAAPQSYSNMQYDGGYNAGYPPAAPQSYRYGA

37

Eutreptiella CAMPEP_0113757424

Articulin. Repeat in ABD domain

MSYEEYGGYGDAEGYGEATTESWTEGYGEGGGEAYAEGYGEASGAAYGEASG
AAEAYGEASYSAYGEASGAAEAYGEAYGDGGGEEYTHTDSWVEHSGGGDGGG
AEQWDQHEWTEEHEWGEPGGYTSEYKMEALPDVDLGGYSEFTPTTDTYDGQA
GVISSSYQVEGEGQKVYTDWSYQEPTPIKEYVPYVSPEIEPEGEKVVEMIHGEPV
LTETKILAENQLDEVIVSVDLLSEGKVSEVETGRRIVSKQRVIENRVEQVVQVPVET
FEEHLVVN

EKPKPVRVNV
DRLVA
KDV
DKIIY
KEVKVPIEVK
KEVVVKQEVEVPV
EKVI
ERIRKVPC
ERII
EKVV
EKVV
EKIVEVPV
ERIVIEEEEIFV
EKII
EKIIEVPVPKIV
EKVIKVPV
ERIV
EKVIKVPV
ERIV
EKIVEVPV
ERIV
EKIMEVPVENII
EKTVEVLVPSRKQHYVEQVVVE
ERPVEYT
RERAPVYVE

Repeat

ERAPVYVE
ERAPVYVE
ERAPVYVE
ERAPVYVE
ERRVE
ERRAPVYMK
ERAPVYVE
ERAPVYVE
ERAPVYVE
ERRVE
ERHPVYVE
ERAPVYVE
ERAPVYVE
ERGPVYAK
EREPIY

TSSTYGTRRYADQEPVYANETSASPIYVDERSAGAYY

38

Eutreptiella CAMPEP_0113758370

Articulin

XFFRLGVSMTTYEELKARREREKRLFEVPSDVLSDRTTERRVVQPAYVQDDM
GALAIRARPWTSPSRDFVGHDKTVSVYGGTVVPSHNTVSTSVLS

REVPIQVPVEVP
REVPI
DRVFKHT
EKIPVPVR
RERKIRVPVTKIVKKPVTKYVDVPVEVIT
EKVLIKRKKKIVEVPV
ERIV
EKPV
EKIIEVKV
EKIVEVPV
ERVV
EKKVNVPYEQVV
EKVVNVPVEILVNKVV
EKVV
EKIVEVPVEQVV
ERTVEVTI
ERIIQKNIEIPV
ERVQENIITIPIIKII
EKVVHVPV
EKII
EREVPVQV
EKIV
EKYD

TSDPIYVDKTVNVVVEPTVHTTTTYETEYNYDVDGDWGGYGDNTEVTTEVITTTEY
DGDWGGDAGVTTTTTEYTYGDDGGYGGDDGYGGY

39

Eutreptiella CAMPEP_0113758614

Articulin

MTTTTGAAARALDLADGVEDGKFYGRRIVEGNSRVLTGSRVYTRSPSRSVVR
TEGSVIRALDAADGVIDGKFYGSRIVDGARLGYSTQTEYGSRIVDGSSQVYSSI
HRPRYSSITR**G**ERRVVDVSSEATYKQKRVEVPVVHHETRVPVEYEVPVHIQVE
VPVRVEVPYPV

ERIVEVPQDVVQ
ERQVIVDVPVEVRVPYPV
ERVRHVPV
ERIIEVQGPTRFVENVVHV
ERLVEVPIEVPVVRTV
ERPYPVEQIVEVMVEQPVPVEHIVQVPEEYIVEEIIEVEQ
ERLVEQVVEVPVPVEHIVEQRVPVPVPHIVEEIVEVPVENIV
EKLIQIPV
ERV
REVPVEQIVRQARYV
DRPYEVL
REKVVHVPV
ERVRHVHVDVPV
ERIV
EKIVEVPFEVPV
EKVVQVPV
ERVVQVPVEHVV
ERPV

TRYSAPTYAAPPLRPVHHNPAFALDAAD**G**RIDGQYFGARIAQPYNPALALDAADG
RIDGTYYGSTIAPQPYGPGYRPY

40

Eutreptiella CAMPEP_0113766106

MSIYVGEQRYSSPQQYAAERQRYVSEPRPLDHIRVADAGYKYGTGDSKVVQG
KRVRSQVVNVPVVHHDVRVPVEVEYPVEVPVEVPYEVSIPREVIREVTVPVERI
VESVN

ERKVI
KEIPVPQEFETIV
EKVV
EKRVSVPGPVKYV
EKIVEV
EKIVEV
ERPVWRI
KEIPVPYPV
EKVV
EKIVNVPKYI
DRKVEVPQ
DRLVQVP
REREYI
REVV
KEVRVPYPV
ERIV
EKVVKVPQ
ERFV
EKKVPFIV
EKVVEHV
EKVEFT
KEVHVTV
EKEVIEEVEVRVPV
EKVI
EKRVPKII
EKVVEI
EKPRIVQKIV
EKTVHMPY
EKIT
EKTIEVPI
ERHVHVPV
ERVI
EKQIEVPI
ERVV
EKVVQVPQEVD

GGDVDKVVWRDIPVFIDKVVYNDYTESGHLFAYHNMPTPPPPLPNFKWVPYSYNALTL
GTGRSAKGPVPARLPGGRVGTIGRLPLPLGDARMLGNLGQLNIIDRFIFVDPEGNVVD
ERADLWTERGLDPNIMHQKVSAAQAAGFHRSDKFWEHHFDELAHAAAQRPIVDTQH
FDHPVELNYHGHVSPTGRAYYPXLLAGGTATGTRVPTSLPAVLTVSLRGAASALRVWL
PTDEDSGVAGALAGTGLLAAPLGPRAFDSAAPNMDEEAVCGGGAPPHPVGQPEPRE
EGSX

41

Eutriptella CAMPEP_0113768498

Articulin

MAEARTGPSAMDALEDADRRTATRQTRSAAEALDAVDGVMDGKFFGRPIVAT
GPSRVVGGRSVVDSIDAFDGRRYRSSGGVHGSAAEALDAADGVMDGRFYGIV
ETRSPQRLRGVTAQALDAADGVIDGRFYGRPIVETRGATTRYDDHVEVRRVH
GGTRHRSKVVEVPVYHHENRIPVEVQVPVQIPVEVPVRIERPLAIERVVEVPRE
VIKHVEVLVEVPHEVKVPYAV

EKVI
ERIVENVVEVPGPTKVV
EKIVEV
EKIVEVEVQV
ERI
REVKVPYPV
ERVV
EKLV
ERTYPV
DRIVEVPV
ERIVHDHVDVP
RERIV
ERIVEVPYPV
EKIV
EKISEVADVRVV
EKVVQIPV
EKVV
EKVTTVPI
EKIVKVPI
EKPVIKLVH
KEVPVEHIV
EKIVEVPV
EKIIEVEVDVPV
EKVV
EKVTRIPVEIPI
EKFVEVPI
EKIVEVPVEHVV
EKIVEVPV
ERII
EKLVKVMVPVP

GPPHENIIYRDREVPIEKRVYRDRIWGGRQRGYEMPPPPPPPPRDVQWISVPEAY
PHQTWEWDRWQNWWDGRYGSNMQYDGGYNAGYPPAAPQSYSNMQYDGGYN
AGYPPAAPQSYRYGA

Eutriptella CAMPEP_0113754468

Articulin

XKVLQVPVEV

EKIIHVPVENVV
EKVVEVPV
ERVV
EKVLQVPVEV
EKIIHVPVEHVV
EKVVEVPV
ERVV
EKVLQVPVEV
DKVA
EKVVEVPVEQAVI
RDVPV

EKTVEMIVEGPNMLDAAIGLAEEGKPFVFEAANGYGLTARHVEAGQQLKNKR
ADNDRWRLHRMSPGEFTVESLSAPGLRLGVGNQSGHGFKAVLVPEGDQRA
LLRLVPARDGSQQRVSFESVSQPGSLLNHCNGLMWFFDRPANQHFSNDSSW
VLLSSEKENSKTSRRGGTAQLRLEGKYLNLDESSGFTIRPRAGQTIVQPVTFT
SAQDVYPGHNPHTWTLKGTNDXGGMGITSGWGLSLEGSGFREYIRFENWKA
YRMYRVTPPPGPELHPRKVDADRAVRRPRPGRRQPPAKKKRERKREEGLSQ
GWX

Alignment of *Eutreptiella* articulins into two groups

Group 1 N-terminal half

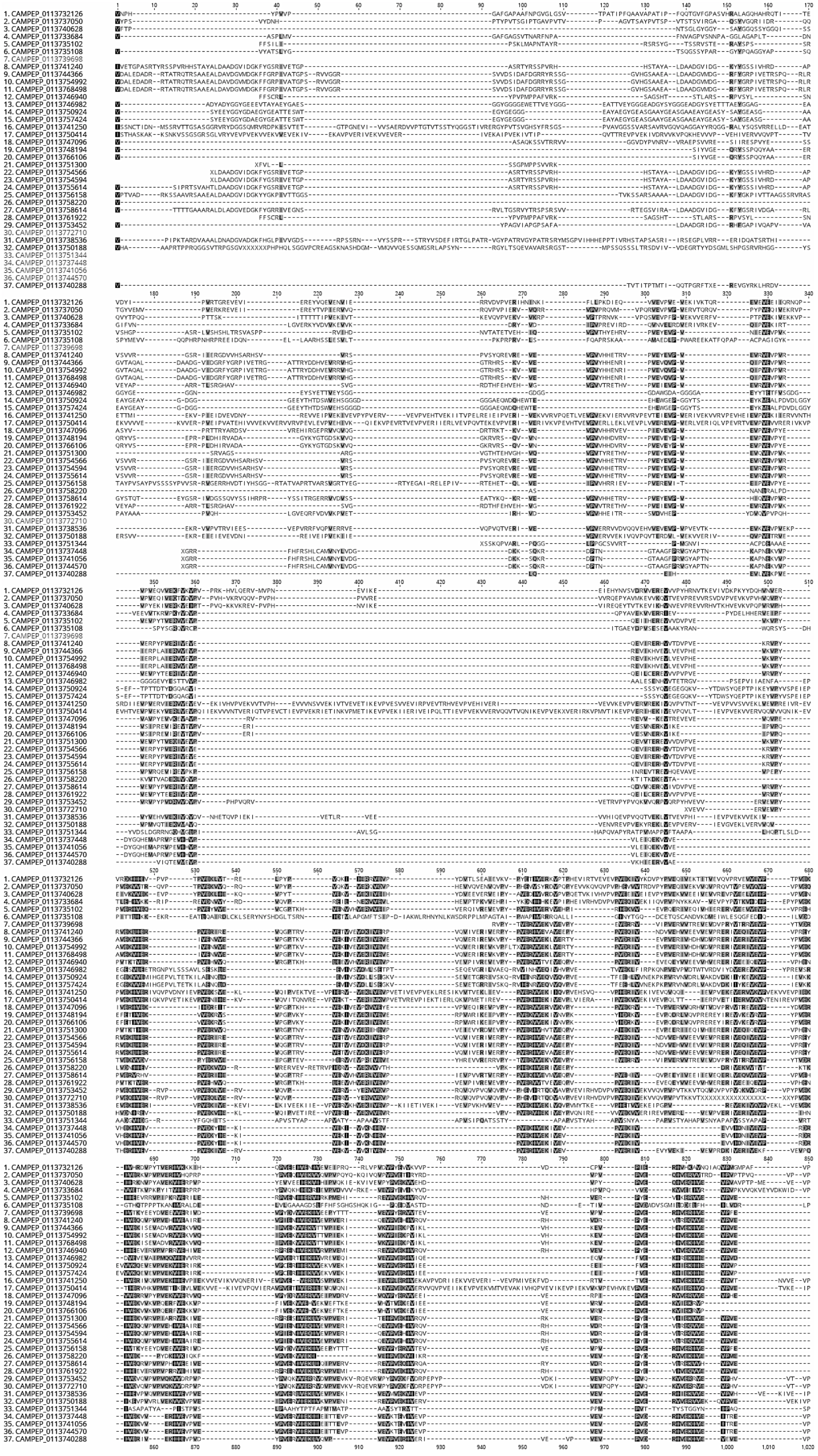

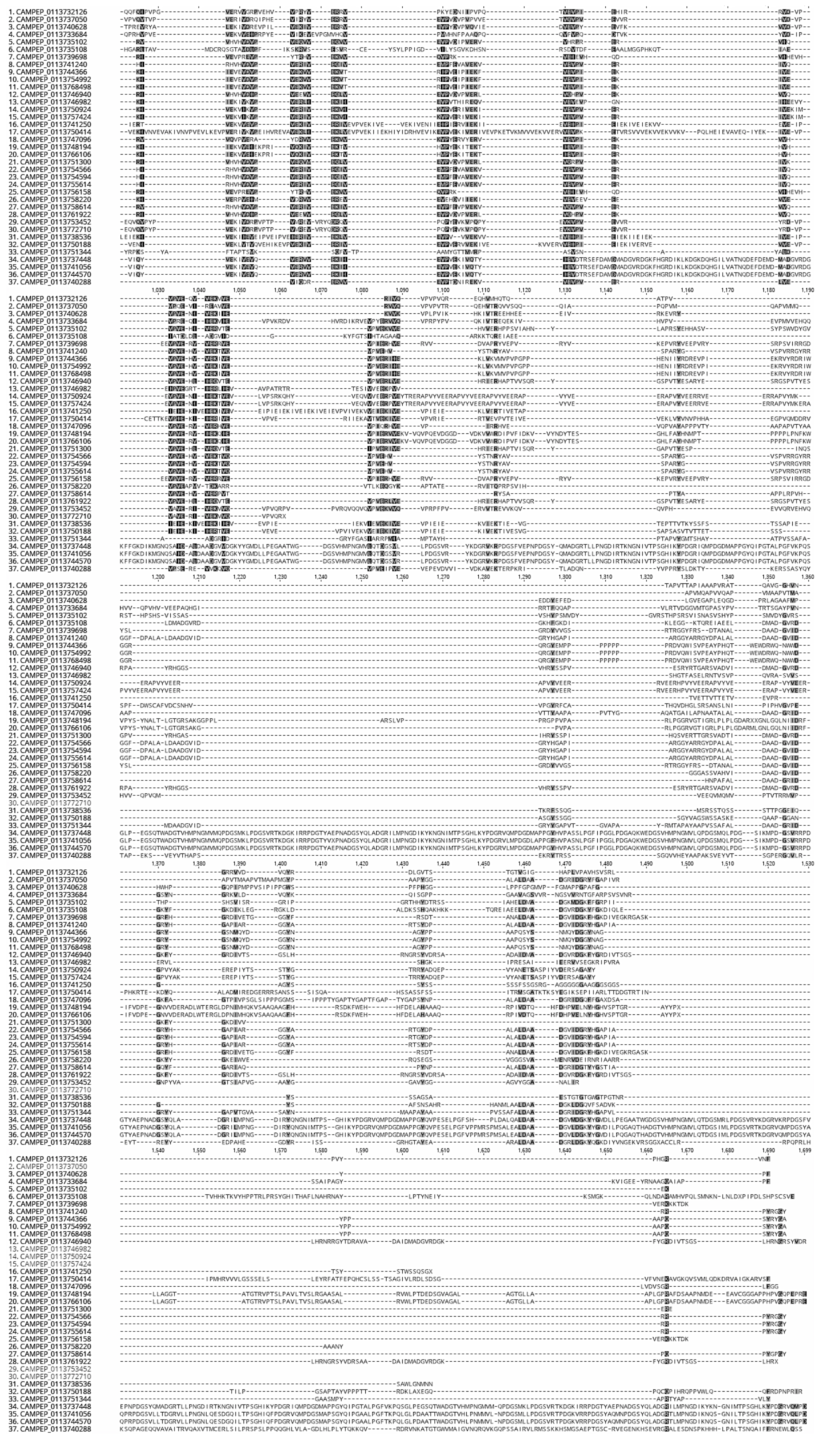

Alignment of *Eutreptiella* articulins into two groups

Group 1 C-terminal half

Alignment of *Eutreptiella* articulins into two groups

Group 2

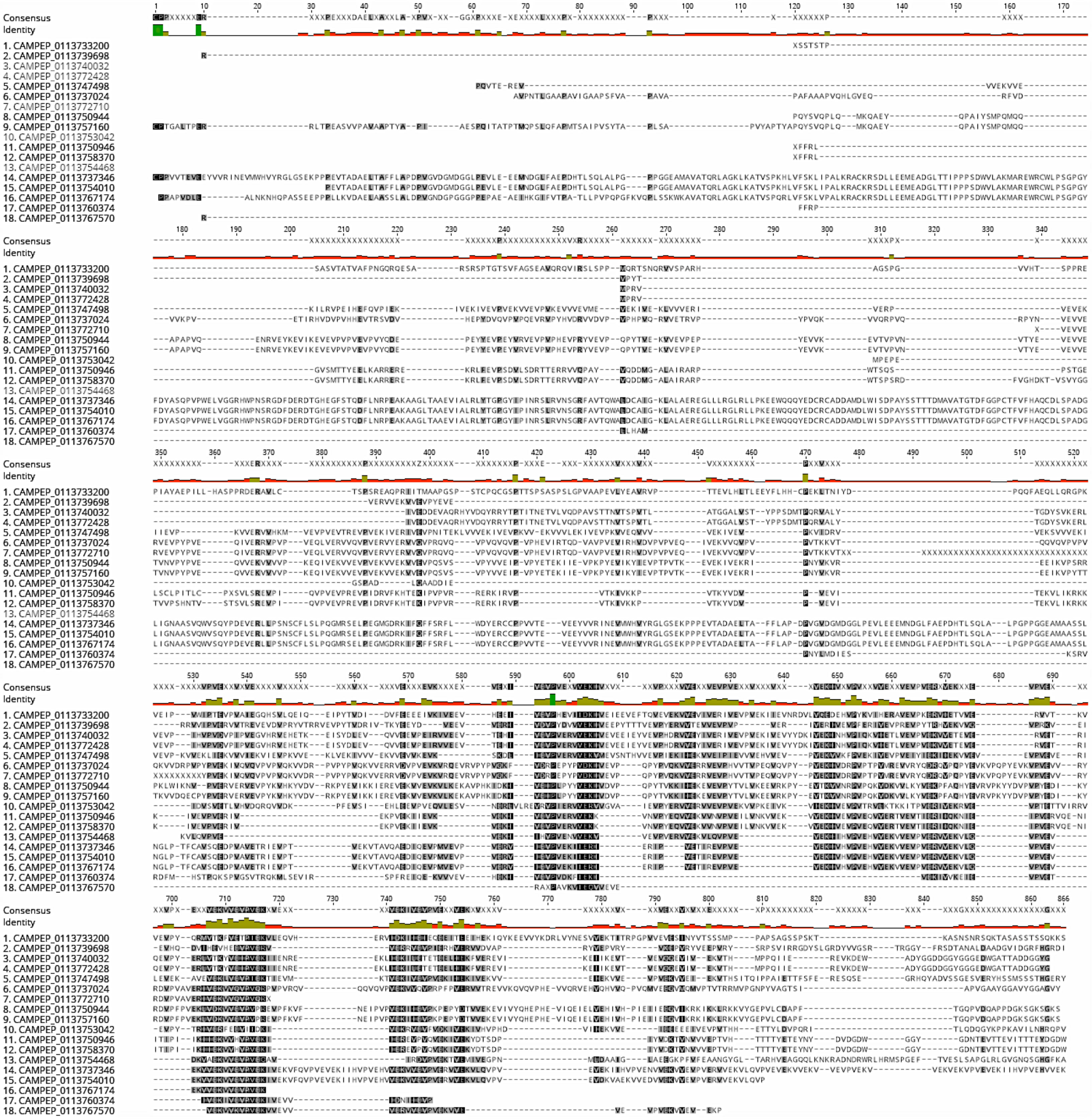

Supplement: TEXT S6 [file mbo005184120sf6.pdf]
