# Supplementary material for: Epiplasts: Membrane Skeletons and Epiplastin Proteins in Euglenids, Glaucophytes, Cryptophytes, Ciliates, Dinoflagellates, and Apicomplexans
Source: mBio. 2018 Oct 30;9(5):e02020-18. doi: 10.1128/mBio.02020-18 (PMC6212826; doi:10.1128/mBio.02020-18)
Supplement: TEXT S7 [file mbo005184120s7.pdf]

# Key to Gene Annotations

- **General organization:** The medial acid-base dyads (ABDs) are "parsed" into "strings that initiate with a dyad. N-term domain is denoted the head, C-term the tail.

- **Highlights**: Yellow, tyrosine; Green, cysteine; Pink, VPV; Gray, ABD in predicted head and tail domains.

- **Colored font:** Red, glycine residues in ABD domains; Bold-faced red, last G residue in head and first G residue in tail; Blue, alanine residues in ABD domains; Green, repeated string domains.

- **Underscores:** Acid-base triads or tetrads.

- **Commentary**: Notes on distinctive gene features (e.g. orthologues, localization patterns) in green font at top of some pages. Predicted homology domains (e.g. PDZ, coiled-coil) are given in Supplement Table 2.

- **Secondary structure**: PSIPRED predictions for a subset of proteins. Yellow, amino acids predicted in β-strand; pink, amino acids predicted in α-helix; no highlight, amino acids predicted in disordered (random coil) domain.

2

# *Cyanophora*_tig00020816_g14161_t1

Secondary structure prediction not commensurate with epiplastin designation but leaving in pool.

MRSAEKLVEVETVPARARRTMIVFRELVPCPVEVPAHTESVKV

REKIAVV
DRPVEQRIEVQVRVPF
REEVNII
REI
EKPVPFEVP
RDVRLPYEVVNRVVEQIVET
REVPV
EKVVFQLQNVVT
EKIV
EKPVPVEQW
ERSRY

ATLGFRSPVPPAQL

```
  1 M R S A E K L V E V E T V P A R A R R T M I V F R E L V P C P V E V P A H T E S V K V R E K I A V V  50
 51 D R P V E Q R I E V Q V R V P F R E E V N I I R E I E K P V P F E V P R D V R L P Y E V V N R V V E 100
101 Q I V E T R E V P V E K V V F Q L Q N V V T E K I V E K P V P V E Q W E R S R Y A T L G F R S P V P 150
151 P A Q L
```

3

# *Cyanophora*_tig00021108_g18294_t1

## Articulin. Secondary structure prediction next slide

MQRLKTLISEDAGAAPKPAAPIAQPVQPVSTGRMSPSFQAASAPGRLSPSFS
GATAGATYISGPLQSVGGTVRLRPRRRRASMSRSSALFPRLGLGGALQHASI
DARVIAASEQHAGPIGNVSETSSDVCITPRGPEARYA

EKLVEVETV
REVVRPVPVETVY
RELVPCPVEVPVHTESVVEVV
REKIVVV
DRPVEQRIEVQVPIEVPAPYEVRVPF
REEVNII
REI
EKPVPFEVP
RDVV
REVRVPYEVMNRVVEQIVET
REVPV
EKVVFQNVIT
EKIV
EKPVPVEIPV
EKLVVV
ERIVEVPV
EKIVEVKVPEIRTIEVPVPYQVPVEVV
REVRVPFEVV
KEVP
REFVTT
REVPV
EKIV
REVQTV
EKIVQVIKNVEIPV
ERIV
EKIV
ERPIIV
EKLVEVPV
DRII
EKEVPVIHEIQRPIVI
EKPVEVPI
DRVQ
DRVI
EKRVPVRRP

APRGSAAPTSTRGAPAQVQVDRVVDRPVPVPVDRPVPVPVAQPVAQPVVQQ
ASRAPRFPLASHPRAHPHARPPMTARARVRAKGDGMAAGSPPAMRMASPPS
AGGAGTPTRLGTPLTGQAAQTTTTSQRIAFSSGSTYTTMPQQQQGYVQGQQQ
QMGPYSASSAPSTAMGATTTAYSTGAPQQAGYAAGQQGYYTQGQGPYSASS
APSTAVGAAAGAGSPQKKGLLSKLGLKHGNK

4

# *Cyanophora*_tig00021108_g18294_t1

## Secondary structure prediction

```
  1 M Q R L K T L I S E D A G A A P K P A A P I A Q P V Q P V S T G R M S P S F Q A A S A P G R L S P S
 51 F S G A T A G A T Y I S G P L Q S V G G T V R L R P R R R R A S M S R S S A L F P R L G L G G A L Q
101 H A S I D A R V I A A S E Q H A G P I G N V S E T S S D V C I T P R G P E A R Y A E K L V E V E T V
151 R E V V R P V P V E T V Y R E L V P C P V E V P V H T E S V V E V V R E K I V V V D R P V E Q R I E
201 V Q V P I E V P A P Y E V R V P F R E E V N I I R E I E K P V P F E V P R D V V R E V R V P Y E V M
251 N R V V E Q I V E T R E V P V E K V V F Q N V I T E K I V E K P V P V E I P V E K L V V V E R I V E
301 V P V E K I V E V K V P E I R T I E V P V P Y Q V P V E V V R E V R V P F E V V K E V P R E F V T T
351 R E V P V E K I V R E V Q T V E K I V Q V I K N V E I P V E R I V E K I V E R P I I V E K L V E V P
401 V D R I I E K E V P V I H E I Q R P I V I E K P V E V P I D R V Q D R V I E K R V P V R R P A P R G
451 S A A P T S T R G A P A Q V Q V D R V V D R P V P V P V D R P V P V P V A Q P V A Q P V V Q Q A S R
501 A P R F P L A S H P R A H P H A R P P M T A R A R V R A K G D G M A A G S P P A M R M A S P P S A G
551 G A G T P T R L G T P L T G Q A A Q T T T T S Q R I A F S S G S T Y T T M P Q Q Q Q G Y V Q G Q Q Q
601 Q M G P Y S A S S A P S T A M G A T T T A Y S T G A P Q Q A G Y A A G Q Q G Y Y T Q G Q G P Y S A S
651 S A P S T A V G A A A G A G S P Q K K G L L S K L G L K H G N K
```

5

# *Cyanophora*_tig00000342_g24244_t1

MVLFVSGLVSSARPVQGTSFLSSAKLCVRREKVGTRRSFYVAASNNQEPRPVSSAKAFGASA
VAALSALIGAAPAAYADAIADMLEDAPTMPDMGVETMNFMMNSTPLEIGLGSAAVVATL
ALFSLLSRVGAREEQLAAALSREAELQARQAALEEELEVKQSAVAAEADKVAALKKEVESLN
QALRSERGELERAKSDTAMYRREVEAARVAVKEAEARVAEEQRAVEEIHTHLDHVRAELST
VSAEMLLEKKKAHEADAAAAAAQEALRQKAAELEHELLERGRAAAALEELATAQKNLAELK
AASEAARAELKQRDAAVNTLQAELTKARADVRVV

EKIV
EKPV
EKVV
ERRVEVPV
EKIV
EKIVT
KEVPV
EKIV
EKIV
EKIV
EKRV
EVPV
EKIV
EKIV
EKRV

EHRPRARPQGTSPAPAAKAAAPAPAKPAPAPVKAEAKPAPAKAEAKAAPAPAKPAPKAAPAK
AEAKAAPAKAEAPKASVEEVVAKAAAAVAKEAKKPAKAKAKRGFS

```
  1 M V L F V S G L V S S A R P V Q G T S F L S S A K L C V R R E K V G T R R S F Y V A A S N N Q E P R
 51 P V S S A K A F G A S A V A A L S A L I G A A P A A Y A D A I A D M L E D A P T M P D M G V E T M N
101 F M M N S T P L E I G L G S A A V V A T L A L F S L L S R V G A R E E Q L A A A L S R E A E L Q A R
151 Q A A L E E E L E V K Q S A V A A E A D K V A A L K K E V E S L N Q A L R S E R G E L E R A K S D T
201 A M Y R R E V E A A R V A V K E A E A R V A E E Q R A V E E I H T H L D H V R A E L S T V S A E M L
251 L E K K K A H E A D A A A A A A Q E A L R Q K A A E L E H E L L E R G R A A A A L E E L A T A Q K N
301 L A E L K A A S E A A R A E L K Q R D A A V N T L Q A E L T K A R A D V R V V E K I V E K P V E K V
351 V E R R V E V P V E K I V E K I V T K E V P V E K I V E K I V E K I V E K R V E V P V E K I V E K I
401 V E K R V E H R P R A R P Q G T S P A P A A K A A A P A P A K P A P A P V K A E A K P A P A K A E A
451 K A A P A P A K P A P K A A P A K A E A K A A P A K A E A P K A S V E E V V A K A A A A V A K E A K
501 K P A K A K A K R G F S
```

6

# *Cyanophora* Articulin tig00021719_g23170_t1

MQALRELFGGSAPKEAPPGEELAPEEEPEDEPEAEIAEQASDAAPRRTACT
TSSATKLQ

DRIVTVEVPRIVPVEVRTIE
ERLQCVEVEVPVPVHRVLEVPT
ERVVIQ
REIV
ERVV
DRPVVV
EKLVEVPV
EKIVVV
EKEV
EKII
EKPVYIEVP
KEVV
REVPVPLETI
REVPVTVEII
KEVPV
ERTVI
KEVIR
DRVVEVPVTVPVRV
EKAVTV
ERPV
ERTV
ERPVVV
ERPVQVPC
ERVVVV
ERPVV
REV
ERPVFI
ERRVEVPV
ERVVEVPVFVFPD

GSSRRAEVPAAAAAAAGAPEAPAAVPAAVPGSSEEPPPAGAPREEIPAPSAAAA
DAAAAPAPTAFEGGAVIGGFSTGVSADAGGAAGLSSSSELRTASQASTLPDGSL
AGEEGEEGSGPGGRGAPEAAAAGEDDEGGEEELEAGEAWGRAERGGRPEFS
DRATAAHESEHLGLQQPLEGLEADEAALSDETGELAEREADAERADERARAEG
RARASEADRDGPAAP

| | | | | | | | | | | | | | | | | | | | | | | | | | | | | | | | | | | | | | | | | | | | | | | | | | | |
|---|---|---|---|---|---|---|---|---|---|---|---|---|---|---|---|---|---|---|---|---|---|---|---|---|---|---|---|---|---|---|---|---|---|---|---|---|---|---|---|---|---|---|---|---|---|---|---|---|---|---|
| 1 | M | Q | A | L | R | E | L | F | G | G | S | A | P | K | E | A | P | P | G | E | E | L | A | P | E | E | E | P | E | D | E | P | E | A | E | I | A | E | Q | A | S | D | A | A | P | R | R | T | A | C |
| 51 | T | T | S | S | A | T | K | L | Q | D | R | I | V | T | V | E | V | P | R | I | V | P | V | E | V | R | T | I | E | E | R | L | Q | C | V | E | V | E | V | P | V | P | V | H | R | V | L | E | V | P |
| 101 | T | E | R | V | V | I | Q | R | E | I | V | E | R | V | V | D | R | P | V | V | V | E | K | L | V | E | V | P | V | E | K | I | V | V | V | E | K | E | V | E | K | I | I | E | K | P | V | Y | I | E |
| 151 | V | P | K | E | V | V | R | E | V | P | V | P | L | E | T | I | R | E | V | P | V | T | V | E | I | I | K | E | V | P | V | E | R | T | V | I | K | E | V | I | R | D | R | V | V | E | V | P | V | T |
| 201 | V | P | V | R | V | E | K | A | V | T | V | E | R | P | V | E | R | T | V | E | R | P | V | V | V | E | R | P | V | Q | V | P | C | E | R | V | V | V | V | E | R | P | V | V | R | E | V | E | R | P |
| 251 | V | F | I | E | R | R | V | E | V | P | V | E | R | V | V | E | V | P | V | F | V | F | P | D | G | S | S | R | R | A | E | V | P | A | A | A | A | A | A | G | A | P | E | A | P | A | A | V | P | A |
| 301 | A | V | P | G | S | S | E | E | P | P | P | A | G | A | P | R | E | E | I | P | A | P | S | A | A | A | A | D | A | A | A | A | P | A | P | T | A | F | E | G | G | A | V | I | G | G | F | S | T | G |
| 351 | V | S | A | D | G | G | A | A | G | L | S | S | S | S | E | L | R | T | A | S | Q | A | S | T | L | P | D | G | S | L | A | G | E | E | G | E | E | G | S | G | P | G | G | R | G | A | P | E | A | A |
| 401 | A | A | G | E | D | D | E | G | G | E | E | E | L | E | A | G | E | A | W | G | R | A | E | R | G | G | R | P | E | F | S | D | R | A | T | A | A | H | E | S | E | H | L | G | L | Q | Q | P | L | E |
| 451 | G | L | E | A | D | E | A | A | L | S | D | E | T | G | E | L | A | E | R | E | A | D | A | E | R | A | D | E | R | A | R | A | E | G | R | A | R | A | S | E | A | D | R | D | G | P | A | A | P | |

7

# *Cyanophora* tig00021493_g21879_t1

MEPQDSSNPYVSVLQKRQRALRKKLEKLTSLSEKQAKGGSLNADQVEALNS
KSKTDSLLEEVTAIIALLEPIA

KEEKK
KEVVV
EKPAPVV
EKPA
EKP
KEVPPPPPPV

APPKPESLVPEQLARLLRVLFAASVFDLASPSGRDARAFLLAENEAKRAQGQK
VVIQSDAEFEALDQFSKLLRGYAREGEDLSSAIQKAVIHATKLIEKNDGNAAGS
VSYKALLSYVDEIFGSTFISTHVPAAPAPVPIAAAHDAAVGGGEYAGAGEGDG
SGQYMQYDDGQYMQADPAYQGEMYHEESYGVPYGNGHYDYGAEEYGATIN
FLQESTLEAHEYMGTEAGAAEAAAQGHPHHVHGHPSLFHGLHGPPPPVPPV
PPPAGHAGPHGLPAPHPHGVPHGHGHGHGHPLAHPGHHPGAPVPPPPRAR
TPARRTPAPSSPRASPFPARRHPRAPRRCPPGPHRPAGPRGPARPGPPPSR
PRPRPVQAPPAASPAPPAAPAATTVHVVAHVATAPSGSPSASPTGAAIPLTTL
EGAAVSAPTNSPPPVSGDMRGGRGGGERGAAGAAGAAGAGAAAWTARAAT
ARRGGAAGGGRGGRGGRGRGGAPSGAPYYEPRGRGGRGGRGGYGYRYD
DSRPEISARDE

8

*Cyanoptyche* MMETSP1086_c2694_g1_i1_g5328

## Articulin

…FSRTEEVTVTRQAPQQ

ERVV
EKIVEVPV
ERVV
ERIVEVPV
EKIV
EKRVEVPV
ERIV
EKRVEVPV
ERIVVQRVEVPV
EKIIHVPV
DRIV
EKFV
DRVI
EKRVPVQVPV
EKIV
EKPV
ERLV
EKIVRVPQIV
EKIV
EKPV
DRIV
EKRVPYPV
EKIVE

1 F S R T E E V T V T R Q A P Q Q E R V V E K I V E V P V E R V V E R I V E V P V E K I V E K R V E V
51 P V E R I V E K R V E V P V E R I V V Q V E V P V E K I I H V P V D R I V E K F V D R V I E K R V P
101 V Q V P V E K I V E K P V E R L V E K I V R V P Q I V E K I V E K P V D R I V E K R V P Y P V E K I
151 V E

9

*Cyanoptyche* MMETSP1086_c18412_g1_i1_g20445

Articulin

…ACVAPGPTTTVV

EKAVPLRQIVEVPVEVPV
ERVI
EKPVPCEVQIPVPYEV
EKIV
ERPVEVV
KEVPV
ERIVEVPV
ERRVEVPV
EKIVQVPVEVPV
ERIV
EKIVEVPV
EKIVNVPV
DRPYPVEV
ERPVPVNIPV
ERVQVELLVPKVPVEV

ASSAAAYSTAPAISSVDTMTPGMAALHTGPVIPTTAAPIAEEHHKRGIGHKMKE
MLGLAHHHPAEGTTTHAHAAPAPVAGAHTTTTTTVHEQQSRSIP

1 A C V A P G P T T T V V E K A V P L R Q I V E V P V E V P V E R V I E K P V P C E V Q I P V P Y E V
51 E K I V E R P V E V V K E V P V E R I V E V P V E R R V E V P V E K I V Q V P V E V P V E R I V E K
101 I V E V P V E K I V N V P V D R P Y P V E V E R P V P V N I P V E R V Q V E L L V P K V P V E V A S
151 S A A A Y S T A P A I S S V D T M T P G M A A L H T G P V I P T T A A P I A E E H H K R G I G H K M
201 K E M L G L A H H H P A E G T T T H A H A A P A P V A G A H T T T T T T V H E Q Q S R S I P

10

# *Gloeochaete* MMETSP1089_c5913_g1_i2_g11946

## Articulin

…EEVPVVSEEVPVLS
KEVPVVC
KEVPVVSEEVPV
DREKVPVVS
EKVSVVEPVVEAPIVSEEVPVLSEEVPVVS
KEVPVVSEEVPVVS
KEVPVVSEEVSVVEPVVEAPIV
REEGPIVQ

SVVEAPIVSEEASVVEPVVEAPVLSEDVPVVEPVIDEAGVVGSEETPVEGVS
TEAGVVPVVDNAEPAETPVVSLEDASPLAAVANVPDVPSAVEPILPLPPVSE
SSAPLEPAPVIEDTEGEVAELSLPEPEQDVDTAAAADPQEDTALEQRVHTPL
SLPSSINETTVEFGEELAPLDLDLDSLPLHVMEPELLESPKDDTSYEDEDEVT
SPQQLGDEDIQQGYIRPLAGDNEVQPAPDDADSPDDESPADDESPADDVAV
RG

11

# *Gloeochaete* MMETSP0308_c12336_g1_i1_g19682

Articulin. Secondary structure prediction next slide

...PKVIWK

EKLVPQ
ERVV
ERMVPVPVETVY
EKAVPCPV
ERPVFN
DRIVTVEV
DRPVEVIR
KEVIEV
ERPVYIEVP
KEVV
REVQVPIETV
REVP
KEIIIT
KEVQVVV
EKFV
EKIV
EKIVEVPRIVEVPC
DRIV
EKIVYEI
KEIT
KEVPII
KEVI
KEV
EKRVEVPIYV
ERVVEVPV
ERPVEV
ERF
REQKV
ERPVYINVPV
EKTITV
ERPIETV
REVP
KEVVIR
RDVPIIQ
DRI
KEVI
REKVVEVPRVVEVPH
EKMVVV
ERPV
ERLMTVHVPVETI
KEVTV
ERRIQVPFAQPVETSVV
KEVPYSV
DRFVEVP
KDRLVEVRHEV

AVPMPTQSEQSTMTATSTQQRYSSALAQSTEREFRQGEEYRESGDYIRSSNV
SEEIALFAEAGMEMAAAAASQTGKGATVLITEEIVITPERQGEE

# *Gloeochaete* MMETSP0308_c12336_g1_i1_g19682

12

Secondary structure

1 P K V I W K E K L V P Q E R V V E R M V P V P V E T V Y E K A V P C P V E R P V F N D R I V T V E V
51 D R P V E V I R K E V I E V E R P V Y I E V P K E V V R E V Q V P I E T V R E V P K E I I I T K E V
101 Q V V V E K F V E K I V E K I V E V P R I V E V P C D R I V E K I V Y E I K E I T K E V P I I K E V
151 I K E V E K R V E V P I Y V E R V V E V P V E R P V E V E R F R E Q K V E R P V Y I N V P V E K T I
201 T V E R P I E T V R E V P K E V V I R R D V P I I Q D R I K E V I R E K V V E V P R V V E V P H E K
251 M V V V E R P V E R L M T V H V P V E T I K E V T V E R R I Q V P F A Q P V E T S V V K E V P Y S V
301 D R F V E V P K D R L V E V R H E V A V P M P T Q S E Q S T M T A T S T Q Q R Y S S A L A Q S T E R
351 E F R Q G E E Y R E S G D Y I R S S N V S E E I A L F A E A G M E M A A A A A S Q T G K G A T V L I
401 T E E I V I T P E R Q G E E
