# Supplementary material for: Epiplasts: Membrane Skeletons and Epiplastin Proteins in Euglenids, Glaucophytes, Cryptophytes, Ciliates, Dinoflagellates, and Apicomplexans
Source: mBio. 2018 Oct 30;9(5):e02020-18. doi: 10.1128/mBio.02020-18 (PMC6212826; doi:10.1128/mBio.02020-18)
Supplement: TEXT S8 [file mbo005184120s8.pdf]

Guide to this Supplement

Figure 8.2 lists non-epiplastin proteins proteins that localize to cortical domains in eukaryotic organisms.

Figures 8.3-8.17 show the amino-acid sequences and predicted secondary structures of a subset of these non-epiplastin proteins (green font in Fig 8.2) that localize to cell-surface domains in eukaryotic microorganisms.

Figure 8.18-8.23 show the amino-acid sequences and predicted secondary structures of non-epiplastin proteins that are known to be dominated by β-strand domains.

2

These proteins have been identified in various publications as associated with the cell surface of organisms that assemble epiplasts, or as being homologous to such proteins, but their sequences and/or predicted secondary structures indicate that they are not members of the epiplastin family.

Green font: sequences and predicted secondary structure are found on subsequent slides (in parentheses)

Cowpox virus A-type inclusion protein BAA00222.1
Euplotes aediculatus platein precursor α-1 AAM94462.1
Euplotes aediculatus platein precursor α-2 AAM94463.1
Euplotes aediculatus platein precursor βγ AAM94464.1
Plasmodium membrane skeleton protein XP_001351115.2
Symbiodinium MMETSP1367_c7881_g1_i1_g17673 (8.3)
Tetrahymena pyriformis Epiplasmin C AAF85984.1
Tetrahymena thermophila TTHERM_00688340 EAS06694.1 (8.13)
Tetrahymena thermophila EPC1 EAR95236.2
Tetrahymena thermophila XP_001016574.1 (8.11)
Tetrahymena thermophila Tetrin A EAR87868.2 (8.14)
Tetrahymena thermophila Tetrin C EAR86044.1
Tetrahymena thermophila Tetrin D EAS03720.1
Tetrahymena thermophila TtDBF1 EAR96064.1
Tetrahymena thermophila TTHERM_00006110 EAR87847.2 (8.12)
Tetrahymena thermophila TTHERM_00578520 XP_001022853.2
Tetrahymena thermophila TTHERM_00945250 XP_001026632.2
Tetrahymena thermophila TTHERM_00578520 XP_001022853.2
Toxoplasma gondii IMC2A AAK38356.3
Toxoplasma gondii IMC2A XP_002366439.1 (8.15)
Toxoplasma gondii IMC18 EPR57770.1 (8.17)
Toxoplasma gondii IMC19 EPR63851.1
Toxoplasma gondii IMC20 EPR64642.1
Toxoplasma gondii ISP1 XP_002365245.1
Toxoplasma gondii ISP2 XP_002372015.1
Toxoplasma gondii ISP4 XP_018637272.1
Toxoplasma gondii RNG2 XP_002366936.1
Trichomonas vaginalis XP_001582404.1 (8.4)
Trichomonas vaginalis EAY04230.1 (8.7)
Trichomonas vaginalis EAX98628.1 (8.8)
Trichomonas vaginalis EAY12684.1

3 *Symbiodinium* MMETSP1367_c7881_g1_i1_g17673

MEAEGHAFEAAEKVKEIQCEVEDLLLHIRRAADIESQHLEAQRVELQKQHA

ERI
KDLQDEVRRV
RESTDESV
EKIEAEMQ
REQSQIEEEVLQKSV
ERSEALKAI
KEEVEDLSAVADQEYRKSL
DKDKLRHEEARKAILDMDNQRWRTVEEMDQSCQMWITAFNQRLEETQAD
LAYRVEHLVGEARAARLGLHKA
RERL
DRQWEDELTQLRQEAL
KERFDAASTLDQARLKR
RELESMS
REKSLAGIEECKLLK
REHLLTLRRVADELDQMRRNI

GLQARDPMLVQSLRELSSKIRHGQMKQQPPKMMEIT

1 M E A E G H A F E A A E K V K E I Q C E V E D L L L H I R R A A D I E S Q H L E A Q R V E L Q K Q H 5
51 A E R I K D L Q D E V R R V R E S T D E S V E K I E A E M Q R E Q S Q I E E E V L Q K S V E R S E A 1
101 L K A I K E E V E D L S A V A D Q E Y R K S L D K D K L R H E E A R K A I L D M D N Q R W R T V E E 1
151 M D Q S C Q M W I T A F N Q R L E E T Q A D L A Y R V E H L V G E A R A A R L G L H K A R E R L D R 2
201 Q W E D E L T Q L R Q E A L K E R F D A A S T L D Q A R L K R R E L E S M S R E K S L A G I E E C K 2
251 L L K R E H L L T L R R V A D E L D Q M R R N I G L Q A R D P M L V Q S L R E L S S K I R H G Q M K 3
301 Q Q P P K M M E I T

4

Trichomonas vaginalis XP_001582404

First half of sequence. Repeats in green and red. Predicted Secondary structure in slide 8.6.

MTTPHRTPKRWEISPEGTPVTPSPGGNISPFVEVGEYNQNVQDLQEENFSLKMKINAL
QQQLATVQEQYRTVTTRIEETTYQYQSNDSSQDQNSTDPKTKGGSSSDF**G**SVAEWM

EKL
ERAT
KENDDLK
REKETYQELAVTLQNQLDTQVSNFEFQI
EKLQ
REKDDLSRLNVQLQ
EKIKLLSTSYTDLSAKF
DKQNEDHEIEIAKLI
DRQN
ERL
KEIEESHKSNIQQIQTN
KDGLTNQIQQEFTKT
KEDL
DKSR
KEYKQLEELQRKAQEENTKTISLLNIQINQLQNQL
EKAYSGKQADDVAVKKNIADL
ERSNA
EKDVVIQSLSTKVGRFE
EKVSNLEAKISEY
EKTIKQLNNS
KEDLQKQINNFSNKIDI
ERA
EKQIYIENNNDL
KEQIQNDEIKFQ
KERKEFQQELENLRIKFVQLSNNT
EKDNLIQHLQEEINALRQKLSEYSKIVENSKSTPG
KESYESTITNLRTQINMLKLENQEIK
KDIENYDTM
EKQNEEMKKQMDDLRSQL
KEN
KDYQSQLENMKLIQEENDDL
KERIGDMSNLSDQILELKKKLNDSEN
EKEILRKQIDNLC
KDDEEEDVPTFSKVISDLKVENQILKKKISDSEQIS
KENEDLKKQINEYIDIENENDEL
KDEISTLQNNIQKIT
ERNEEI
EKQNDDLKKNNDDLHVKIHNLEQKVDNLTNLNNELTINQMKYEDI
KEENDLLKNKSAS
PVSATPRTQQNKIQQLQMRNDELKTEIEILHQTI
DKLTSARI
KDNDSKTVDNEIDLLK
KENERLNAMLDDSSMQIIMLQQEIDENKSNSLKQEN
EKLQEQIEELQKHSPSPKKLQQENNSLKQEN
EKLQEEIEELQNTV
DKLQNENNLQSLQEEN
DKLQDEIEELQSTV
EKLQQENEELKNNKPIYSPSPKKLQNENNSLKQEN
EKLQEQIEELQNTI
DKLQNSNKSPNKLQQENNSLKQEIENL
KEEIEQNNKSKSYSPNKLQNENESLKQEN

5

Trichomonas vaginalis XP_001582404

Second half of sequence

EKLQEQIEELQNTV
EKLQQENDLLKNNKSVSPSPKKLQQENDLLKNNKSVSPSPKKLQNENNSLKQEN
EKLQEEIEELQNTI
DKLQNSNKSPKKLQQENKSMLNSPNKLQNEYETLQEEN
EKLQDEIEELQSTV
EKLQQENDLLKNSKSKSVSPSPKRLQQENNSLKQEN
EKLQEEINQLQNTI
EKLQNNKSKLYSPSPKKLQNENESLKQEN
EKLQEQI
EKLQQENDSKPKYSPSPRKLQQENNSLKQEN
EKLQEEIDQLQNTI
EKLQQENNKSKSLLNTPNKLQNEYETLQEEN
DKLQDEIEELQSTV
EKLQQENEELKNNKPIYSPSPKKLQNENNSLKQEN
EKLQEEIEELQNTI
DKLQNSNKSPNKLQQENNSLKQEIENL
KEEIEQNNKSKSYSPNKLQNENESLKQEN
EKLQEEIEELQNTV
EKLQQENDLLKNNKSVSPSPKKLQNENNSLKQEN
EKLQEEIEELQNTI
DKLQNSNKSPKKLQQENKSMLNSPNKLQNEYETLQEEN
EKLQDEIEELQSTV
EKLQQENDLLKNSKSKSVSPSPKRLQQENNSLKQEN
EKLQEEINQLQNTI
EKLQNNKSKLYSPSPKKLQNENESLKQEN
EKLQEQI
EKLQQENDSKPKYSPSPRKLQQENNSLKQEN
EKLQEEIDQLQNTI
EKLQQENNKSKSLLNTPNKLQNEYETLQEEN
DKLQ
DKIEELQSTI
EKLQQENEELKNNKPIYSPSPKKLQNENNSLKQEN
EKLQEEIEELQNTI
DKLQIENKSPNKLQQENNSLKQEIENL
KEEIEQNNKSKSYSPKKLQQENNSLKQEN
EKLQEEIDELQNTV
DKLQNENNLQSLQEEN
DKLQDEIEELQSTV
EKLQQENEELKNNKPIYSPSPKKLQNENNSLKQEN
EKLQEEIEELQNTI
DKLQIENKSPNKLQQENNSLKQEIENL
KEEIEQNNKSKSYSPKKLQQENNSLKQEN
EKLQEEIDELQNTV
DKLQNENNLQSLQEEN
DKLQDEIEELQSTV
EKLQQENEELKNNKPIYSPSPKKLQNENNSLKQEN
EKLQEEIEELQNTI
DKLQIENKSPNKLQQENNSLKQEIENL
KEEIEQNNKSKSYSPNKL

6

Trichomonas vaginalis XP_001582404

Predicted secondary structure

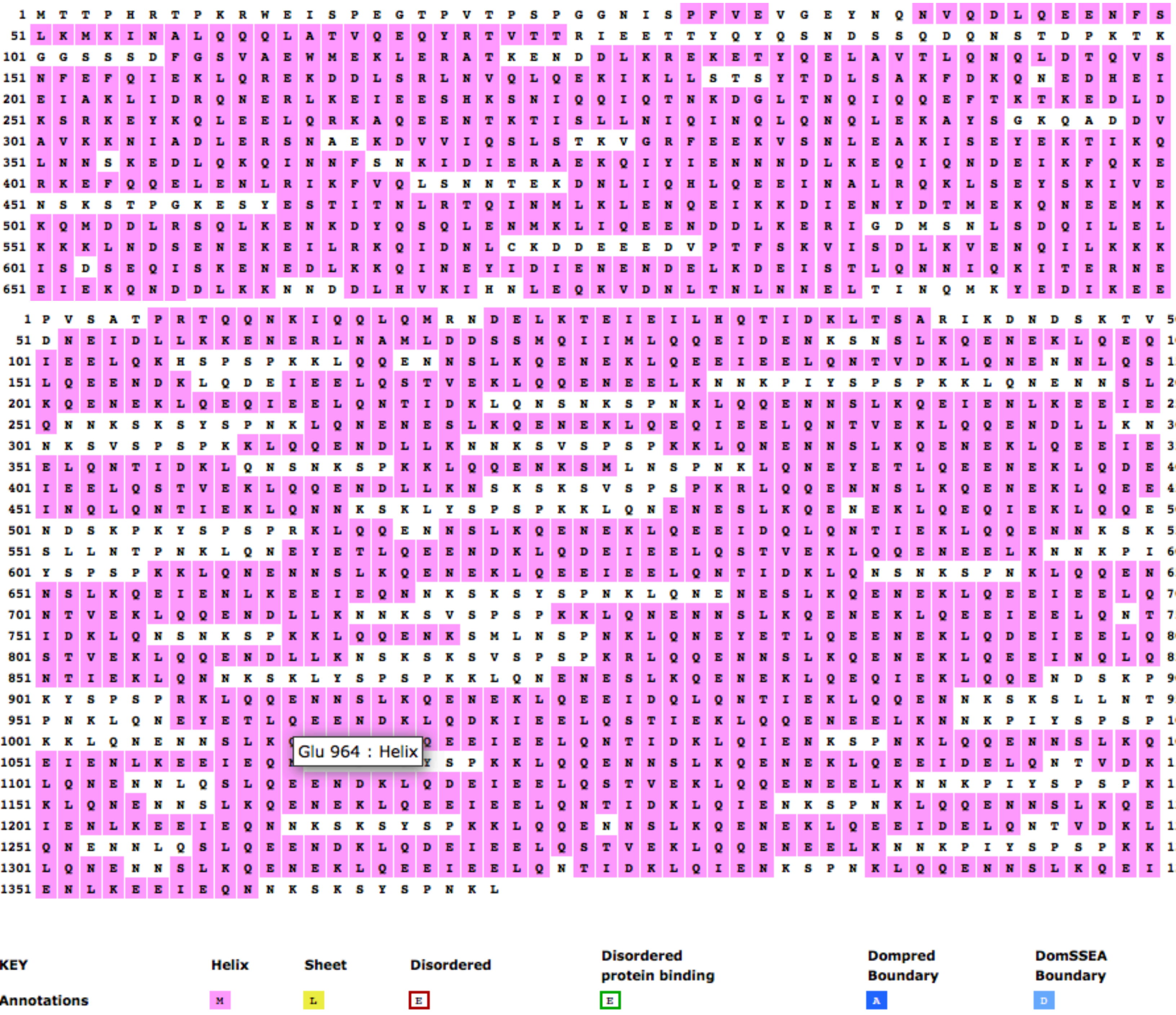

Trichomonas vaginalis EAY04230.1

Predicted secondary structure next slide

MSDQEIAATPNEEMTEEQLEWQAK

EKEYQDQIEDLKS
EKLELEAQVKQVDSFIQKLQETRNENSELKAKVTDLTRTIDDYDHRLQLLNE
EKSRLESEIKSTVQASAQLHSEDFVAMSENYDASKKQYEQQIQKLKAQIAEQ
QEMLNK
RDTEQKLVASQN
DRLIQSARRFF
KENISSLDQII
EKFDEPQIIEQPPVAPKSPTKSPTKTMPIPQTPQVDE
EKYNNLKKRYQQAKQTIAQLQEQIEQNNQQAKQQNNANQKTIKNLQNKLDEA
QENARH
DRDVLSRQINDLNGKLSDLKSQLKTPAPTKLEDDDDDITVVK
RDITLPTSMMDSAVKSPMKGIAPQPSNNDLQQVM
DKHAAEMNALK
KELK
DRMAQS
DKAINEASKAAEDLRAKLIQTENQ
RDDANSQNKILQSKLDSLQSLNDGLNQQIEGLRQALHAKQPEQKPQSNSQAKPA
APQQPSNEM
KEQVRKLKAELEELNNKLMAQQIALNTAENTIH
DKDKTLSEQQKKLDEAEAKQQQLSMDL
REAQNKIANTPKPDPNQWLP
RDAWHYAGFATPLSTAI
DKIAANQSLLPASKLTHVFAEIDDFFTEEINKA
DKNASQANQNFENTKAKITTFVVDSSIALGIEPKTFEQFQTGQAPSEIVKAIKQ
LKQEFAELQALSRNQQETLMHFAESFELQEGQDVDEHIDAIRSGIDT
QNQKYAATY
EKARRYKQAYADLAAQSKKQIQELVE
EKERLQDENDDLTQKVESTQRSLMQA
REETEQYATQLNNSVQ
ERDLGDINHVNEME
ERTNALAGKYETQVADLVGQINNVKQENAKLSNDLVNKKAESQELQQSLEEAQY
ENEQLA
KELDQTVEDFKNKLNQAQQQ
KETESAAAK
KDFDDVIAKLQKQIEGLTQDVNRLVGELKDSETANKQLKGQVSKLQQ
EKQRAAE
EKKSLLARL
EREVMLADAKARARIIE
EKALMD
EKLHTAEAKFEQTKMQLINQFV
DKFTKFFNPAQAVT
EREYRAGV
EKAHDEFQRITESDESIRKMLRA
KDGQTTEDAVAQILVAKN

8

Trichomonas vaginalis EAY04230.1

Predicted secondary structure

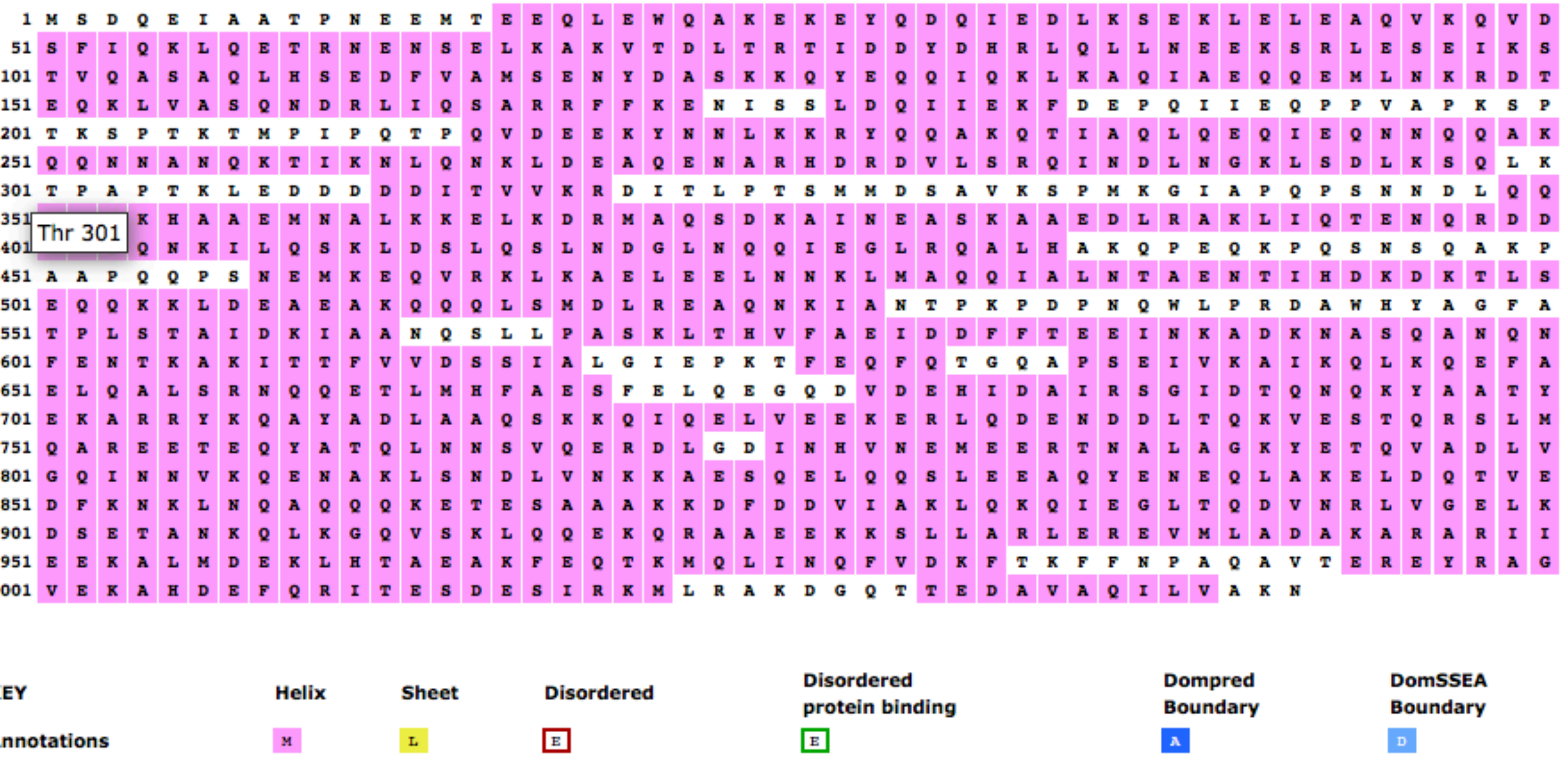

9

Trichomonas vaginalis EAX98628.1

Predicted secondary structure next slide

MSDQESFGDLSFPTSPNKSGATADSEASAKQQLQKVYKILSTSFSREVQSXXX
XXXXXXXXXXXXXXXXXXXXXXXXXXXXXXYKKRVLALEDQLREQQTANEEAQ
TQIRELKQAQDDLK

REKTKLTEDLTKAQLAAIQNQPVVQETDPV
EKTAKLFEELVAAQASEIEDLVHQ
RDILIENIRSLDGAVLDA
EKMLTEYAN
EKAES
EKMKSELIKKNEALT
DKLPTLAQEVEQRIPCDLRSSLPQNGSDS
EKFILATIEALVQTRAVPQQPVVE
EKEETVAKNKYIALLTRLEDAVRFIQSIAKAG
DKAGDICPLGIDSEVRTQLLTQAARMGHFVDENLLAVGIENLPNHMSIFEPGSF
ESTEAQL
KEFYNFVTEEQL
KESPV
RELFALFASVCEVNKMVMNFAENQRNKSHQT
DRNVVNSEALRNLQ
RDNFDLAQWKAVNQ
EKIEAATAVLAKLTEDDGETAMDVMANKLAQKYEDAA
KEN
KEL
KETI
EKLEN
EKKEMEESNQVSMQEATEQIQT
RDISLQEA
EKSRLEETQQLN
EKVSQLSQQNQSLAQRFKATTEGLEAALR
EKTAKLKKALLAQTETKKMLDQIQ
ERTNAIVSENETLHQQNAEMEETLTIQ
KEQLDALAEN
EKKL
RDS
REALKKRIANYEQQNSTTLQDLKTRNDAVQQKYQDTITNL
EKEIQDLKAATEQL
KEENESGKK
ERQELQQTITNLRVS
ERSLNLKLKTLQ
ERQAL
EKSAAEARNNTYMISLKAQAGRQIDEAKAQLEQTRQAITKILQNQFGEQLEEDASFND
LFNRL
DRRLQMYTS
DKRILADAIKLRGDLKLAANVTLSEAWASEVQSKNNAETAVARITK
ERRIIENELTNLKRST
EKMSQDAKQAAEWENWGRSLFKQVNESSAEIPVQELRFKLSEDVLAAIK
DRSTLKRLDTLRQQKKVLKNPALQEPIKTNQELSIRSIMCILIFAQRINGGPGNLTPIS
ISPRKD

10

Trichomonas vaginalis EAX98628.1

Predicted secondary structure

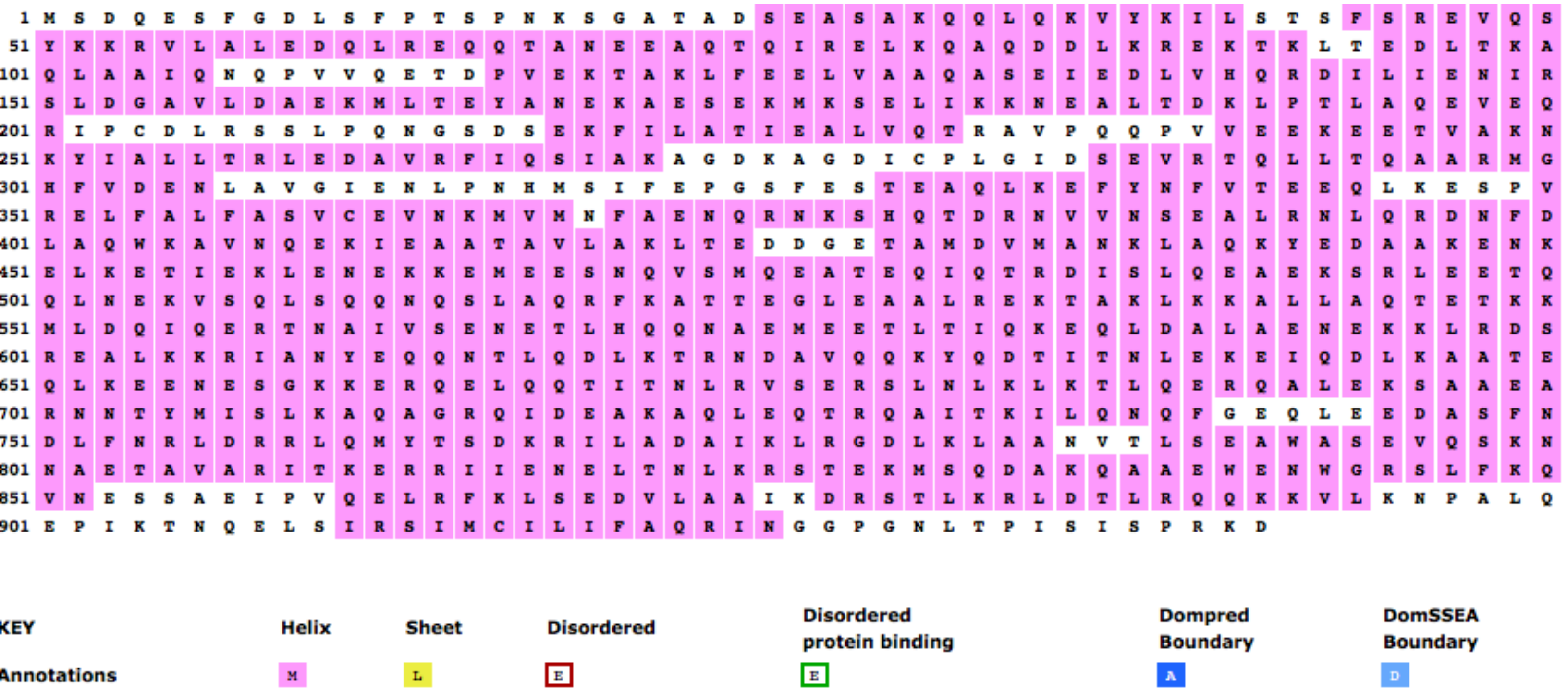

11

Tetrahymena XP_001016574.1

MSSYSQNRFIPNSPHN

DRISKIQ
EKLNSIQIGVES
ERYRKFEQAESHLCVLEEQFFEVQGDF
DKTLNSI
RENLIRF
EKVLQE
ERINHDSQIDQGNQEIAQIENKFVQAIESETEAR
KDYEAKVIRNLE
DKYSFLKNEIS
KEQLSRNQFIDELYQTLQSDLPKIEGAIEN
EKQ
EREENDQAMMKTVTYELSQINEIVITQKKQ
REQSEAAIFDML
KDVVNRVKIELDN
EKKT
REETEDHLLTLLEETCSKLQTASQF

1	M	S	S	Y	S	Q	N	R	F	I	P	N	S	P	H	N	D	R	I	S	K	I	Q	E	K	L	N	S	I	Q	I	G	V	E	S	E	R	Y	R	K	F	E	Q	A	E	S	H	L	C	V
51	L	E	E	Q	F	F	E	V	Q	G	D	F	D	K	T	L	N	S	I	R	E	N	L	I	R	F	E	K	V	L	Q	E	E	R	I	N	H	D	S	Q	I	D	Q	G	N	Q	E	I	A	Q
101	I	E	N	K	F	V	Q	A	I	E	S	E	T	E	A	R	K	D	Y	E	A	K	V	I	R	N	L	E	D	K	Y	S	F	L	K	N	E	I	S	K	E	Q	L	S	R	N	Q	F	I	D
151	E	L	Y	Q	T	L	Q	S	D	L	P	K	I	E	G	A	I	E	N	E	K	Q	E	R	E	E	N	D	Q	A	M	M	K	T	V	T	Y	E	L	S	Q	I	N	E	I	V	I	T	Q	K
201	K	Q	R	E	Q	S	E	A	A	I	F	D	M	L	K	D	V	V	N	R	V	K	I	E	L	D	N	E	K	K	T	R	E	E	T	E	D	H	L	L	T	L	L	E	E	T	C	S	K	L
251	Q	T	A	S	Q	F																																												

KEY	Helix	Sheet	Disordered	Disordered protein binding	Dompred Boundary	DomSSEA Boundary
Annotations	M	L	E	E	A	D

12 *Tetrahymena* TTHERM_00006110 EAR87847.2

MSNNPNHQENILVNQDYMQDNQEIGPIEINDEYYHDQNEQQQQDIDENDHHDEEYDEQGE
HEQGDDDNNEDQAYNLQNSARQQMEGINQAVINYQGKMQEIQNQIQHEQPQKANFKQYQI
QESHIREQTEEDHYDEQIINDGHKKQYDELDNDEQYQQQYKQMSQEKNQEYKQNIQQKRY
NQPQQFIEYKTSPRNAGLQQYQESESNYQLDESSDQNQNRPQKHYFDGQQKNIKSNQKNS
DKAKHIKNASESLTSQLDNVSNIMDDHSKSLVRQIKQDESQLETSQANVSVNQNNPYDEH
AEYQKKHLLDSPGNIELKHSKYYQKYLNGESLNDSYSILPLQTNNNLPSLQNSTSFVDPK
VIATVLDQNLQGNELYILKMKLFESNNLMVEMNRNRQYRQENSQLKLTIENLQFQLQQAS
QAISEYEKQIQDFQINSSQQQQQVMMNTFSSPLKSPTFENQGQSMQQSEVMQQKSIQLQE
EQNKNYNLRQDLENLRTRLLQEQV

RDQEQII
KDQQNQIQ
EKNSVISSKSEEIRKLLATSESYRIEIQNLNINLSKSKQAQDDIQRQKNEQVAEFNYLISE
NKQLQAKLQNYNQDNLL
EKSDNQVVIHQNQIYNQKIEQSQQQTNYVSNKHHNTTI
EKLQLEISELKSNLIATSQQLELQQ
ERNDYLSVRLEEA
EKLNKQFNSQKPSQNVTKTIKQQSPPKYRNESELEYQQRLLEHQML
EKQHKL
ERDFQQKQLELMTQY

AVNQNTVKQIHGGSHGRVDKNEKTVKQSEQHHEQHQRSRNTDEHNQQYASQQLRQSYQQNSN
YYSQNQNINPNQHHPHHNIQNYASNKSESNSHNQQQISKQQQHNQESLNQYKQNQNQQVDNN
RFSSPPPKNIKQNIPPNHNHDYLFYQDIEEEKQKLQNQPQYIPIGVTSVNENAYVSNTQRIKD
LDLLLNQLLQKKKLAESELLKLEESKRKSSLIIIQKQELEERIRQYSQEISNVKNQLRNLHA
LK

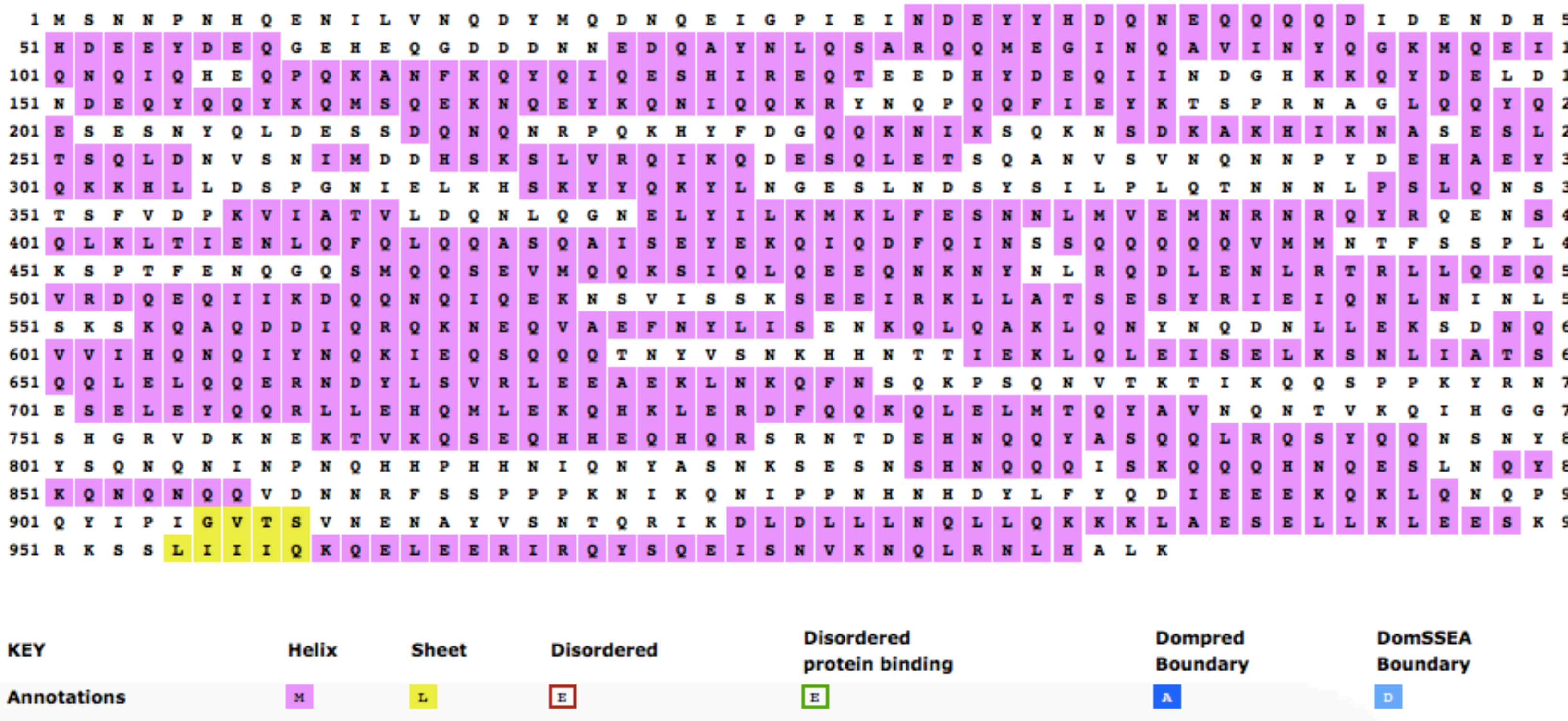

13

Tetrahymena EAS06694.1

MQETKYFNFIKETIKFYEFQLDLRYLKNIIKIQTNERQQQNKQNTKICHSEV
RYFFYPKEYFSFELTNLQKPLSQNILTIKLTVSSPHNERIAKISEKLSQVQS
NANNDRSAKLDQGEQRIKLLEDSYTEFE

EKL
EKKVTTI
RETIQKLSKLVQE
DKLN
REHHFEQKQ
KEIASLDQKLSSAIESEIASR
KEGENKITRAID
EKTSVI
RDEIS
RESKIRNETIEEINRKLEIELPKL
RDEVNN
EKVQ
REEMDQNIMKKTTEELVKLTNIIQQ
EKKDREESEQSIFDML
KEVVHRVKTEIEN
EKKN
REMTEETLL

GLLEDTCNKLNAAALV

1	M	Q	E	T	K	Y	F	N	F	I	K	E	T	I	K	F	Y	E	F	Q	L	D	L	R	Y	L	K	N	I	I	K	I	Q	T	N	E	R	Q	Q	Q	N	K	Q	N	T	K	I	C	H	S
51	E	V	R	Y	F	F	Y	P	K	E	Y	F	S	F	E	L	T	N	L	Q	K	P	S	Q	N	I	L	T	I	K	L	T	V	S	S	P	H	N	E	R	I	A	K	I	S	E	K	L	S	Q
101	V	Q	S	N	A	N	N	D	R	S	A	K	L	D	Q	G	E	Q	R	I	K	L	L	E	D	S	Y	T	E	F	E	E	K	L	E	K	K	V	T	T	I	R	E	T	I	Q	K	L	S	K
151	L	V	Q	E	D	K	L	N	R	E	H	H	F	E	Q	K	Q	K	E	I	A	S	L	D	Q	K	L	S	S	A	I	E	S	E	I	A	S	R	K	E	G	E	N	K	I	T	R	A	I	D
201	E	K	T	S	V	I	R	D	E	I	S	R	E	S	K	I	R	N	T	I	E	E	I	N	R	K	L	E	I	E	L	P	K	L	R	D	E	V	N	N	E	K	V	Q	R	E	E	M	D	Q
251	N	I	M	K	K	T	T	E	E	L	V	K	L	T	N	I	I	Q	Q	E	K	K	D	R	E	E	S	E	Q	S	I	F	D	M	L	K	E	V	V	H	R	V	K	T	E	I	E	N	E	K
301	K	N	R	E	M	T	E	E	T	L	L	G	L	L	E	D	T	C	N	K	L	N	A	A	A	L	V																								

KEY	Helix	Sheet	Disordered	Disordered protein binding	Dompred Boundary	DomSSEA Boundary

Tetrahymena thermophila Tetrin A EAR87868.2

14

MSNLDLPTSLSYFRKSPAKQYILQYESPTKVGQRDTYYSKSPLTAEARQLNN
YYDLKNTMELRYQGDLDKWKSRAQESELRKDILEKENLRLIEELNFWKTKNY
TAEREKDLEVQLKDQLAKSQADILRRSVDVQQDLLQVEIESLRNQLHEKMKE
VDEWKSLYFNQELRVSGVKQTSEQQLRIYEAQLNNILKDIQSKIDEADDLHR
RNSTLLTVPSKLASAK

KEV
KDIRSLLDMKYDES
EKLKQDMTKSQIENYSIRKNELKL
REQEQNLLAQIETIQKKIENTHELKNLEEQQA
DREHNEML
EKLKDDHQRAKEALDAKKLTYSQLIV
EKEKNLDDLRSNLEDVKRRINKT
RERELSNK
KEANDWRVRQKSIDIL
KEEEVKKIHQ
EREKSIEVLKNSHHS
EREVLEGRINHLRSDLNQ
KE
ERI
RDTSNNLARSRLARNNAEI
EKRALEADI
ERKRLLAEEA
ERKRLLELEVQEQIHK
RDIDVLRNSHNF
EKQKLEEQ
EKTLNYVLDAKQ
KEALEQK
ERARNYLISASEA
ER
KEREKSIEAELWKNTYTRT
EREKSIEVEVA
KDLRRSQIEALKRSHDYEMIQQ
ERNTAVLRNVLDSKQYEVRNL
RDSHSNIVHELINTSQDAKRANLEAQIWRNHANSQ
ERDRHTALIT
RDIVQSQELDSLRRSQLAESQYLRQEINNLANVIDHKA
REAADW
RENYNRLYNSQYRR

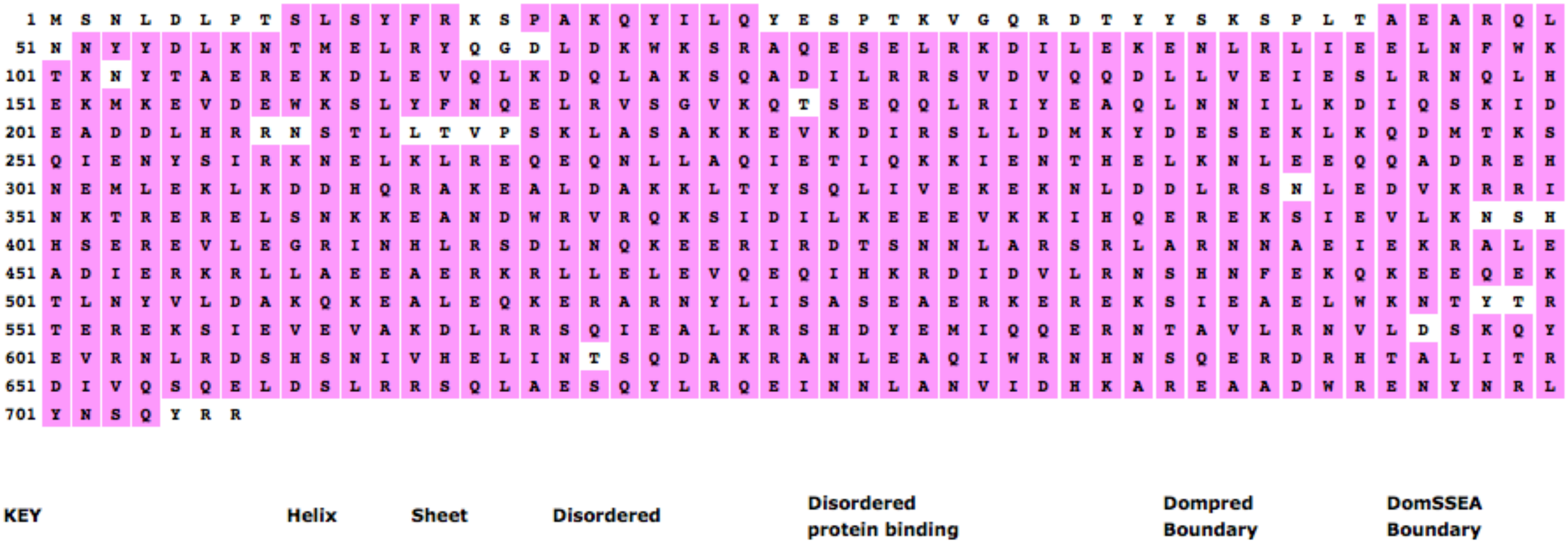

Toxoplasma IMC2A XP_002366439.1 (First half of sequence)

MERRTRPDVSPRWQARRIHFVWLTALAVPIFLLFPSLSLLESTSSAGCSG
IQCFASFP**G**VAAGSTHS

REGADPSPQETLMQPRRLSGIIKTLVLWDPVQRLMPSLNLDSVVFVAGKH
KADRSAAFHTTMDDVMTLLLRWFN
EKDKPNWLLQGFVTEAALF
REHVRYQDQMIHEQTQNQSEQQPTDPQAGM
DKVLNIEVYTYLAGAKRRVRDWICDGNTFMRTGEEGSHAFKQPFLT
ERDVGFLRGLPYNVLRVARGVYSKRVPASVKRLQEELFHRGIRTLNDLLLK
TNLMDLFVLFDIM
DESFTQIDVL
REFSNRWKKYRAEEVVPPLLPPGKWEG
ERSIEELMRDDHYPSMKFPRCTMLKCVMQDTWSKNAVETITV
EK
KDTNSLKLLLIGNTGIGEYKSRA
ERKGLWYKLKRFLWTN
EFDQTVSALAKWHAE
EKADAVLGLGDFLGIPGPLSARD
ERFTKRWYDIFV
KDAKLDIPWLMTLGEEEALVNPSASVRHHYTGEHPNWYMPNDAYTATFSFS
TSMTMANGTIQHEAFNATVINVNTWNLFVGNPIANNMQSMM
DRLMWLSDQLYTAVNQTTNWLIIMGHLPLVSTGPQGEQGRLQYVDDLYKNG
QPRGPEAVLIQMLLSHYQVDLYVSAHDHFMEYVALEDLSKNTTTAF
ITSGAAVRLL
DKDVGRGWIGRLRGALYPILCWSGRRILYAFHPGGCHP
ERWDQNQAYRFFAPVNSQYKVNIV
ERVTKATGFAALRLT
KDYLVAEFIDSRSKKLAGRRASKRSN
KDQRDIQFMDPVAEGRLRYDELEAARSAFADANE
ERIS
KEIQFARQCPILAQRIKFYQTEINDLVSKDQIGKQ
KDAYEVMDDNTIEILLSEGVNVVSRIAQAVSEMNVLAGDY
DKMVKKY
RELIDLKDTMPAADDPRYAQLFQL
EKAYIDARKMRQQTA
RELAVGRDGMQ
DREG
EKDL
ERMILNMQVIR
REIVAVENDIKANPFVPSEEQPEEGKVDVNGAETPEGGDG
KEPLPAIKYPDDSLEARI
ERK
REQLQKH
ERSLSALKRASPE
DREKIKMRIVEL
ERLIKGTQEELAAMQQRLQRQQQAMRPSRVWQLLQRRQDLETKLLQIKILVNQV
EKLPEETRTSPDARAQV
ERLTAQRVNI
EKDI
RDISDELGT
REPTEL
EKQCL
DKKASVWRALVLAQESLLS
EKQRSIPVVRDAVHAAKRSIGGLR
RELEELT
EKVKVELR

16

Toxoplasma IMC2A XP_002366439.1
(Second half of sequence)

ERDAAFDAGLEASRVPPPEAEGRKVTPVPAPHVQANLETQGRSRADALM
HEFNGGEDEIAVIDEAIRVSPLLSPE
ERAEL
EKLVGPVQDLPQKKADL
EKRLAALKDQAEQEAQEANVDPEAKRAGSGSRRLFELFEPK
KEPTPYKQLEPIDLGPVDSCLQVPMLEYHVEIALHPTMQKYIK
DKCIFIFAQMAYQTQYRVIQPVHLYLRSHKGLQTLLYRVP

SVHVFRAWNQFFGSMHNTRFKSLLKTLKKGLKFVHETFKPVGARGEALEAS

1 M E R R T R P D V S P R W Q A R R I H F V W L T A L A V P I F L L F P S L S L L E S T S S A G C S G
51 I Q C F A S F P G V A A G S T H S R E G A D P S P Q E T L M Q P R R L S G I I K T L V L W D P V Q R
101 L M P S L N L D S V V F V A G K H K A D R S A A F H T T M D D V M T L L L R W F N E K D K P N W L L
151 Q G F V T E A A L F R E H V R Y Q D Q M I H E Q T Q N Q S E Q Q P T D P Q A G M D K V L N I E V Y T
201 Y L A G A K R R V R D W I C D G N T F M R T G E E G S H A F K Q P F L T E R D V G F L R G L P Y N V
251 L R V A R G V Y S K R V P A S V K R L Q E E L F H R G I R T L N D L L L K T N L M D L F V L F D I M
301 D E S F T Q I D V L R E F S N R W K K Y R A E E V V P P L L P P G K W E G E R S I E E L M R D D H Y
351 P S M K F P R C T M L K C V M Q D T W S K N A V E T I T V E K K D T N S L K L L L I G N T G I G E Y
401 K S R A E R K G L W Y K L K R F L W T N E F D Q T V S A L A K W H A E E K A D A V L G L G D F L G I
451 P G P L S A R D E R F T K R W Y D I F V K D A K L D I P W L M T L G E E E A L V N P S A S V R H H Y
501 T G E H P N W Y M P N D A Y T A T F S F S T S M T M A N G T I Q H E A F N A T V I N V N T W N L F V
551 G N P I A N N M Q S M M D R L M W L S D Q L Y T A V N Q T T N W L I I M G H L P L V S T G P Q G E Q
601 G R L Q Y V D D L Y K N G Q P R G P E A V L I Q M L L S H Y Q V D L Y V S A H D H F M E Y V A L E D
651 L S K N T T T A F I T S G A A V R L L D K D V G R G W I G R L R G A L Y P I L C W S G R R I L Y A F
701 H P G G C H P E R W D Q N Q A Y R F F A P V N S Q Y K V N I V E R V T K A T G F A A L R L T K D Y L
751 V A E F I D S R S K K L A G R R A S K R S N K D Q R D I Q F M D P V A E G R L R Y D E L E A A R S A
801 F A D A N E E R I S K E I Q F A R Q C P I L A Q R I K F Y Q T E I N D L V S K D Q I G K Q K D A Y E
851 V M D D N T I E I L L S E G V N V V S R I A Q A V S E M N V L A G D Y D K M V K K Y R E L I D L K D
901 T M P A A D D P R Y A Q L F Q L E K A Y I D A R K M R Q Q T A R E L A V G R D G M Q D R E G E K D L
951 E R M I L N M Q V I R R E I V A V E N D I K A N P F V P S E E Q P E E G K V D V N G A E T P E G G D
1001 G K E P L P A I K Y P D D S L E A R I E R K R E Q L Q K H E R S L S A L K R A S P E D R E K I K M R
1051 I V E L E R L I K G T Q E E L A A M Q Q R L Q R Q Q Q A M R P S R V W Q L L Q R R Q D L E T K L L Q
1101 I K I L V N Q V E K L P E E T R T S P D A R A Q V E R L T A Q R V N I E K D I R D I S D E L G T R E
1151 P T E L E K Q C L D K K A S V W R A L V L A Q E S L L S E K Q R S I P V V R D A V H A A K R S I G G
1201 L R R E L E E L T E K V K V E L R E R D A A F D A G L E A S R V P P P E A E G R K V T P V P A P H V
1251 Q A N L E T Q G R S R A D A L M H E F N G G E D E I A V I D E A I R V S P L L S P E E R A E L E K L
1301 V G P V Q D L P Q K K A D L E K R L A A L K D Q A E Q E A Q E A N V D P E A K R A G S G S R R L F E
1351 L F E P K K E P T P Y K Q L E P I D L G P V D S C L Q V

Toxoplasma IMC18 EPR57770.1

MEQQQDELKHSWGANELPAGQQGSPLAERQDKTEQGKGGSAPNHQNHAHFV
ADEADCSTEDGDESLSYMRRGVYVGRGKNYWPVDYSGYPGDPAGGFRRVSIQ
ITPERMRPRKSILKSRCAAPPLDAKAKMNISFGADQVWNSDSGARPSFSDVPF**G**
RSRSFVFSE

EKNGYVDITNGEVEDVVPLGRTASVARTIVDEIQ
DRLC
ERKLTTVSILW
ERFQSHGNMG
DKTEGW

APNL**G**MPRVAMANLPPRYHVKYPGGAPRPTTCGTCGF

```
  1 M E Q Q Q D E L K H S W G A N E L P A G Q Q G S P L A E R Q D K T E Q G K G G S A P N H Q N H A H F [illegible]
 51 V A D E A D C S T E D G D E S L S Y M R R G V Y V G R G K N Y W P V D Y S G Y P G D P A G G F R R V [illegible]
101 S I Q I T P E R M R P R K S I L K S R C A A P P L D A K A K M N I S F G A D Q V W N S D S G A R P S [illegible]
151 F S D V P F G R S R S F V F S E E K N G Y V D I T N G E V E D V V P L G R T A S V A R T I V D E I Q [illegible]
201 D R L C E R K L T T V S I L W E R F Q S H G N M G D K T E G W A P N L G M P R V A M A N L P P R Y H [illegible]
251 V K Y P G G A P R P T T C G T C G F
```

Secondary structures of proteins predicted, and in most cases shown by biophysical techniques, to adopt β-sheet topologies, but lacking the amino-acid profiles of epiplastins

19

IgG Heavy chain (red and gray), mouse AAA51043.1

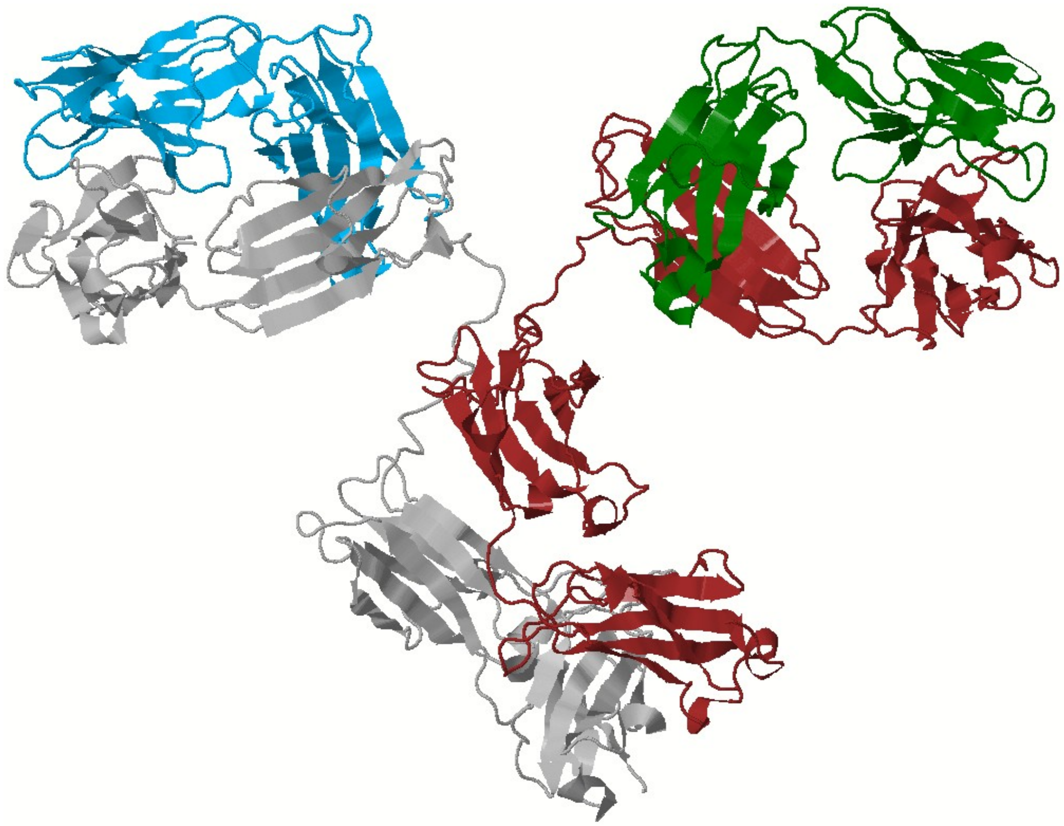

```
  1 M G W T W I F I L I L S V T T G V H S D V Q L Q Q S G P E L E K P G A S V K I S C K A S G F S L P G
 51 H N I N W I V Q R N G K S L E W I G N I D P Y Y G G T N F N P K F K G K A T L T V D K S S S T L Y M
101 H L T S L Q S E D S A V Y Y C A R R R D G N Y G F T Y W G Q G T L V T V S A A K T T P P S V Y P L A
151 P G S A A Q T N S M V T L G C L V K G Y F P E P V T V T W N S G S L S S G V H T F P A V L Q S D L Y
201 T L S S S V T V P S S T W P S E T V T C N V A H P A S S T K V D K K I V P R D C G C K P C I C T V P
251 E V S S V F I F P P K P K D V L T I T L T P K V T C V V V D I S K D D P E V Q F S W F V D D V E V H
301 T A Q T Q P R E E Q F N S T F R S V S E L P I M H Q D W L N G K E F K C R V N S A A F P A P I E K T
351 I S K T K G R P K A P Q V Y T I P P P K E Q M A K D K V S L T C M I T D F F P E D I T V E W Q W N G
401 Q P A E N Y K N T Q P I M N T N G S Y F V Y S K L N V Q K S N W E A G N T F T C S V L H E G L H N H
451 H T E K S L S H S P G K
```

KEY	Helix	Sheet	Disordered	Disordered protein binding	Dompred Boundary	DomSSEA Boundary
Annotations	M	L	E	E	A	D

20

Muscle titin modules (human)

upper: PDB 1TIU

lower: PDB 2RQ8_A

1 M H H H H H H S S L I E V E K P L Y G V E V F V G E T A H F E I E L S E P D V H G Q W K L K G Q P L

51 T A S P D C E I I E D G K K H I L I L H N C Q L G M T G E V S F Q A A N A K S A A N L K V K E L

KEY Helix Sheet Disordered Disordered Dompred DomSSEA

1 M L I E V E K P L P G V E V F V G E T A H F E I E L S E P D V H G Q W K L K G Q P L A A S P D C E I

51 I E D G K K H I L I L H N C Q L G M T G E V S F Q A A N T K S A A N L K V K E L L E H H H H H H

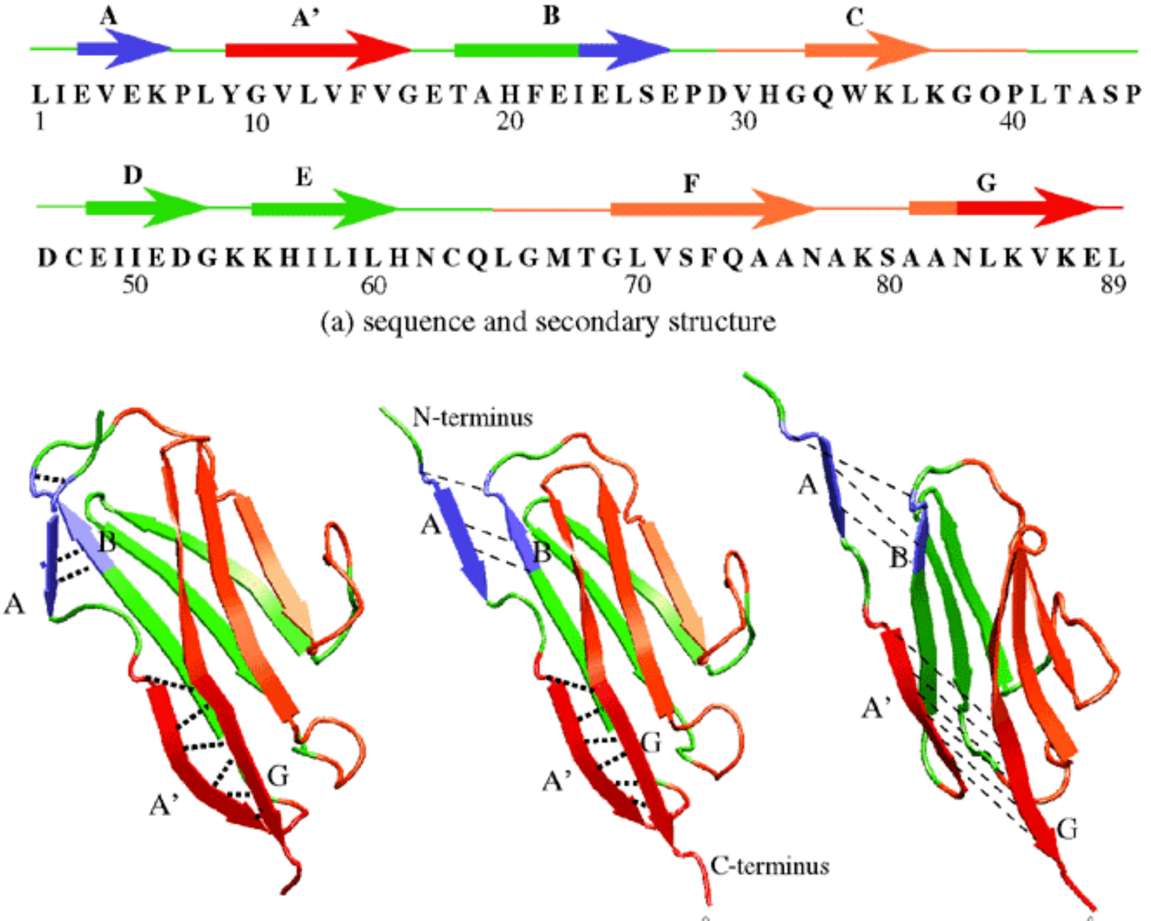

(a) sequence and secondary structure

(b) native structure (c) extension of 10 Å (d) extension of 25 Å

β-Keratins

Chicken NP_001001310.2

1 M S C Y K E M I S S R C L P P C E V T C P Q P Y A D A C S Q P C V T S C G D S R A V V Y P P P V V I [illegible]
51 T F P G P I L S S C P Q E S I V G S S A P A G I G S S F G Y E S S L G I R E L S G F F G P P M A T G [illegible]
101 S P Y K Y G R S F S S Y G Y G G Y G A G S C R P C

Gekko ABU98606.1

1 M S Y C G P S F A V P S Y A S T P A I G F G S A G F G Y G G L H S G T I I G S G S P A F A V P S Y A
51 S A P A V G F G S V G L G Y G G L H S G T L I G A G S P S F A V P S V A S A P V V G F G S A S L G Q
101 N T G V P S A S L G I L S G V N P S A I N Q I P P A E V V I Q P P P S V V T L P G P I L S A T G E P
151 V S V G G N T P C A V S Y G G S S S G L S I G S G L Y G G F S R G L S G G S F G A I G G R L G S Y G
201 G R R G S L I L G R R G S T C L V P Q

Finch OWK51963.1

1 M S C Y D L C R P C G P T P L A N S C N E P C V R Q C Q D S R V V I Q P S P V V V T L P G P I L S S
51 F P Q N T A V G S S T S A A V G S I L S E E G V P I N S G G F G L S G L S G L E F P P A T A M S C Y
101 D L C A P T S C G P T P L A N S C N E P C V R Q C Q D S T V V I Q P S P V V V T L P G P I L S S F P
151 Q N T T V G S S A S A A V G S A L S A G G V P I S S G S S L G F G G F G Y P G L G S G Y S R P Y R R
201 Y N A S R S G F Y G P C

Skink CCK73385.1

1 M A A C G P S C T V P S C A S S P V G G F G S G G I G G G Y G G L G Y G F G G L G Y G Y G A G G L A
51 E T S G D L G T L A G V I P S C I N Q I P P A E V V I Q P P A S I V T I P G P I L F A S C E P V A V
101 G G I T P C A A G G S G V T G S G L L G S G L Y G G F G Y G G L G Y G G L G Y G Y G L R R G G F F G
151 R R S L L R R R G N I C Y

22

Porin (*Mesorhizobium opportunistum*) AEH89755.1

MNIKSLLLGSAAALIAVSGARAADAVVVAEPEPAEYVKICDVYGAGYFYIPG
TETCLRIGGYIRYDIGAGDVNGLTDVNDRSDPGSLNDTFYKHARFALKTWT
GQETELGTLKTYTETRFNWETQREGGNYAVAAGRGVSLNFAWIQLGGFR
VGKDESAWVTFSGYAGNVIDDSLVGYGGDFDTGVVQYYFDAGNGFSAVV
SLEDGAGANTVDSYVPHIVGGVKYTQGWGSISATGVYDSVWEEWGGKVR
VDVNASDALSLWAMVNYGSESNVIHNSFKNWEGNWAVFGGGTYKFNEKT
SFNAQVSYDEGKSLGVAANIAYNIVPGFAIIGEVDYVNKATSDYRAVSSDGF
GGMLRFQRDF

1 M N I K S L L L G S A A A L I A V S G A R A A D A V V V A E P E P A E Y V K I C D V Y G A G Y F Y I
51 P G T E T C L R I G G Y I R Y D I G A G D V N G L T D V N D R S D P G S L N D T F Y K H A R F A L K
101 T W T G Q E T E L G T L K T Y T E T R F N W E T Q R E G G N Y A V A A G R G V S L N F A W I Q L G G
151 F R V G K D E S A W V T F S G Y A G N V I D D S L V G Y G G D F D T G V V Q Y Y F D A G N G F S A V
201 V S L E D G A G A N T V D S Y V P H I V G G V K Y T Q G W G S I S A T G V Y D S V W E E W G G K V R
251 V D V N A S D A L S L W A M V N Y G S E S N V I H N S F K N W E G N W A V F G G G T Y K F N E K T S
301 F N A Q V S Y D E G K S L G V A A N I A Y N I V P G F A I I G E V D Y V N K A T S D Y R A V S S D G
351 F G G M L R F Q R D F

23

Trichomonas vaginalis EAX91117.1

MADELKLCSTIGFSGQVAGGLIAHPDNKHIIYPLGSILVVMEKGKAATQRFL
TGHTSEITAIAVSRSGHYIASGQYSAIDQESTLILWDFDKMAQVAKWTMH
KDSIRCLSFSMSDKYLASLGGDDRIVIWDVARRAGLNGSTATIGSTGGCN
CVGFSNNDDQFFVSAGDTNVRFWRIDEERRNFTADNMKLDITKRNVTAL
SLDANDTYVYCGTTTGDVLKVHCEQKKLITVGPRKPIGEGITALQVTPWG
DIAVGSGCGRVAILDAGDLHAITGTDLQGRVTSVSVVPRTNSEILCGTSES
DICSINTDTFKASILSKGHSSAISDVFFPERSSDMFLTCSSGGFHVWNSRT
YQELLRVSLARSECNCIAVPADGKIILTGWSDGRIRGYAPQSGRELWVING
AHLNGVTAIAARGSFIVTGGMTGDISIWELGAKNMHLVKTLKEHHQMVSQI
KFSRDGNEFWSCSHDGSVIIWDANRVVSRQRFMQQAFFNGADVHTETGI
LVTVSSDKRIVFWDGFNASIIRELEASVNAQPNSICLSPDETKFVTGGDDK
LVKVWGFQTGQLEALGKGHCGNIKKAIYSPDQSIIVSVGAEGGIYIWKMK

1 M A D E L K L C S T I G F S G Q V A G G L I A H P D N K H I I Y P L G S I L V V M E K G K A A T Q R
51 F L T G H T S E I T A I A V S R S G H Y I A S G Q Y A I D Q E S T L I L W D F D K M A Q V A K W T M
101 H K D S I R C L S F S M S D K Y L A S L G G D D R I V I W D V A R R A G L N G S T A T I G S T G G C
151 N C V G F S N N D D Q F F V S A G D T N V R F W R I D E E R R N F T A D N M K L D I T K R N V T A L
201 S L D A N D T Y V Y C G T T T G D V L K V H C E Q K K L I T V G P R K P I G E G I T A L Q V T P W G
251 D I A V G S G C G R V A I L D A G D L H A I T G T D L Q G R V T S V S V V P R T N S E I L C G T S E
301 S D I C S I N T D T F K A S I L S K G H S S A I S D V F F P E R S S D M F L T C S S G G F H V W N S
351 R T Y Q E L L R V S L A R S E C N C I A V P A D G K I I L T G W S D R I R G Y A P Q S G R E L W V I
401 N G A H L N G V T A I A A R G S F I V T G G M T G D I S I W E L G A K N M H L V K T L K E H H Q M V
451 S Q I K F S R D G N E F W S C S H D G S V I I W D A N R V V S R Q R F M Q Q A F F N G A D V H T E T
501 G I L V T V S S D K R I V F W D G F N A S I I R E L E A S V N A Q P N S I C L S P D E T K F V T G G
551 D D K L V K V W G F Q T G Q L E A L G K G H C G N I K K A I Y S P D Q S I I V S V G A E G G I Y I W
601 K M K
