# Supplementary material for: Epiplasts: Membrane Skeletons and Epiplastin Proteins in Euglenids, Glaucophytes, Cryptophytes, Ciliates, Dinoflagellates, and Apicomplexans
Source: mBio. 2018 Oct 30;9(5):e02020-18. doi: 10.1128/mBio.02020-18 (PMC6212826; doi:10.1128/mBio.02020-18)
Supplement: TABLE S1 [file mbo005184120st1.docx]

Genome resources used in this study

| Classification | | Species | IMCp domain | Genome/EST | Sources |
| --- | --- | --- | --- | --- | --- |
| Viridiplantae | |  |  |  |  |
|  | Embryophyta | Arabidopsis thaliana | no | Genome | https://www.arabidopsis.org/download/index-auto.jsp?dir=/download_files/Genes |
|  | Embryophyta | Oryza sativa | no | Genome | http://www.plantgdb.org/XGDB/phplib/download.php?GDB=Os |
|  | Embryophyta | Brachypodium | no | Genome | Genbank: GCA_000005505.4 |
|  | Embryophyta | Picea abies | no | Genome | Spruce Genome Project |
|  | Embryophyta | Aquilegia coerulea | no | Genome | Phytozome |
|  | Embryophyta | Selaginella moellendorfii | no | Genome | Phytozome |
|  | Embryophyta | Physcomitrella patens | no | Genome | Physcomitrella patens v3.3, DOE-JGI, http://phytozome.jgi.doe.gov/ |
|  | Embryophyta | Mercantia polymorpha | no | Genome | Phytozome |
|  | Charophyta | Klebsormidium flaccidum | no | Genome | http://www.plantmorphogenesis.bio.titech.ac.jp/~algae_genome_project/klebsormidium/ |
|  | Chlorophyceae-V | Chlamydomonas reinhardtii | no | Genome | Phytozome |
|  | Chlorophyceae-V | Volvox cateri f. nagariensis | no | Genome | Phytozome |
|  | Chlorophyceae-V | Gonium pectorale | no | Genome | Genbank: GCA_001584585.1 |
|  | Chlorophyceae-S | Chromochloris zofingiensis SAG 211–14 | no | Genome | Phytozome |
|  | Trebouxiophyceae | Chlorella sp. NC64A | no | Genome | Phytozome |
|  | Trebouxiophyceae | Cocomyxa subellipsoidea C-169 | no | Genome | Phytozome |
|  | Trebouxiophyceae | Asterochloris sp. Cgr/DA1pho | no | Genome | Phytozome |
|  | Trebouxiophyceae | Auxenochlorella protothecoides sp 0710 | no | Genome | JGI: MycoCosm |
|  | Prasinophyta | Micromonas commoda RCC299 | no | Genome | http://genome.jgi-psf.org/MicpuN3/MicpuN3.download.ftp.html |
|  | Prasinophyta | Micromonas pusilla CCMP1545 | no | Genome | http://genome.jgi-psf.org/pages/dynamicOrganismDownload.jsf?organism=MicpuC3 |
|  | Prasinophyta | Ostreococcus tauri RCC4221 | no | Genome | http://genome.jgi-psf.org/Ostta4/Ostta4.download.ftp.html |
|  | Prasinophyta | Ostreococcus lucimarinus CCE9901 | no | Genome | http://genome.jgi-psf.org/Ost9901_3/Ost9901_3.download.ftp.html |
|  | Prasinophyta | Bathycoccus prasinos RCC1105 | no | Genome | https://bioinformatics.psb.ugent.be/gdb/bathycoccus/ |
| Rhodophyta | |  |  |  |  |
|  | Cyanidiophyceae | Cyanidiozyon merolae | no | Genome | http://merolae.biol.s.u-tokyo.ac.jp/ |
|  | Cyanidiophyceae | Galdieria sulfuraria | no | Genome | http://genomics.msu.edu/galdieria/sequence_data.html; Genbank |
|  | Florideophyceae | Chondrus crisus | no | Genome | GenBank: CAKH01000001–CAKH01003241 |
|  | Bangiophyceae | Pyropia yezoensis | no | Genome | http://nrifs.fra.affrc.go.jp/ResearchCenter/5_AG/genomes/nori/ |
|  | Bangiophyceae | Porphyridium purpureum | no | Genome | http://cyanophora.rutgers.edu/porphyridium/ |
| Glaucophyta | |  |  |  |  |
|  | Glaucophyceae | Cyanophora paradoxica | yes | Genome | http://cyanophora.rutgers.edu/cyanophora/blast.php |
|  | Glaucophyceae | Cyanoptyche_gloeocystis | yes | EST | MMETSP1086 |
|  | Glaucophyceae | Gloeochaete_witrockiana | no | EST | MMETSP0308 |
|  | Glaucophyceae | Gloeochaete_wittrockiana | no | EST | MMETSP1089 |
| Cryptophyte | |  |  |  |  |
|  | Pyrenomonadales | Guilardia theta | yes | Genome | JGI:MycoCosm |
|  | Pyrenomonadales | Rhodomonas salina | yes | EST | MMETSP1047 |
|  | Goniomonadida | Goniomonas pacifica CCMP1869 | yes | EST | MMETSP0107 |
| Haptophyte | |  |  |  |  |
|  | Prymnesiales | Prymnesium parvum | no | EST | MMETSP0006 |
| Alveolata | |  |  |  |  |
|  |  | Chromera velia | yes | Genome | MMETSP0290 |
| Rhizaria | |  |  |  |  |
|  | Chlorarachniophyta | Bigelowiella natans | no | Genome | JGI:MycoCosm |
|  | Cercozoa | Latharella globosa CCCM811 | no | EST | MMETSP0111 |
|  | Cercozoa | Lotharella amoebiformis CCMP2058 | no | EST | MMETSP0042 |
|  | Cercozoa | Plasmodiophora brassicae | no | Genome | Genbank: GCA_001049375.1 |
|  | Foraminifera | Reticulomyxa filosa | no | Genome | Genbank: GCA_000512085.1 |
|  | Foraminifera | Ammonia sp. | no | EST | MMETSP1384 |
|  | Foraminifera | Elphidium margaritaceum | no | EST | MMETSP1385 |
| Amoebozoa | |  |  |  |  |
|  | Mycetozoa | Dictyostelium discoideum | no | Genome | DictyBase |
|  | Mycetozoa | Dictyostelium purpureum | no | Genome | DictyBase |
|  | Discosea | Acanthamoeba castellani | no | Genome | Genbank: GCA_000313135.1 |
|  | Archamoebae | Entamoeba histolytica | no | Genome | Genbank: GCA_001662325.1 |
|  |  | Polysphondylium pallidum | no | Genome | Genbank: GCA_000004825.1 |
| Fungi | |  |  |  |  |
|  | Chytridiomycota | Spizellomyces punctatus | no | Genome | JGI:MycoCosm |
| Metazoa | |  |  |  |  |
|  | Sea anemone | Nematostella vectensis | no | Genome | JGI:MycoCosm |
|  | Sea snail | Lottia gigantea | yes | Genome | JGI:MycoCosm |
|  | Vertebrata | Xenopus tropicalis | no | Genome | Genbank: GCA_000004195.1 |
| Proto-opisthokont | |  |  |  |  |
|  | Ichthyosporea | Capsaspora owczarzki | yes | Genome | Genbank: GCA_000151315.2 |
|  | Ichthyosporea | Sphaeroforma arctica | yes | Genome | Genbank: GCA_001186125.1 |
|  | Apusozoa | Thecamonas trahens | no | Genome | Genbank: GCA_000142905.1 |
|  | Apusozoa-Amoebozoa | Stygamoeba regulata | no | EST | MMETSP0447 |
|  | Choanoflagellida | Monosiga brevicolis | no | Genome | JGI:MycoCosm |
|  | Choanoflagellida | Salpingoeca rosetta | no | Genome | Genbank: GCA_000188695.1 |
|  | Slime mold | Fonticula alba | no | Genome | Genbank: GCA_000388065.1 |
| Excavata | |  |  |  |  |
|  | Discoba | Percolomonas cosmopolitus | weak | EST | MMETSP0759 |
|  | Discoba | Neobodo designis | no | EST | MMETSP1114 |
|  | Kinetoplastida | Angomonas deanei | no | Genome | Genbank: GCA_000442575.2 |
|  | Kinetoplastida | Leishmania major | no | Genome | Genbank: GCA_000002725.2 |
|  | Kinetoplastida | Trypanosoma cruzi | no | Genome | TcruziDB |
|  | Kinetoplastida | Trypanosoma brucei | no | Genome | https://www.sanger.ac.uk/resources/downloads/protozoa/trypanosoma-brucei.html |
|  | Kinetoplastida | Leptomonas seymouri | no | Genome | Genbank: GCA_001299535.1 |
|  | Kinetoplastida | Bodo saltans | no | Genome | https://www.sanger.ac.uk/resources/downloads/protozoa/bodo-saltans.html |
|  | Euglenida | Eutreptiella gymnastica | yes | EST | MMETSP0039 |
|  | Schizopyrenida | Naegleria gruberi | yes | Genome | JGI:MycoCosm |
|  | Trichomonadida | Trichomonas vaginalis G3 | no | Genome | ASM282v1:DS113702 |
|  | Diplomonads | Giardia lamblia ATCC_50803 | no | Genome | Genbank: GCA_000002435.1 |
|  | Placozoa | Trichoplax adhaerens Grell-BS-1999 | no | Genome | JGI:Mycocosm |

Species-specific epiplastin summary

|  | Species | # epiplastin candidates analyzed | Average size of ABD domain (aa) | VI content (%) | Charged aa content (%) |
| --- | --- | --- | --- | --- | --- |
| Excavata | Euglena gracilis | 7 | 484.57 | 42 | 33 |
|  | Eutreptiella gymnastica | 20 | 319.35 | 41 | 37 |
|  |  |  |  |  |  |
| Cryptophyta | Chroomonas mesostigmatica | 15 | 192.87 | 40 | 38 |
|  | Goniomonas pacifica | 18 | 227.89 | 30 | 39 |
|  | Guillaridia theta | 8 | 188.00 | 34 | 34 |
|  | Rhodomonas salina | 10 | 277.30 | 38 | 39 |
|  |  |  |  |  |  |
| Glaucophyta | Cyanophora paradoxa | 5 | 143.40 | 35 | 41 |
|  | Cyanoptyche gloeocystis | 2 | 136.50 | 42 | 38 |
|  | Gloeochaete witrockiana | 2 | 214.00 | 38 | 34 |
|  |  |  |  |  |  |
| Alveolata-Api Chromerid | Chromera velia | 13 | 177.92 | 30 | 34 |
|  | Vitrella brassicaformis | 11 | 179.09 | 35 | 33 |
|  |  |  |  |  |  |
| Alveolata-Api parasite | Babesia bovis | 2 | 145.00 | 35 | 39 |
|  | Cryptosporidium parvum | 5 | 194.40 | 32 | 33 |
|  | Cryptosporidium ubiquitum | 2 | 170.50 | 30 | 34 |
|  | Cyclospora cayetanensis | 1 | 182.00 | 28 | 35 |
|  | Eimeria necatrix | 1 | 151.00 | 29 | 36 |
|  | Hammondia hammondi | 1 | 104.00 | 38 | 37 |
|  | Plasmodium falciparum | 16 | 185.31 | 28 | 34 |
|  | Toxoplasma gondii | 14 | 184.71 | 31 | 33 |
|  |  |  |  |  |  |
| Alveolata- Ciliate | Paramecium tetraurelia | 15 | 102.73 | 24 | 34 |
|  | Pseudocohnilembus persalinus | 3 | 592.33 | 40 | 39 |
|  | Pseudomicrothorax dubius | 3 | 150.33 | 38 | 33 |
|  | Tetrahymena thermophila | 4 | 161.00 | 24 | 37 |
|  |  |  |  |  |  |
| Alveolata-Dinoflagellate | Karlodinium veneficum | 3 | 379.00 | 37 | 20 |
|  | Kryptoperidinium foliaceum | 17 | 236.59 | 33 | 32 |
|  | Oxyrrhis marina | 2 | 171.50 | 37 | 29 |
|  | Symbiodinium sp. | 19 | 263.68 | 32 | 33 |
|  |  |  |  |  |  |
|  |  |  | **Average** |  |  |
|  |  |  | 219.07 | 34 | 35 |
