# Supplementary material for: Epiplasts: Membrane Skeletons and Epiplastin Proteins in Euglenids, Glaucophytes, Cryptophytes, Ciliates, Dinoflagellates, and Apicomplexans
Source: mBio. 2018 Oct 30;9(5):e02020-18. doi: 10.1128/mBio.02020-18 (PMC6212826; doi:10.1128/mBio.02020-18)
Supplement: TEXT S9 [file mbo005184120s9.pdf]

1. *Cyanophora* epiplasts

2. Epiplast plates with mucocysts (m) in suture (s) corners

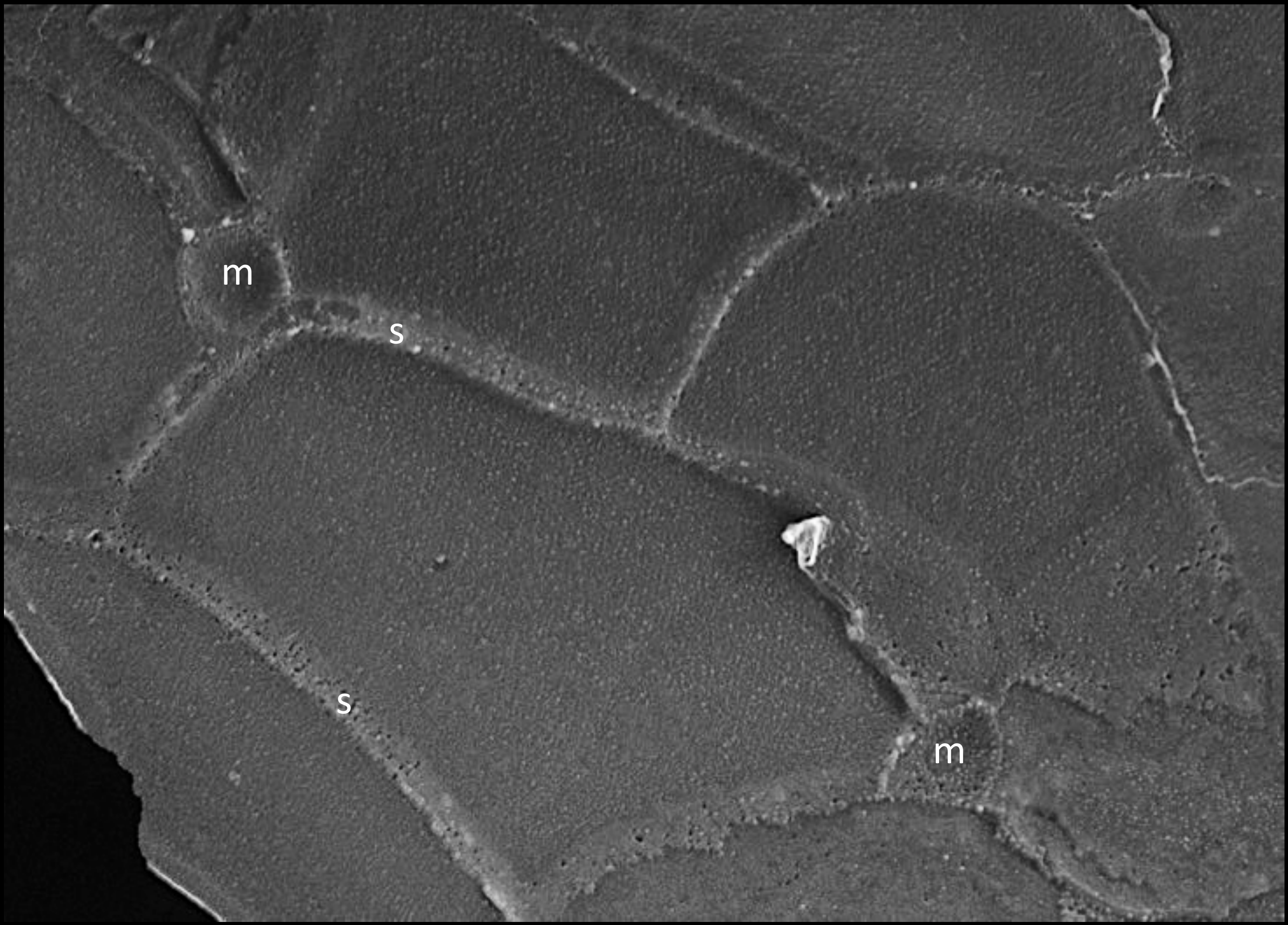

3. Epiplast plates with mucocysts (m) in suture (s) corners

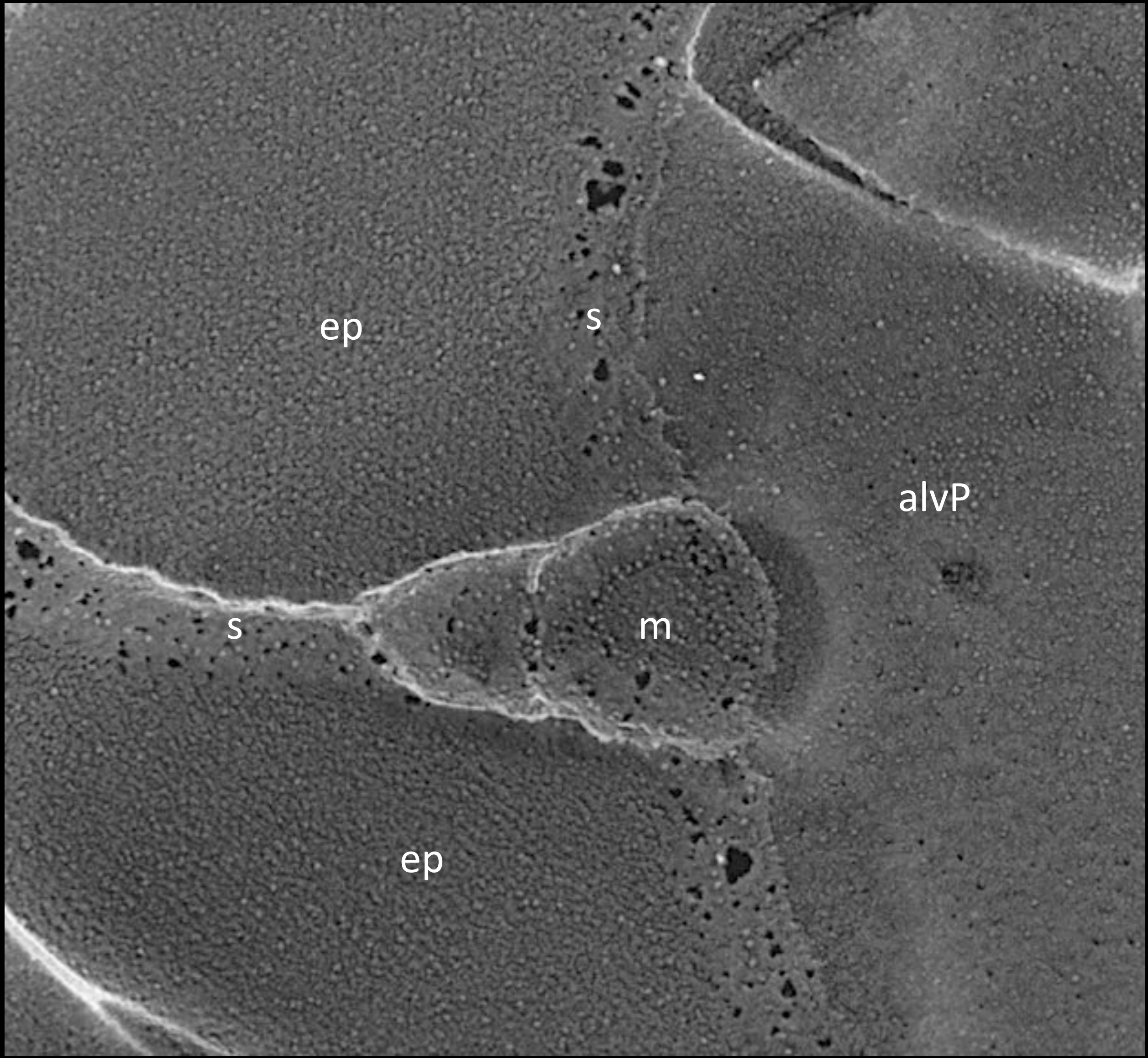

4. Alveolar membranes and region where fracture exposes an underlying epiplast plate (arrow)

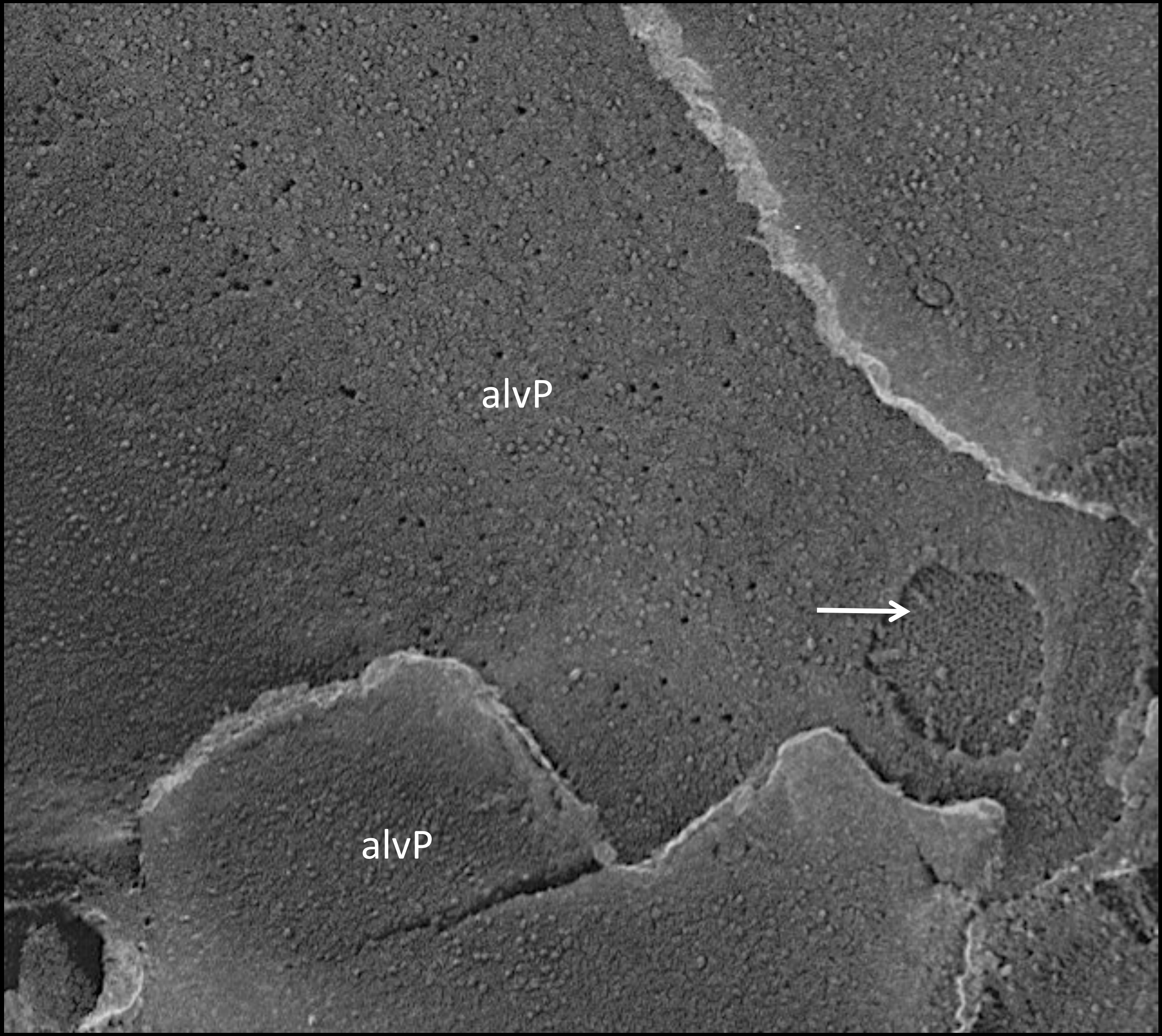

Cyanophora ghosts

6. Stationary-phase culture: “shells” (arrows) around cells and in the media. Smaller round profiles (arrowheads) may represent the peptidoglycan layer of plastids.

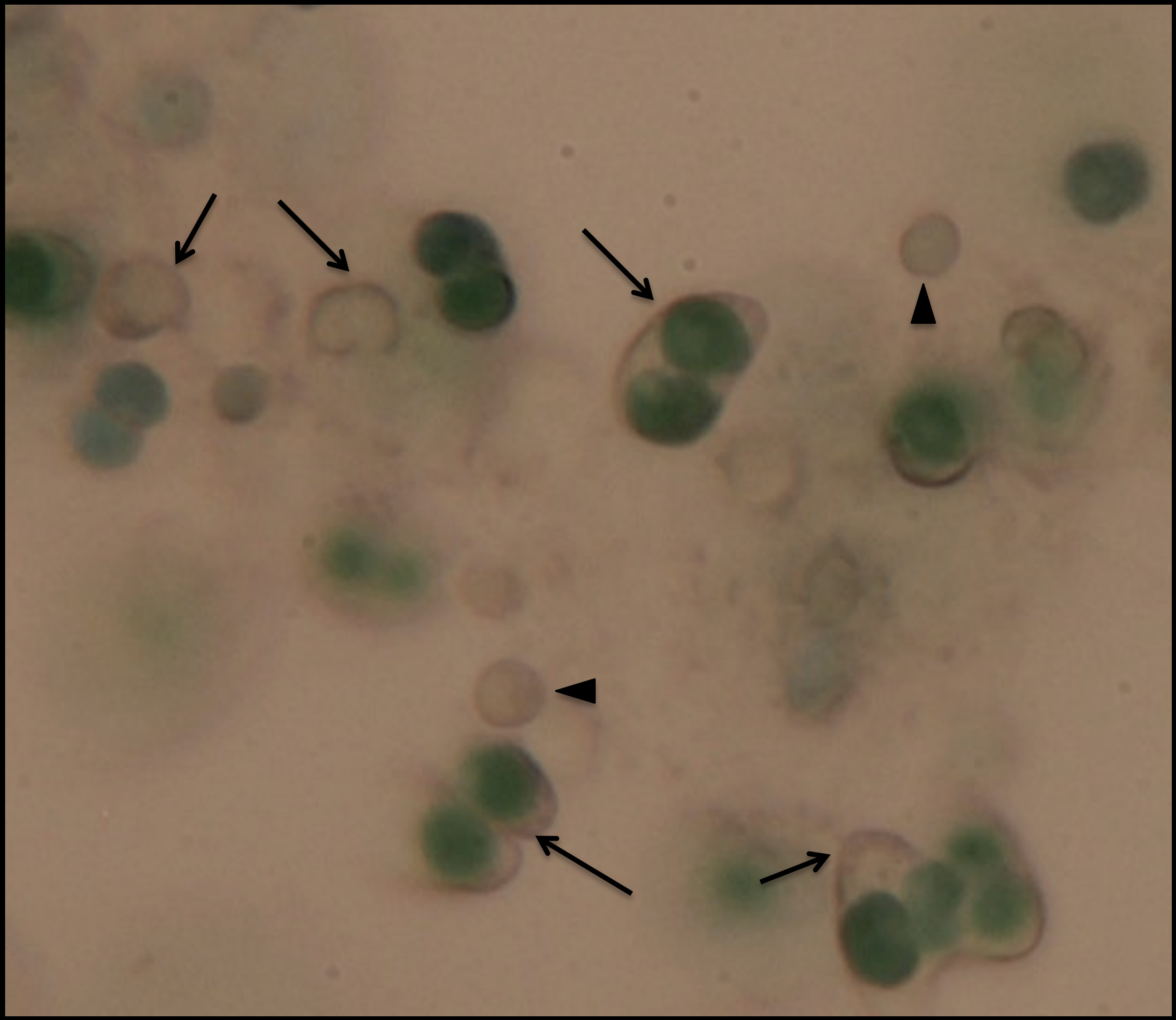

7. Control cells fixed with 1% glutaraldehyde

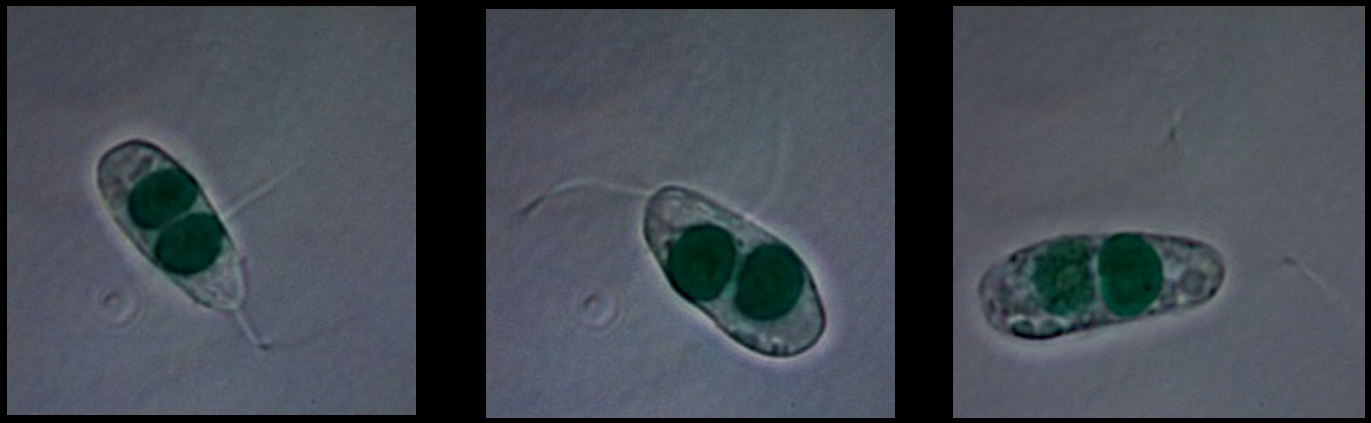

Cells treated 3 min with NP-40 detergent followed by 1% glutaraldehyde

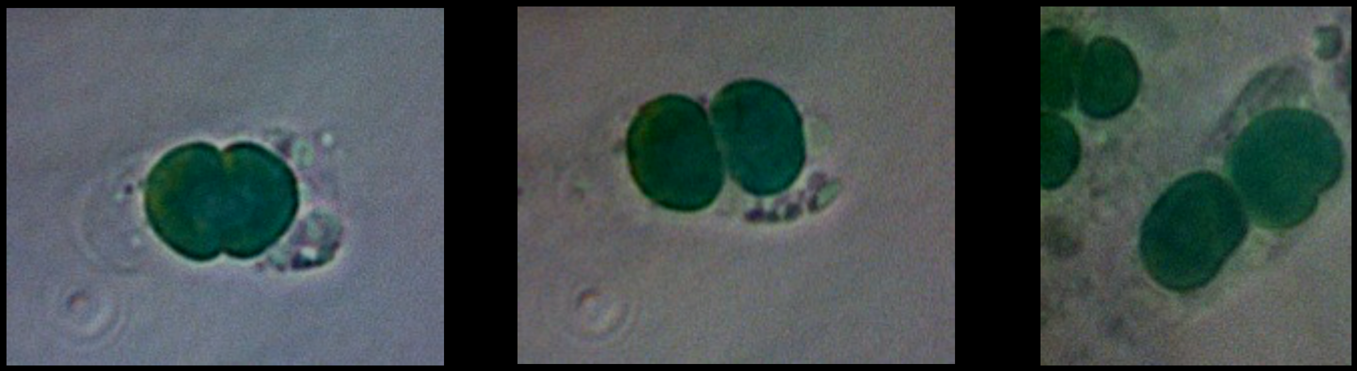

0.05% NP-40 1% NP-40 5% NP-40

8. Control cells suspended in 1M glycerol + 5% sucrose, then fixed with 1% glutaraldehyde

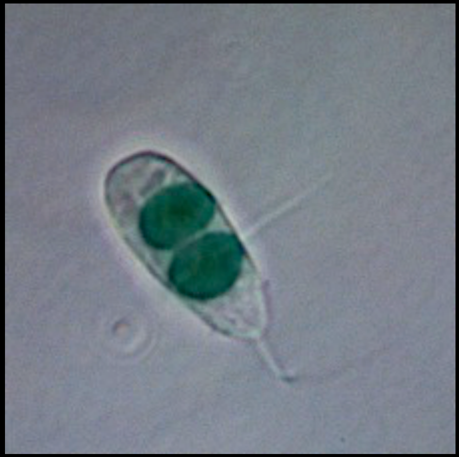

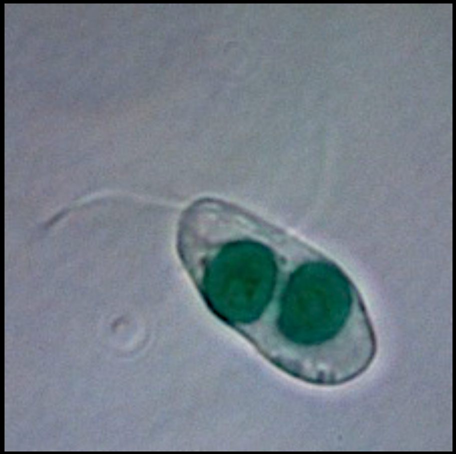

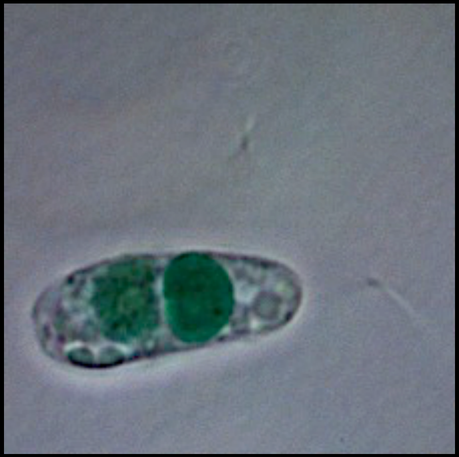

Cells treated 3 min with NP-40 detergent followed by 1M glycerol + 5% sucrose, then fixed with 1% glutaraldehyde

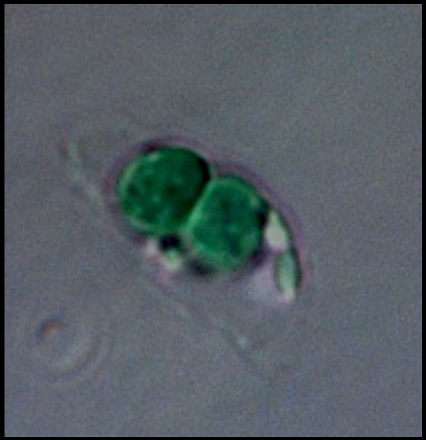

0.05% NP-40

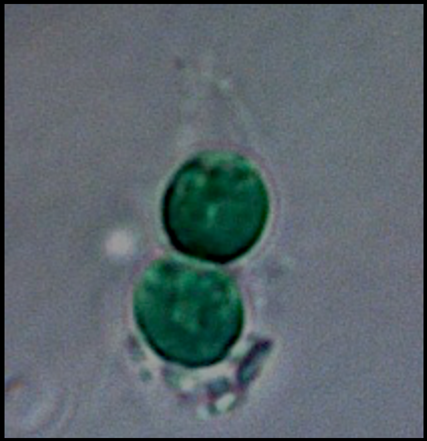

1% NP-40

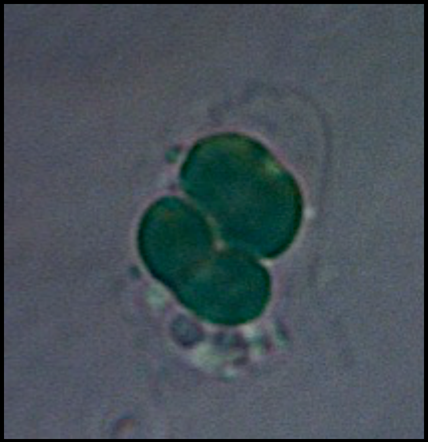

5% NP-40

9. SYBR green II signal (DNA) is lost with NP-40 treatment

Control

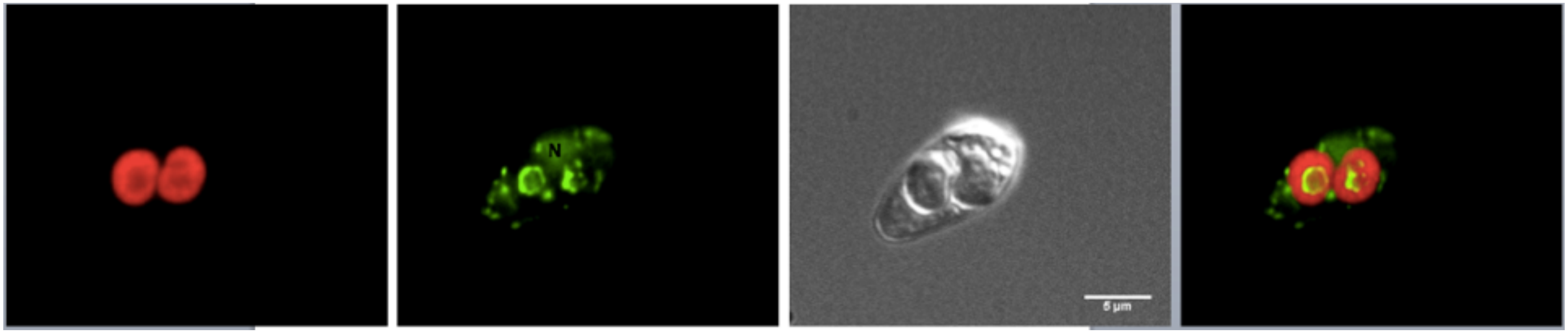

Chlorophyll | SYBR green II signal | DIC | Overlay

1% NP-40 treated

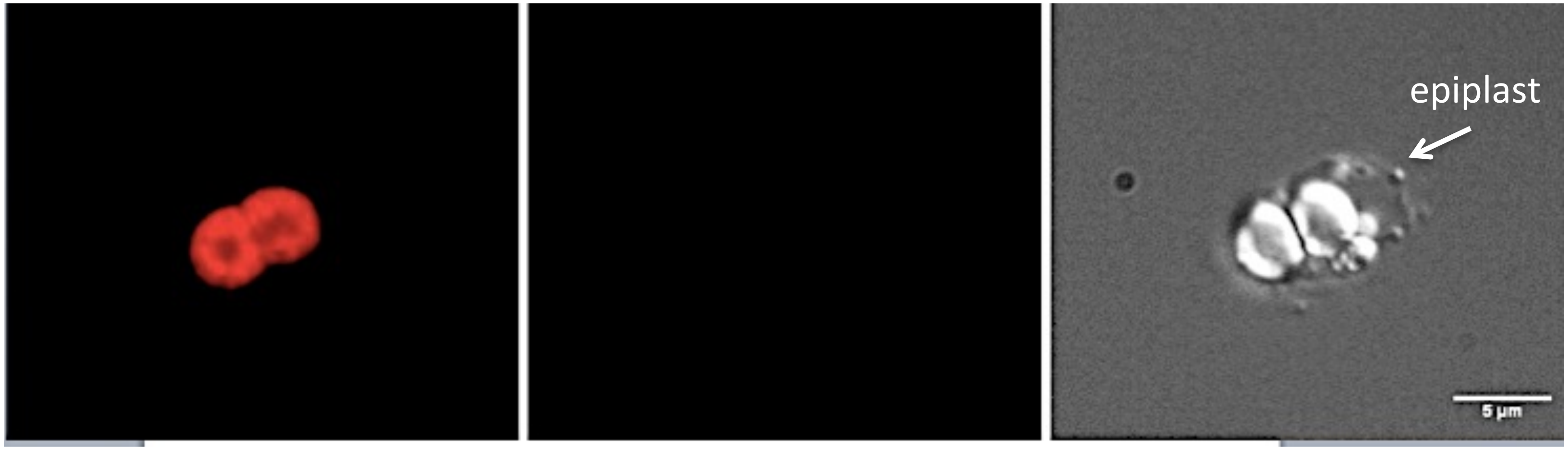

Chlorophyll | SYBR green II signal | DIC

10. *Neospora caninum* (a close relative of *Toxoplasma gondii*)

Legend for slide 10.10

A) Cross-fracture of cell surface membranes. Arrows indicate plasma membrane (pm) and epiplast (ep), between which are the two alveolar membranes separated by a narrow lumen.

B) Alveolar P fracture face displaying a row of double particle units (double arrows) beneath a microtubule (mt) and rows of single particles (single arrows).

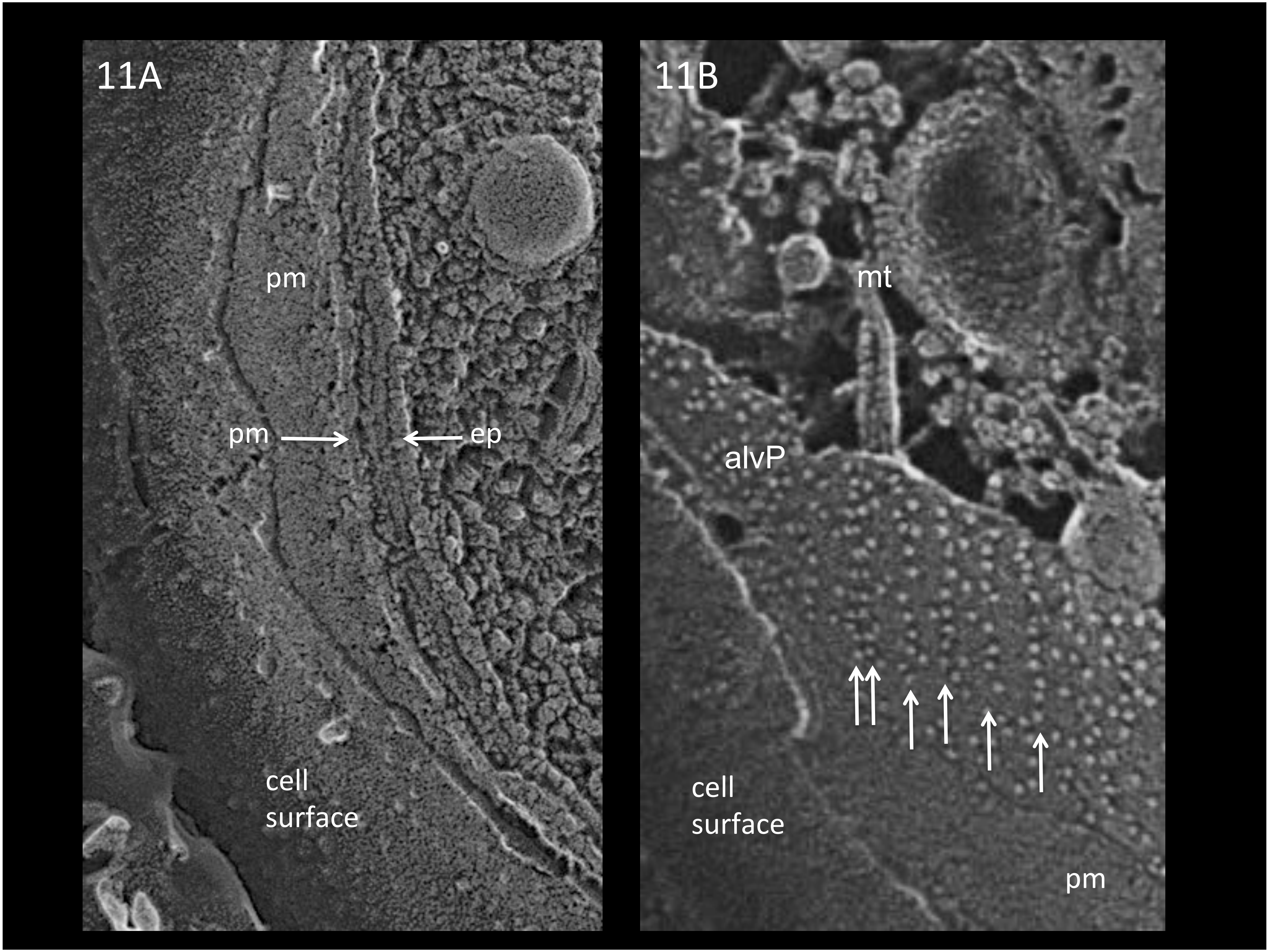
11A
pm
pm
ep
cell
surface
11B
mt
alvP
cell
surface
pm

12. *Toxoplasma* Stereo images of text Fig. 6
(use red/green glasses)

13

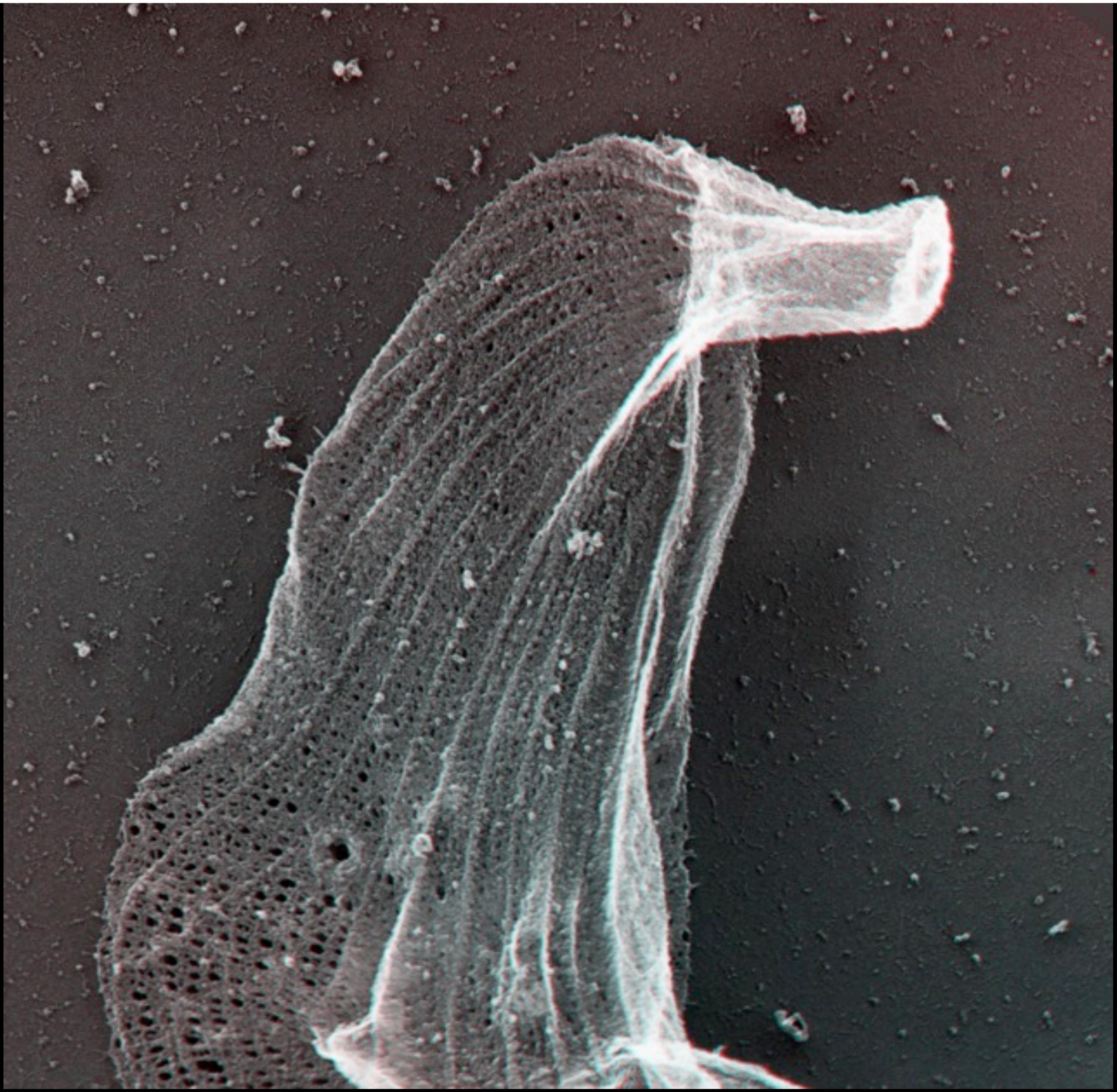

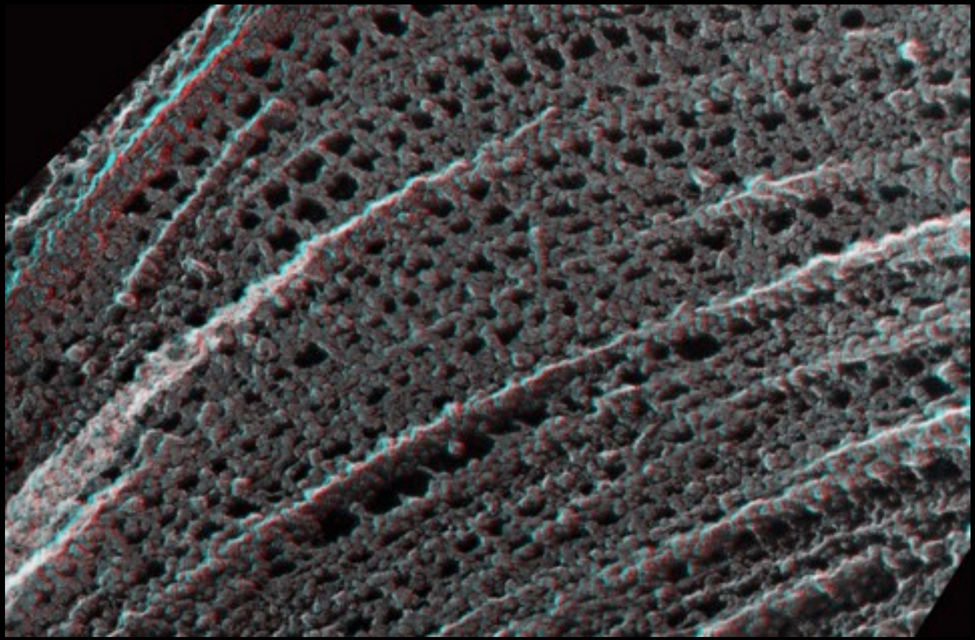

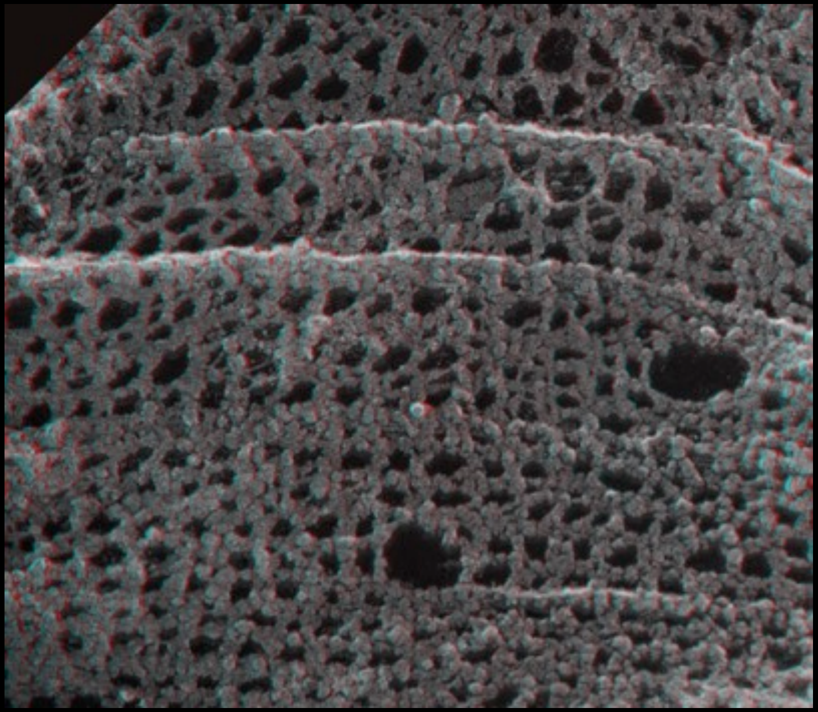

15. Dinoflagellates

Abbreviations:

PI, algaenan-like outer layer of pellicle wall
PII, cellulosic pellicle wall layer
White arrows, microtubules
al, alveolar cisternae
cingulum, groove around cell midline containing flagellum

16. Empty alveoli and epiplast cross-fractures (red arrows) in *Glenodinium foliacium*

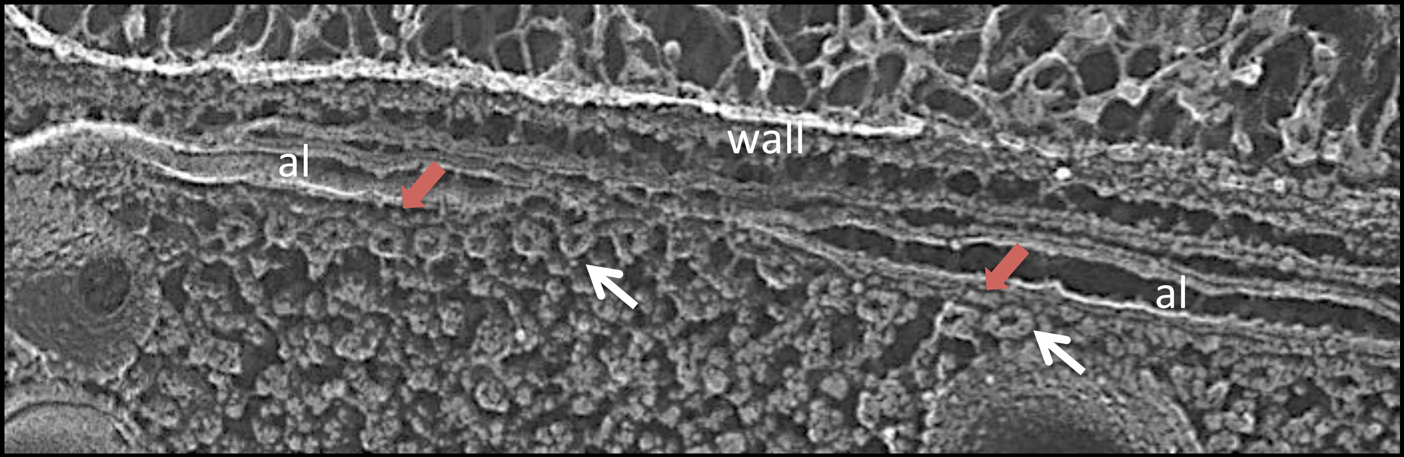

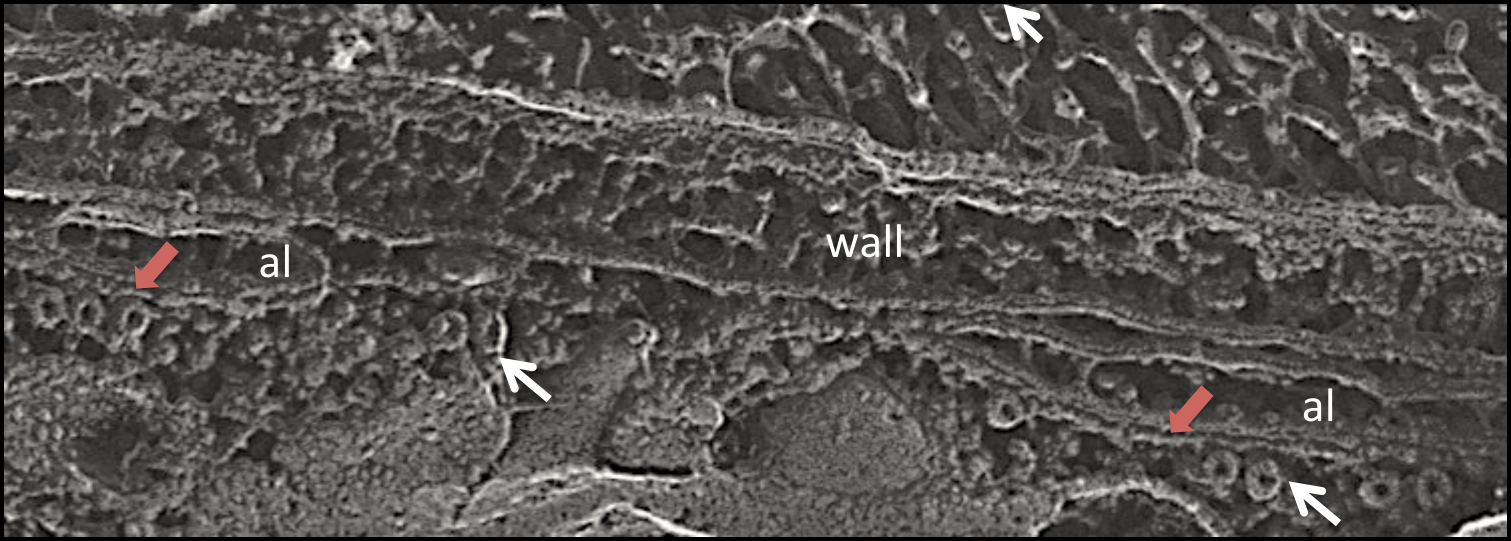

17. Pellicle ultrastructure in *Symbiodinium* sp

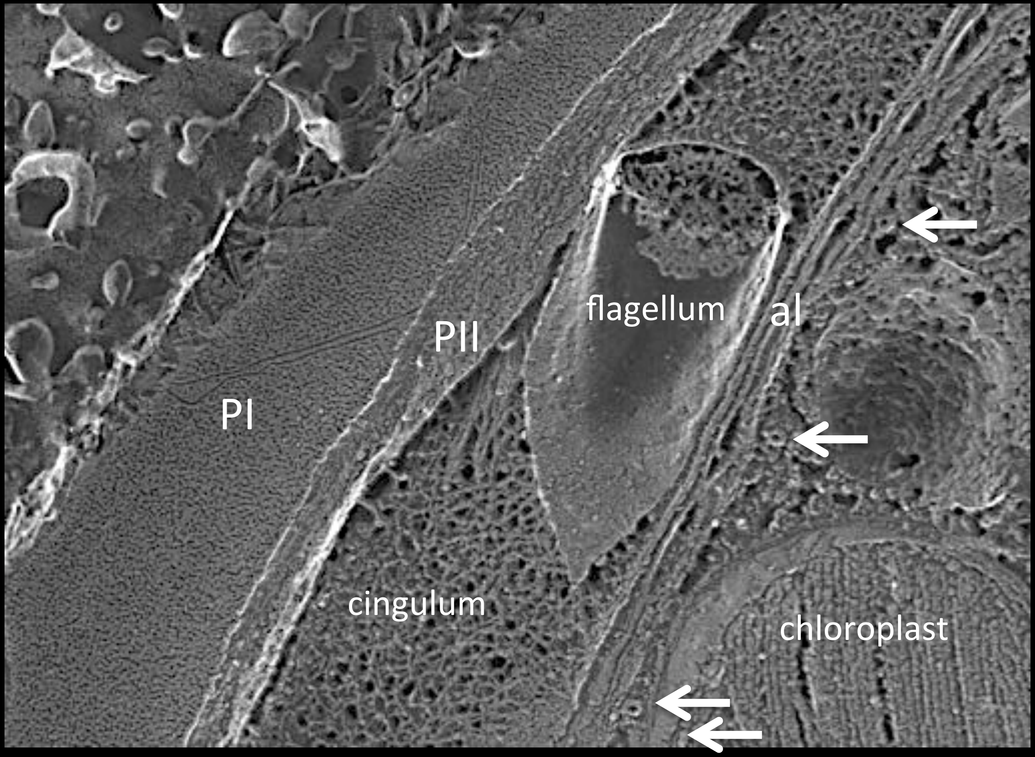

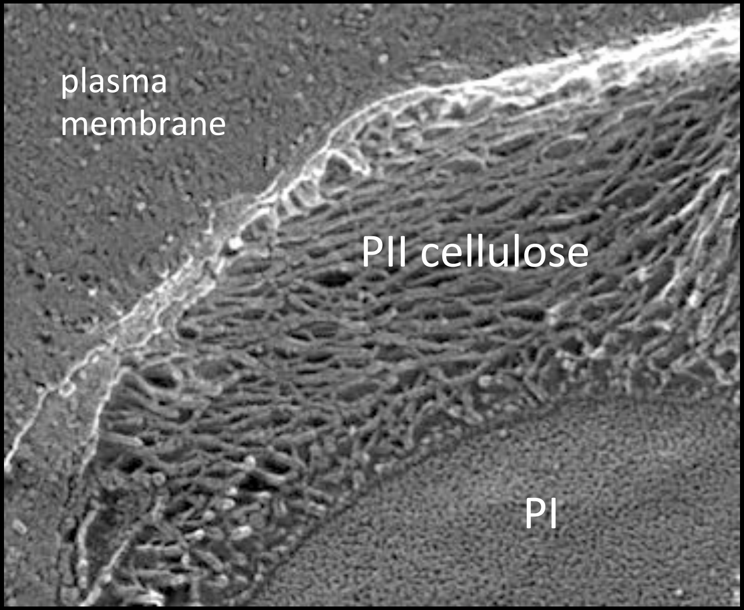

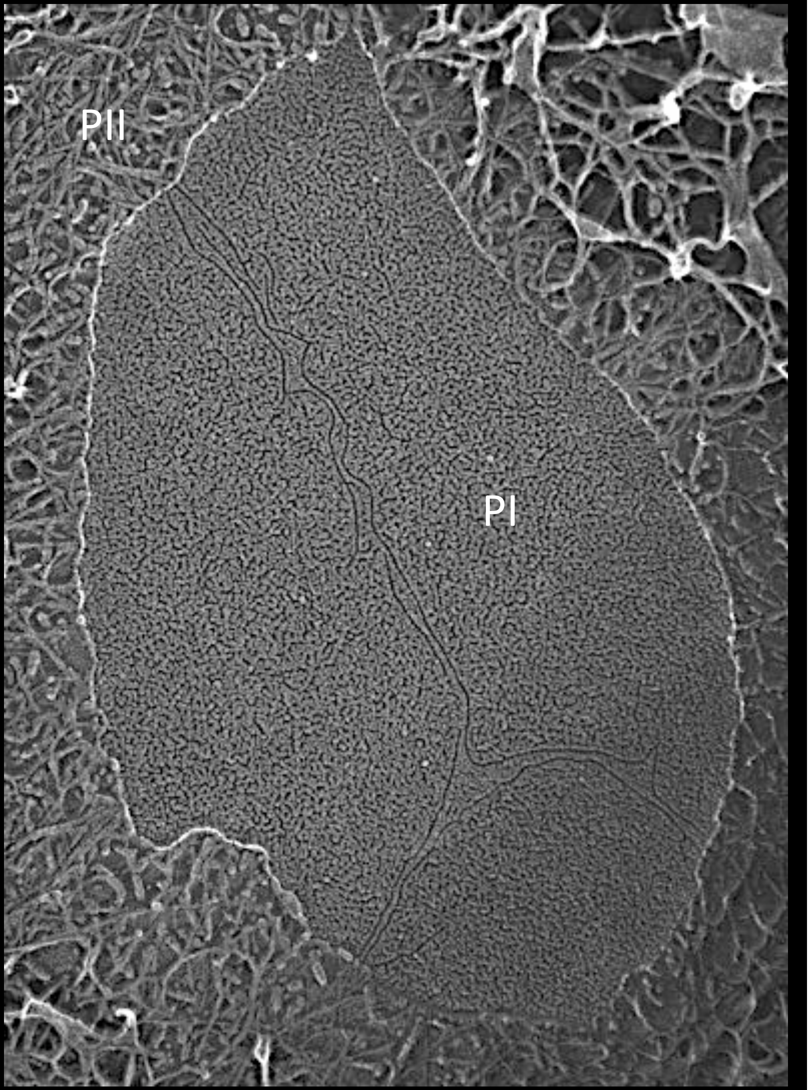

18. *Tetrahymena* ghosts

Abbreviations:

arrows, epiplast
pm, fragments of plasma membrane
mt, microtubule

19. *Tetrahymena* ghosts

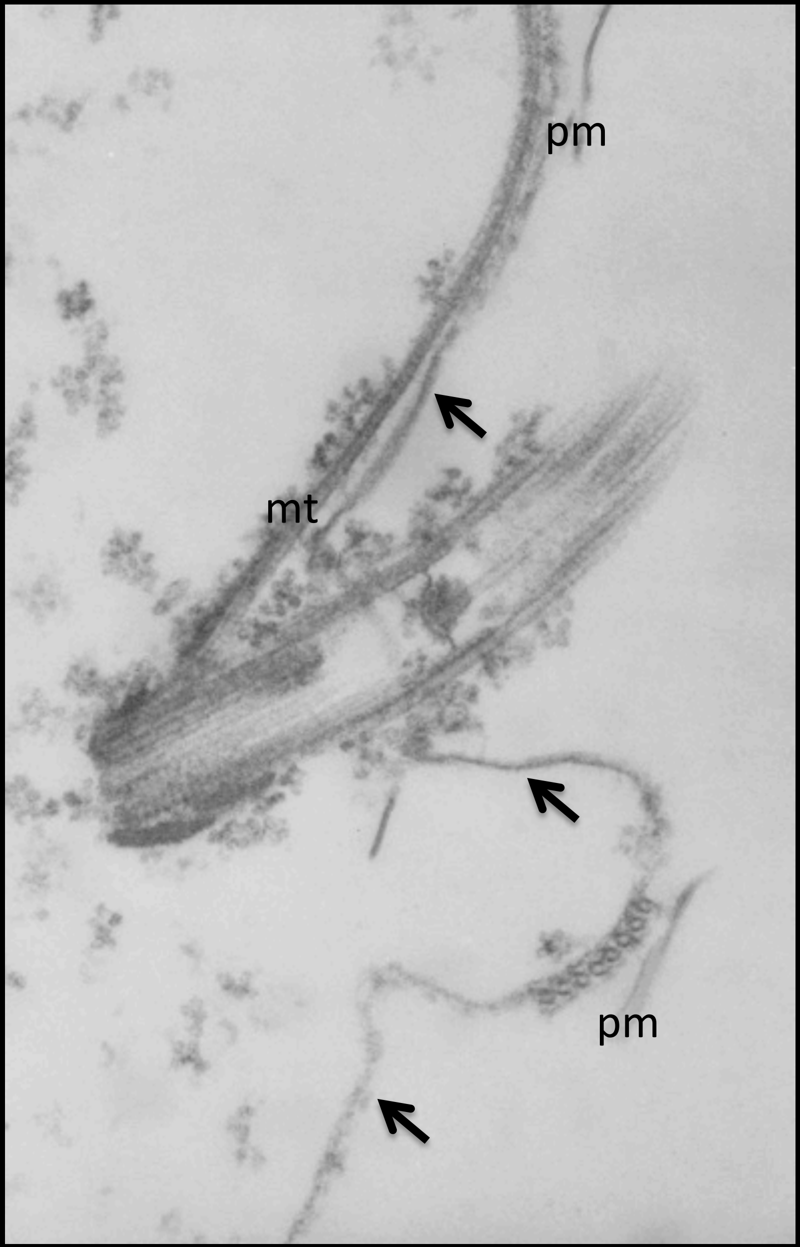

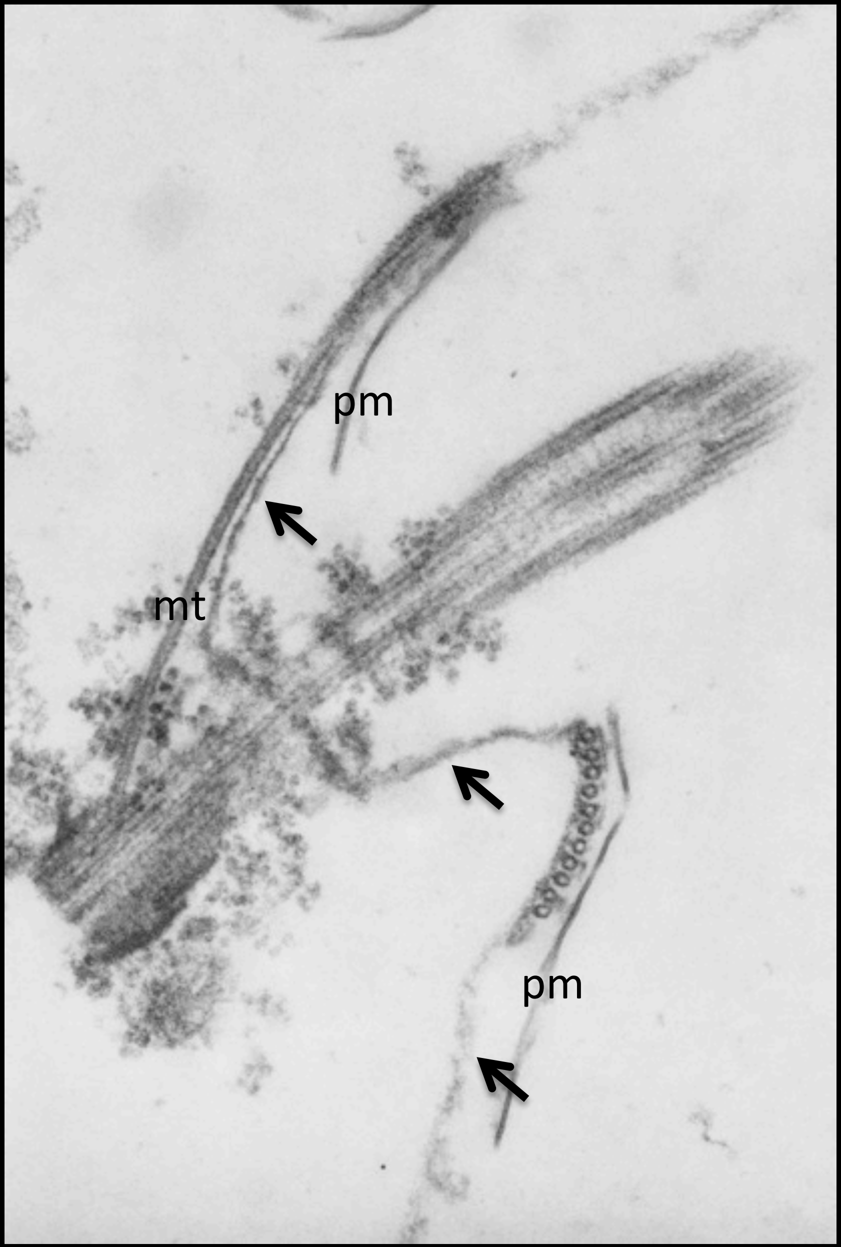

20. *Guillardia theta*

Abbreviations:

pmP, P fracture face of plasma membrane
pmS, etched surface of plasma membrane

21. *Guillardia* cell surface.

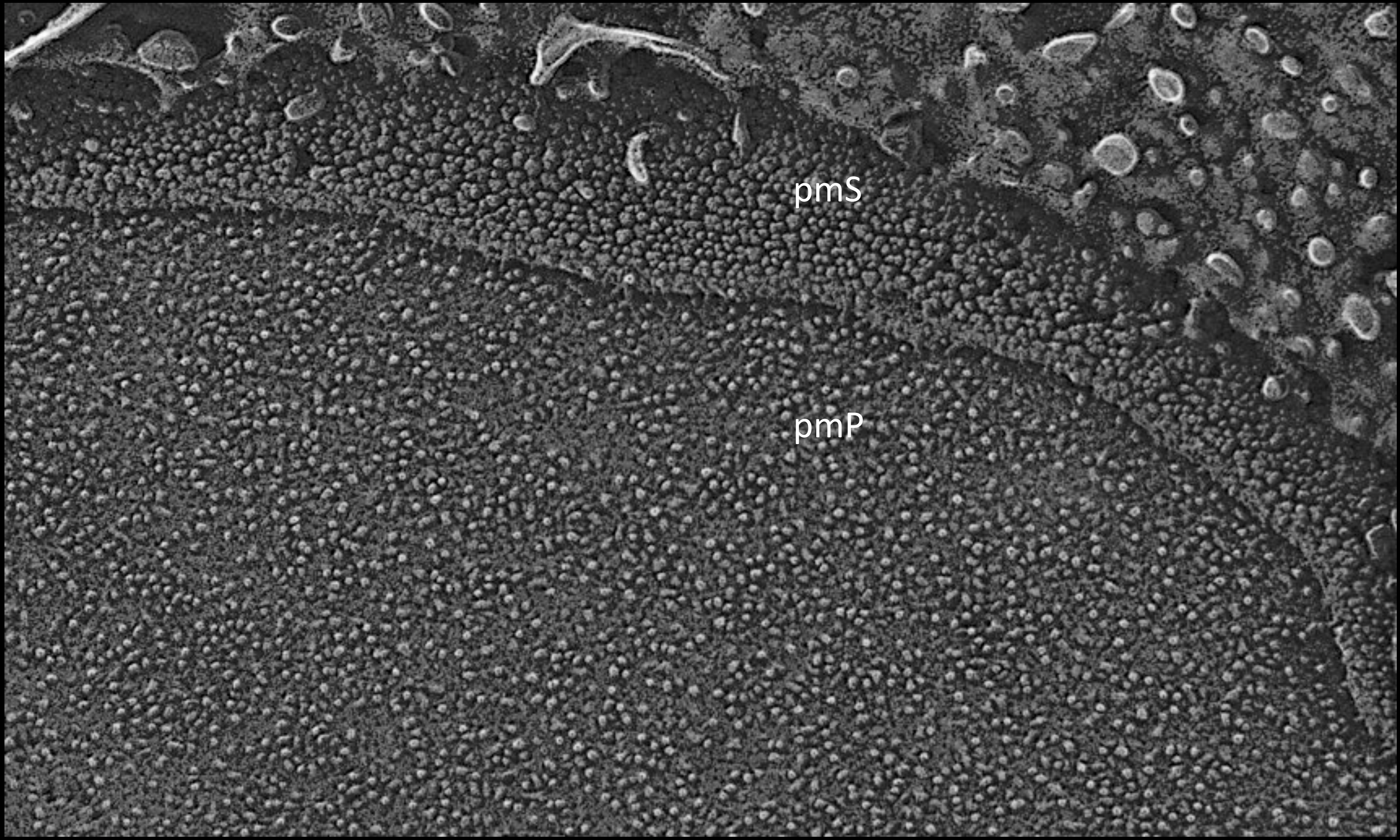

22. *Chroomonas mesostigmatica*

Abbreviations:

L, lip domain of plasma membrane
cp, chloroplast
cpe, chloroplast envelope
f, flagellum
lb, lipid body
arrow, membranes delimiting periplastidial compartment
E, ejectosome at lip boundary
asterisks, IMP-free bands in plasma membrane fracture face
fsl, fibrillar surface layer (aka surface periplast component)
ep, epiplast plate

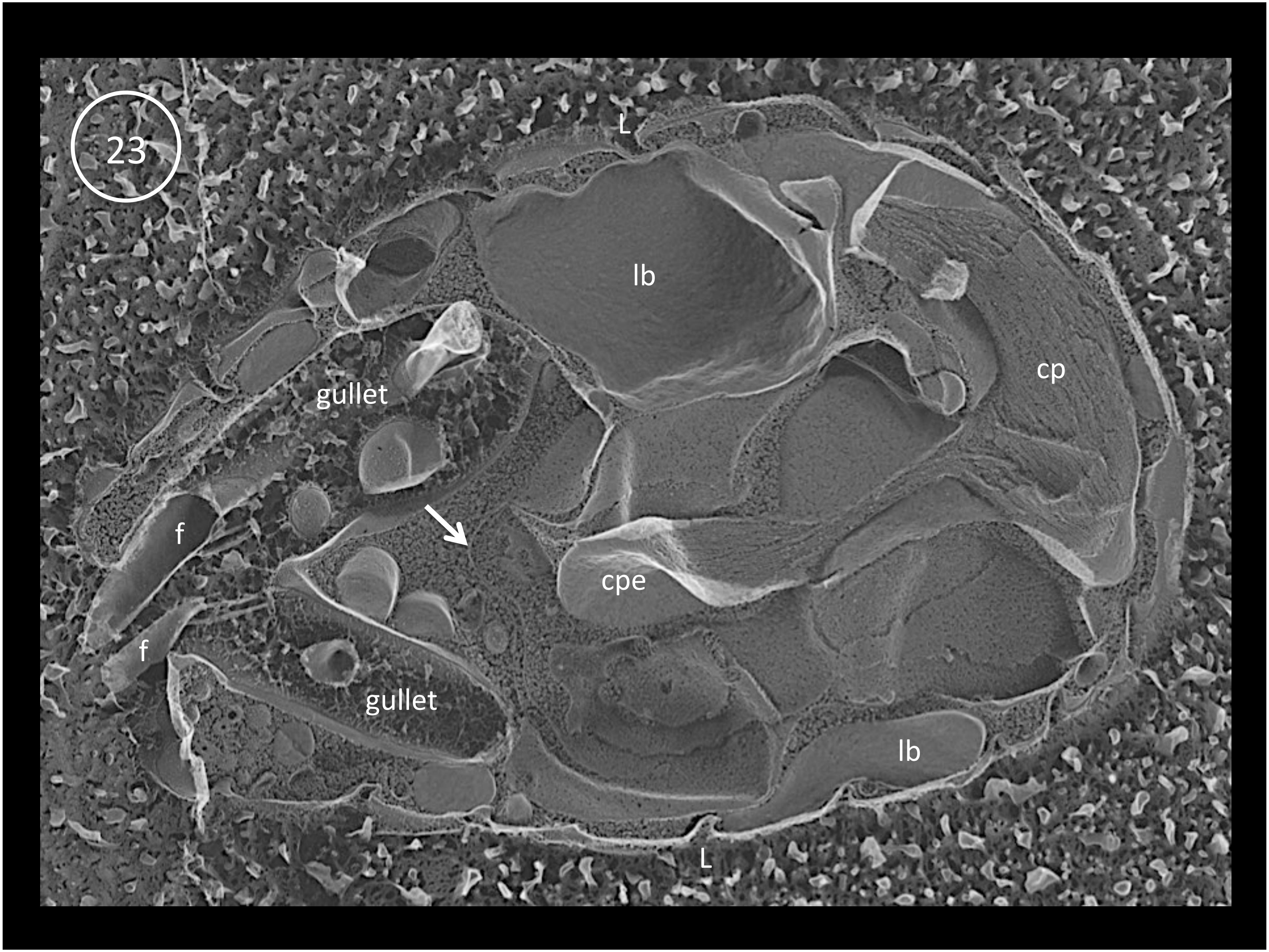
23
L
lb
cp
gullet
f
cpe
f
gullet
lb
L

24

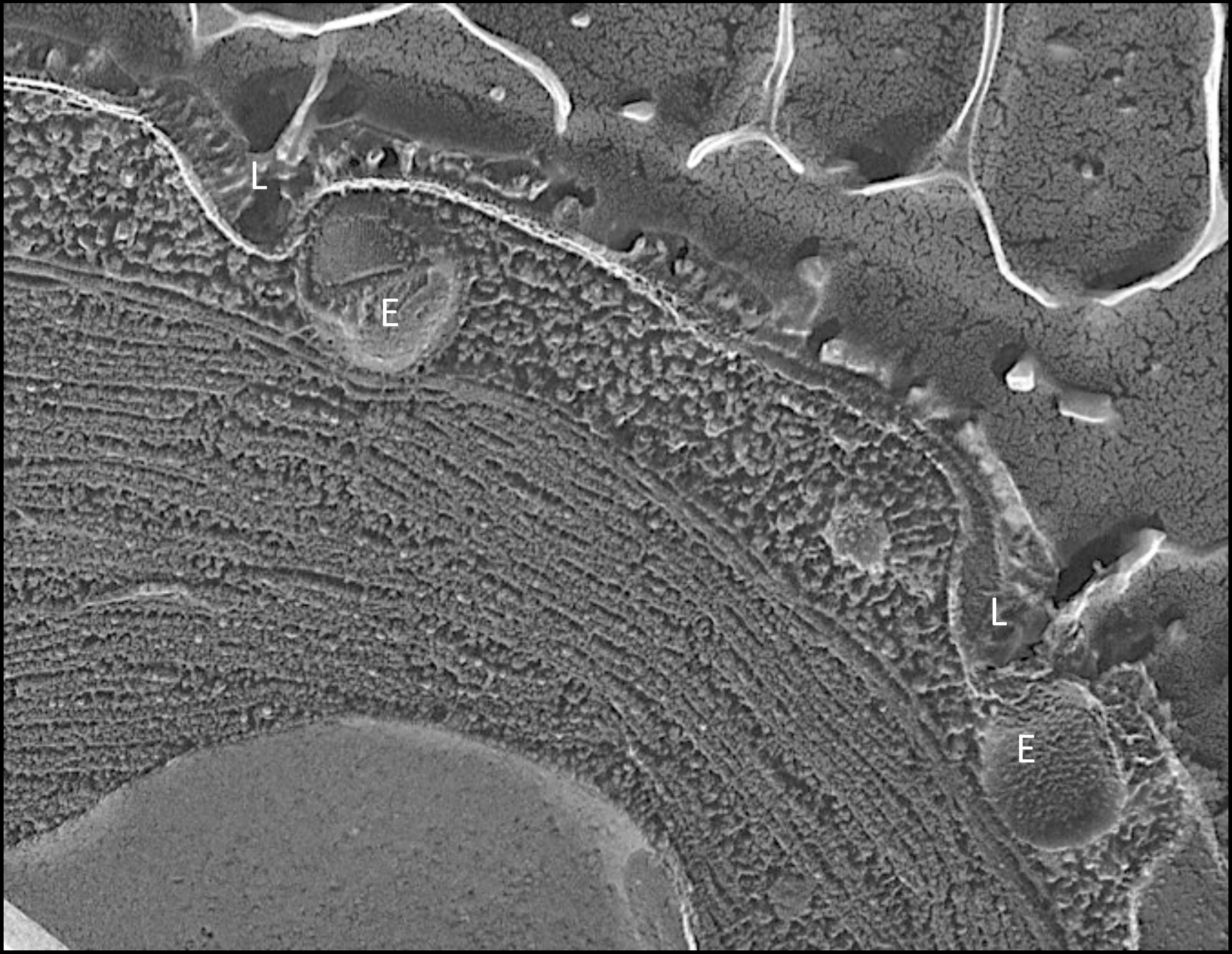

25. Fibrillar surface layer (fsl) (aka surface periplast component) extends into the lip cavities
Asterisks, IMP-free bands in plasma membrane fracture face are vulnerable to etching

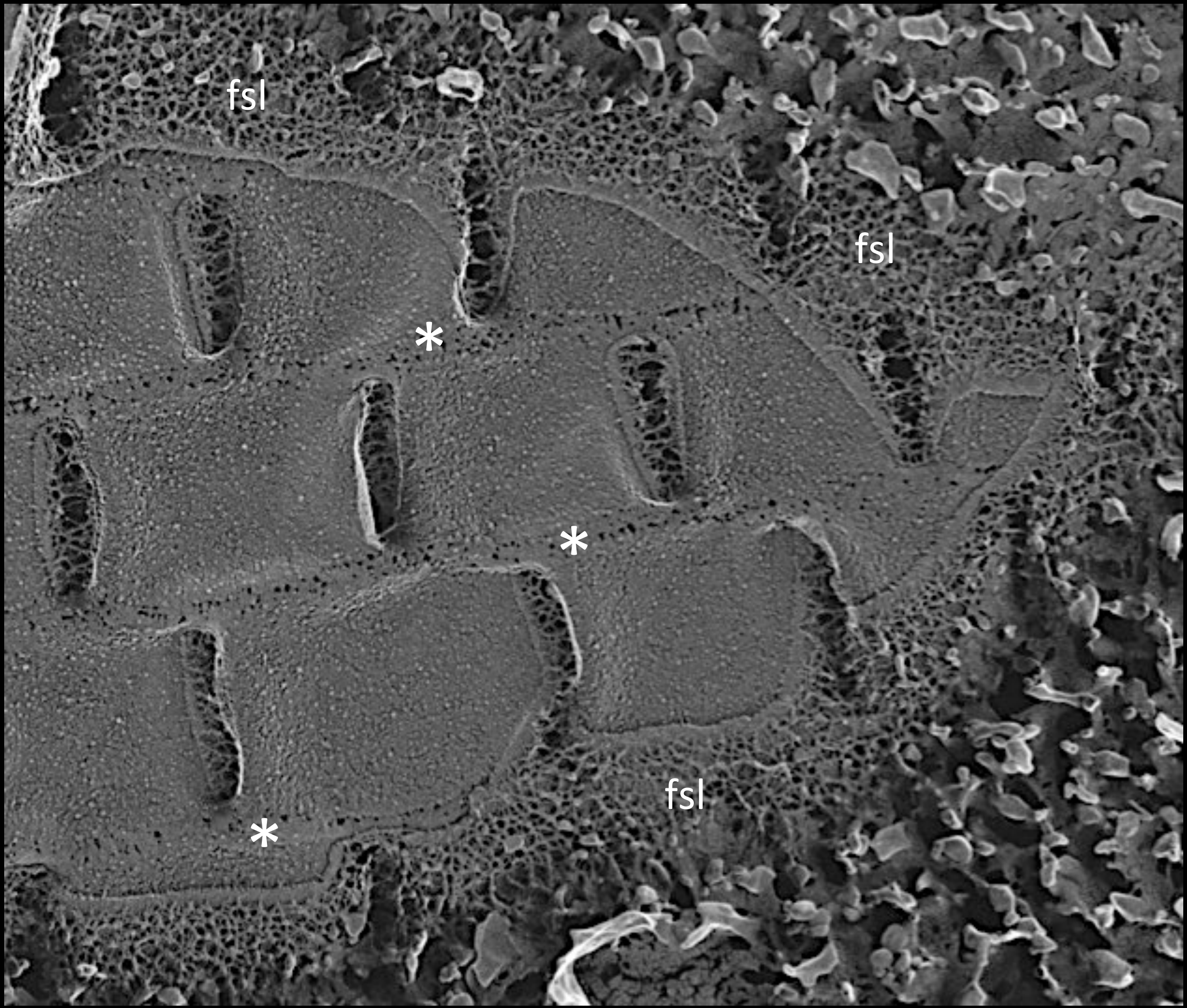

26

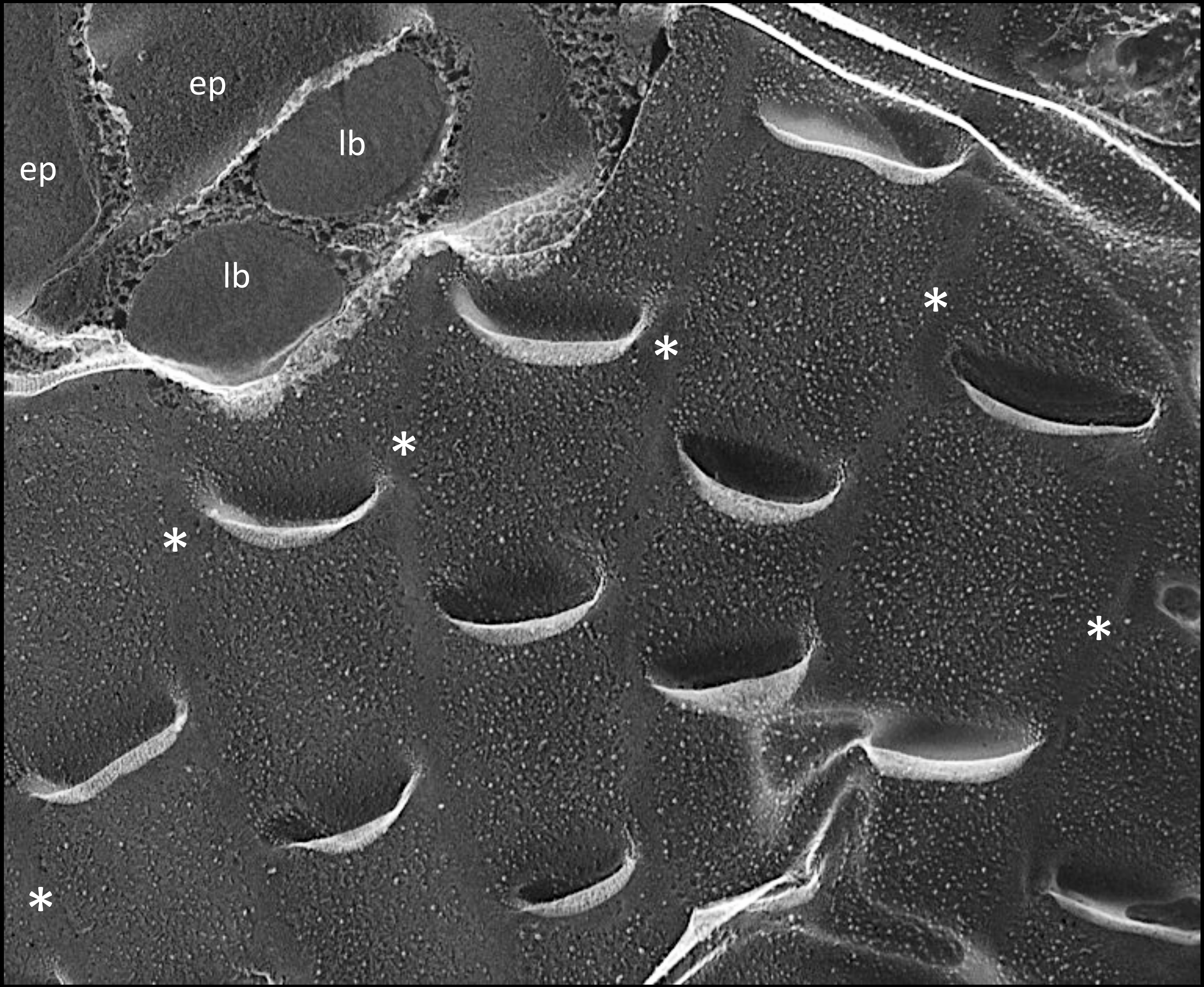

27. Graded spacing of plasma-membrane lips (L) towards anterior end of cell (right)

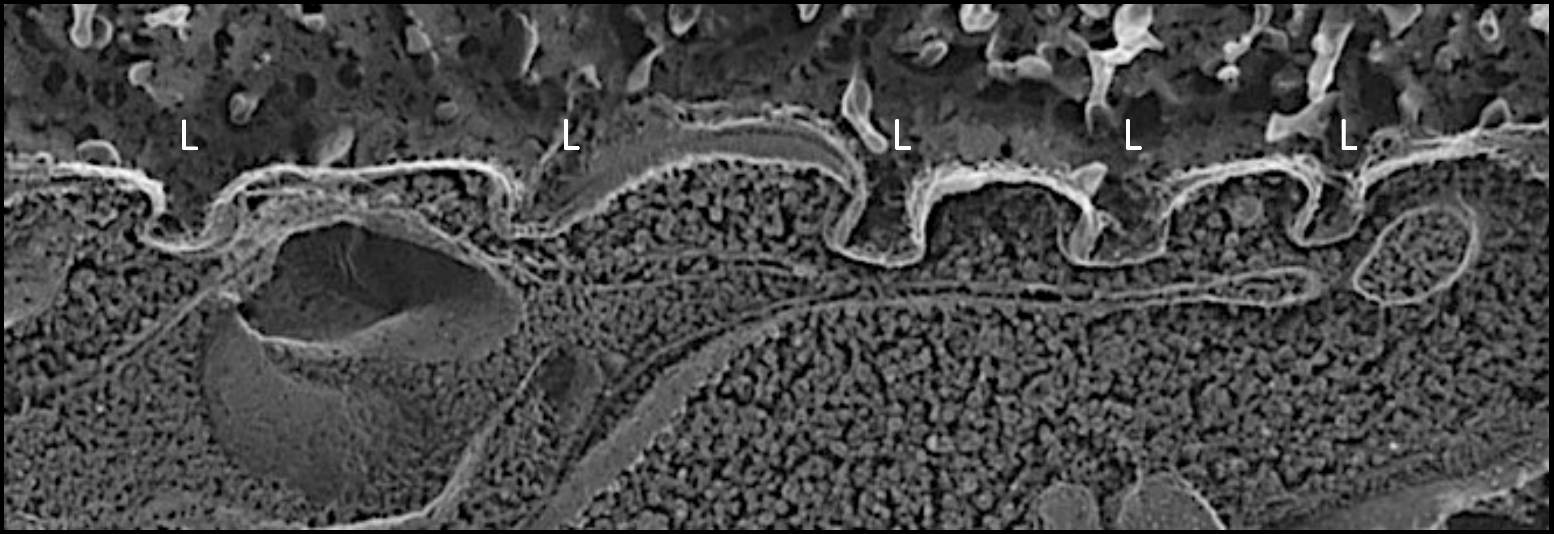

28. Relevant Cryptomonad epiplast images from previous publications

29. Epiplast plates released from sonicated *Chroomonas ovata* cells

Faust MA. 1974. J Phycol. 10: 121-124.

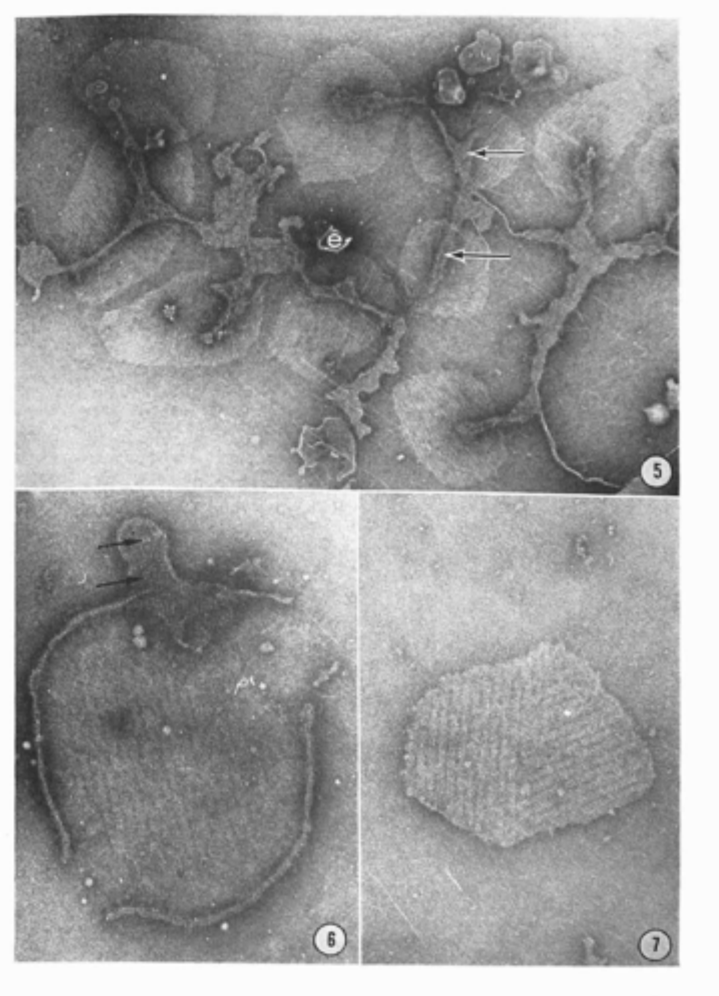

FIG. 5–7. Prolonged sonication 6–8 sec separated the polygonal plates as shown in this negatively (PTA) stained preparation. FIG. 5. The plates are of various shapes and sizes with a definite lattice pattern. Note the attachment of the outer periplast layer to the plates has a fine granular appearance (*arrows*) and separate ejectosome chambers (*e*). × 35,000. FIG. 6. In this negatively (PTA) stained preparation a single plate is shown. The outer periplast layer is partially torn away, yet in some places still held firmly to the edges of the polygonal plate. Note the fine particulate appearance (*arrows*) of the outer periplast layer and the striations on the polygonal plate. × 96,000. FIG. 7. A single striated polygonal plate is visible. The striated lattice pattern appears about 20 nm in size and 1 set of lattices underlies another set at certain angle. × 96,000.

30. Cryptophyte ghosts

Hoef-Emden K., Melkonian M. 2003. Protist 154: 371-409.

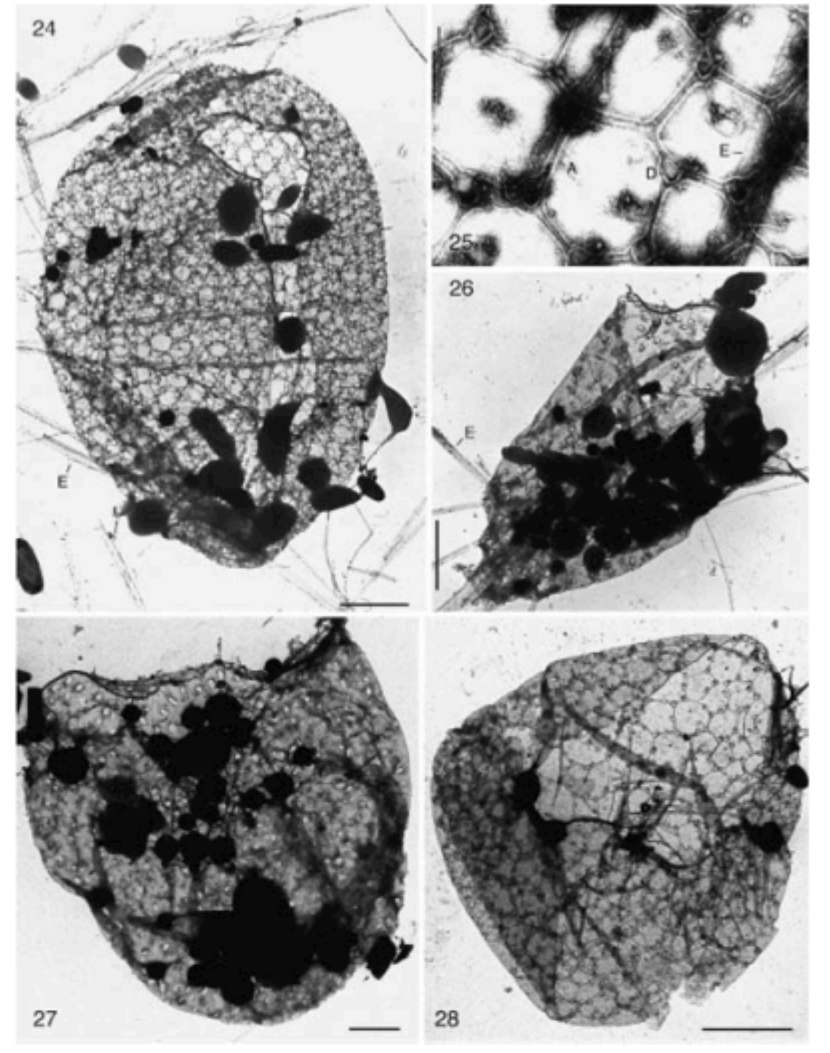

Figures 24–28. Electron micrographs of isolated IPCs (whole mount, uranylacetate). The cultures were not axenic, thus bacteria are scattered around, but also starch grains and discharged ejectosomes. **24.** Complete cryptomorph IPC of strain M0742. The IPC is made up of distinct polygonal plates and kept the approximate shape of the former cell (opening for vestibulum and furrow is clearly visible; apex points to the top of the picture). Scale bar – 2 µm. **25.** Detail of an isolated cryptomorph IPC of strain CCAP 979/61. The IPC consists of distinct polygonal plates with fortified margins. Ejectosome discharge holes between the plates. Negative contrasted stain. Scale bar – 0.5 µm. **26.** Complete campylomorph IPC of strain CCMP 152. The IPC consists of a continuous sheet interrupted by ejectosome discharge holes. The apex of the cell points to the top right corner of the micrograph. The opening for vestibulum and furrow is visible but lost its shape. Scale bar – 2 µm. **27, 28.** Complete IPCs of the dimorphic strain M1077. **27.** Campylomorph IPC interrupted by ejectosome discharge holes (apical part with subapical furrow and vestibulum opening towards the top of the picture). Scale bar – 1 µm. **28.** Cryptomorph IPC. Between the plates are additional ejectosome discharge holes visible (subapical opening for vestibulum and furrow to the top right corner of the picture). In the bottom right corner, a disruption of the periplast is obvious. A, fortified margins of the polygonal plates; D, ejectosome discharge hole; E, discharged ejectosome. Scale bar – 3 µm.

31. Epiplast plates released from sonicated *Chroomonas* sp. cells

Gantt E. 1971. J. Phycol. 7: 177-184.

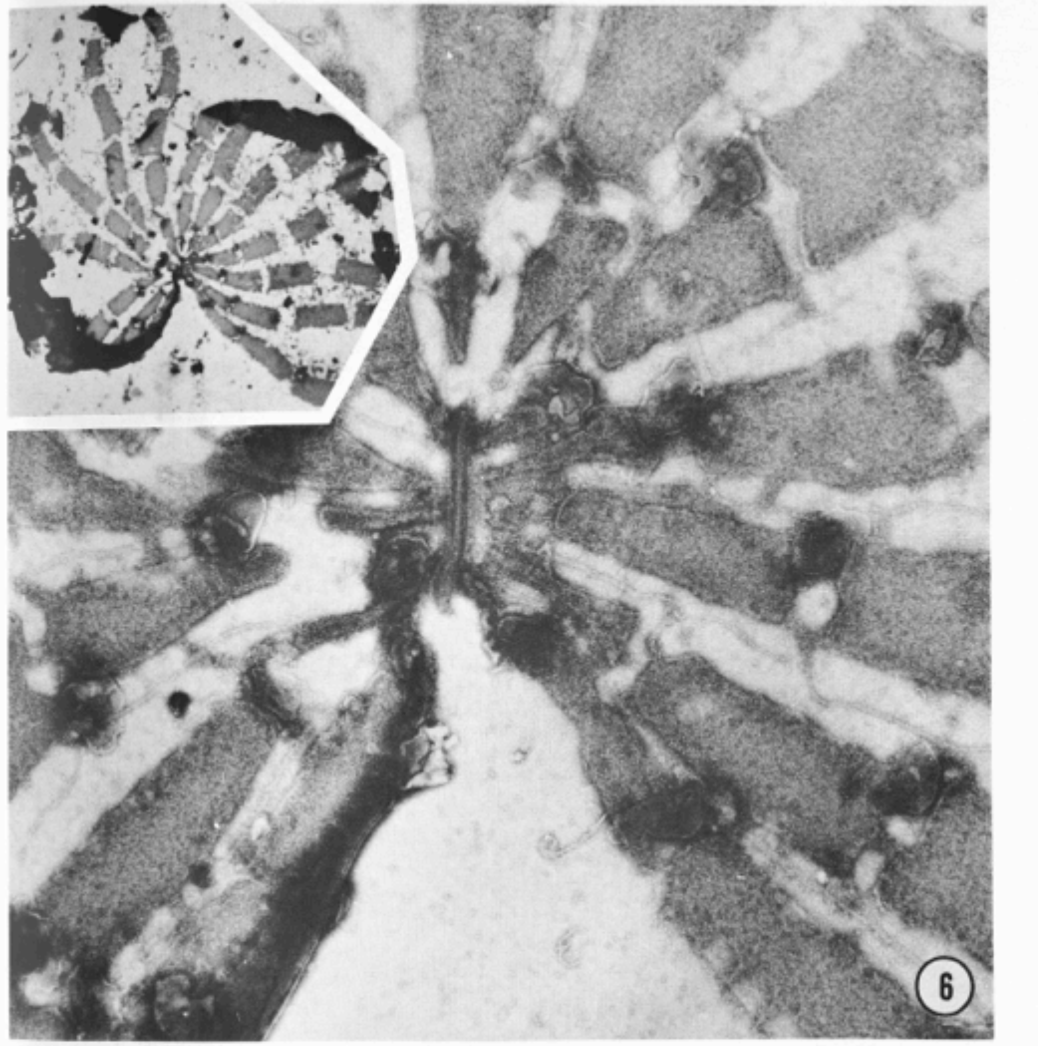

FIG. 6. In this negatively stained preparation (PTA) of the periplast rows of plate areas are separated. The more darkly staining [illegible] structures are empty ejectosome chambers. At least 13 (probably 15 when intact) longitudinal segmented rows are con-[illegible] to the rod-shaped central structure. Note the folding over at the lower left. Faint cords can be seen between the rows. Each [illegible] area is covered by numerous small particles, which at this stage of preparation are irregularly spaced. The inset is an overall [illegible] of the same periplast. ×50,000. Inset ×5600.
