# Supplementary material for: Epiplasts: Membrane Skeletons and Epiplastin Proteins in Euglenids, Glaucophytes, Cryptophytes, Ciliates, Dinoflagellates, and Apicomplexans
Source: mBio. 2018 Oct 30;9(5):e02020-18. doi: 10.1128/mBio.02020-18 (PMC6212826; doi:10.1128/mBio.02020-18)
Supplement: TEXT S10 [file mbo005184120s10.pdf]

1

Key to Gene Annotations

- **General organization:** The medial acid-base dyads (ABDs) are "parsed" into "strings that initiate with a dyad. N-term domain is denoted the head, C-term the tail.
- **Highlights**: Yellow, tyrosine; Green, cysteine; Pink, VPV; Gray, ABD in predicted head and tail domains.
- **Colored font:** Red, glycine residues in ABD domains; Bold-faced red, last G residue in head and first G residue in tail; Blue, alanine residues in ABD domains; Green, repeated string domains.
- **Underscores:** Acid-base triads or tetrads.
- **Commentary**: Notes on distinctive gene features (e.g. orthologues, localization patterns) in green font at top of some pages. Predicted homology domains (e.g. PDZ, coiled-coil) are given in Supplement Table 2.
- **Secondary structure**: PSIPRED predictions for a subset of proteins. Yellow, amino acids predicted in β-strand; pink, amino acids predicted in α-helix; no highlight, amino acids predicted in disordered (random coil) domain.

2

Naegleria gruberi jgi|Naegr1|70383|

Secondary structure prediction next slide

MSSIDDDNIFIEQEEPTEEDVLKYAEFLGIDPVLEKELIPIALEGIKAPLPDGWRPCQSDGQL
YYFNFNSGESIWDHPLDAYYKEKVAKEREKLKKEKKKKKKITTTKLDPLNSLRSSDEIPSLT
NMTTLSSSSSVKKLRESSSSSPQNNEELIKLQDKIRSLDQQLELSNRERIELQKKIIHLNNEN
EKLENDLRENKNKMEVEYRNKITQLQTEKDIMEKKHKQTLQDTFIENRKQIALARQEADE
MFEENARLKKNQAQTEAKTFKAIECQT

DKIDTKVLV
EKIV
EKIV
EKPV
EKIV
EKIV
EKVV
EKPV
EKII
EKIV
EKPV
EKIV
EKVV
EKIV
EKPKIV
EKIVEV
ERKK

TSLSKTTQTEIQTISSLCQTDGVKTTSDGVNTITKTFKDMLAQTSEPRQITPKKVVTIDQSQQ
TTEKTTRDECLQADIQLPANKHVEIETITLSPIPVQQGPKSPVLATPLKPILVKTKKPKQSNKY
ESPSPLSISDLDKENTPPRQLAKQNTEFLLYKEKTEKKISEEKLKLKKAKEFCSQEKETIRARQE
MLEQAREEWKTDMLNTSIIKDGSQHNMNKILKSVKQHLEKQAHSLNNDVKRVNEIQNLIA
LRKKKIKVLEDSIIDQSHFESYISADPDSPNSLSTSSSSDSDTNKSHLAKILNRIEEDLALLHKKIE
LQNKPPVDENYKAYKKIFPELDQTNLDETSQSYQMISTKWHNYFKQTEKRRKILQGKINAFQ
NQLDKWISERDHKRDMFLKHGNWLLSLKHELT

3

Naegleria gruberi jgi|Naegr1|70383|

Secondary structure prediction

```
  1 M S S I D D D N I F I E Q E E P T E E D V L K Y A E F L G I D P V L E K E L I P I A L E G I K A P L
 51 P D G W R P C Q S D G Q L Y Y F N F N S G E S I W D H P L D A Y Y K E K V A K E R E K L K K E K K K
101 K K K K I T T T K L D P L N S L R S S D E I P S L T N M T T L S S S S S V K K L R E S S S S S P Q N
151 N E E L I K L Q D K I R S L D Q Q L E L S N R E R I E L Q K K I I H L N N E N E K L E N D L R E N K
201 N K M E V E Y R N K I T Q L Q T E K D I M E K K H K Q T L Q D T F I E N R K Q I A L A R Q E A D E M
251 F E E N A R L K K N Q A Q T E A K T F K A I E C Q T D K I D T K V L V E K I V E K I V E K P V E K I
301 V E K I V E K V V E K P V E K I I E K I V E K P V E K I V E K V V E K I V E K P K I V E K I V E V E
351 R K K T S L S K T T Q T E I Q T I S S L C Q T D G V K T T S D G V N T I T K T F K D M L A Q T S E P
401 R Q I T P K K V V T I D Q S Q Q T T E K T T R D E C L Q A D I Q L P A N K H V E I E T I T L S P I P
451 V Q Q G P K S P V L A T P L K P I L V K T K K P K Q S N K Y E S P S P L S I S D L D K E N T P P R Q
501 L A K Q N T E F L L Y K E K T E K K I S E E K L K L K K A K E F C S Q E K E T I R A R Q E M L E Q A
551 R E E W K T D M L N T S I I K D G S Q H N M N K I L K S V K Q H L E K Q A H S L N N D V K R V N E I
601 Q N L I A L R K K K I K V L E D S I I D Q S H F E S Y I S A D P D S P N S L S T S S S S D S D T N K
651 S H L A K I L N R I E E D L A L L H K K I E L Q N K P P V D E N Y K A Y K K I F P E L D Q T N L D E
701 T S Q S Y Q M I S T K W H N Y F K Q T E K R R K I L Q G K I N A F Q N Q L D K W I S E R D H K R D M
751 F L K H G N W L L S L K H E L T
```

4

Naegleria gruberii >jgi|Naegr1|58849|

MTKKKEKKVEEKKVVVEEKKPEPEKPKKSLTPSFFSRKKKDDKKQDAQTPST
PGSSQIFEQQPTTPRDATSSEQTPVVLSSDNHLLEDTTTQASTTLQSDEVSTT
SGTTTTDDSMLNSTSDVPVITNNEPISSLELINTTTTVMTEQYQENNLGDSVQS
IDTTLTSSQINDSMLASVTSVESSPATPVD

EKKEEETPVQQETQPVVVETIVE
EKKEEIVVVETTPEVVVD
EKKEESVVTETTPTSPVVVE
EKKEVEVVPESVVS
EKKEETQETPIVE
DKKEVVIETPVVE
DKKEETPVVVEETQPVVETVV
EKKEEETPIESTPAVTESPVETIIVEETQPVVVE
EKKEEIVSENVETVVAETTPVQQEVTEVSE
EKKEETPVVVEESQPVIE
EKKEESV
KEDHEAIL
EKALESTSIESIPEPVVEQPTPVTETVVAEPTPVPVVTETTESSDDQ
DKKILDQVTSEFFA
EKSELRQIVH
DKAETVDLYHQY
REQD
EKEKKKLL
EKQEQTK

AASTSTSTTTSTTTTEKPSSTKTTKTTTKTTIEESSSALNWVIGIGAVAASAVIGIF
AAKKFKLF

1 M T K K K E K K V E E K K V V V E E K K P E P E K P K K S L T P S F F S R K K K D D K K Q D A Q T P
51 S T P G S S Q I F E Q Q P T T P R D A T S S E Q T P V V L S S D N H L L E D T T T Q A S T T L Q S D
101 E V S T T S G T T T T D D S M L N S T S D V P V I T N N E P I S S L E L I N T T T V M T E Q Y Q E N
151 N L G D S V Q S I D T T L T S S Q I N D S M L A S V T S V E S S P A T P V D E K K E E E T P V Q Q E
201 T Q P V V V E T I V E E K K E E I V V V E T T P E V V V D E K K E E S V V T E T T P T S P V V V E E
251 K K E V E V V P E S V V S E K K E E T Q E T P I V E D K K E V V I E T P V V E D K K E E T P V V V E
301 E T Q P V V E T V V E K K E E E T P I E S T P A V T E S P V E T I I V E E T Q P V V V E E K K E E I
351 V S E N V E T V V A E T T P V Q Q E V T E V S E E K K E E T P V V V E E S Q P V I E E K K E E S V K
401 E D H E A I L E K A L E S T S I E S I P E P V V E Q P T P V T E T V V A E P T P V P V V T E T T E S
451 S D D Q D K K I L D Q V T S E F F A E K S E L R Q I V H D K A E T V D L Y H Q Y R E Q D E K E K K K
501 L L E K Q E Q T K A A S T S T S T T T S T T T T E K P S S T K T T K T T T K T T I E E S S S A L N W
551 V I G I G A V A A S A V I G I F A A K K F K L F

Percolomonas >CAMPEP_0117428174

MKTALAAQTNAHFFTIGEITDFLIMLGYGIFPFAIHWLMYEIMFTCFKKSDGA
VVFHPLIDYTAGKLIVLGGLGIVTAICDAIENFLMLLLIQPDYERNGNVSTAAV
IVYSLFALFKWIGLCINILFIFIYSIIFYAKILAFIVWKSSSKGDDDYLDPLKIETA
IEPQVIIDTLPS

EKEEHVQEQELEVV
KEDSEVAEEVAEEVAEEVAEEV
KEEV
KEEM
KEELMEEAVEEIVEEVVEEGV
KEIMEEE

GELQEEEKEVVEEAVQEAVEEVVEEAIEEAVEEAVEEVIEEAVEEVVEEAVE
EAVEEVIEEEQQEQQEEEQQQEEEVAEEVAEE

1 M K T A L A A Q T N A H F F T I G E I T D F L I M L G Y G I F P F A I H W L M Y E I M F T C F K K S [illegible]
51 D G A V V F H P L I D Y T A G K L I V L G G G I V T A I C D A I E N F L M L L L I Q P D Y E R N G N [illegible]
101 V S T A A V I V Y S L F A L F K W I G L C I N I L F I F I Y S I I F Y A K I L A F I V W K S S S K G [illegible]
151 D D Y L D P L K I E T A I E P Q V I I D T L P S E K E E H V Q E Q E L E V V K E D S E V A E E V A E [illegible]
201 E V A E E V A E E V K E E V K E E M K E E L M E E A V E E I V E E V V E E G V K E I M E E E G E L Q [illegible]
251 E E E K E V V E E A V Q E A V E E V V E E A I E E A V E E A V E E V I E E A V E E V V E E A V E E A [illegible]
301 V E E V I E E E Q Q E Q Q E E E Q Q Q E E E V A E E V A E E

6

Caulobacter crescentus NP_419492.1

Articulin. VYV modules

MISFKTLCRLGAATLAALSLSAAPVLADGFPGAPTRSPVPKQATPKPRTKIVYV

DRPVVV
EKRVPVYI
DRPTTQIV
EKVV
EKPVII
ERPVVV
EKRIEVPV
EKLVEVPV
EKKVYV
DRPV
EKKVYV
DRTV
EKKVYVDRPV
EKKVYVDRIV
EKVV
EKRVEVPVQKIV
EKVV
EKPVYV
DRPVPS
DKPAEVV
EKIIQVPIYVYV
DRPVATPAP

GCAQPCGYVERPAPCVGPCGYPQPAPCASRCDIHWRPAPPPSCGHPCAPIPAPT
PCASPCGYGGVIEHREERSSYDERHDGYAELPPLPMGYVGSASGGRGGGYGGV
GLIGGGGWRTGGYAGASASARASATASATASTSIRIGGGGGGCSGCGGKRH

1 M I S F K T L C R L G A A T L A A L S L S A A P V L A D G F P G A P T R S P V P K Q A T P K P R T K
51 I V Y V D R P V V V E K R V P V Y I D R P T T Q I V E K V V E K P V I I E R P V V V E K R I E V P V
101 E K L V E V P V E K K V Y V D R P V E K K V Y V D R T V E K K V Y V D R P V E K K V Y V D R I V E K
151 V V E K R V E V P V Q K I V E K V V E K P V Y V D R P V P S D K P A E V V E K I I Q V P I Y V Y V D
201 R P V A T P A P G C A Q P C G Y V E R P A P C V G P C G Y P Q P A P C A S R C D I H W R P A P P P S
251 C G H P C A P I P A P T P C A S P C G Y G G V I E H R E E R S Y D E R H D G Y A E L P P L P M G Y V
301 G S A S G G R G G G Y G G V G L I G G G G W R T G G Y A G A S A S A R A S A T A S A T A S T S I R I
351 G G G G G G C S G C G G K R H

7

Caulobacter vibrioides PLR11764.1

Articulin VYV modules Orthologue of NP_419492.1

MISFKTLCRLGAATLAALSLSAAPVLADGFPGAPTRSPVPKQATPKP
RTKIVYV

DRPVVV
EKRVPVYI
DRPTTQIV
EKVV
EKPVII
ERPVVV
EKRIEVPV
EKLVEVPV
EKKVYV
DRPV
EKKVYV
DRTV
EKKVYV
DRPV
EKKIYV
DRVV
EKVV
EKRVEVPVQKIV
EKVV
EKPVYV
DRPVPS
DKPAEVV
EKIIQVPIYVYV
DRPV

ATPAPGCAQPCGYVERPAPCVGPCGYPQPAPCASRCDIHWRPAPPP
SCGHPCGPIPAPTPCASPCGYGGVIEHREERSSYDERHDGYAELPPL
PMGYVGSASGGRGGGYGGVGLIGGGGWRTGGYAGASASARASAT
ASATASTSIRIGGGGGGCSGCGGKRH

8

Caulobacter vibrioides ATC34941.1

Articulin
VYV modules Orthologue to PLR11764.1

MISLKTLCRLGAATLAALSLSAAPALADGFPGAPARSPVPKQATPKP
RTKIVYV

DRPVVV
EKRVPVYI
DRPTTQIV
EKVV
EKPVII
ERPVVV
EKRIEVPV
EKLVQVPV
EKTVYV
DRPV
EKLVYV
DRPV
EKKVYV
DRVV
EKRVEVPV
EKIV
EKVV
EKPVYV
DRPVPT
DKPGEVV
EKIIQVP
IYVYV
DRPVP

TPSQGCAQPCGYVERPAPCANPCGSVAPAPCASRCDIHWRPAPPPS
CSHPCGPIQVPPPCASPCGYGGVIEHREERSSYDERHDGYAELPPLP
MGYVGSASGGRGGGYGGVGLIGGGGWRSGAYGGASASARASATA
SATATTSIRIGGGGGGCSGCGGKRH

9

Caulobacter sp. KRA75086.1.

Articulin
VYV modules. Orthologue to KRA59406.1

MVRTRVKIV

ERPVYV
EKIVKQPVYI
EKRTEVPV
DRPVYI
DRPV
DRIV
EKRVEVPV
ERIV
EKRVEVPVVKIV
EKPV
DRPVYI
DRPV
DRVI
EKRVEVPV
DRPVYV
DRPV
DRVI
EKRVEIPV
DRPVYV
DRPV
DRVV
ERRVEIPV
DRPVYV
DRVI
EKRVEVPV
ERIVRTPVYVEHQ
ERRV
ERR

GCDCEGDYYEQGGAYDRVQGGFERSEESYESESSRYSEEGQGWSSQ
GSYMAGDGYAQSLGGVGGGGYYGYSSARYGAGGGWLGGQSYGRSG
YSGGYSGGYSSGGAGAGAASSARASSSASSSTSVTVSGGGHYGGGG
HGGGHGGGSTGGHGGCCGR

10

Caulobacter sp. KRA59406.1

Articulin
VYV modules. Orthologue to KRA75086.1

MNPKNILLIGAAAITFAALGPVMISTAAADGYPARVVPVTKAKPARKPA
VRVRVV

ERPVYV
EKIVKQPVYIEHRVEVPV
DRPVYV
DRPV
DRIV
EKRVEVPVVKIV
EKPV
DRPVYV
DRPV
DRIV
EKRVEVPV
DRIV
EKRVEVPVVRVV
DRPV
DRPVYI
DRPV
DRVV
ERRVEVPV
DRPVYV
DRPV
DRII
EKRVPVYV
ERQPPVAYRR

GCDCDDDRRVYQDHGRVYGGSAVEQSVESSEESYESYSERYDGDGWS
SQGSYLAGDGYAQSLDGYYYGGTTARYGVGGGWNGGQSYGGQSHGG
QSYGGRSYYGAGGYGRAWSGAGASAASSASSSTSVTVSGGGRYGGGG
HGGGSTGGHGGCCGR

11

Caulobacter sp. WP_099575684.1

Articulin
VYV modules

MSPKTLLRVGVLAVFAGLTLASPVLADGYERAPVRQHHAPKRVTTRTKV
VYV

DRVV
ERRVPVYV
DRPTTQIV
EKIV
EKPVII
ERPVVV
EKRIEVPV
EKLVQVPV
EKIV
DRPVYI
DRPV
EKIV
EKIV
EKRVEVPV
DRPVYV
DRPVPV
DRPVPV
DRPVPTP

APTVTPCQQACSGPGGEVVDRIIQVPVYVYVDRPVDRPVYVPVQQQGGCQ
PRVCGGAVYQQQCSPCGGQRYEDSYYEESRERSSYEYSSDYSGSRYSAG
GGWNAGSVGSSAWGNYIEPPPLPMGYVGAQSGGRGGGYGGPGLAGGGG
YAGGYAGSSSSGYASSSASASASASSSIHIGGGYHGGGSKGGCGACGGGG
KHH

```
  1 M S P K T L L R V G V L A V F A G L T L A S P V L A D G Y E R A P V R Q H H A P K R V T T R T K V V
 51 Y V D R V V E R R V P V Y V D R P T T Q I V E K I V E K P V I I E R P V V V E K R I E V P V E K L V
101 Q V P V E K I V D R P V Y I D R P V E K I V E K I V E K R V E V P V D R P V Y V D R P V P V D R P V
151 P V D R P V P T P A P T V T P C Q Q A C S G P G G E V V D R I I Q V P V Y V Y V D R P V D R P V Y V
201 P V Q Q Q G G C Q P R V C G G A V Y Q Q Q C S P C G G Q R Y E D S Y Y E E S R E R S S Y E Y S S D Y
251 S G S R Y S A G G G W N A G S V G S S A W G N Y I E P P P L P M G Y V G A Q S G G R G G G Y G G P G
301 L A G G G G Y A G G Y A G S S S S G Y A S S S A S A S A S A S S S I H I G G G Y H G G G S K G G C G
351 A C G G G G K H H
```

12

Caulobacter henricii WP_081943177.1

Articulin. VYV modules Predicted secondary structure next slide.

MVRTRVRIV

ERPVYV
EKIVKQPVYI
EKRTEVPV
DRPVYI
DRPV
DRIV
EKRVEVPV
DRIV
EKRVEVPVVKIV
EKPVDRPVYI
DRPVDRVI
EKRVEIPVDRPVYVDRPV
DRVI
EKRVEIPV
DRPVYV
DRPV
DRVV
ERRVEVPVDRPVYVDRVV
EKRVEVPV
ERIVRTPVYV
ERQ
ERRV
ERR

GCDCDGDRGYYEQGRVYDRAEGSVERSEESYESESSRYSEEGQGWSS
QGSYMAGDGYAQSLGGVGGGGYYGYSSARYGAGGGWLGGQTYGRSG
YSGGYSSGWAGAGAASSARASSSASSSTSVTVSGGGHYGGGGHGGGY
GGGHGGGHGGGSTGGHGGCCGR

13

Caulobacter henricii WP_081943177.1

Predicted secondary structure.

```
1   M V R T R V R I V E R P V Y V E K I V K Q P V Y I E K R T E V P V D R P V Y I D R P V D R I V E K R
51  V E V P V D R I V E K R V E V P V V K I V E K P V D R P V Y I D R P V D R V I E K R V E I P V D R P
101 V Y V D R P V D R V I E K R V E I P V D R P V Y V D R P V D R V V E R R V E V P V D R P V Y V D R V
151 V E K R V E V P V E R I V R T P V Y V E R Q E R R V E R R G C D C D G D R G Y Y E Q G R V Y D R A E
201 G S V E R S E E S Y E S E S S R Y S E E G Q G W S S Q G S Y M A G D G Y A Q S L G G V G G G G Y Y G
251 Y S S A R Y G A G G G W L G G Q T Y G R S G Y S G G Y S S G W A G A G A A S S A R A S S S A S S S T
301 S V T V S G G G H Y G G G G H G G G Y G G G H G G G S T G G H G G C C G R
```

14

Caulobacter henricii WP_081942626.1

Articulin
VYV modules

MVRTRVRIV

ERPVYV
EKIVKQPVYI
EKRTEVPV
DRPVYI
DRPV
DRIV
EKRVEVPV
DRIV
EKRVEVPVVKIV
EKPV
DRPVYI
DRPV
DRII
EKRVEIPVDRPVYVDRPVDRVI
EKRVEVPVDRPVYVDRPVDRVV
ERRVEVPVDRPVYVDRVV
EKRVEVPV
ERIVRTPVYV

ERQERRVERRGCDCDGDRGYYEQGRVYDRAEGSVERSEESYESESSRYSEE
GQGWSSQGSYMA**G**DGYAQSLGGVGGGGYYGYSSARYGAGGGWLGGQTYG
RSGYSGGYSSGWAGAGAASSARASSSASSSTSVTVSGGGHYGGGGHGGGY
GGGHGGGHGGGSTGGHGGCCGR

Crescentin YP_002519186.1

15

MRLLSKNSRETKNGKPTVLGDEARAEAMQHQIESTQAIGQRYETIHGGLDSI
GRVMEHLKAIEPLIAEIRGPVSQEFEARRAEHAELIAVRANLDQAQRQIALIQAE

EREVSARLAAAETALGESDARRQTQDAALEDNALEI
DRLRNALLQSDLKVSSLDASL
RDATARIEHLVQDVEGLRVQAQDIDARRGDAEAALARANQDNALLGEEAATL
KKRVDQAGLDLARLSRIETDLEAQLAA
ERARVQAVENALAAHQADSGRTIRGLESQVEANRAEISALQTRLETATGRA
DKLEEMNGQISARLADSSAQQKAV
ERRAGDLNVAL
ERAL
DRIRALEEEADGLRQRHAGVDTARATAI
ERADQLAKSAVAQ
EKALKRAE
ERAQQLRARLDAMQEAQDQVR
RDHEAKIAELQATI
ERLTSEAALAEGALEAAR
RDRSRLQMALLGASDGDVAASA

1 M R L L S K N S R E T K N G K P T V L G D E A R A E A M Q H Q I E S T Q A I G Q R Y E T I H G G L D
51 S I G R V M E H L K A I E P L I A E I R G P V S Q E F E A R R A E H A E L I A V R A N L D Q A Q R Q
101 I A L I Q A E E R E V S A R L A A A E T A L G E S D A R R Q T Q D A A L E D N A L E I D R L R N A L
151 L Q S D L K V S S L D A S L R D A T A R I E H L V Q D V E G L R V Q A Q D I D A R R G D A E A A L A
201 R A N Q D N A L L G E E A A T L K K R V D Q A G L D L A R L S R I E T D L E A Q L A A E R A R V Q A
251 V E N A L A A H Q A D S G R T I R G L E S Q V E A N R A E I S A L Q T R L E T A T G R A D K L E E M
301 N G Q I S A R L A D S S A Q Q K A V E R R A G D L N V A L E R A L D R I R A L E E E A D G L R Q R H
351 A G V D T A R A T A I E R A D Q L A K S A V A Q E K A L K R A E E R A Q Q L R A R L D A M Q E A Q D
401 Q V R R D H E A K I A E L Q A T I E R L T S E A A L A E G A L E A A R R D R S R L Q M A L L G A S D
451 G D V A A S A

16

BLAST hits to *Chroomonas*MMETSP0047_c25199_g1_i1_g48336 (1)

Description	Max score	Total score	Query cover	E value	Ident	Accession
hypothetical protein GUITHDRAFT_162236 [Guillardia theta CCMP2712]	87.8	129	95%	7e-17	36%	XP_005836053.1
hypothetical protein BCR39DRAFT_591213 [Naematelia encephala]	83.6	269	99%	4e-15	44%	ORY22767.1
hypothetical protein C367_01045 [Cryptococcus neoformans var. grubii Ze90-1]	74.3	203	99%	6e-12	44%	OXG32277.1
hypothetical protein CNAG_03655 [Cryptococcus neoformans var. grubii H99]	72.4	122	97%	2e-11	40%	XP_012047367.1
hypothetical protein C361_01040 [Cryptococcus neoformans var. grubii Tu259-1]	72.4	162	97%	2e-11	40%	OXG27769.1
hypothetical protein C353_01066 [Cryptococcus neoformans var. grubii AD1-83a]	72.4	161	97%	2e-11	40%	OWZ52057.1
hypothetical protein C349_01145 [Cryptococcus neoformans var. grubii Br795]	72.4	123	97%	3e-11	40%	OXG88078.1
hypothetical protein C350_01054 [Cryptococcus neoformans var. grubii MW-RSA36]	72.0	121	97%	3e-11	40%	OXG86558.1
hypothetical protein J007_01050 [Cryptococcus neoformans var. grubii]	72.0	206	99%	3e-11	39%	OXB39147.1
hypothetical protein C356_01056 [Cryptococcus neoformans var. grubii c45]	72.0	207	100%	4e-11	39%	OWZ59297.1
hypothetical protein C347_01119 [Cryptococcus neoformans var. grubii AD2-60a]	71.6	159	97%	4e-11	39%	OWZ35382.1
hypothetical protein AYX13_01638 [Cryptococcus neoformans var. grubii]	71.6	200	99%	4e-11	39%	OXC69871.1
hypothetical protein C364_01035 [Cryptococcus neoformans var. grubii Bt63]	71.6	251	99%	5e-11	38%	OXM81172.1
hypothetical protein C358_01047 [Cryptococcus neoformans var. grubii MW-RSA852]	71.2	123	97%	5e-11	39%	OXC64217.1
hypothetical protein C366_01032 [Cryptococcus neoformans var. grubii Tu401-1]	71.2	250	99%	5e-11	38%	OXG22134.1
hypothetical protein C365_01100 [Cryptococcus neoformans var. grubii Bt85]	71.2	249	99%	5e-11	38%	OWZ80149.1
hypothetical protein C355_01116 [Cryptococcus neoformans var. grubii Th84]	71.2	199	99%	6e-11	39%	OXG53139.1
hypothetical protein J000_01038 [Cryptococcus neoformans var. grubii]	71.2	197	99%	6e-11	39%	OXH74095.1
hypothetical protein AYX14_03263 [Cryptococcus neoformans var. grubii]	71.2	199	99%	6e-11	38%	OWZ71333.1
hypothetical protein H072_6521 [Dactylellina haptotyla CBS 200.50]	70.9	272	99%	7e-11	38%	EPS39696.1
hypothetical protein AYX15_04466 [Cryptococcus neoformans var. grubii]	70.5	198	99%	1e-10	38%	OWZ63593.1
hypothetical protein C362_00532 [Cryptococcus neoformans var. grubii Bt1]	70.5	120	99%	1e-10	39%	OWT42160.1
hypothetical protein J008_01035 [Cryptococcus neoformans var. grubii]	70.5	197	99%	1e-10	39%	OXH40688.1
hypothetical protein CALVIDRAFT_558348 [Calocera viscosa TUFC12733]	67.8	169	99%	9e-10	43%	KZO91079.1
PREDICTED: titin isoform X2 [Zeugodacus cucurbitae]	67.0	186	100%	2e-09	36%	XP_011189876.1
PREDICTED: titin isoform X1 [Zeugodacus cucurbitae]	67.0	186	100%	2e-09	36%	XP_011189875.1
hypothetical protein CALCODRAFT_505507 [Calocera cornea HHB12733]	66.2	243	97%	3e-09	44%	KZT62434.1
hypothetical protein I352_05180, partial [Cryptococcus gattii VGII MMRL2647]	65.5	161	97%	6e-09	40%	KIR32353.1
uncharacterized protein LOC111508187 [Leptinotarsa decemlineata]	65.1	126	99%	9e-09	37%	XP_023019384.1
hypothetical protein PPERSA_02566 [Pseudocohnilembus persalinus]	64.7	511	99%	1e-08	36%	KRX09694.1
PREDICTED: skin secretory protein xP2-like [Diaphorina citri]	64.3	164	98%	1e-08	38%	XP_017299517.1
hypothetical protein I313_06003 [Cryptococcus gattii VGII Ram5]	64.3	149	99%	1e-08	40%	KIR38008.1
hypothetical protein CNBB4170 [Cryptococcus neoformans var. neoformans B-3501A]	63.5	203	99%	3e-08	41%	XP_777186.1
tubulin binding protein, putative [Cryptococcus neoformans var. neoformans JEC21]	63.5	203	99%	3e-08	41%	XP_024512134.1
hypothetical protein L198_02309 [Tsuchiyaea wingfieldii CBS 7118]	63.2	206	99%	3e-08	40%	XP_019033513.1
hypothetical protein CNBG_0058 [Cryptococcus gattii VGII R265]	63.2	63.2	97%	4e-08	39%	KGB74220.2
articulin [Caulobacter sp. Root656]	62.4	110	100%	4e-08	40%	KRA75086.1
conserved hypothetical protein [Culex quinquefasciatus]	62.8	102	84%	5e-08	39%	XP_001866136.1
hypothetical protein I309_06410, partial [Cryptococcus gattii VGII LA55]	62.8	122	99%	5e-08	40%	KIR24750.1
hypothetical protein FF38_10240 [Lucilia cuprina]	62.4	167	97%	6e-08	40%	KNC31755.1
titin-like [Lucilia cuprina]	62.4	167	97%	6e-08	40%	XP_023293383.1
hypothetical protein CC85DRAFT_93486 [Cutaneotrichosporon oleaginosum]	62.4	361	100%	7e-08	44%	XP_018278880.1
hypothetical protein I310_01652 [Cryptococcus gattii VGII CA1014]	62.0	182	99%	8e-08	40%	KIR74055.1
hypothetical protein RHOSPDRAFT_37112 [Rhodotorula sp. JG-1b]	61.2	166	98%	1e-07	45%	KWU41351.1
hypothetical protein [Caulobacter sp. Root1472]	60.5	108	100%	2e-07	38%	WP_056752882.1
hypothetical protein GUITHDRAFT_117614 [Guillardia theta CCMP2712]	60.8	221	98%	2e-07	35%	XP_005823239.1
hypothetical protein I304_02100 [Cryptococcus gattii VGII CBS 10090]	60.1	158	97%	4e-07	42%	KIR94458.1
hypothetical protein I308_05094 [Cryptococcus gattii VGIV IND107]	59.7	99.4	97%	6e-07	38%	KIR84507.1

17 BLAST hits to *Chroomonas*MMETSP0047_c25199_g1_i1_g48336 (2)

hypothetical protein I304_02100 [Cryptococcus gattii VGII CBS 10090]	60.1	158	97%	4e-07	42%	KIR94458.1
hypothetical protein I308_05094 [Cryptococcus gattii VGIV IND107]	59.7	99.4	97%	6e-07	38%	KIR84507.1
PREDICTED: titin [Stomoxys calcitrans]	59.3	113	81%	6e-07	42%	XP_013098347.1
cytoskeletal protein [Euglena gracilis]	58.9	204	85%	8e-07	38%	CAA78363.1
hypothetical protein AOL_s00078g398 [Arthrobotrys oligospora ATCC 24927]	59.3	117	97%	8e-07	40%	XP_011121963.1
PREDICTED: titin [Rhagoletis zephyria]	58.9	105	80%	9e-07	38%	XP_017485291.1
titin [Ceratitis capitata]	58.9	150	90%	9e-07	36%	XP_012155257.1
hypothetical protein L804_05855, partial [Cryptococcus gattii VGII 2001/935-1]	58.9	160	99%	1e-06	39%	KIR96757.1
Tubulin binding protein, putative [Cryptococcus gattii WM276]	58.5	256	97%	1e-06	36%	XP_003192384.1
80 kda articulin=major membrane skeletal protein [Euglena gracilis, Peptide, 651 aa]	58.2	249	87%	1e-06	38%	AAB23240.1
hypothetical protein [Nocardia sp. BMG111209]	58.2	139	99%	1e-06	38%	WP_019925771.1
hypothetical protein BGN86_01150 [Caulobacterales bacterium 68-7]	58.2	115	95%	1e-06	43%	OJU12606.1
PREDICTED: titin [Bactrocera latifrons]	56.6	98.6	84%	5e-06	37%	XP_018783252.1
hypothetical protein CONPUDRAFT_167623 [Coniophora puteana RWD-64-598 SS2]	56.6	56.6	93%	6e-06	42%	XP_007771658.1
titin isoform X1 [Aedes aegypti]	55.5	55.5	76%	1e-05	39%	XP_001659466.2
AAEL008752-PA [Aedes aegypti]	55.5	55.5	76%	1e-05	39%	EAT39445.1
titin isoform X2 [Aedes aegypti]	55.5	55.5	76%	2e-05	39%	XP_021708219.1
hypothetical protein PPERSA_12380 [Pseudocohnilembus persalinus]	55.1	258	99%	2e-05	37%	KRW98423.1
articulin [Caulobacter sp. UNC279MFTsu5.1]	54.3	138	99%	3e-05	43%	WP_089929575.1
hypothetical protein SAMN02799626_03325 [Caulobacter sp. UNC279MFTsu5.1]	53.9	137	100%	3e-05	42%	SFK07116.1
hypothetical protein [Gordonia sihwensis]	54.3	96.7	99%	4e-05	37%	WP_045537586.1
hypothetical protein RP20_CCG005438 [Aedes albopictus]	53.9	53.9	76%	5e-05	36%	KXJ78170.1
uncharacterized protein LOC111002932 [Pieris rapae]	53.5	94.0	79%	6e-05	42%	XP_022128914.1
PREDICTED: titin [Bactrocera oleae]	53.5	96.7	90%	7e-05	38%	XP_014088400.1
PREDICTED: titin-like isoform X2 [Aedes albopictus]	53.5	53.5	76%	7e-05	35%	XP_019544309.1

18

Cryptococcus neoformans (basidiomycete) tubulin binding protein XP_568826.1

(First half of sequence)

MGSPLPRQRSRANLKHALPAPPSTRSLASMAGNSPTSSPTPQRRSEITADAL
RSHLSSLLEQKSSQLQMLGTMGQEILRQQQELEERIRDFEGEGIEGDAIAEET
KEKLRELDEAMKAWENQNEDMMRELGGKHRQLDMEFATEIGQNLLVEVRRL
QALLSERDRALEKLSEEKETWEQESQTLLIAVRAAESSVDRYKEENWNLEVN
LQELRSSVADMQDQLTKANAEQARLQKTLVSSREAAEAYKTEAEKHAQAIEE
LKAKHETDMAQARRTTAGLQRDKSDLLGELNHERQRRVSARGRMSQSLSAS
PGMLSPNHLDSDEDDVFAAGGKGNSSPTKRGPGFDANDNALSPSQLYESDF
DSPNPTPSKPFPRSPLGEMFVSENDELREKLRVAEQEIEALKSENERVRLGSF
SKKESVVDEFGTRAPAGEWEEDEHTIGASSRGRGSTRGRRGGRGKNFAASI
GRKFGFNRAVSGLSTPNAGDRSFNSTSSGTPDLLRPRDMSASPAPSVIGGET
LGNVLGNRSVERLGSASPFNTPSIDSLKANFGQPTALADEIGAPIEPSTDYVDA
GIMTDKWSPEKPIMPGTQSPAVIPSPTTPTELIASWGAETPKRGVRDESQFAN
LPPLPPPPATPTAAGDTTPTKKNTPLPMPSRLQQVFAPISIDDSMSITTDTDAD
DYESAAETIGSLTPNRTQSELPTDTEAYQTGREWPNESSAEDSDTEEVQEQE
HTLRGLKASSTIGLGLASAAAGGWAAAKQAHKMASRDRI ADAPV

EKIV
EKIVEV
EKRIEVPV
DRIVEV
EKRVEVPV
DRIVEV
EKII
EKIVEVPV
ERIIEV
EKIVEIPV
EKIVEV
EKIVEV
EKRIEVPV
DRIIEV
EKRVEVPV
DRIVEV
EKII
EKIVEVPV
EKIIEV
EKIV
EKIVEVPV
DRIVEVPV
EKIIEV
EKLVEVPVEV
EKIV
EKIV
EKIIEVPVEV
EKIVEVPV
EKIVEV
EKRVEVPVEV
EKIVEVPVEV
EKII
EKIVEVPI
DKIIEI
EKRVEVPVEI
EKIVEVPVETIV
EKIVEV
EKIV
EKTVEVPV
EKIIEV

19

Cryptococcus neoformans (basidiomycete) tubulin binding protein XP_568826.1 (Second half of sequence)

Predicted secondary structure next slide

EKIVEVPV
EKIIEV
EKIV
EKIVEVPV
EKIIEV
EKIVEVPKIIEV
EKVV
EKIVEV
EKEVI
KEVEV
EKIV
EKIVEVEVV
KEVPVNVDV

EKIV
ERVVEV
EKPV
EKIIEVPKIVEV
EKIV
EKVIEVI
KEVP
KEIEII
KEVEV
EKIVEVI
KEVEV
EKIVEVI
KEVEV
EKVV
ERIVEVPVEV
EKIVEVEV
EKRVEVPFEV
EKIVEV
EKVVEVLVEV
EKRVEVPVEV
EKIV
EKTIEVPMEV
EKIV
EKIVEVPVEI
EKRIEVPVEV
EKIVQIPVEV
EKIV
EKMVEVPVEV
EKIV
EKVVEVPIEI
ERIVEVQKIVEVPVEV
EKIV
ERIVELPVEVQRRIEVPV
EKIVEV
ERIVEV
EKIVEVLV
EKIVEVPV
ERVV
ERQVEVPVEI
ERII
ERRVEVPI
EKIVTI

20

Cryptococcus neoformans (basidiomycete) tubulin binding protein XP_568826.1

Predicted secondary structure

1 M G S P L P R Q R S R A N L K H A L P A P P S T R S L A S M A G N S P T S S P T P Q R R S E I T A D
51 A L R S H L S S L L E Q K S S Q L Q M L G T M G Q E I L R Q Q Q E L E E R I R D F E G E G I E G D A
101 I A E E T K E K L R E L D E A M K A W E N Q N E D M M R E L G G K H R Q L D M E F A T E I G Q N L L
151 V E V R R L Q A L L S E R D R A L E K L S E E K E T W E Q E S Q T L L I A V R A A E S S V D R Y K E
201 E N W N L E V N L Q E L R S S V A D M Q D Q L T K A N A E Q A R L Q K T L V S S R E A A E A Y K T E
251 A E K H A Q A I E E L K A K H E T D M A Q A R R T T A G L Q R D K S D L L G E L N H E R Q R R V S A
301 R G R M S Q S L S A S P G M L S P N H L D S D E D D V F A A G G K G N S S P T K R G P G F D A N D N
351 A L S P S Q L Y E S D F D S P N P T P S K P F P R S P L G E M F V S E N D E L R E K L R V A E Q E I
401 E A L K S E N E R V R L G S F S K K E S V V D E F G T R A P A G E W E E D E H T I G A S S R G R G S
451 T R G R R G G R G K N F A A S I G R K F G F N R A V S G L S T P N A G D R S F N S T S S G T P D L L
501 R P R D M S A S P A P S V I G G E T L G N V L G N R S V E R L G S A S P F N T P S I D S L K A N F G
551 Q P T A L A D E I G A P I E P S T D Y V D A G I M T D K W S P E K P I M P G T Q S P A V I P S P T T
601 P T E L I A S W G A E T P K R G

1 V R D E S Q F A N L P P L P P P P A T P T A A G D T T P T K K N T P L P M P S R L Q Q V F A P I S I
51 D D S M S I T T D T D A D D Y E S A A E T I G S L T P N R T Q S E L P T D T E A Y Q T G R E W P N E
101 S S A E D S D T E E V Q E Q E H T L R G L K A S S T I G L G L A S A A A G G W A A A K Q A H K M A S
151 R D R I A D A P V E K I V E K I V E V E K R I E V P V D R I V E V E K R V E V P V D R I V E V E K I
201 I E K I V E V P V E R I I E V E K I V E I P V E K I V E V E K I V E V E K R I E V P V D R I I E V E
251 K R V E V P V D R I V E V E K I I E K I V E V P V E K I I E V E K I V E K I V E V P V D R I V E V P
301 V E K I I E V E K L V E V P V E V E K I V E K I V E K I I E V P V E V E K I V E V P V E K I V E V E
351 K R V E V P V E V E K I V E V P V E V E K I I E K I V E V P I D K I I E I E K R V E V P V E I E K I
401 V E V P V E T I V E K I V E V E K I V E K T V E V P V E K I I E V E K I V E V P V E K I I E V E K I
451 V E K I V E V P V E K I I E V E K I V E V P K I I E V E K V V E K I V E V E K E V I K E V E V E K I
501 V E K I V E V E V V K E V P V N V D V E K I V E R V V E V E K P V E K I I E V P K I V E V E K I V E
551 K V I E V I K E V P K E I E I I K E V E V E K I V E V I K E V E V E K I V E V I K E V E V E K V V E
601 R I V E V P V E V E K I V E V E V E K R V E V P F E V E K I V E V E K V V E V L V E V E K R V E V P
651 V E V E K I V E K T I E V P M E V E K I V E K I V E V P V E I E K R I E V P V E V E K I V Q I P V E
701 V E K I V E K M V E V P V E V E K I V E K V V E V P I E I E R I V E V Q K I V E V P V E V E K I V E
751 R I V E L P V E V Q R R I E V P V E K I V E V E R I V E V E K I V E V L V E K I V E V P V E R V V E
801 R Q V E V P V E I E R I I E R R V E V P I E K I V T I E K R I E I P I E R I V T V E K I V E V P V A
851 A N K S L F S D S T S Q T E P L A S V N S S S L A P N P D I G L F R V T P G T S Y D F L K A P P S T
901 V R R V S A D N L A A V A S G S A D S T K T V E T K S V P T S P A D K S Q P P M M N L P P P P S N P
951 P P S R I G K K M S M G P P P L P T S P H P D D F L Q R A T S P A F Q S T V N R R S S T R T A P S A
[illegible]

1 G A A I R A S G G D M P P P A S T S R K P S R S S F K P A A S A Q A T P V R D D V K S R Q S V R R R
51 T D N T F A S S G Y T S A N S S A T G H D Q L I E H D R N P S L S S F D S Y A G T V P K P R G V P T
101 A G S T D P Q T I H A I T Q T M I G E Y L Y K Y T R R A V G K G Q S S N R H K R F F W V H P Y T K T
151 L Y W S T E D P G S S R A S E S S A K S V F I S N V K V I E D T N I Q P P G L F T K S I V V S T P G
201 R E I Q I T A P T Q E R H E L W M S A L Q F L L Q K Q S P E A N N T L A E T T V R N S S R P A L A S
251 I A D E Q G R L T M P K S P L S L R S F G S E R H S L S N I T P K A N R S H S T M S N Y P R S A S T
301 M G K R A G T V A H E Y T R R H E V P Q P F H G G H R Y K G P Y K G A P I V D D E F D V V S R E D G
351 E D M D T S F E G L E N V R A C C D G K H L V G Q H N H H H D H P N V P R T P A R A E T P S L R G W
401 S M R S G R A S N V L S D G E S I F S T A K K R E E R S K S A M G H R D R R D G A K S P A M G S L R
451 N L K Q A

21

Kockovaella imperatae (basidiomycete) XP_021873516.1(First half of sequence)

MDDPFGSPTTSTLPRQRSRPNLAHHLPAPPSSRSLASQLRDSPTKPSQKLEY
TGHALHQHLSSLLEQKTAQLQMLGTMGQEILKQQQELEERIKGFDEDDGIDEE
VGEDTKVKLKDLDEAMRQWEAQNEDMMRELGGKMPDSLEGLDLKAPVMEN
GTTSASMNRRQRNAQHRALDMEFATEIGQNLLAEVRRLQSLLNEREKSVEKL
NEEKDSWEVEKASLVGAVRNAESSVDRYKEENWNLEVNLQELRAAHSDAQE
QLTKSNIEHTRLNRSILSVRELAEMHKTDAEKNAQLLEDLKAKHETDMAQARKN
YAGLQRDKSDLLTELHVERNRRVSALRGRMSRGASASPGLSTPGGADESFEE
DEDVFAATVDDARKRSLAAENNALSPGNITAFSSSPDPTPSKDRVPLSDVYINE
IDELRENLERARAEIATLKGEQHKARRMASREDITPRTSGDWEEEDSILGRGRG
TVRGRRGRGRGFAASLSRKLGFSRVPSSLSNGSTPNDKSFNSTSTGTPDLLRY
RESVSPGLDSPSLASRALSSETSLDRPTSFMGPSTALADELGSGNLDSMDYAS
GSNATDWSPEKQPQAYLENQVEVTTPTKGVFAESTKSNIEESTQPTGERTPTK
PAKALPIAVISRRPSSVADTTTDGETDYADAESTMDVATPREGTAELPTDADES
SYATGREYATAESS AGSDSDEGDYTQRLGISGSMLGLGGAAAAAGGYAAYKQ
AHKVASRDLI

REKIV
ERIVEVPV
EKIVEV
EKRVEVPVEVI
KEVMV
EKPIEVI
KEVFV
DRPVEVI
KEVII
DRPVEVI
KEVIV
EKPVEVI
KEVLS
EKPIEVI
KEIIV
DRPVEVI
KEVIV
EKPVEVI
KEVIV
DRPVEVI
KEVIV
EKLVEVI
KEVIV
DRPVEVI
KEVVV
EKPIEVI
KEVIV
DRHVEVI
KEVPV
DRPVEII
KEVII
DRPVEVI
KEVIV
DRPVEII
KELVV
EKSVEVI
KEV
EKPVEVI
KEV
EKIIEVPKIVEV

Kockovaella imperatae (basidiomycete)
XP_021873516.1(Second half of sequence)

22

Predicted secondary structure next slide.

EKIV
ERIV
EKEVEVPKIVEV
EKII
EKIVNV
DREV
EKPV
ERIVEIPKIVEV
EKIV
EKIV
EKPVEVFKQVEV
EKIVEV
ERIV
EKPVEVI
REV
EKIVEVQV
DRVVEVPV
EKIV
EKIVEVPVEVV
KEV
EKIVEVPVEVI
KEV
EKIVELPVEVI
REVIKTIEVPVEVLKTVEIPVEVI
KEVSKTVEVPIEVVRIVEVI
KEV
EKIVEVPVEVIKTVEVPV
DRIV
EKIV
EKRVEVPV
EKIVP

MPSPTPSSPTVSPDLGIWRVQPGANYDFLKAPPPSGSLGGKTRASTETSALG
LGFPADDGLPATRGLSPALTVDGLPPASPSASVDRTLPPTMSLPPPPTIPPP
AEQKKTSTGPPPRPLSPPPDEFVSRTARRASRPAVVTKGAMGPPESVIRKPS
RTSVRPPPSPAASAFETRRRGHRGTLPASGQSSFSMRNDNGSISSMGSVVPP
GEHKRSGSVVAGTTDPATIHAITQTMIGEYLWKYTRRALGKGQSENRHKRFF
WIHPYTKTLYWSASDPGSLGAQESSAKSVVITNVKAIDDANNSPPGLHSQSI
VVSTTGRDIQFTAESKDRHDLWMSALLFLLSESAPTGVANTLKHSSTVRAMS
SRSLAADEQGRLTYTPTIPKSPMSMRSLHSVNSFTMTPKAYPRPPSALSARA
ASAMEKRSGTPAFEYYRRHEVPATIHGGHRYKGTYKGAPFMEDFDVITAEDG
DDPDASFEGMDNVRACCDGRHTVGHHHHPENESVTGPPERPAPRSVTPSLRA
FSMRSRRASAASSKRPGIPKGKFREEEPAVPKIDERSKSVTGHI

23

Kockovaella imperatae (basidiomycete)
XP_021873516.1

Predicted secondary structure

```
   1 M D D P F G S P T T S T L P R Q R S R P N L A H H L P A P P S S R S L A S Q L R D S P T K P S Q K L
  51 E Y T G H A L H Q H L S S L L E Q K T A Q L Q M L G T M G Q E I L K Q Q Q E L E E R I K G F D E D D
 101 G I D E E V G E D T K V K L K D L D E A M R Q W E A Q N E D M M R E L G G K M P D S L E G L D L K A
 151 P V M E N G T T S A S M N R R Q R N A Q H R A L D M E F A T E I G Q N L L A E V R R L Q S L L N E R
 201 E K S V E K L N E E K D S W E V E K A S L V G A V R N A E S S V D R Y K E E N W N L E V N L Q E L R
 251 A A H S D A Q E Q L T K S N I E H T R L N R S I L S V R E L A E M H K T D A E K N A Q L L E D L K A
 301 K H E T D M A Q A R K N Y A G L Q R D K S D L L T E L H V E R N R R V S A L R G R M S R G A S A S P
 351 G L S T P G G A D E S F E E D E D V F A A T V D D A R K R S L A A E N N A L S P G N I T A F S S S P
 401 D P T P S K D R V P L S D V Y I N E I D E L R E N L E R A R A E I A T L K G E Q H K A R R M A S R E
 451 D I T P R T S G D W E E E D S I L S G R G R G T V R G R R G R G R G F A A S L S R K L G F S R V P S
 501 S L S N G S T P N D K S F N S T S T G T P D L L R Y R E S V S P G L D S P S L A S R A L S S E T S L
 551 D R P T S F M G P S
   1 T A L A D E L G S G N L D S M D Y A S G S N A T D W S P E K Q P Q A Y L E N Q V E V T T P T K G V F
  51 A E S T K S N I E E S T Q P T G E R T P T K P A K A L P I A V I S R R P S S V A D T T T D G E T D Y
 101 A D A E S T M D V A T P R E G T A E L P T D A D E S S Y A T G R E Y A T A E S S A G S D S D E G D Y
 151 T Q R L G I S G S M L G L G G A A A A A A G G Y A A Y K Q A H K V A S R D L I R E K I V E R I V E V P
 201 V E K I V E V E K R V E V P V E V I K E V M V E K P I E V I K E V F V D R P V E V I K E V I I D R P
 251 V E V I K E V I V E K P V E V I K E V L S E K P I E V I K E I I V D R P V E V I K E V I V E K P V E
 301 V I K E V I V D R P V E V I K E V I V E K L V E V I K E V I V D R P V E V I K E V V V E K P I E V I
 351 K E V I V D R H V E V I K E V P V D R P V E I I K E V I I D R P V E V I K E V I V D R P V E I I K E
 401 L V V E K S V E V I K E V E K P V E V I K E V E K I I E V P K I V E V E K I V E R I V E K E V E V P
 451 K I V E V E K I I E K I V N V D R E V E K P V E R I V E I P K I V E V E K I V E K I V E K P V E V F
 501 K Q V E V E K I V E V E R I V E K P V E V I R E V E K I V E V Q V D R V V E V P V E K I V E K I V E
 551 V P V E V V K E V E K I V E V P V E V I K E V E K I V E L P V E V I R E V I K T I E V P V E V L K T
 601 V E I P V E V I K E V S K T V E V P I E V V R I V E V I K E V E K I V E V P V E V I K T V E V P V D
 651 R I V E K I V E K R V E V P V E K I V P M P S P T P S S P T V S P D L G I W R V Q P G A N Y D F L K
 701 A P P P S G S L G G K T R A S T E T S A L G L G F P A D D G L P A T R G L S P A L T V D G L P P A S
 751 P S A S V D R T L P P T M S L P P P P T I P P P A E Q K K T S T G P P P R P L S P P P D E F V S R T
 801 A R R A S R P A V V T K G A M G P P E S V I R K P S R T S V R P P P S P A A S A F E T R R R G H R G
 851 T L P A S G Q S S F S M R N D N G S I S S M G S V V P P G E H K R S G S V V A G T T D P A T I H A I
 901 T Q T M I G E Y L W K Y T R R A L G K G Q S E N R H K R F F W I H P Y T K T L Y W S A S D P G S L G
 951 A Q E S S A K S V V I T N V K A I D D A N N S P P G L H S Q S I V V S T T G R D I Q F T A E S K D R
1001 H D L W M S A L L F L L S E S A P T G V A N T L K H S S T V R A M S S R S L A A D E Q G R L T Y T P
1051 T I P K S P M S M R S L H S V N S F T M T P K A Y P R P P S A L S A R A A S A M E K R S G T P A F E
1101 Y Y R R H E V P A T I H G G H R Y K G T Y K G A P F M E D F D V I T A E D G D D P D A S F E G M D N
1151 V R A C C D G R H T V G H H H H P E N E S V T G P P E R P A P R S V T P S L R A F S M R S R R A S A
1201 A S S K R P G I P K G K F R E E E P A V P K I D E R S K S V T G H I
```

24

Zeugodacus (fly) titan isoform XP_011189876.1

Predicted secondary structure next slide

MDTQLSIQHRQHRLRLPLLLLFFLCYFASSQAKRTTRRLPRDEVHEYAIHQY
NYAERDTQEPSGSVADLHADESKREVQARPPKGSRTTAGMTRDKRGAKKIRH
IVHLVNTAQQQKKKKPLTTNTRSNSHQHRQQNAVEDDSKSFHTYEETHEHIV
EPAQEQESAPKPAFASSPGHGAKLGLKSSTAHTQNEPLSQASTDNYATHAFL
KPPEEPHEEPEMSSTEHHEHEIQHEEEEHHHVEKIKVKHHHHHHHHHNHV

KEIIKKVPEPYPV
EKIVHVPV
EKIV
EKLVHVPKPYPV
EKII
EKKVPYPV
EKII
EKVV
EKKVPYPV
EKIV
EKIVHVPV
EKMVHVPQPYPV
EKII
EKKVPYPV
EKII
EKVV
EKKVPYPV
EKIV
EKIVHVPV
EKIVHVPKPYPV
EKII
EKKVPYPV
EKIV
EKVV
EKKVPYPV
EKIV
EKVVHVPKPYPV
EKVI
EKIV
EKKIHVPV
EKIV
EKIIHIPKPFPV
EKIV
EKVVHVPKP

FPVIKHIPYPVEVKVPVHIEKPVPYPVEKKVHVPYRVEVEKKIPVPYKVEVEK
KVPIFIHTPHEHHEPYKFEHHEHEHKEEQHEQHEEKHDFEEHQHFEHHEHHHD
LSGYPTNQKANYARLQQQQPRHIANGAKAVLAKAHTPAAAAAIRAELLHQQVQ
NFGYKITTSATPTDLEQSSSDNKSQAMRLVPTPTANNFGFKQPTPAAPSDLES
SASENKPVVAHFEPTSLPFRIHVDDGATSADGSSTLGAPSSTNDMQAQASTHS
FRMLARLQPIALPVQVYQFHSIPFQQPLGFSLPALRAAVPSGGP

25

Zeugodacus (fly) titin isoform XP_011189876.1

Predicted secondary structure

```
  1 M D T Q L S I Q H R Q H R L R L P L L L L F F L C Y F A S S Q A K R T T R R L P R D E V H E Y A I H
 51 Q Y N Y A E R D T Q E P S G S V A D L H A D E S K R V E V Q A R P P K G S R T T A G M T R D K R G A
101 K K I R H I V H L V N T A Q Q Q K K K K P L T T N T R S N S H Q H R Q Q N A V E D D S K S F H T Y E
151 E T H E H I V E P A Q E Q E S A P K P A F A S S P G H G A K L G L K S S T A H T Q N E P L S Q A S T
201 D N Y A T H A F L K P P E E P H E E P E M S S T E H H E H E I Q H E E E E H H H V E K I K V K H H H
251 H H H H H N H V K E I I K K V P E P Y P V E K I V H V P V E K I V E K L V H V P K P Y P V E K I I E
301 K K V P Y P V E K I I E K V V E K K V P Y P V E K I V E K I V H V P V E K M V H V P Q P Y P V E K I
351 I E K K V P Y P V E K I I E K V V E K K V P Y P V E K I V E K I V H V P V E K I V H V P K P Y P V E
401 K I I E K K V P Y P V E K I V E K V V E K K V P Y P V E K I V E K V V H V P K P Y P V E K V I E K I
451 V E K K I H V P V E K I V E K I I H I P K P F P V E K I V E K V V H V P K P F P V I K H I P Y P V E
501 V K V P V H I E K P V P Y P V E K K V H V P Y R V E V E K K I P V P Y K V E V E K K V P I F I H T P
551 H E H H E P Y K F E H H E H E H K E E Q H E Q H E E K H D F E E H Q H F E H H E H H H D L S G Y P T
601 N Q K A N Y A R L Q Q Q Q P R H I A N G A K A V L A K A H T P A A A A A I R A E L L H Q Q V Q N F G
651 Y K I T T S A T P T D L E Q S S S D N K S Q A M R L V P T P T A N N F G F K Q P T P A A P S D L E S
701 S A S E N K P V V A H F E P T S L P F R I H V D D G A T S A D G S S T L G A P S S T N D M Q A Q A S
751 T H S F R M L A R L Q P I A L P V Q V Y Q F H S I P F Q Q P L G F S L P A L R A A V P S G G P
```

26

Zootermopsis nevadensis (termite)
XP_021932507.1

MKITIVVLFLALAILATAENNQPGHKEAEKKEAAIKQTEEKKNDQQKTEESK
QTEQKQEVEQGDRKQEKRGLIHDFYGVGFGHGLDSGIFGGYDHTLHQEHVKT
ITLT

EKVPVPQPYPVHVPV
EKHVPYPV
EKPVPYPVKVPV
DRPYPVPVTVHKPVPYTVHKPVPYAVKVPV
DKPYPVHVTV
EKKVPYPVEVKVPVPQPYTVHVPKPYTVIV
EKKVPAPYPVHVKVPVPQPYPVHVKVPVTVHV
DRPVPVEVKVPV
DRPIPVPV
EKPYPVPV
EKKVPYPV
EKPVPYPVKVPY
DRPIPVHVVKPYPVHV
EKKVPYPV
EKIVPYPV
EKQVPYPVKVPY
DRPVPVHVDKPYPVPV
EKEIPYPV
ERPVPYPVKVPV
EKEVPYPV
EKPVPYPVKVPV

AIPVHHEHGGIEYGGLEHGGFEQGGFGQGGFGHGGLEHGGFGEGFGHHK

```
  1 M K I T I V V L F L A L A I L A T A E N N Q P G H K E A E K K E A A I K Q T E E K K N D Q Q K T E E
 51 S K Q T E Q K Q E V E Q G D R K Q E K R G L I H D F Y G V G F G H G L D S G I F G G Y D H T L H Q E
101 H V K T I T L T E K V P V P Q P Y P V H V P V E K H V P Y P V E K P V P Y P V K V P V D R P Y P V P
151 V T V H K P V P Y T V H K P V P Y A V K V P V D K P Y P V H V T V E K K V P Y P V E V K V P V P Q P
201 Y T V H V P K P Y T V I V E K K V P A P Y P V H V K V P V P Q P Y P V H V K V P V T V H V D R P V P
251 V E V K V P V D R P I P V P V E K P Y P V P V E K K V P Y P V E K P V P Y P V K V P Y D R P I P V H
301 V V K P Y P V H V E K K V P Y P V E K I V P Y P V E K Q V P Y P V K V P Y D R P V P V H V D K P Y P
351 V P V E K E I P Y P V E R P V P Y P V K V P V E K E V P Y P V E K P V P Y P V K V P V A I P V H H E
401 H G G I E Y G G L E H G G F E Q G G F G Q G G F G H G G L E H G G F G E G F G H H K
```
