# Supplementary material for: Epiplasts: Membrane Skeletons and Epiplastin Proteins in Euglenids, Glaucophytes, Cryptophytes, Ciliates, Dinoflagellates, and Apicomplexans
Source: mBio. 2018 Oct 30;9(5):e02020-18. doi: 10.1128/mBio.02020-18 (PMC6212826; doi:10.1128/mBio.02020-18)
Supplement: TEXT S11 [file mbo005184120s11.docx]

Supplement 11: Evolutionary Scenario

Glaucophytes are widely posited to represent the most direct living descendants of the original host cell that engulfed and domesticated a cyanobacterium to generate the modern chloroplast (1). Our results suggest that this host cell produced epiplasts/epiplastin and utilized mucocyst-type organelles (Supp Fig. 11.1A-C) for defense, both traits conserved in glaucophytes

___________________________________________________________
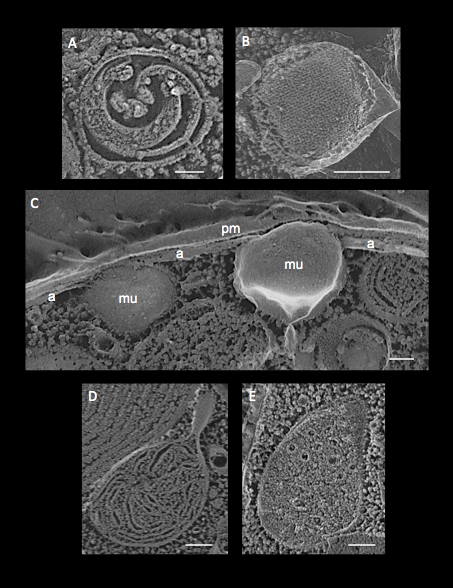


______________________________________________________________________________

Supp Figure 11.1 Mucocyst-like organelles: A) Ejectosome in *Guillardia*. Scale bar, 100 nm. B) Trichocyst in *Glenodinium*. Scale bar, 100 nm. C) Mucocysts (mu) positioned for secretion at junctions of alveolae (a) in *Cyanophora*. pm, plasma membrane. Scale bar, 100 nm. Mitochondrial cristae: D) Flat mitochondrial cristae in *Cyanophora*. Scale bar, 200 nm. E) Round mitochondrial cristae in *Thalassiosira*. Scale bar, 200 nm.

______________________________________________________________________________

and later lost in red and green lineages. There remains, however, a conundrum relating to mitochondria and alveolae.

While mitochondria are monophyletic (2), their morphology has been subject to numerous modifications, notably in the Excavates and the Amoebozoa, and is responsive to physiological status (3,4). That said, two readily distinguished crista phenotypes, designated flat/disc-like and round/tubular/ampulliform (Supp Fig. 11.1D and E), have served as useful criteria in higher taxonomic rankings (5,6). Mitochondria with flat cristae are ubiquitous in the eponymous Discicristata and in the Opisthokonta, Archaeplastida, and Cryptophyta, while round cristae are ubiquitous in Alveolata, Stramenopiles, Haptophyta, and Rhizaria (5.7). Glaucophyte mitochondria are flat (Supp Fig 11.1D), suggesting that the cyanobacterium-containing host had flat mitochondria, but the only other extant group with alveoli is the Alveolata, which has round mitochondria.

These observations are compatible with the following hypothetical sequence, illustrated in Supp Fig. 11.2. Many lineages are not included in the proposal and figure, and it is not intended as an evolutionary tree (e.g. the relationships between alveolates, stramenopiles, rhizaria, and haptophytes, while still subject to debate, are certainly more complex than illustrated, and the branching patterns of the glaucophytes, rhodophytes, and Viridiplantae are also debated). Rather, the intent is to offer a testable hypothesis, assuming deep evolutionary relationships, based on epiplast formation and mitochondrial morphology.


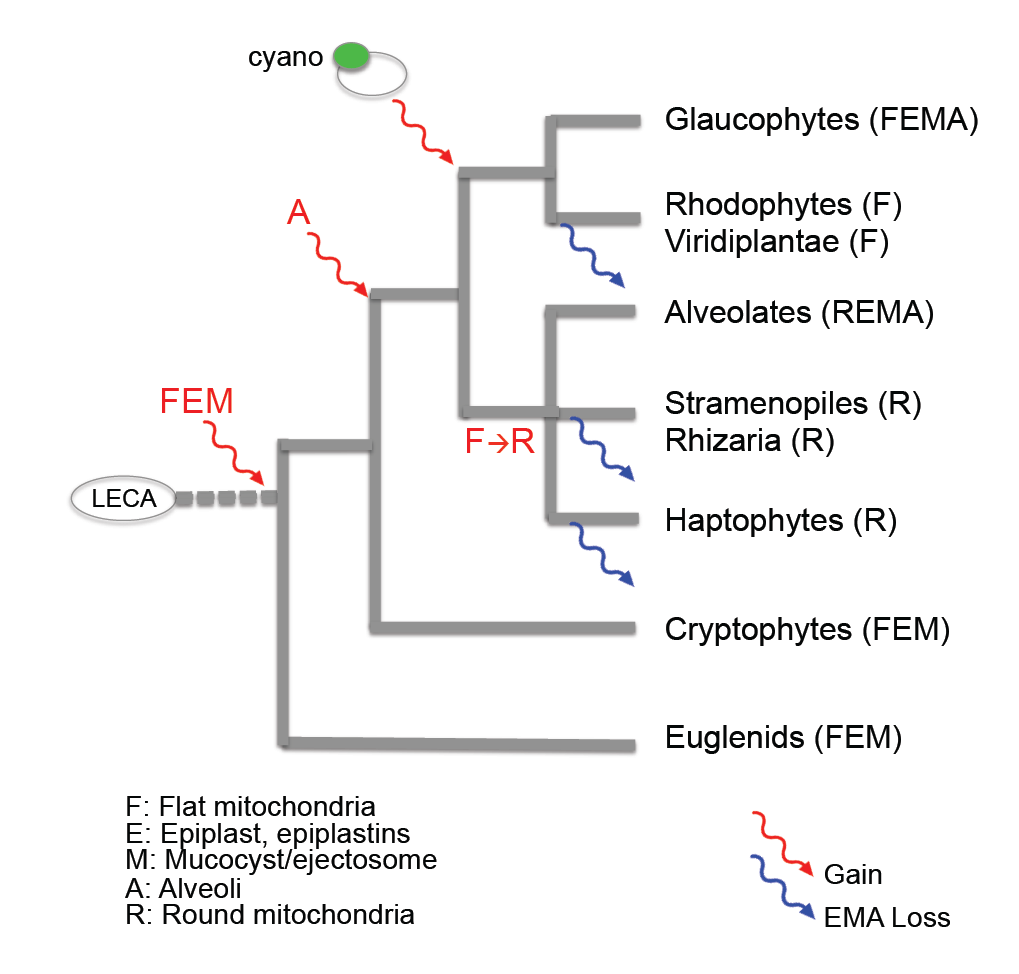


_____________________________________________________________________________

Supp Figure 11.2 Diagram of an evolutionary scenario suggested by this study. Lines denote posited lineage relationships based on the listed traits (FEMAR) and not evolutionary distances.

______________________________________________________________________________

1) An ancient flagellated bacteriovorous eukaryote possessed an epiplast and epiplastins, mucocyst-like organelles, and flat mitochondria (FEM); modern euglenids and cryptophytes are direct descendants of this forebear. 2) An early lineage derived from this forebear invented the insertion of alveoli between its epiplast and its plasma membrane, after which it diverged into two sublineages, one retaining flat mitochondria (FEMA) and the other developing round mitochondria (REMA). 3) A member of the “flat” sublineage served as the cyanobacterial host to found the glaucophytes, with subsequent alveolar/epiplastin/mucocyst loss, and cell-wall acquisition, in the red and green algae and land plants. 4) The “round” sublineage gave rise to the alveolates and, with alveolar/epiplastin/mucocyst loss, and cell-wall acquisition, to the Stramenopiles, Haptophytes, and Rhizaria. This sequence suggests that the non-alveolar forebear and its alveolar sublineages arose very early in eukaryotic evolution; hence the deep phylogenomic distances that separate their modern descendants (3,5) are to be expected.

**References**

1. Price DC, Steiner JM, Yoon HS, Bhattacharya D, Löffelhardt W. 2016. Glaucophyta, p 23-87. *In* Archibald JM, Simpson AGB, Slamovits CH (ed), Handbook of the Protists. Springer International Publishing, Basel, Switzerland.

2. Gray MW, Burger G, Lang BF. 2001. The origin and early evolution of mitochondria. Genome Biol 2:1-5.

3. Scheffler IE. 2008. Mitochondria 2nd Ed. Wiley-Liss, Hoboken, NJ.

4. Cogliati S, Enriquez JA, Scorrano L. 2016. Mitochondrial cristae: Where beauty meets functionality. Trends Biochem Sci 41:261–273.

5. Adl SM, Simpson AGB, Lane CE, Lukeš J, Bass D, Bowser SS, Brown MW, Burki F, Dunthorn M, Hampl V, Heiss A, Hoppenrath M, Lara E, Le Gall L, Lynn DH, McManus H, Mitchell EAD, Mozley-Stanridge SE, Parfrey LW, Pawlowski J, Rueckert S, Shadwick L, Schoch CL, Smirnov A, Spiegel FW. 2012. The revised classification of eukaryotes. J Eukaryot Microbiol 59:429–493.

6. Okamoto N, Inouye I. 2005. The Katablepharids are a distant sister group of the Cryptophyta: A proposal for Katablepharidophyta division Nova/ Kathablepharida phylum novum based on SSU rDNA and beta-tubulin phylogeny. Protist 156:163–179.

7. Burki F. 2017. The convoluted evolution of eukaryotes with complex plastids. Secondary Endosymbioses 84:1–30.
